# Effects of Nitrogen Emissions on Fish Species Richness across the World’s Freshwater Ecoregions

**DOI:** 10.1021/acs.est.2c09333

**Published:** 2023-05-22

**Authors:** Jinhui Zhou, José M. Mogollón, Peter M. van Bodegom, Valerio Barbarossa, Arthur H. W. Beusen, Laura Scherer

**Affiliations:** † Institute of Environmental Sciences (CML), Leiden University, 2311 EZ Leiden, The Netherlands; ‡ PBL Netherlands Environmental Assessment Agency, 2594 AV The Hague, The Netherlands; § Department of Earth Sciences, Utrecht University, 3584 CS Utrecht, The Netherlands

**Keywords:** biodiversity loss, ecosystem quality, effect factors, eutrophication, life cycle impact assessment, species sensitivity distribution

## Abstract

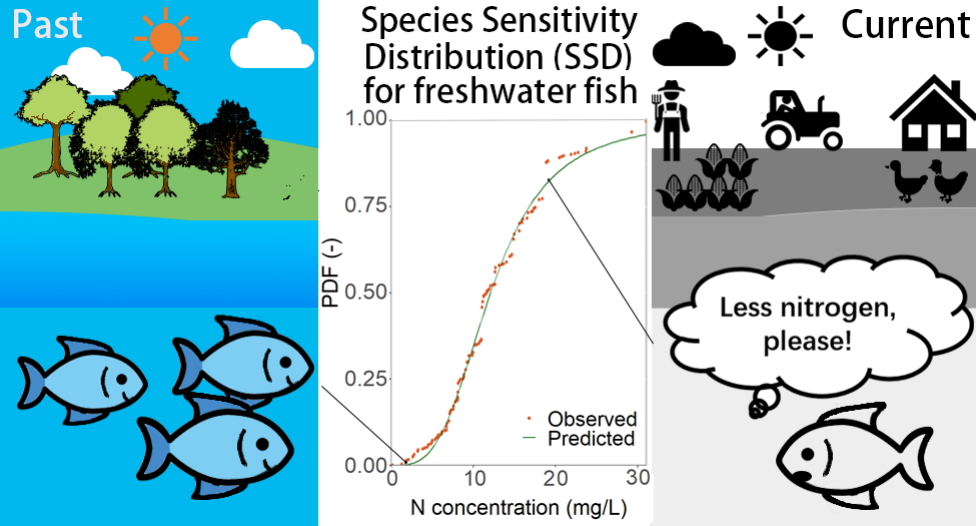

The increasing application of synthetic fertilizer has tripled nitrogen (N) inputs over the 20th century. N enrichment decreases water quality and threatens aquatic species such as fish through eutrophication and toxicity. However, the impacts of N on freshwater ecosystems are typically neglected in life cycle assessment (LCA). Due to the variety of environmental conditions and species compositions, the response of species to N emissions differs among ecoregions, requiring a regionalized effect assessment. Our study tackled this issue by establishing regionalized species sensitivity distributions (SSDs) of freshwater fish against N concentrations for 367 ecoregions and 48 combinations of realms and major habitat types globally. Subsequently, effect factors (EFs) were derived for LCA to assess the effects of N on fish species richness at a 0.5 degree × 0.5 degree resolution. Results show good SSD fits for all of the ecoregions that contain sufficient data and similar patterns for average and marginal EFs. The SSDs highlight strong effects on species richness due to high N concentrations in the tropical zone and the vulnerability of cold regions. Our study revealed the regional differences in sensitivities of freshwater ecosystems against N content in great spatial detail and can be used to assess more precisely and comprehensively nutrient-induced impacts in LCA.

## Abstract 2

Nitrogen emissions threaten freshwater ecosystems through eutrophication and toxicity, but such effects have rarely been studied. This study developed spatially explicit effect factors to assess the effects of nitrogen on freshwater fish biodiversity.

## 1 Introduction

Global food production has tripled in the past five decades to meet the demand of growing populations.^1,2^ This has been achieved using large amounts of synthetic fertilizer for cultivating crops,^3^ importing excessive nitrogen (N) into local nutrient cycles.^4−6^ During the latter half of the 20th century, the global use of nitrogen fertilizers increased 7-fold,^7^ leading to a tripling of nitrogen inputs into freshwater systems.^8^ This input reached approximately 120 Tg of N year^–1^ in 2000. It represents a combination of fertilizer, manure, biological N_2_ fixation, and nitrogen deposition. Future scenarios posit that N inputs will continue to increase due to population growth and the increasing proportions of proteins in human diets.^9−11^

The excessive release of nitrogen into the environment is adversely affecting ecosystems.^12−14^ For instance, N enrichment can induce eutrophication in water bodies and toxicity to species. Eutrophication can cause hypoxia, the severity of which determines the survival of aerobic organisms in water. Moreover, nitrate (NO_3_^–^) in drinking water is not only harmful to human health,^15^ but ammonia (NH_4_), NO_3_^–^, and nitrite (NO_2_^–^) also perturb the pH and become toxic to fish.^16−18^ Many fish species are top predators, and their survival, diversity, and health are good indicators of the functioning of aquatic ecosystems.^19,20^

Life cycle assessment (LCA) can be used to characterize the impact of eutrophication on biodiversity.^23^ LCA is a tool for assessing the environmental impacts of products across their life cycles and can help analyze the trade-offs between economic activities and the environment.^21,22^ Within the life cycle impact assessment (LCIA) phase of an LCA, characterization factors (CFs) express the relative magnitude of a certain environmental impact per unit of the characterized activity.^23^ As an endpoint-level component of CFs, effect factors (EFs) describe the sensitivity of the species community to environmental pressure.^24^ Such EFs can be used to assess the effects of N on freshwater biodiversity.^25^ Cosme and Hauschild^26^ estimated the effect of marine eutrophication-induced hypoxia on species. They used dissolved oxygen (DO) as an intermediate factor (exposure factor) connecting N with effects on species. Azevedo et al.^27^ investigated the patterns of biodiversity along phosphorus (P) concentration gradients in lakes and streams based on limited data from peer-reviewed papers. LC-IMPACT,^28^ ReCiPe2016,^29^ and Jwaideh et al.^30^ applied EFs derived from the P species sensitivity distributions of Azevedo et al.^27^ that directly link to the fate of the nutrient (i.e., without exposure as an intermediate factor). However, Cosme and Hauschild^26^ focused on marine ecosystems and considered only the hypoxia induced by N yet ignored the effect of the toxicity of N on species, while Azevedo et al.^27^ and subsequent phosphorus EF studies have not accounted for N. Furthermore, both of these studies were conducted at a very coarse scale of distinguishing the effects for only four or five global biogeographical regions. In various cases, N contributes strongly to freshwater eutrophication. Globally, 26% of the area with undesirable periphyton growth is limited by N compared with 74% for P. When considering acceptable and undesirable periphyton growth, even 66% of the area is limited by N compared with 34% for P.^31^ However, the effects of N on the ecosystem have not been explored globally.^25,32^

Hydro-climatic and morphological conditions specific to catchments/ecoregions dictate patterns of fish distribution.^33^ Therefore, the response of fish assemblages to human stressors is contingent on these environmental conditions.^33,34^ For instance, variations in water temperature may change dissolved oxygen demand for respiratory purposes for organisms.^35^ Meanwhile, biotic sensitivity to such hypoxia differs between species. Therefore, N-induced eutrophication has divergent effects on diverse species composition across distinct ecoregions.^34,36,37^ Regionalized EFs of fish biodiversity loss are therefore required to describe the impact of N on fish diversity across different ecoregions.

This study aimed to explore the regionalized effects of N on global freshwater ecosystems. On the basis of 41 years of fish occurrence data (covering 13 920 freshwater fish species) and N concentration simulations from the Integrated Model to Assess the Global Environment - Global Nutrient Model (IMAGE-GNM),^38^ we calculated the fish species sensitivity distribution (SSD) over 367 ecoregions and provide EFs of potential N-induced species loss at a half-degree resolution. This study is the first to reveal the statistical relationships between N content and species loss in the global freshwater system.

## 2 Methods

### 2.1 Global Nutrient Model

Measurement data for nutrients in global rivers and lakes are rare, especially for N. Worldwide total N (TN) sampling occurs in roughly one fourth of water quality stations [e.g., 4685 N sampling stations out of more than 18 000 river stations from the Global River Chemistry Database (GLORICH^39^)] and cover few regions [e.g., only 83 countries are covered by N stations in the global water quality database (GEMStat^40^)].

The use of a global nutrient model can fill the gap of a lack of spatial N information, as it can predict the unknown N concentrations in water bodies without sampling.^41−43^ Among recognized global nutrient models, IMAGE-GNM is a spatially explicit, dynamic model with the finest resolution (0.5 degree × 0.5 degree) that has been validated with sampling station data.^44^ The validation of Zhou et al.^45^ found a NRMSE of 2.29 and a Pearson correlation coefficient (*r*) of 0.58 of IMAGE-GNM-generated estimates versus 9770 records of total nitrogen observed data from 1199 global river stations. Of these, the North Frigid Zone performed best in terms of NRMSE (0.57) but had a low *r* (0.14), the North Temperate Zone performed best in terms of *r* (0.59) but had a high NRMSE (2.35), the Torrid Zone had a relatively low NRMSE (1.71) but the lowest *r* (0.05), and the South Temperate Zone generated a relatively high *r* (0.46) but the second highest NRMSE (1.91) (the scale of these regions can be found in Ref. (46)). In this study, we employed IMAGE-GNM to provide global N concentration estimates from 1970 to 2010,^5,38,42^ as these are the most recent years that can be accessed from IMAGE-GNM. A detailed model description of IMAGE-GNM can be found in ref (38).

### 2.2 Freshwater Fish Species Inventory

We compiled point occurrence data for freshwater fish species following the same methodology used by Barbarossa et al.^47,48^ First, we retrieved occurrence data from the Global Biodiversity Information Facility (GBIF),^49^ FishNet network,^50^ SpeciesLink,^51^ Portal da Biodiversidade,^52^ and the Atlas of Living Australia (ALA).^53^ An overview of these source data can be found in Table S1 of ref (47). Second, we coupled the fish occurrence data to freshwater fish species names and associated synonyms, which were derived from Fishbase (we used the R package “rfishbase 4.0.0”^54^ and set FISHBASE_VERSION as “19.04”) and Tedesco et al.^55^ On the basis of these data sets, we harmonized freshwater fish species names and excluded the occurrence records without year and geographic information. This step selected 13 774 unique freshwater fish species with scientific names and 825 species with synonyms. Third, we merged these occurrence records by assigning the scientific names for those with synonyms and removing the duplicates. In total, we obtained 13 920 freshwater fish species and 5 427 740 occurrence records from 1970 to 2010. Details can be seen in the Supporting Information.

### 2.3 Species Sensitivity Distributions

EFs [PDF cubic meters per kilogram (described in section 2.4)] are used within life cycle impact assessment^56^ and typically expressed as the potentially disappeared fraction (PDF) of species, i.e., the relative species richness as a fraction of the total, per unit of an increase in stressor. EFs reflect both the sensitivity of the species (PDF between 0 and 1) and the size of the system being affected (here, volume). EFs can be derived from species sensitivity distributions (SSDs), which represent the continuous relationship between PDF and a stressor. In this case, PDF (dimensionless, eq 1) depends on the loss of species richness under the influence of N concentration levels (milligrams per liter) compared to the maximum species richness that can be observed within ecological units. N can stress ecosystems through both eutrophication and toxicity. As an ecological unit, we used freshwater ecoregions, which are deemed a characteristic, geographically distinct combination of natural communities.^57^ Thus, we set ecoregions as the smallest ecological units to model EF [426 freshwater ecoregions over the globe, data from Freshwater Ecoregions of the World (FEOW)].^57^ We also employed a coarser biogeographical classification by combining the realms and the major habitat types as a supplement to some ecoregions that lack occurrence records (49 realm-major habitat types globally, of which 48 have sufficient data to support regressions). The ecoregions and realm-major habitat types were rasterized before the derivation of SSDs. When the number of pairs of PDF-N concentration data was three or fewer, we deemed it insufficient for fitting SSD curves.
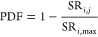
1where SR_*i*,*j*_ is the species richness of ecoregion *i* at N concentration level *j* and SR_*i*,max_ is the maximum species richness in ecoregion *i*.

We extracted the species richness by counting the number of fish species that can survive at a given N concentration level for each ecoregion/realm-major habitat type. We assume that the fish species are tolerant to the prevailing N-induced hypoxia/toxicity up to the N concentration at which they are observed within an ecoregion, whereas fish species richness gradually decreases with an increase in N concentration by exceeding the tolerance levels of the species. This follows an approach similar to that of Gade et al.^61^ for terrestrial acidification. To keep consistency with current LCA practices, the lower tolerance threshold was not considered. The lower tolerance threshold ensures organisms do not starve from a lack of nutrients. This threshold is beyond the scope of this study and may contain a functionally distinct ecosystem from the reference system.^25^ Therefore, we consider only the upper threshold of the stressor.

By matching the species occurrences with N concentration of the same year and location (by pixels of 0.5 degree × 0.5 degree), we derived the N tolerance thresholds for each species within each ecoregion/realm-major habitat type.

We can predict SSD curves with a logistic function (eq 2) to fit the data pairs of PDF (calculated in eq 1) and N concentration. This function is widely used in LCIA method development.^61−62^
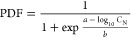
2where *a* and *b* are empirical coefficients, *a* indicates the N concentration at which 50% of the species have disappeared, and *b* can be interpreted as the slope of the SSD. *C*_N_ (milligrams per liter) is the N concentration.

We evaluated the performance of the regression using the pseudo-*R*^2^ value and the normalized root-mean-square error (NRMSE). Normal *R*^2^ values for linear regressions have been shown to be inappropriate for nonlinear fits.^63^ We, therefore, selected Cox–Snell pseudo-*R*^2^, one of the most commonly used *R*^2^ for nonlinear regressions.^64^ This index compares the likelihood ratio of the fitting function model to a null model that contains only the intercept. NRMSE discloses the magnitude of the errors normalized by the average value through division. We regarded as a good fit a Cox–Snell pseudo-*R*^2^ of >0.5 and a NRMSE of <1, following Scherer et al.^62^ and nutrient model research.^45,65^

### 2.4 Effect Factors

Two main approaches can be used to calculate EFs: marginal and average.

In this study, we employed both approaches to show different perspectives of the effects of N on fish biodiversity. The marginal approach (EF_marginal_, PDF cubic meters per kilogram, eq 3) denotes the instantaneous change of effect due to the current stressor and is calculated as the derivative of the SSD (i.e., eq 2) at the current state.
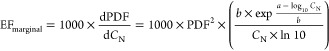
3where 1000 is the coefficient to convert the units of the reciprocal of *C*_N_ from liters per milligram to cubic meters per kilogram.

The average approach (EF_average_, PDF cubic meters per kilogram, eq 4) represents the long-term change in the effect, comparing the current state with a desired reference state. This could be a state without anthropogenic interference, a political target, or a zero effect, and in this study, we took the year 1900 as the reference year, with the N concentrations taken again from IMAGE-GNM. The average approach has also been used to assess the effect of future states under different greenhouse gas concentration trajectories.^62,66^
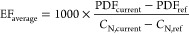
4We calculated globally marginal and average EFs based on the gridded N concentration at the current state (represented by the year 2010) at a resolution of 0.5 degree × 0.5 degree and the SSDs for the corresponding ecoregion/realm-major habitat type. EFs were derived from ecoregion-level SSDs first, and the realm-major habitat type was employed only if the species–stressor information was not sufficient for the ecoregions.

In those regions with zero N concentration, EFs were set to no value because no SSD could be derived. We also regarded a N concentration of <0.0001 mg L^–1^ as zero N concentration due to the uncertainty in measurements and modeling.

## 3 Results

### 3.1 Species Sensitivity Distributions and Potentially Disappeared Fractions

**Figure 1:**
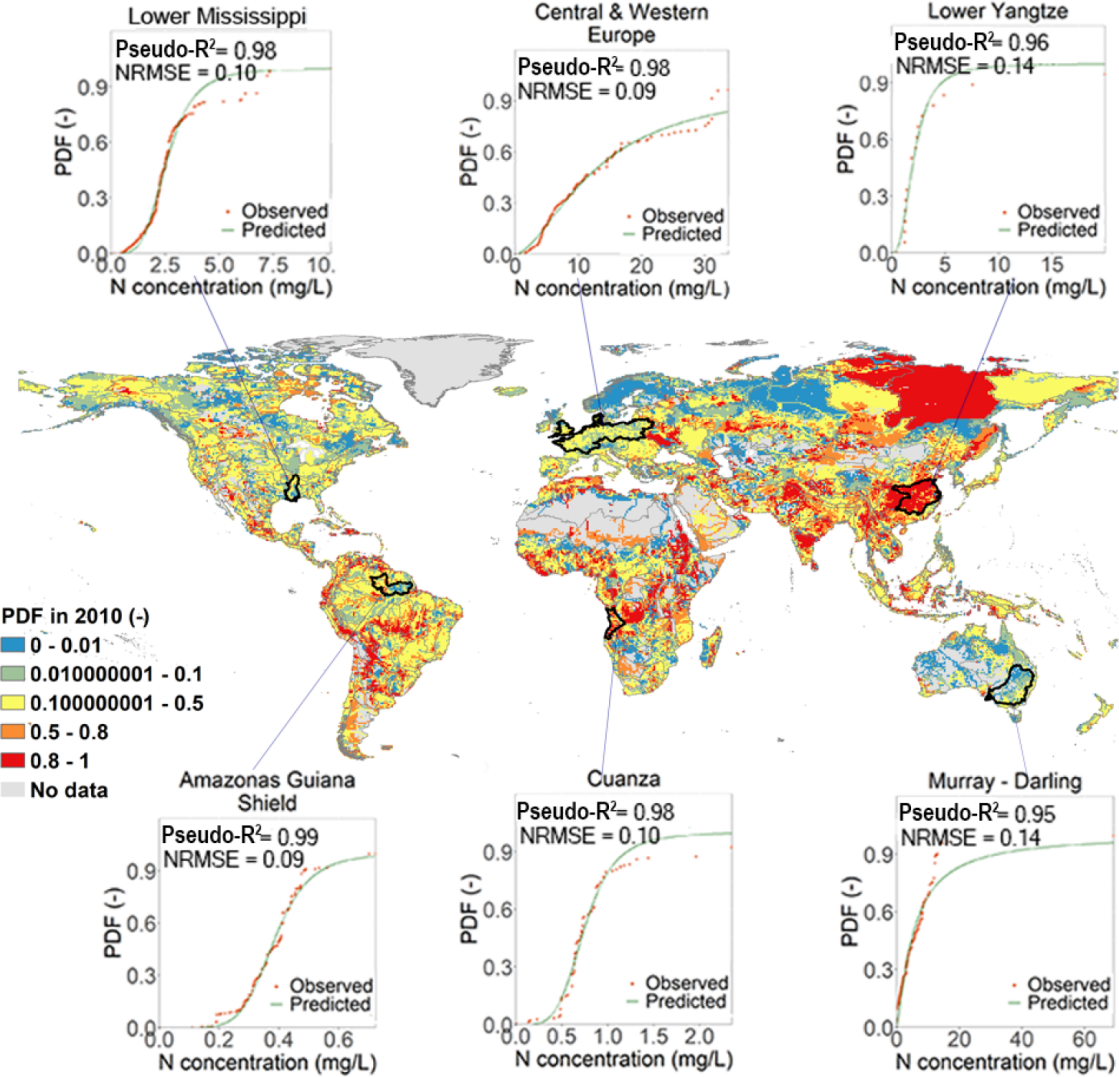
Potentially disappeared fraction (PDF) of the current fish species diversity (2010) at a 0.5 degree × 0.5 degree resolution and species sensitivity distributions (SSDs, following eq 2) for examples of large ecoregions on six continents. Note that the right limit is contained, while the left limit is not contained in the segment; e.g., 0–0.01 means 0 < PDF ≤ 0.01. The SSD plots of all ecoregions can be found in Supporting Information 2.

Among the 426 ecoregions, SSD curves could be derived for 367 ecoregions, and all of them performed well (Cox–Snell pseudo-*R*^2^ > 0.5, and NRMSE < 1). Data from 22 ecoregions were insufficient to fit SSD curves (no more than three PDF-N concentration data pairs), and 37 ecoregions did not have any data. The minimum Cox–Snell pseudo-*R*^2^ was 0.57, found for Lake Tanganyika (NRMSE = 0.36), and the maximum NRMSE equaled 0.55, found for Chuya (Cox–Snell pseudo-*R*^2^ = 0.75). In total, the 367 analyzed ecoregions occupy 95% of the global area. Among them, 357 ecoregions had a Cox–Snell pseudo-*R*^2^ of >0.8 and a NRMSE of <0.4 (maps of Cox–Snell pseudo-*R*^2^ and NRMSE can be found in Figures S3 and S4). Ecoregion-level SSD plots can be found in Supporting Information 2, and the SSD curves of six of the large ecoregions of different continents are shown in Figure 1 as examples.

For those 59 ecoregions without an ecoregion-level regression, we provided the realm-major habitat type-level SSDs. With these SSDs, we can fill the gaps of 58 of these ecoregions (Cox–Snell pseudo-*R*^2^ > 0.5, and NRMSE < 1). The remaining ecoregion Bermuda, which belongs to Nearctic-Oceanic Islands, had data for neither the ecoregion level nor the realm-major habitat type level. The 58 SSD plots for the realm-major habitat type can be found in Supporting Information 3, and an overview of regression coefficients, criteria, area, etc., of ecoregions and realm-major habitat type are listed in Supporting Information 4.

Figure 1 illustrates the PDF of the current state (2010). The regions with zero N concentration and consequently no value for PDF occupy 14% of the global area. These regions are remote and included several lakes and arid zones (e.g., Sahel Desert and Australian deserts). In the regions with non-zero N concentration, ∼15% of the area is at severe risk of the potential disappearance of the local fish communities (PDF > 0.8). Among these, the high PDF of ecoregions may be caused by high N emissions to freshwater over the years due to a large increase in population density (e.g., Lower Yangtze River in China and Southern Deccan Plateau in India). Conversely, the high PDF of a few regions in polar freshwater systems may result from the uncertainty raised by a lack of observational data; e.g., fish biodiversity of Lena and Taimyr showed high sensitivities to N content because all of the occurrence was recorded at low N concentrations. The changes in PDF and N concentration can be found in Figures S5 and S6.

### 3.2 Effect Factors

**Figure 2:**
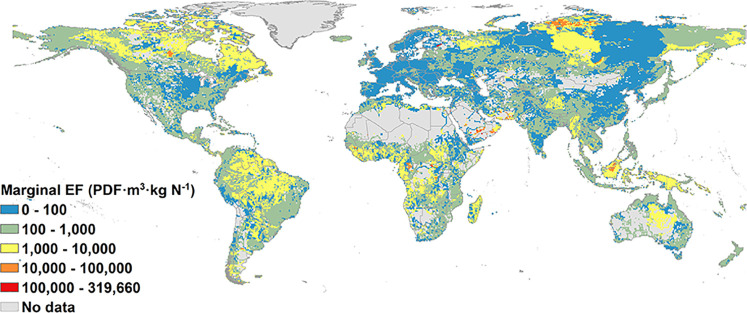
Marginal effect factors (EFs) at a 0.5 degree × 0.5 degree resolution. The boundaries of the background map represent countries. Note that the right limit is contained, while the left limit is not contained in the segment; e.g., 0–100 means 0 < EF ≤ 100.

**Figure 3:**
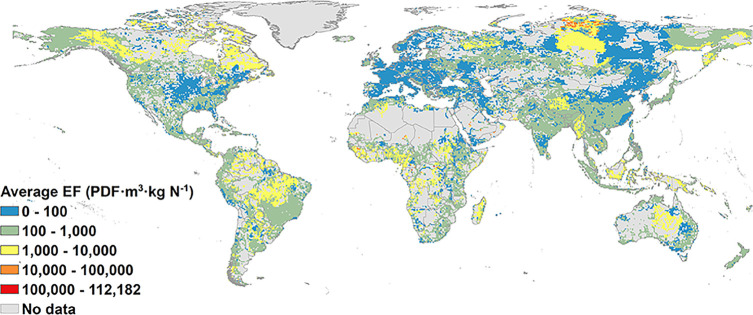
Average effect factors (EFs) at a 0.5 degree × 0.5 degree resolution. The boundaries of the background map represent countries. Note that the right limit is contained, while the left limit is not contained in the segment; e.g., 0–100 means 0 < EF ≤ 100.

Marginal EFs and average EFs showed similar spatial patterns (Figures 2 and 3). High values for marginal and average EFs (>100 000 PDF m^3^ kg^–1^) occurred in <1% of the non-zero N concentration area and were distributed in, e.g., Taimyr, Arabian Interior, Baluchistan, Borneo Highlands, and Sangha. Despite the similarity, marginal EFs include slightly more areas with high values (>100 000 PDF m^3^ kg^–1^) and low values (≤100 PDF m^3^ kg^–1^); i.e., 33% had low values and 1.2% high values, while the average EF had 29% and 0.6% of low and high values, respectively.

The reason for more areas with high values for marginal EFs is that the current state is at a stage of a rapid increase in PDF, while the average EFs smoothen the change of effect by taking the difference between the current and desired state. In particular, the marginal EF showed an accelerated species sensitivity at PDF ≤ 0.5 (occupying 70% of the area).

The higher percentage of low values in marginal EFs was caused by situations in which PDF approaches 0 and 1, as, at these stages, little change in species richness can happen under the perturbation of the N content in water. When the current PDF is close to 1, average EFs can show higher values than marginal EFs, as long as N concentrations varied from 1900 to 2010 in those regions. For instance, an average EF of >100 PDF m^3^ kg^–1^ can be found in some pixels in the Middle and Lower Yangtze River, Southern Deccan Plateau, and Amazonas High Andes, compared to their marginal EF close to 0. The average EF of some regions in Lena was <100 PDF m^3^ kg^–1^ due to the small increase in N concentration during the past 110 years.

## 4 Discussion

This study is the first to showcase a regionalized (ecoregion-level) relationship between freshwater species loss and N concentrations. Such information can provide more local support for assessing the impacts of eutrophication and ecotoxicity on local biodiversity.

Our study also provides the EFs at a resolution (0.5 degree × 0.5 degree) much finer than what is common in previous studies, such as Cosme and Hauschild,^26^ LC-IMPACT,^28^ and ReCiPe2016.^29^ It allows for providing more detailed information about the local ecosystems and supports future research on assessing the impacts of nutrient emissions on biodiversity, for instance, when integrated into life cycle impact assessments.

Because no study established EFs or sensitivity to N inputs for freshwater species, we compared the spatial variability of our results with EFs for marine eutrophication^26^ and the sensitivity of freshwater fish to P.^27^ In all of these studies, N and P emissions are tied to densely populated regions and induce eutrophication (and toxicity) downstream in both freshwater and marine ecosystems. In line with marine EFs for N estimated by Cosme and Hauschild,^26^ our EFs for N for freshwater fish are higher in tropical regions than in temperate zones. Our results also agreed with their EFs patterns showcasing an increasing trend from the polar to the tropical regions for Eurasia, Africa, and South America. However, in some polar regions (North America and North Asia, namely the Western Hudson Bay, Lena, and Taimyr regions) our results find higher EFs in the polar region than in the temperate zone. These latter results agree with those of Azevedo et al.,^27^ who posit that heterotrophic species are more sensitive to nutrient concentrations in cold regions because these species are adapted to low nutrient concentrations. These studies may result from a larger uncertainty in the SSDs for polar regions due to fewer species occurrence data . These findings highlight the need to better assess the effects of high concentrations on species loss in the tropical zone, while at the same time, the vulnerability of species in cold regions should also be considered.

Our result for the current PDF states reflects the environmental threshold of N concentrations for decreasing freshwater fish biodiversity. It shows a spatial pattern similar to that of the regional boundaries for N surplus,^67^ which were also derived from IMAGE-GNM. A consensus about the most severe N exceedances exists in India/Pakistan, eastern China, the Nile Basin, areas in Saudi Arabia, and areas along the Peruvian coast. From our results and the comparison with other studies, it follows that using a finer scale allows for describing the nutrient effects on species in more detail but also influences the reflection of the realistic species sensitivity. Using broader regions erases the geographical distinction of natural community responses to the various environments in smaller regions and therefore overestimates the effect for hyposensitive ecosystems and underestimates the effect for hypersensitive ecosystems. Thus, we recommend calculating the SSDs for ecoregions, unless insufficient data precludes this calculation. In such instances we advise the use of realm-and-major-habitat-level SSDs.

An underlying assumption of this study is that fish species loss is tied to the increase in the N concentrations. However, the individual N limitation may also be affected by the co-limiting effects of N and P under the influence of eutrophication.^31,68^ Our method may overestimate N effects because the disappearance of fish species in some areas is co-affected by other stressors such as P. As McDowell et al.^31^ found, 66% of the global freshwater system is limited by N. Even though the loss of species induced by P limitations or other stressors might have influenced the species occurrence at a certain N concentration at some locations, the species may still be observed at the same N concentration at other locations within the same ecoregion and would, therefore, be considered tolerant. This approach undermines the effect of co-stressors. For future studies, we suggest evaluating the effects on fish species richness by simultaneously considering other human pressures and especially the co-limitation effects due to P emissions. Observations may, be coupled to models of global P fate,^38^ land-use change interactions,^69^ global warming impact,^48,69^ and water consumption threats to freshwater fish communities.^70^ Such a coupling will help to further disentangle the direct impacts of nitrogen loads and will help in understanding whether and how its effects interact with other biodiversity threats.

Furthermore, the species sensitivity relations could be refined by increasing the number of species observations and the quality of global N predictions. Another source of uncertainty lies in potential sampling bias for our underlying point occurrence data set. For instance, our study encountered the same problem of lacking occurrence data as the previous studies in cold regions, whereby the species sensitivity could be overestimated due to the underestimation of species richness. The accessibility to more species occurrence records can decrease the uncertainty in some ecoregions due to a lack of data, e.g., Lena and Taimyr in polar freshwater systems. The accuracy of IMAGE-GNM is tied to the uncertainty introduced in N concentration predictions and can be affected by various reasons such as model inputs, retention models, and hydrological parameters. The predictions of N concentration can be improved by using a mechanistic model, such as IMAGE-DGNM.^71^ The current version of IMAGE-DGNM has been applied for several watersheds, and a future version of global N modeling can better support the research on the N-induced impact on fish biodiversity.^71^

Last but not least, we used 1900 as the reference year, as it is the earliest simulated year in IMAGE-GNM, but ideally, the reference state could reflect the preindustrial levels.

In conclusion, our study quantified the regional relationships between N enrichment in freshwater and fish species loss, which complements the current freshwater eutrophication studies based on P in LCA. The regionalized freshwater SSDs and EFs reveal the sensitivities of ecosystems to nutrient emissions at a fine resolution. They can be applied to assess the spatially differentiated biodiversity impacts of N emissions over the world in LCA.

## Supporting Information Available

A global map of freshwater ecoregions, a global map of occurrence records of freshwater fish species, an overview of the pairwise species occurrence–N concentration data for all of the ecoregions, pseudo-*R*^2^ and NRMSE values of SSDs, and changes in PDF and N concentration between current and reference year (PDF)
Archive of images of the SSDs for ecoregions mentioned in section 3.1 (ZIP)
Archive of images of the SSDs for the realm-major habitat types mentioned in section 3.1 (ZIP)
Overview of regression coefficients, criteria, area, etc., of ecoregions and realm-major habitat types mentioned in section 3.1 (XLSX)
