# Supplementary material for: Effects of Nitrogen Emissions on Fish Species Richness across the World’s Freshwater Ecoregions
Source: Environ Sci Technol. 2023 May 22;57(22):8347–54. doi: 10.1021/acs.est.2c09333 (PMC10249400; doi:10.1021/acs.est.2c09333)
Supplement: Supplementary file 1 — es2c09333_si_001.pdf [file es2c09333_si_001.pdf]

Supporting information

Effects of nitrogen emissions on fish species richness across the world's freshwater ecoregions

Jinhui Zhou, José M. Mogollón, Peter M. van Bodegom, Valerio Barbarossa, Arthur H. W. Beusen, Laura Scherer

Summary of supporting information 1:

14 Pages

6 figures

1 table

This document shows (1) a global map of freshwater ecoregions; (2) a global map of occurrence records of freshwater fish species; (3) an overview of the pairwise species occurrence-N concentration data for all the ecoregions mentioned in section 2.2; (4) Pseudo-R^2 and Normalized root mean square error (NRMSE) of the SSDs; and (5) changes in potentially disappeared fraction (PDF) and N concentration between current and reference year.

S1. Global map of freshwater ecoregions

We show a map of freshwater ecoregions, which can also be found in Larentis et al.[1].

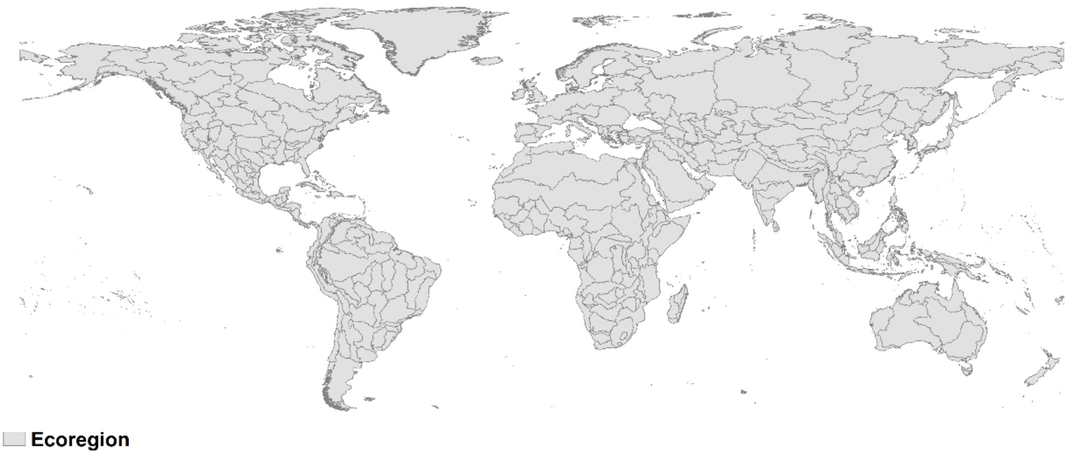

Figure S1 Freshwater ecoregions

S2. Occurrence records of freshwater fish species

We collected the occurrence records of freshwater fish species from 1970 to 2010, counted the numbers of the records during these 41 years, and rasterized them to a 0.5°×0.5° resolution (Figure S2). Fishes defined as freshwater species can be observed in freshwater, coastal, and

marine ecosystems during their different life stages. We excluded the occurrences recorded in coastal and marine ecosystems when we matched the occurrence records with N concentration data in the freshwater.

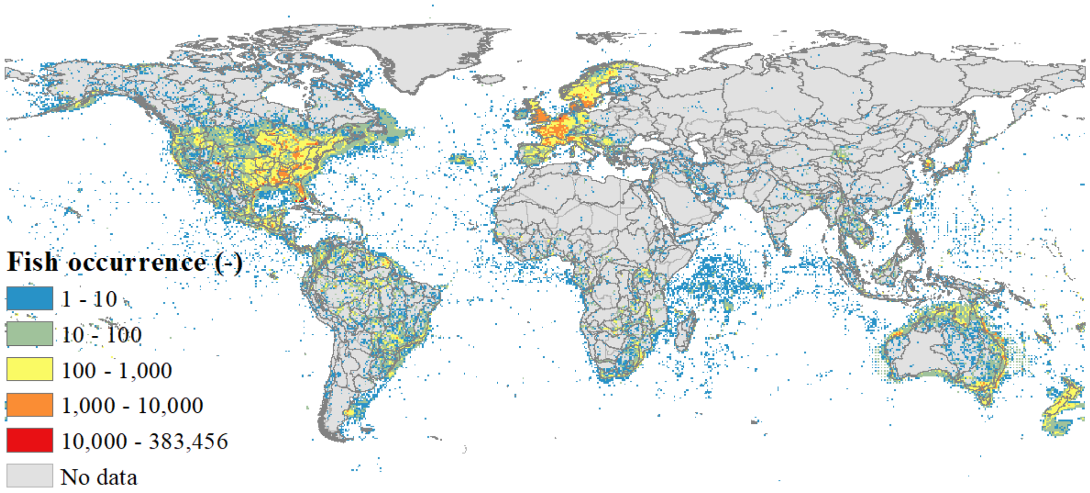

Figure S2 Occurrence records of freshwater fish species at a resolution of 0.5°×0.5°. The polygons illustrate freshwater ecoregions.

S3. Overview of data across ecoregions

Table S1 shows the number of pairs of freshwater fish occurrence and N concentration data in ecoregions. Note that we showed 5,427,740 records in Figure S2, whereas only 3,949,797 records were used for calculating SSDs and effect factors due to the exclusion of coastal and marine areas.

Table S1 Overview of pairwise species occurrence-N concentration data at the ecoregion level

FEOW ID	Ecoregion name	Number of fish occurrence data	Number of fish species-N threshold data
101	Alaskan Coastal	4861	33
102	Upper Yukon	420	20
103	Alaska & Canada Pacific Coastal	8245	76
104	Upper Mackenzie	1307	30
105	Lower Mackenzie	3868	33
106	Central Arctic Coastal	566	20
107	Upper Saskatchewan	1552	36
108	Middle Saskatchewan	503	24
109	English - Winnipeg Lakes	4859	69
110	Southern Hudson Bay	2074	46
111	Western Hudson Bay	1028	22
112	Canadian Arctic Archipelago	1605	16
113	Eastern Hudson Bay - Ungava	1864	24

FEOW ID	Ecoregion name	Number of fish occurrence data	Number of fish species-N threshold data
114	Gulf of St.Lawrence Coastal Drainages	498	15
115	Canadian Atlantic Islands	1529	28
116	Laurentian Great Lakes	230906	132
117	St.Lawrence	12545	78
118	Northeast US & Southeast Canada Atlantic Drainages	40224	168
119	Scotia - Fundy	1882	36
120	Columbia Glaciated	6803	52
121	Columbia Unglaciated	19850	67
122	Upper Snake	5220	34
123	Oregon & Northern California Coastal	9855	71
124	Oregon Lakes	975	36
125	Sacramento - San Joaquin	14773	89
126	Lahontan	1185	57
127	Bonneville	1894	47
128	Death Valley	611	37
129	Vegas - Virgin	318	33
130	Colorado	55586	100
131	Gila	2381	52
132	Upper Rio Grande - Bravo	11466	43
133	Pecos	9547	52
135	Lower Rio Grande - Bravo	6046	73
137	Rio Salado	1458	26
138	Rio San Juan (Mexico)	1297	31
139	West Texas Gulf	87983	81
140	East Texas Gulf	283859	168
141	Sabine - Galveston	48100	135
142	Upper Missouri	16233	89
143	Middle Missouri	17208	102
144	US Southern Plains	25086	112
145	Ouachita Highlands	17341	107
146	Central Prairie	26767	114
147	Ozark Highlands	15271	111
148	Upper Mississippi	84641	126
149	Lower Mississippi	73993	209
150	Teays - Old Ohio	77039	156
151	Cumberland	7447	81
152	Tennessee	52924	185

FEOW ID	Ecoregion name	Number of fish occurrence data	Number of fish species-N threshold data
153	Mobile Bay	87011	181
154	West Florida Gulf	53317	139
155	Apalachicola	29054	139
156	Florida Peninsula	361518	191
157	Appalachian Piedmont	77499	258
158	Chesapeake Bay	37603	138
159	Southern California Coastal - Baja California	23391	183
160	Sonora	4347	81
161	Guzman - Samalayuca	3283	51
162	Sinaloa	2793	94
163	Mayran - Viesca	449	36
164	Rio Santiago	1930	66
165	Lerma - Chapala	1482	34
166	Llanos El Salado	46	9
167	Panuco	9420	131
168	Ameca - Manantlan	6130	78
169	Rio Balsas	2898	70
170	Sierra Madre del Sur	5123	71
171	Papaloapan	4193	78
173	Grijalva - Usumacinta	8206	90
174	Upper Usumacinta	7111	51
175	Yucatan	7543	75
201	Chiapas - Fonseca	6857	93
202	Quintana Roo - Motagua	13202	148
203	Mosquitia	1090	28
204	Estero Real - Tempisque	622	28
205	San Juan (Nicaragua/Costa Rica)	2223	83
206	Chiriqui	1753	40
207	Isthmus Caribbean	934	53
208	Santa Maria	1429	39
210	Rio Tuira	2290	90
211	Cuba - Cayman Islands	145	19
212	Bahama Archipelago	620	40
213	Jamaica	148	25
214	Hispaniola	879	46
215	Puerto Rico - Virgin Islands	1066	50
216	Windward & Leeward Islands	2142	37
301	North Andean Pacific Slopes - Rio Atrato	2519	149

FEOW ID	Ecoregion name	Number of fish occurrence data	Number of fish species-N threshold data
302	Magdalena - Sinu	8301	221
303	Maracaibo	489	56
304	South America Caribbean Drainages - Trinidad	2919	143
305	Orinoco High Andes	365	60
306	Orinoco Piedmont	863	73
307	Orinoco Llanos	12262	216
308	Orinoco Guiana Shield	11445	156
309	Orinoco Delta & Coastal Drainages	3078	58
310	Essequibo	1996	47
311	Guianas	17067	135
312	Amazonas High Andes	229	41
313	Western Amazon Piedmont	1848	87
314	Rio Negro	4338	143
315	Amazonas Guiana Shield	5750	94
316	Amazonas Lowlands	27497	373
317	Ucayali - Urubamba Piedmont	785	41
318	Mamore - Madre de Dios Piedmont	4389	108
319	Guapore - Itenez	2947	62
320	Tapajos - Juruena	1952	66
321	Madeira Brazilian Shield	2854	115
322	Xingu	1419	67
323	Amazonas Estuary & Coastal Drainages	4966	184
324	Tocantins - Araguaia	2932	162
325	Parnaiba	804	42
326	Northeastern Caatinga & Coastal Drainages	845	51
327	S. Francisco	1534	82
328	Northeastern Mata Atlantica	7151	216
329	Paraiba do Sul	1799	46
330	Ribeira de Iguape	2578	68
331	Southeastern Mata Atlantica	987	62
332	Lower Uruguay	773	60
333	Upper Uruguay	454	44
334	Laguna dos Patos	5291	131
335	Tramandai - Mampituba	1212	51
336	Central Andean Pacific Slopes	156	40

FEOW ID	Ecoregion name	Number of fish occurrence data	Number of fish species-N threshold data
337	Titicaca	59	11
338	Atacama	37	8
339	Mar Chiquita - Salinas Grandes	8	6
340	Cuyan - Desaguadero	36	8
341	South Andean Pacific Slopes	276	36
342	Chaco	434	32
343	Paraguay	7276	175
344	Upper Parana	19320	334
345	Lower Parana	655	71
346	Iguassu	1717	105
347	Bonaerensean Drainages	160	15
348	Patagonia	5690	48
349	Valdivian Lakes	91	17
350	Galapagos Islands	959	31
352	Fluminense	2448	95
401	Iceland - Jan Mayen	41	1
402	Northern British Isles	29763	49
403	Cantabric Coast - Languedoc	52546	71
404	Central & Western Europe	646580	111
405	Norwegian Sea Drainages	31698	34
406	Northern Baltic Drainages	300037	52
407	Barents Sea Drainages	11054	17
408	Southern Baltic Lowlands	73	16
409	Lake Onega - Lake Ladoga	79	5
410	Volga - Ural	45	13
411	Western Caspian Drainages	1	1
412	Western Iberia	5154	32
413	Southern Iberia	3912	43
414	Eastern Iberia	16324	65
415	Gulf of Venice Drainages	40145	65
416	Italian Peninsula & Islands	17958	65
417	Upper Danube	7182	69
418	Dniester - Lower Danube	10175	74
419	Dalmatia	1339	68
420	Southeast Adriatic Drainages	803	27
421	Ionian Drainages	275	22
422	Vardar	911	41
423	Thrace	1288	55
424	Aegean Drainages	510	34
425	Dnieper - South Bug	18	8

FEOW ID	Ecoregion name	Number of fish occurrence data	Number of fish species-N threshold data
426	Crimea Peninsula	1	1
427	Don	2	2
428	Kuban	1	1
429	Western Anatolia	115	13
430	Northern Anatolia	31	8
431	Central Anatolia	9	5
432	Southern Anatolia	40	17
433	Western Transcaucasia	9	9
434	Kura - South Caspian Drainages	42	6
435	Sinai	12	2
436	Coastal Levant	28	7
437	Orontes	184	15
438	Jordan River	580	39
439	Southwestern Arabian Coast	359	39
440	Arabian Interior	898	46
441	Lower Tigris & Euphrates	265	28
442	Upper Tigris & Euphrates	801	57
443	Oman Mountains	375	11
444	Lake Van	12	3
445	Orumiyeh	39	8
446	Caspian Highlands	50	7
447	Namak	71	13
448	Kavir & Lut Deserts	39	16
449	Esfahan	22	9
450	Turan Plain	48	11
451	Northern Hormuz Drainages	147	22
452	Caspian Marine	219	7
453	Volga Delta - Northern Caspian Drainages	8	2
501	Atlantic Northwest Africa	7	2
502	Mediterranean Northwest Africa	49	15
503	Sahara	174	15
504	Dry Sahel	345	13
505	Lower Niger - Benue	1177	39
506	Niger Delta	56	13
507	Upper Niger	662	19
508	Inner Niger Delta	66	2
509	Senegal - Gambia	7809	66
510	Fouta - Djalon	52	7
511	Northern Upper Guinea	308	27

FEOW ID	Ecoregion name	Number of fish occurrence data	Number of fish species-N threshold data
512	Southern Upper Guinea	51	14
514	Eburneo	2657	33
515	Ashanti	177	20
516	Volta	2678	35
517	Bight Drainages	1327	39
518	Northern Gulf of Guinea Drainages - Bioko	652	22
519	Western Equatorial Crater Lakes	397	20
520	Lake Chad	171	11
521	Lake Victoria Basin	7661	67
522	Upper Nile	1284	29
523	Lower Nile	143	11
524	Nile Delta	55	9
525	Ethiopian Highlands	273	16
526	Lake Tana	416	10
527	Western Red Sea Drainages	91	13
528	Northern Eastern Rift	102	17
529	Horn of Africa	117	15
530	Lake Turkana	114	14
531	Shebelle - Juba	67	16
532	Ogooue - Nyanga - Kouilou - Niari	1299	49
533	Southern Gulf of Guinea Drainages	2569	56
534	Sangha	1236	17
535	Sudanic Congo - Oubangi	1557	27
536	Uele	84	3
537	Cuvette Centrale	255	16
540	Upper Congo	1217	10
541	Albertine Highlands	21	4
542	Lake Tanganyika	751	16
543	Malagarasi - Moyowosi	387	15
544	Bangweulu - Mweru	1748	27
545	Upper Lualaba	422	14
546	Kasai	80	16
547	Mai Ndombe	41	6
550	Lower Congo	1303	46
551	Cuanza	572	52
552	Namib	439	41
553	Etosha	596	18

FEOW ID	Ecoregion name	Number of fish occurrence data	Number of fish species-N threshold data
554	Karstveld Sink Holes	21	6
555	Zambezian Headwaters	1410	28
556	Upper Zambezi Floodplains	3732	29
557	Kafue	362	16
558	Middle Zambezi - Luangwa	1063	43
559	Lake Malawi	2040	15
560	Zambezian Highveld	233	25
561	Lower Zambezi	523	13
563	Eastern Zimbabwe Highlands	256	12
564	Coastal East Africa	8787	138
565	Lake Rukwa	1085	15
566	Southern Eastern Rift	38	6
567	Tana, Athi & Coastal Drainages	611	53
568	Pangani	302	12
569	Okavango	6830	44
570	Kalahari	240	16
571	Southern Kalahari	106	12
572	Western Orange	665	12
573	Karoo	99	12
575	Southern Temperate Highveld	3500	139
576	Zambezian Lowveld	18015	201
577	Amatolo - Winterberg Highlands	1800	80
578	Cape Fold	4070	99
579	Western Madagascar	133	20
580	Northwestern Madagascar	572	29
581	Madagascar Eastern Highlands	147	18
582	Southern Madagascar	30	9
583	Madagascar Eastern Lowlands	521	30
586	Mascarenes	430	26
602	Ob	45	14
604	Chuya	41	4
605	Yenisei	34	11
606	Lake Baikal	46	9
607	Taimyr	13	5
608	Lena	28	7
609	Kolyma	13	2
610	Anadyr	10	6
611	East Chukotka	9	3
612	Koryakia	4	2
613	Kamchatka & Northern Kurils	418	11

FEOW ID	Ecoregion name	Number of fish occurrence data	Number of fish species-N threshold data
614	Okhotsk Coast	39	4
615	Coastal Amur	4	1
616	Lower Amur	284	8
617	Middle Amur	5	3
618	Argun	16	4
619	Shilka (Amur)	19	5
620	Songhua Jiang	3	1
621	Inner Mongolia Endorheic Basins	2333	26
622	Western Mongolia	18	5
623	Dzungaria	87	5
624	Balkash - Alakul	21	11
625	Tarim	881	23
628	Northern Central Asian Highlands	9	1
629	Aral Sea Drainages	5	4
630	Middle Amu Darya	1	1
631	Upper Amu Darya	38	11
632	Qaidan	109	7
633	Upper Huang He	423	8
634	Upper Huang He Corridor	283	12
636	Lower Huang He	54	5
637	Liao He	6	2
638	Eastern Yellow Sea Drainages	5944	36
639	Southeastern Korean Peninsula	4152	41
640	Hamgyong - Sanmaek	193	10
641	Sakhalin, Hokkaido, & Sikhote - Alin Coast	1869	40
642	Honshu - Shikoku - Kyushu	23088	185
701	Baluchistan	144	15
702	Helmand - Sistan	41	8
703	Lower & Middle Indus	782	23
704	Yaghistan	7	3
705	Indus Himalayan Foothills	39	10
706	Upper Indus	9	3
707	Tibetan Plateau Endorheic Drainages	93	15
708	Narmada-Tapti	158	10
709	Ganges Delta & Plain	889	48
710	Ganges Himalayan Foothills	2620	30
711	Upper Brahmaputra	224	17

FEOW ID	Ecoregion name	Number of fish occurrence data	Number of fish species-N threshold data
712	Middle Brahmaputra	567	22
713	Northern Deccan Plateau	248	22
714	Southern Deccan Plateau	81	5
715	Western Ghats	385	52
716	Southeastern Ghats	208	21
717	Sri Lanka Dry Zone	186	17
718	Sri Lanka Wet Zone	270	22
719	Chin Hills - Arakan Coast	1003	31
720	Sitang - Irawaddy	859	62
721	Upper Salween	323	19
722	Lower & Middle Salween	234	22
724	Upper Lancang (Mekong)	192	12
726	Lower Lancang (Mekong)	704	36
727	Khorat Plateau (Mekong)	2956	68
728	Kratie - Stung Treng (Mekong)	560	17
729	Mekong Delta	1886	41
730	Southern Annam	1035	44
731	Eastern Gulf of Thailand Drainages	560	21
732	Chao Phraya	1382	107
733	Mae Khlong	699	46
734	Malay Peninsula Eastern Slope	1410	109
735	Northern Central Sumatra - Western Malaysia	1387	73
736	Aceh	300	12
737	Indian Ocean Slope of Sumatra & Java	64	12
738	Southern Central Sumatra	318	39
739	Southern Sumatra - Western Java	1161	39
740	Central & Eastern Java	315	15
741	Kapuas	548	20
742	Northwestern Borneo	1366	39
743	Borneo Highlands	1109	17
744	Northeastern Borneo	649	45
745	Eastern Borneo	353	30
746	Southeastern Borneo	654	32
747	Malukku	1237	29
748	Lesser Sunda Islands	1879	85
749	Sulawesi	830	30
752	Mindanao	205	16

FEOW ID	Ecoregion name	Number of fish occurrence data	Number of fish species-N threshold data
755	Northern Philippine Islands	4363	94
756	Palawan - Busuanga - Mindoro	1606	22
757	Western Taiwan	2338	98
758	Eastern Taiwan	3081	116
759	Hainan	218	17
760	Northern Annam	171	18
761	Song Hong	230	26
763	Xi Yiang	970	96
764	Upper Yangtze	93	20
765	Middle Yangtze	58	29
766	Lower Yangtze	25	16
767	Coastal Fujian - Zeijang	72	17
768	Andaman Islands	34	4
801	Southwestern Australia	5341	79
802	Pilbara	4331	136
803	Kimberley	1538	62
804	Paleo	3437	89
805	Arafura - Carpentaria	29706	216
806	Lake Eyre Basin	1481	49
807	Eastern Coastal Australia	61882	254
808	Murray - Darling	45678	116
809	Bass Strait Drainages	35483	104
810	Southern Tasmania	16141	64
811	New Zealand	89394	85
812	Vogelkop - Bomberai	786	72
813	New Guinea North Coast	883	40
814	New Guinea Central Mountains	160	16
815	Southwest New Guinea - Trans-Fly Lowland	2565	101
816	Papuan Peninsula	1755	74
817	Bismarck Archipelago	2615	34
818	Solomon Islands	1267	40
819	Vanuatu	1559	28
820	New Caledonia	8777	63
821	Fiji	3310	48
823	Samoas	245	6
828	Hawaiian Islands	851	61

S4. Pseudo-R^2 and NRMSE of SSDs

We show maps of pseudo-R^2 and normalized root mean square error (NRMSE) in Figures S3

and S4.

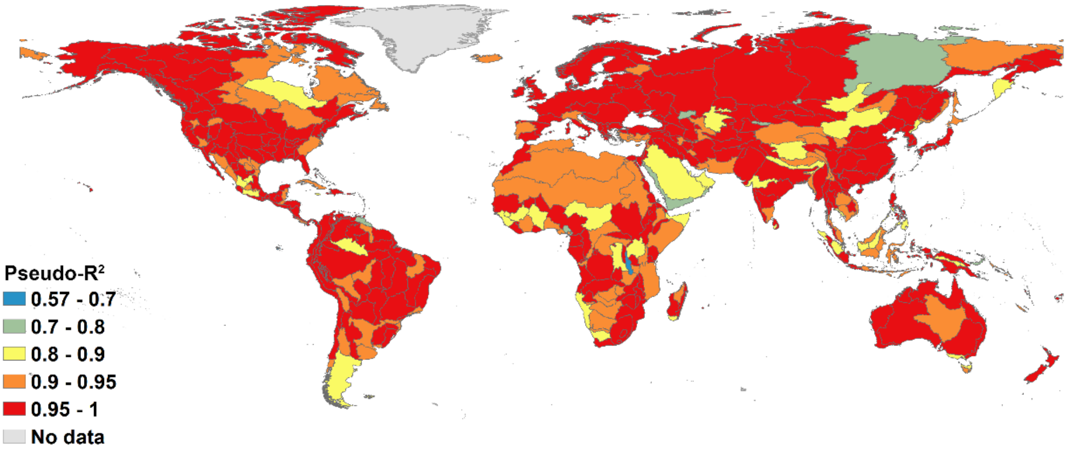

Figure S3 Cox-Snell pseudo-R^2 of species sensitivity distributions (SSDs) at the ecoregion level

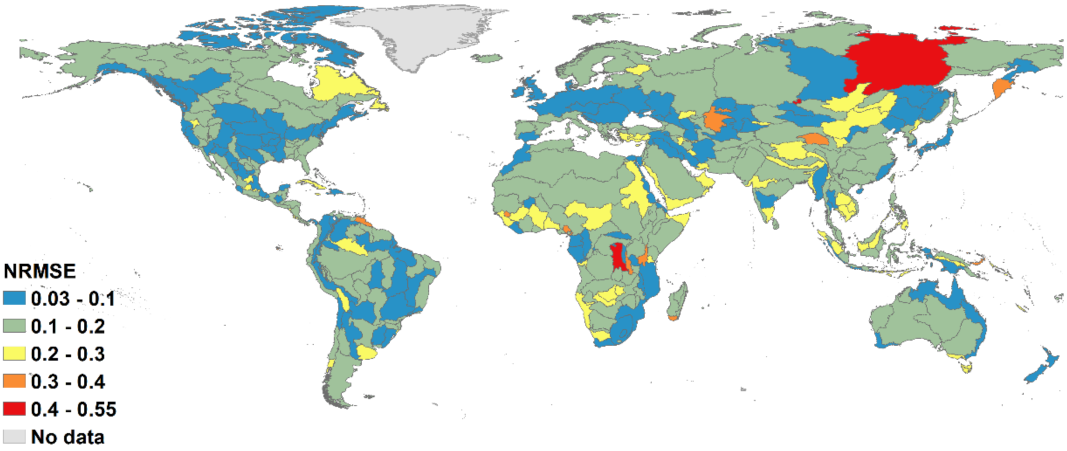

Figure S4 Normalized root mean square error (NRMSE) of species sensitivity distributions (SSDs) at the ecoregion level

S5. Changes in PDF and N concentration

In some grid cells, PDF decreases from the reference year 1900 to the current year 2010 (Figure S5) along with a decrease of N concentration (Figure S6). This results from changes in hydrological conditions (e.g., change in discharge and/or runoff, land use change (e.g., conversion from agricultural land to natural land), and the inconsistency of the dataset whether there is ice cover or not (e.g., Iceland). In such cases, we set the average EF for these regions to "no value".

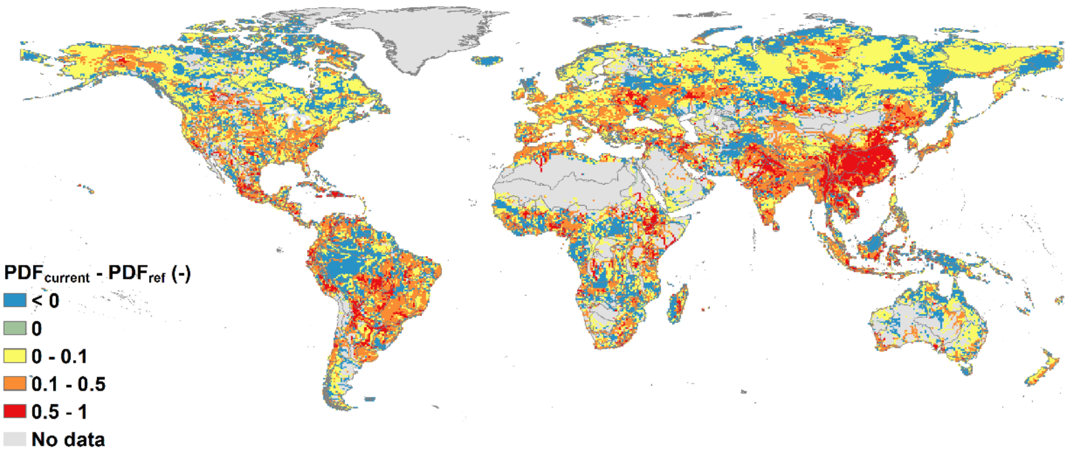

Figure S5 Change in the potentially disappeared fraction of species (PDF) between the current and reference states

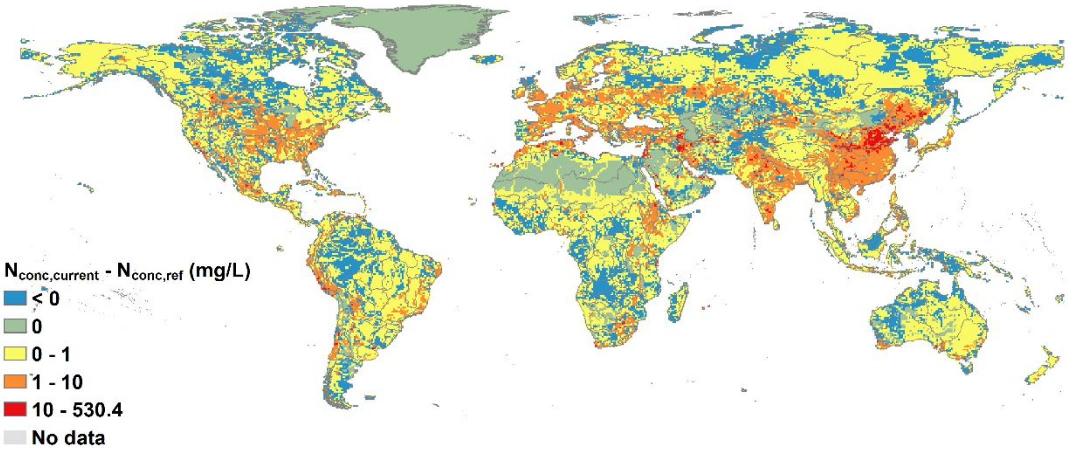

Figure S6 Change in N concentrations between the current and reference states

References:

(1) Larentis, C.; Kotz Kliemann, B. C.; Neves, M. P.; Delariva, R. L. Effects of Human Disturbance on Habitat and Fish Diversity in Neotropical Streams. *PLoS One* **2022**, *17* (9), e0274191–e0274191. https://doi.org/10.1371/journal.pone.0274191.
