# Supplementary figures and images for: Effects of Nitrogen Emissions on Fish Species Richness across the World’s Freshwater Ecoregions (part 2 of 3)
Source: Environ Sci Technol. 2023 May 22;57(22):8347–54. doi: 10.1021/acs.est.2c09333 (PMC10249400; doi:10.1021/acs.est.2c09333)

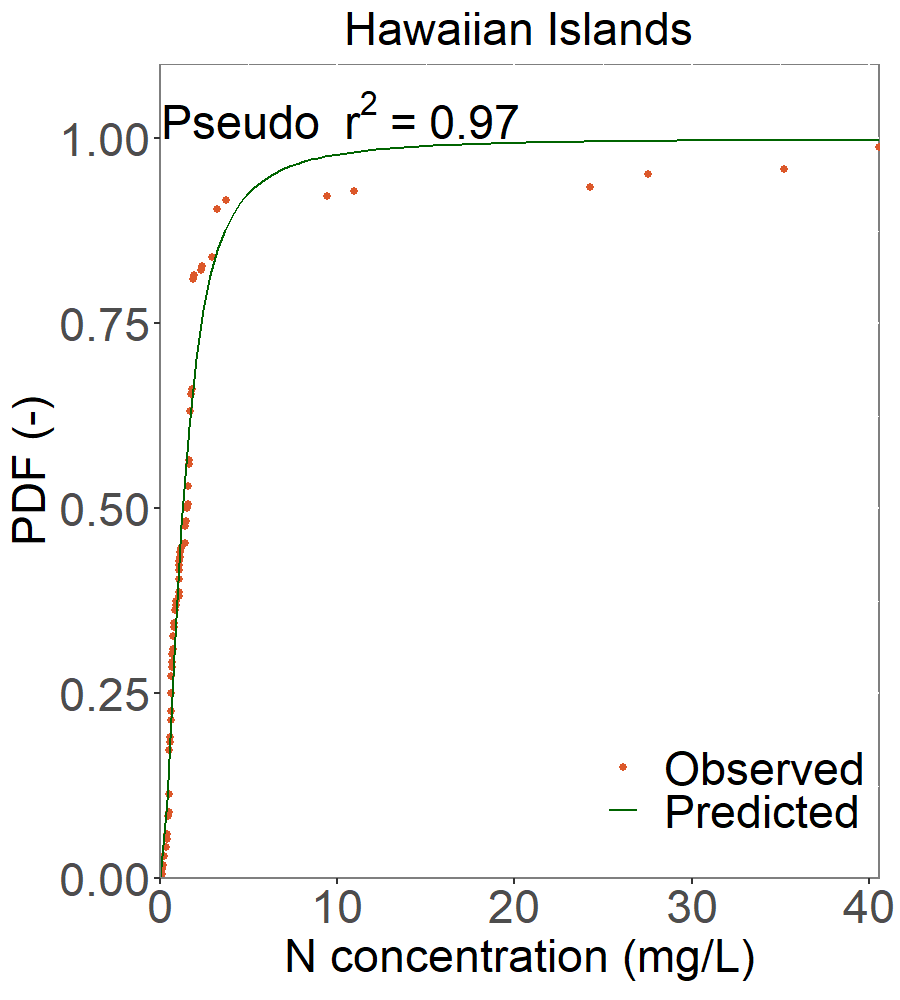

Supplement: Supplementary file 2 — es2c09333_si_002.zip [file es2c09333_si_002.zip › SSD_Ecoregion/Hawaiian Islands.tif]

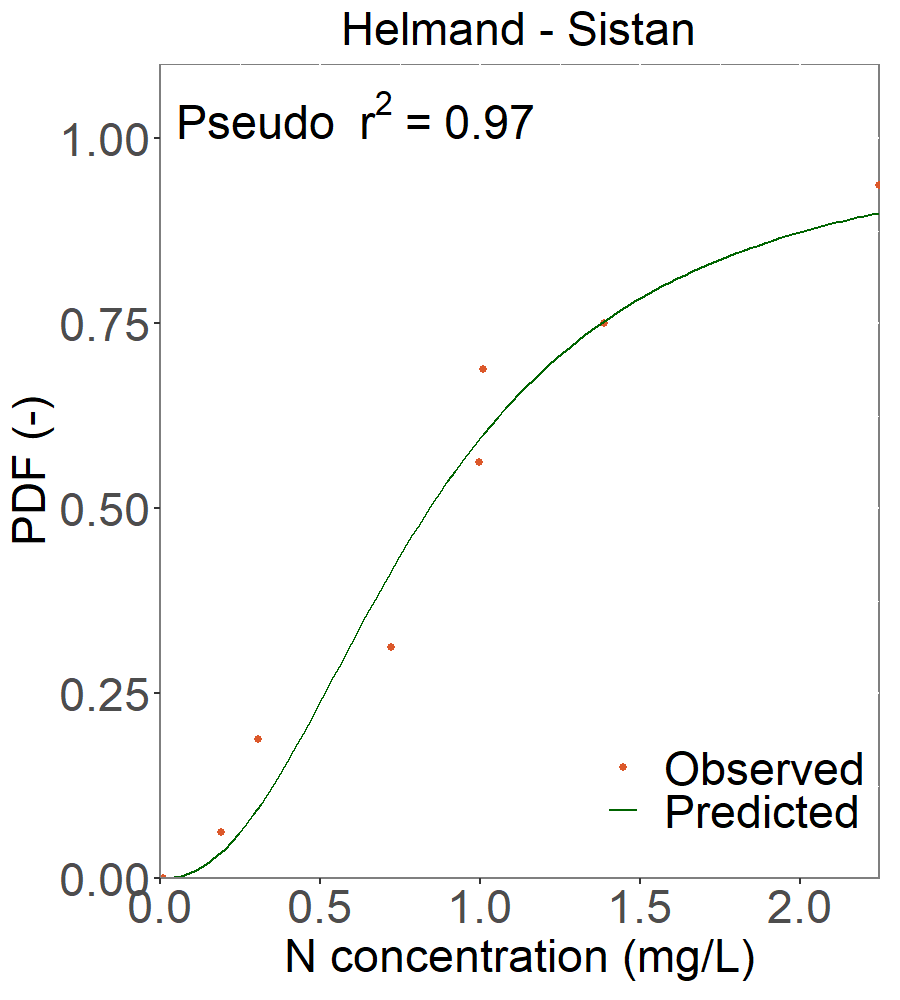

Supplement: Supplementary file 2 — es2c09333_si_002.zip [file es2c09333_si_002.zip › SSD_Ecoregion/Helmand - Sistan.tif]

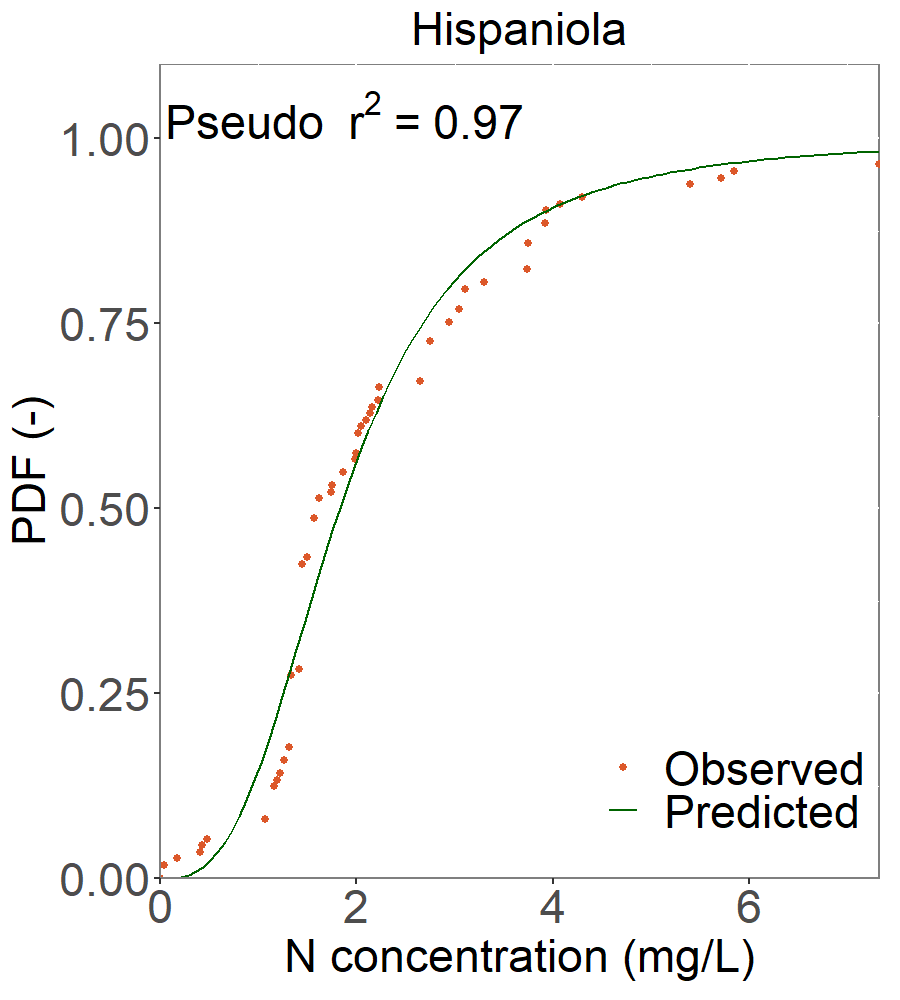

Supplement: Supplementary file 2 — es2c09333_si_002.zip [file es2c09333_si_002.zip › SSD_Ecoregion/Hispaniola.tif]

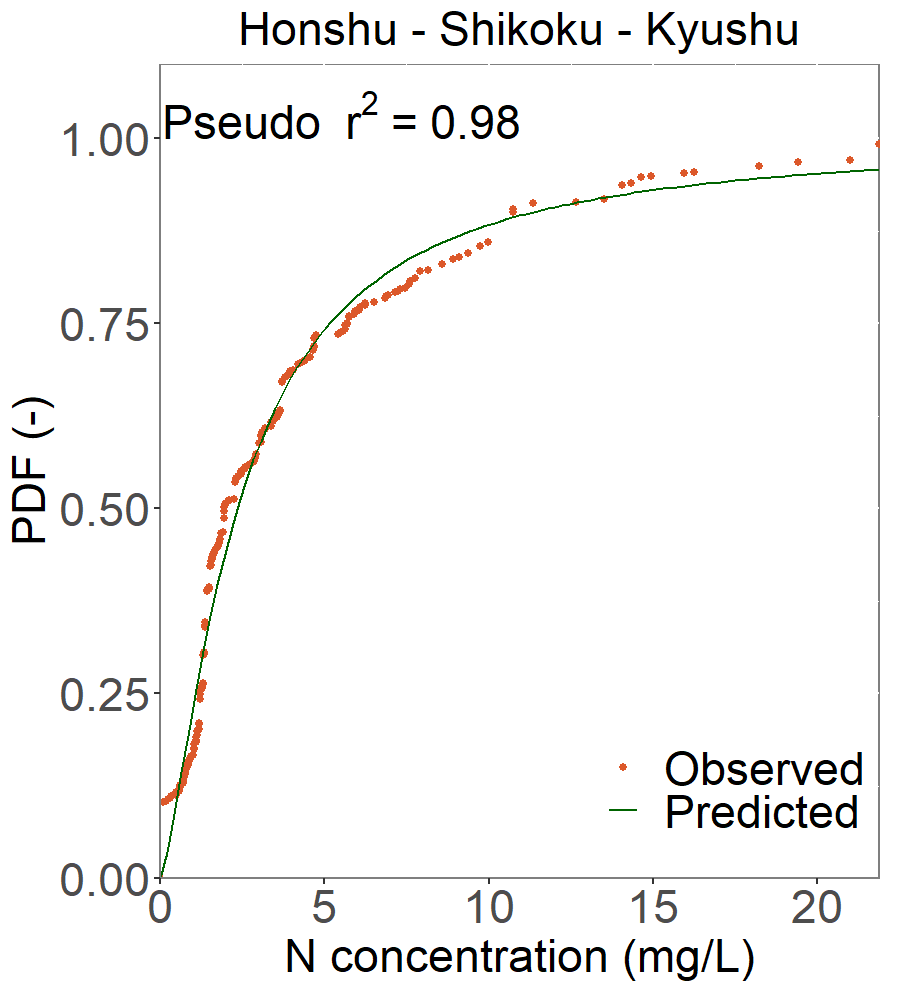

Supplement: Supplementary file 2 — es2c09333_si_002.zip [file es2c09333_si_002.zip › SSD_Ecoregion/Honshu - Shikoku - Kyushu.tif]

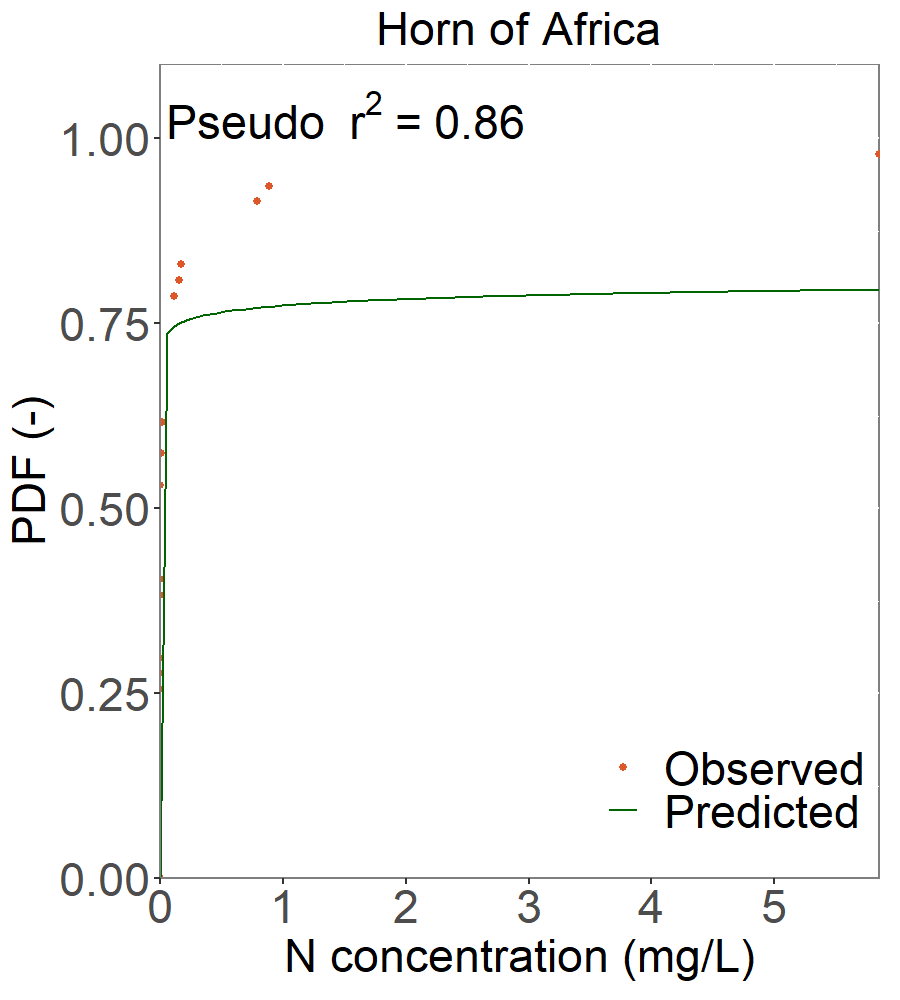

Supplement: Supplementary file 2 — es2c09333_si_002.zip [file es2c09333_si_002.zip › SSD_Ecoregion/Horn of Africa.tif]

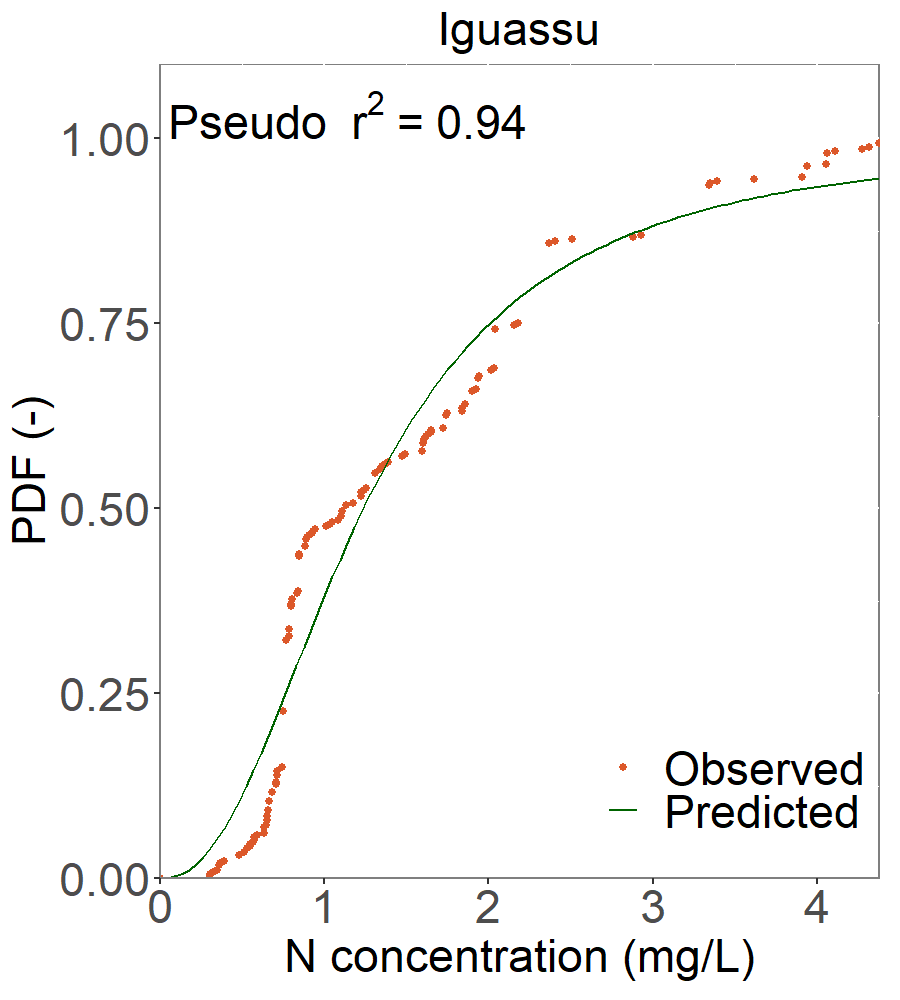

Supplement: Supplementary file 2 — es2c09333_si_002.zip [file es2c09333_si_002.zip › SSD_Ecoregion/Iguassu.tif]

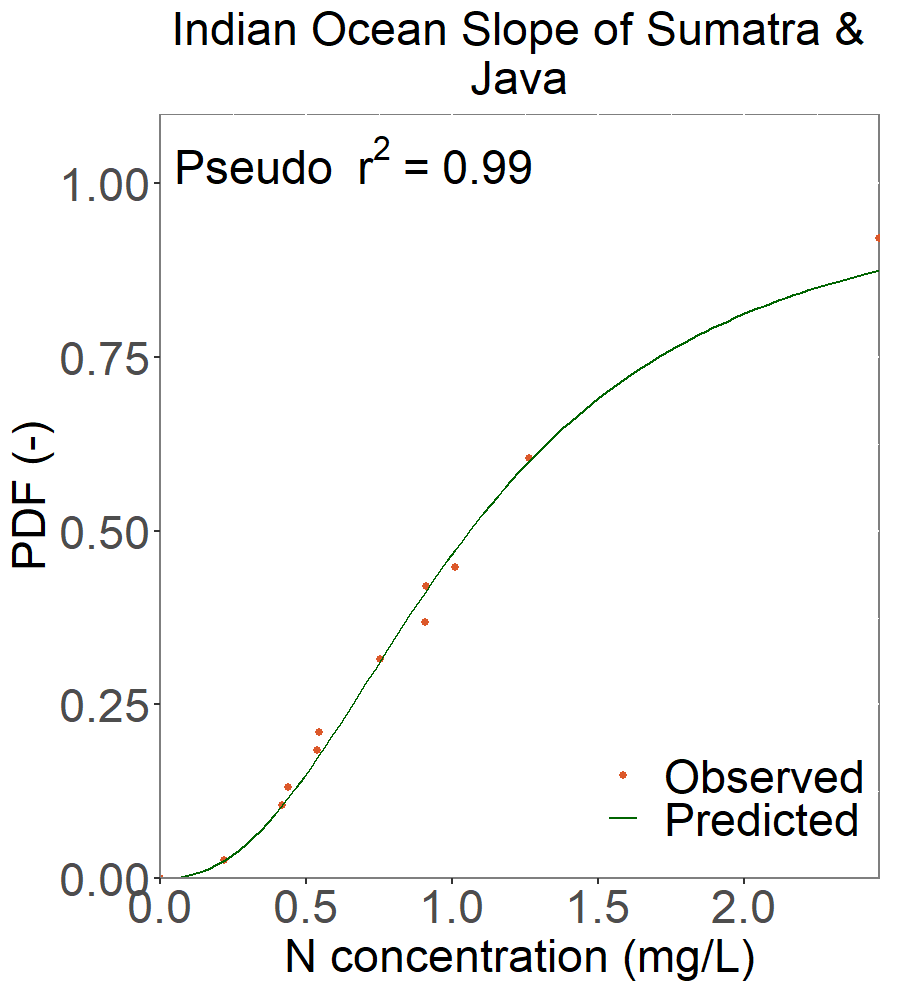

Supplement: Supplementary file 2 — es2c09333_si_002.zip [file es2c09333_si_002.zip › SSD_Ecoregion/Indian Ocean Slope of Sumatra & Java.tif]

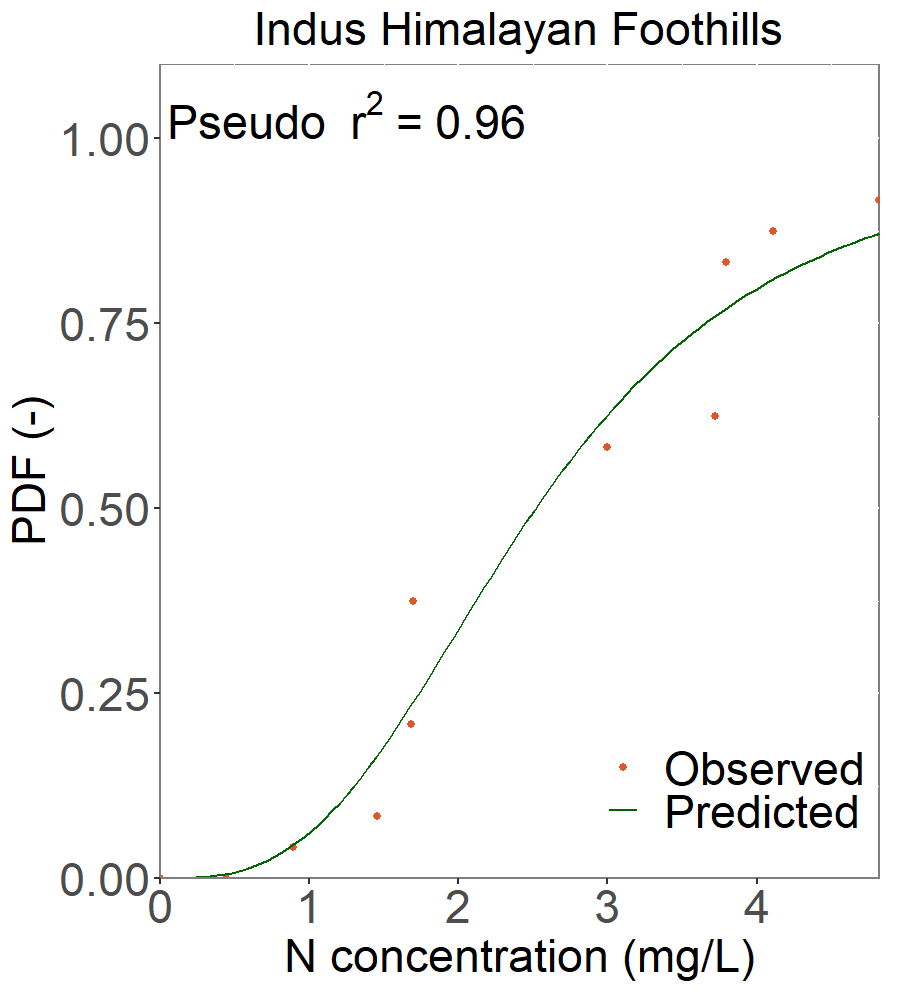

Supplement: Supplementary file 2 — es2c09333_si_002.zip [file es2c09333_si_002.zip › SSD_Ecoregion/Indus Himalayan Foothills.tif]

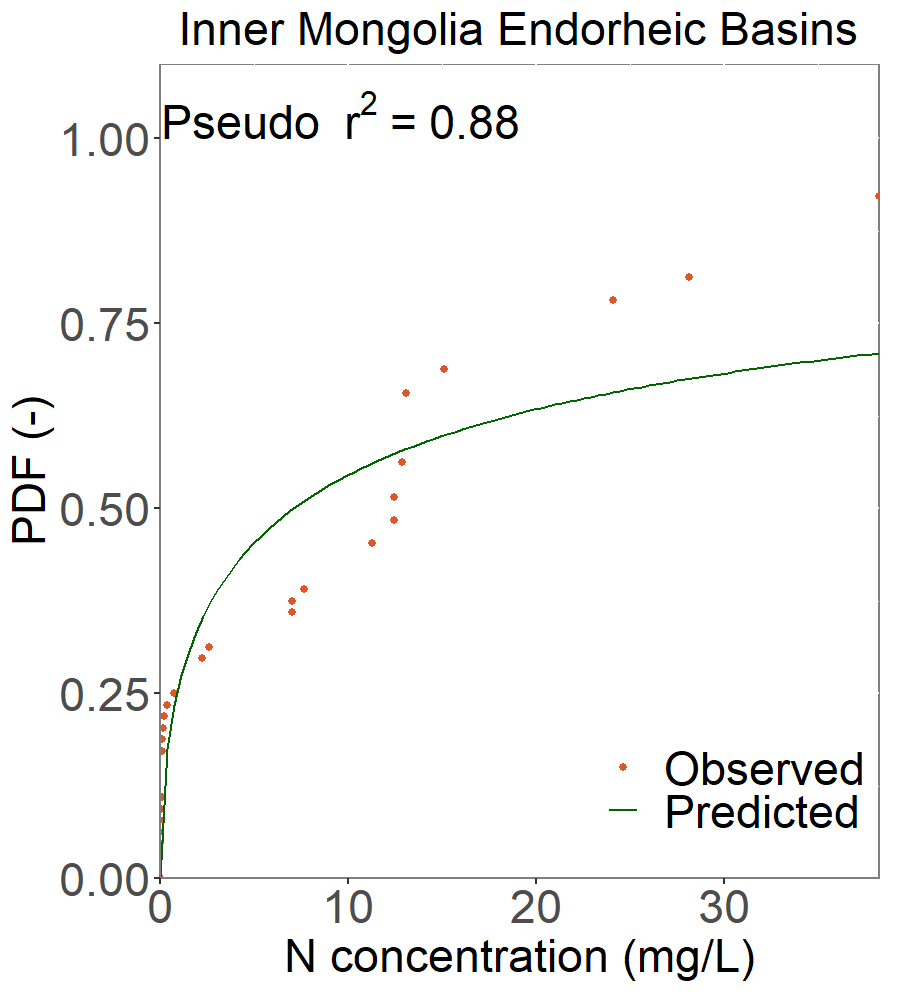

Supplement: Supplementary file 2 — es2c09333_si_002.zip [file es2c09333_si_002.zip › SSD_Ecoregion/Inner Mongolia Endorheic Basins.tif]

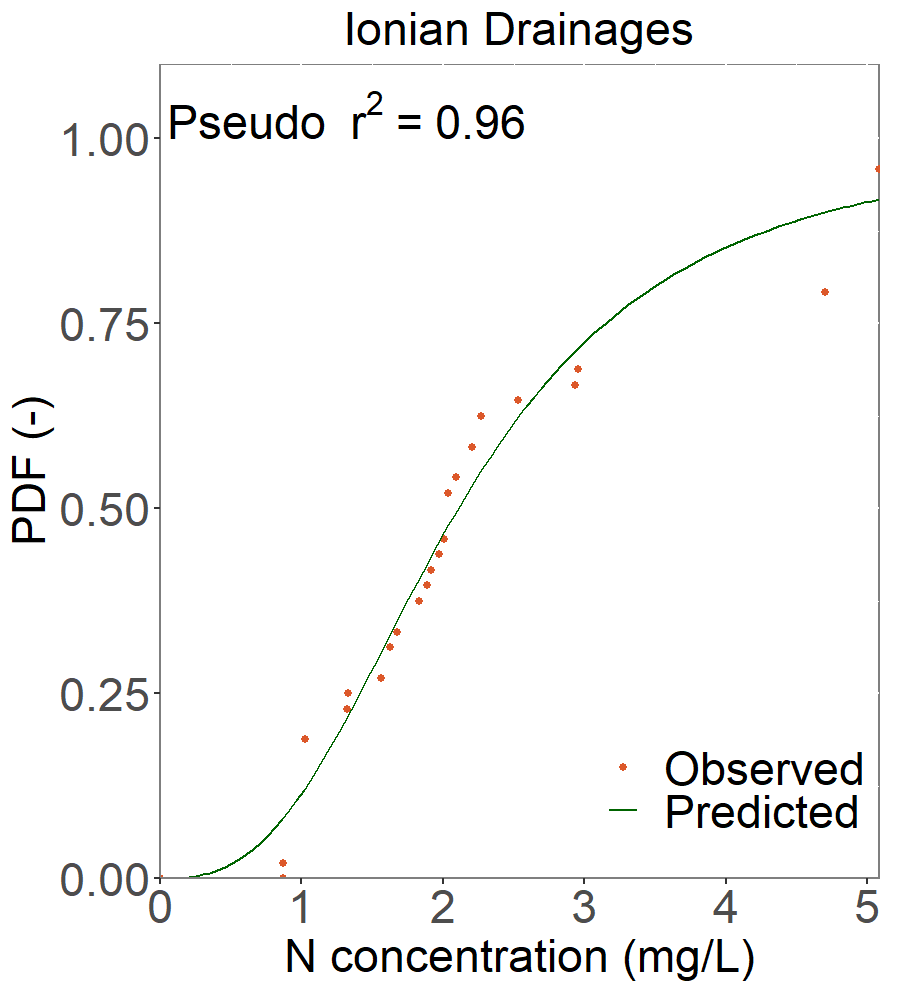

Supplement: Supplementary file 2 — es2c09333_si_002.zip [file es2c09333_si_002.zip › SSD_Ecoregion/Ionian Drainages.tif]

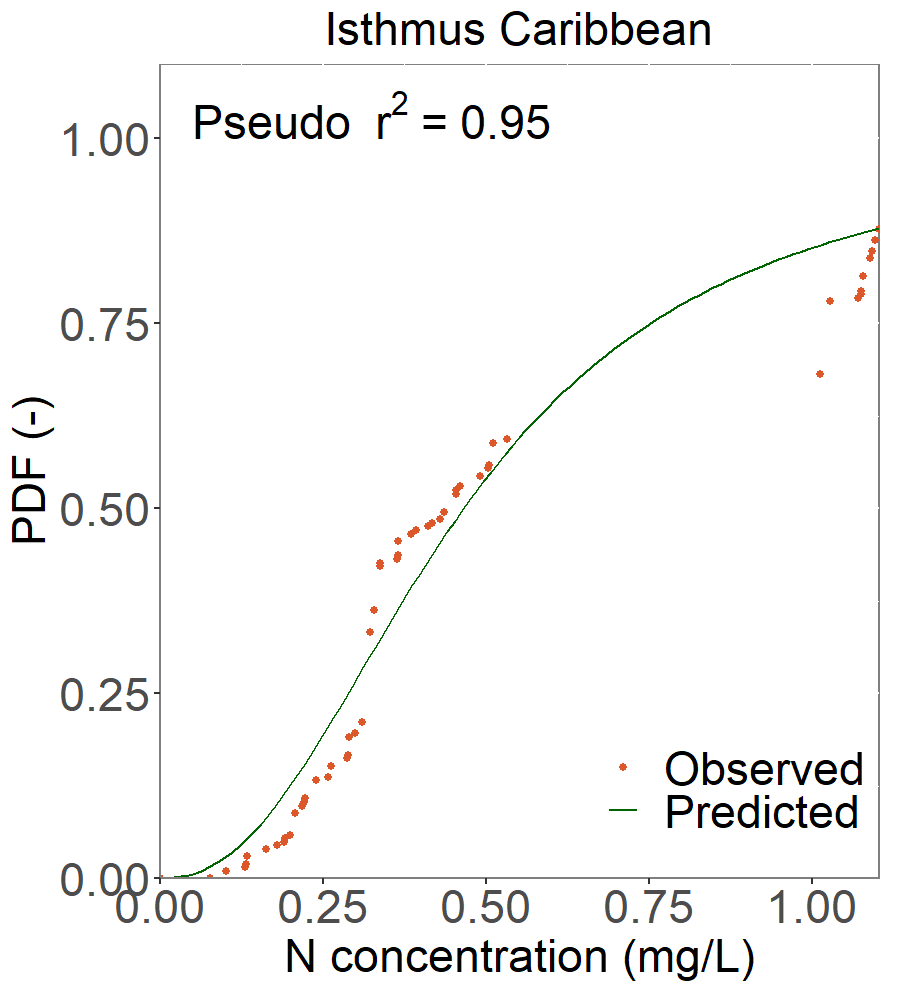

Supplement: Supplementary file 2 — es2c09333_si_002.zip [file es2c09333_si_002.zip › SSD_Ecoregion/Isthmus Caribbean.tif]

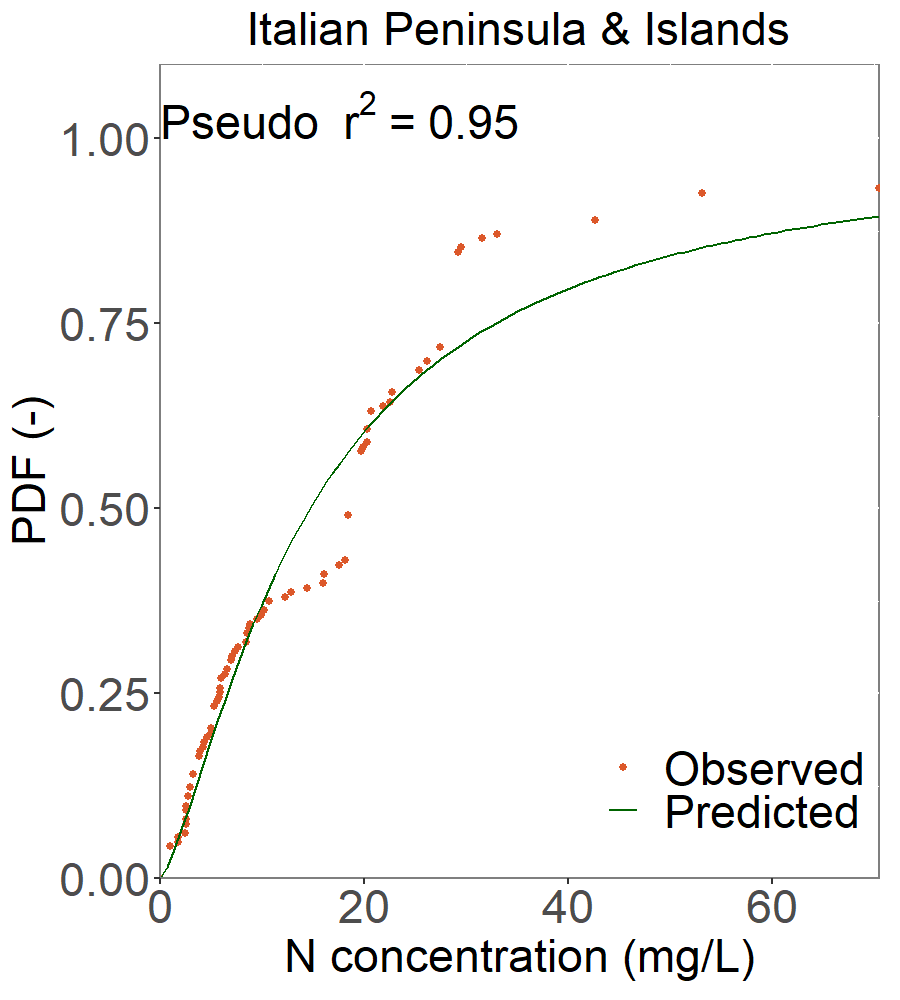

Supplement: Supplementary file 2 — es2c09333_si_002.zip [file es2c09333_si_002.zip › SSD_Ecoregion/Italian Peninsula & Islands.tif]

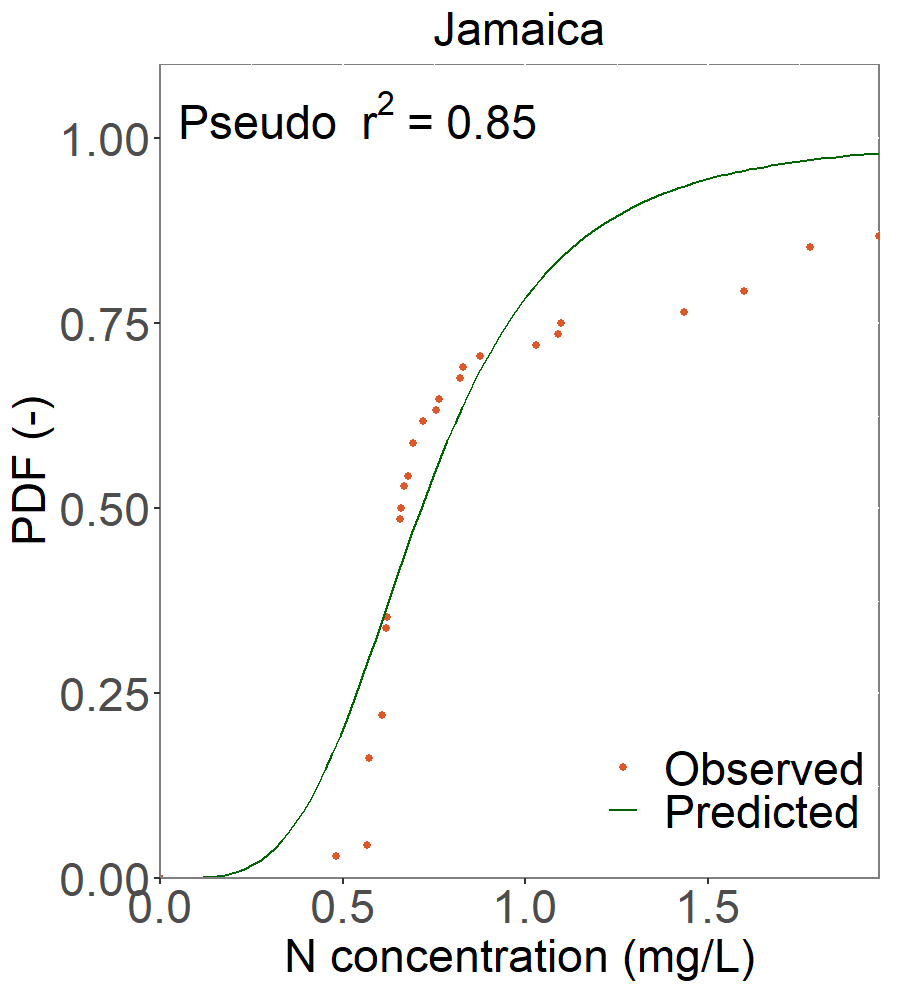

Supplement: Supplementary file 2 — es2c09333_si_002.zip [file es2c09333_si_002.zip › SSD_Ecoregion/Jamaica.tif]

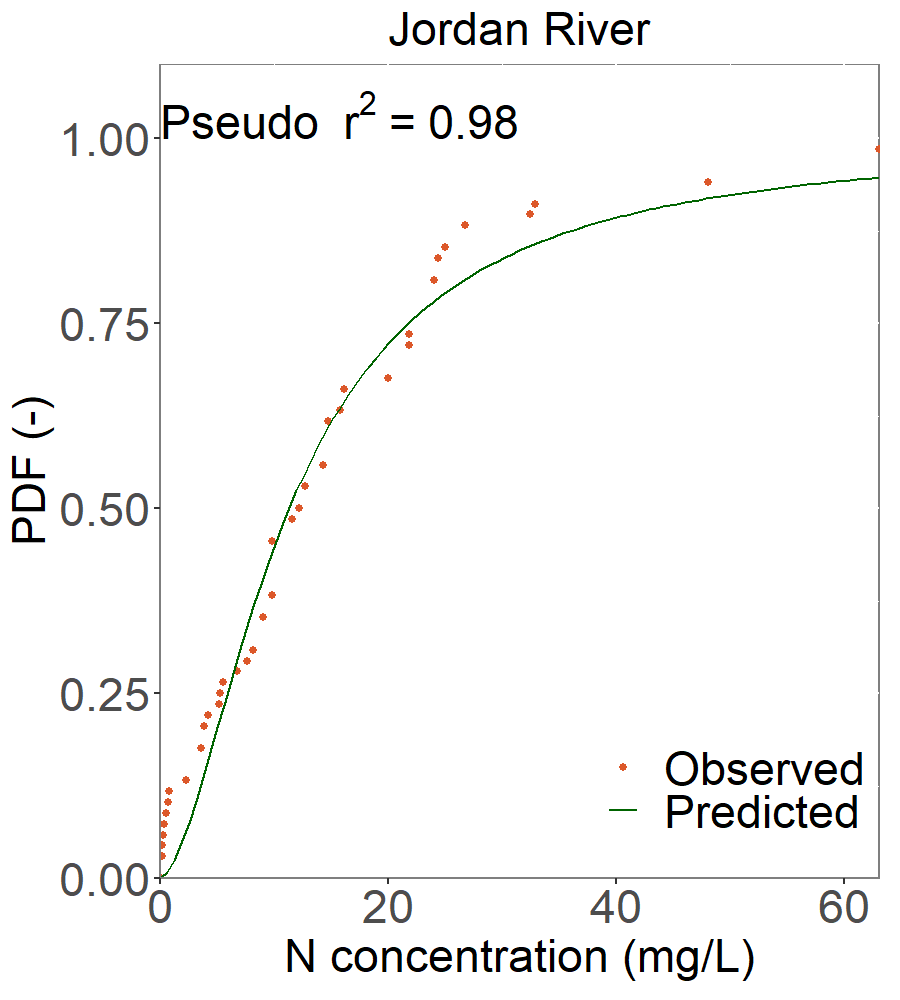

Supplement: Supplementary file 2 — es2c09333_si_002.zip [file es2c09333_si_002.zip › SSD_Ecoregion/Jordan River.tif]

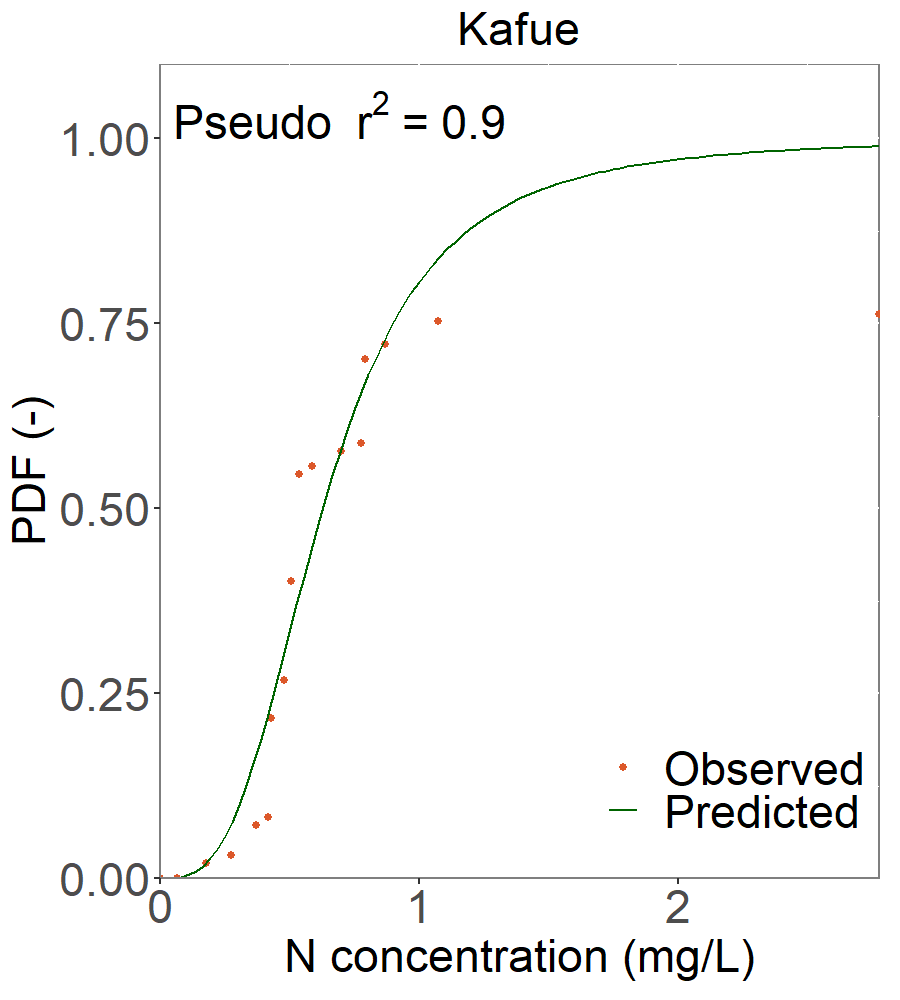

Supplement: Supplementary file 2 — es2c09333_si_002.zip [file es2c09333_si_002.zip › SSD_Ecoregion/Kafue.tif]

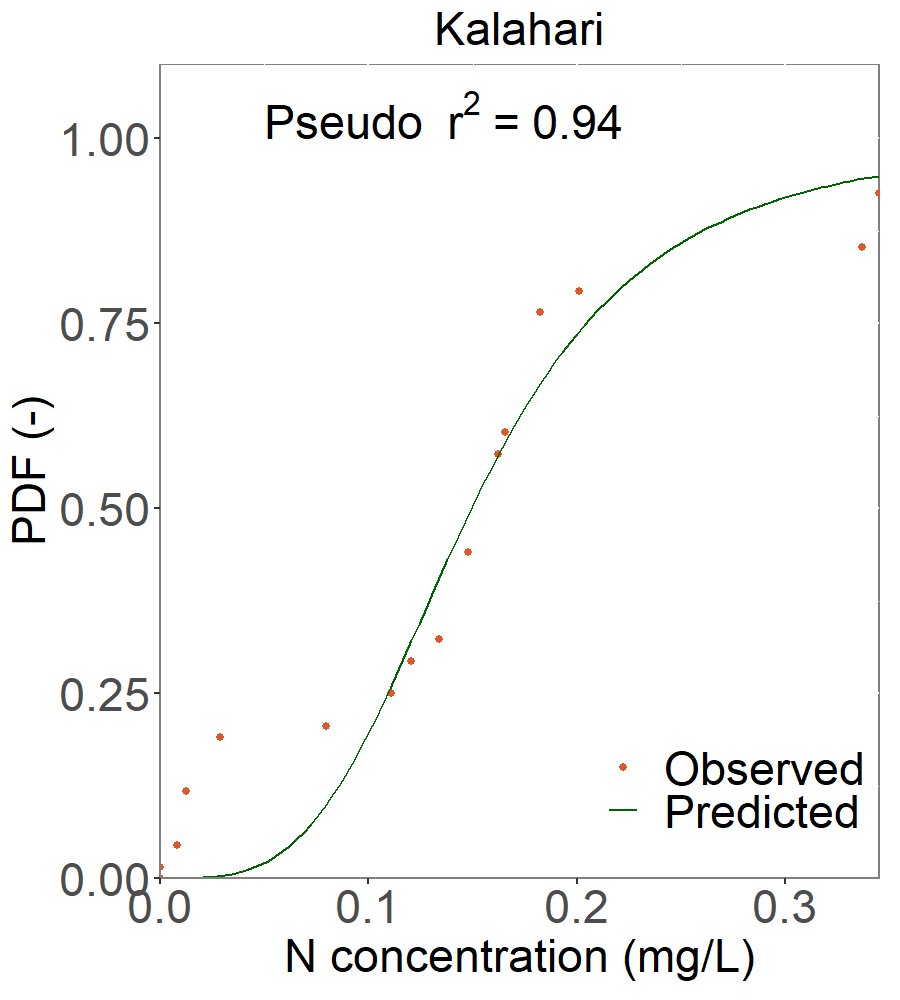

Supplement: Supplementary file 2 — es2c09333_si_002.zip [file es2c09333_si_002.zip › SSD_Ecoregion/Kalahari.tif]

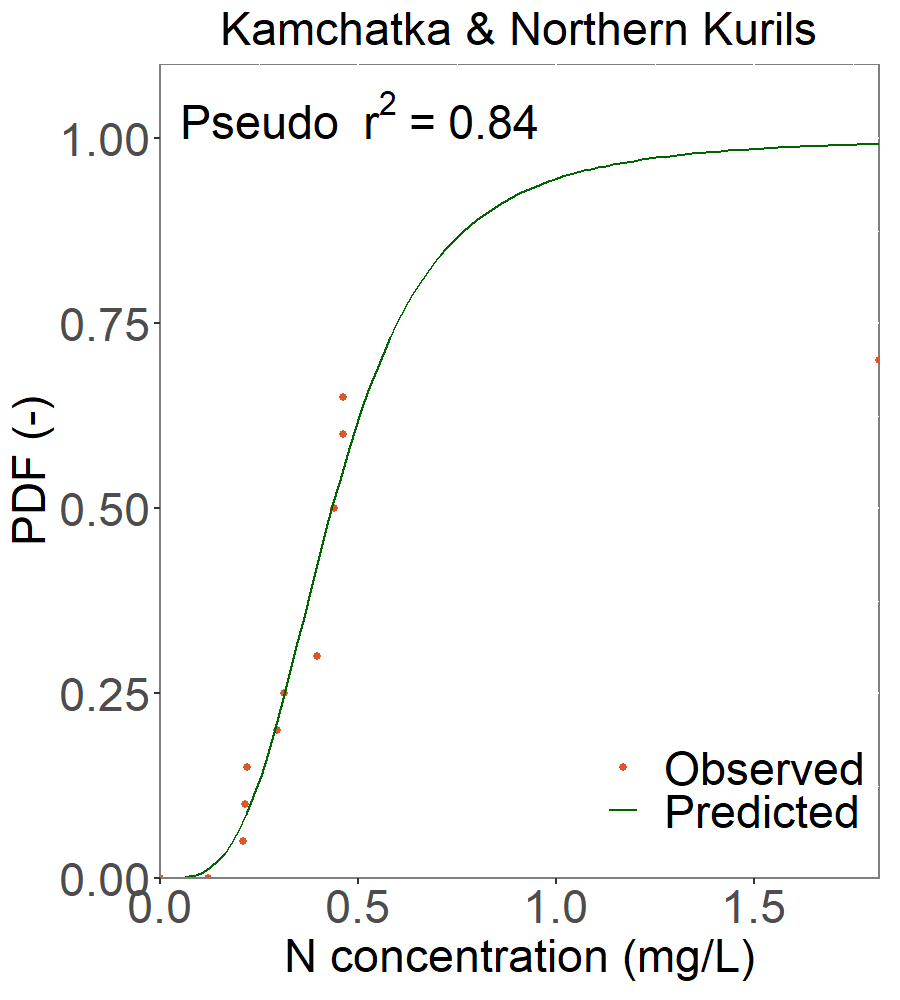

Supplement: Supplementary file 2 — es2c09333_si_002.zip [file es2c09333_si_002.zip › SSD_Ecoregion/Kamchatka & Northern Kurils.tif]

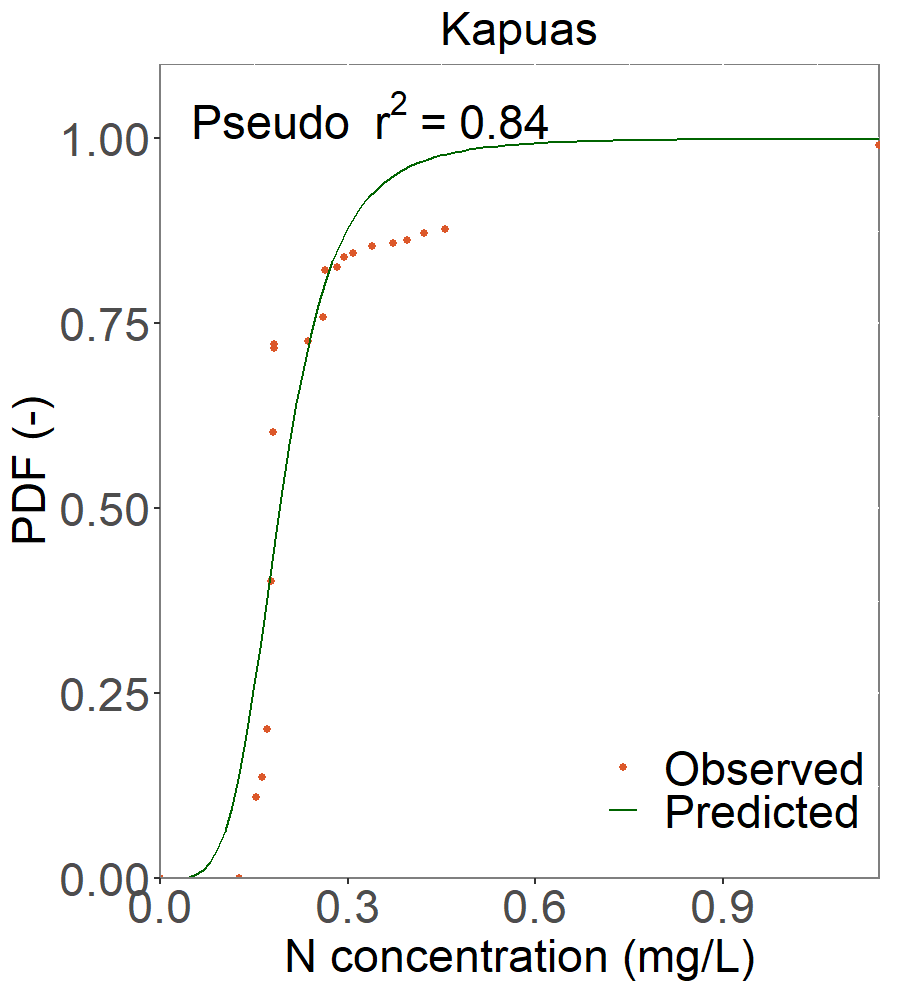

Supplement: Supplementary file 2 — es2c09333_si_002.zip [file es2c09333_si_002.zip › SSD_Ecoregion/Kapuas.tif]

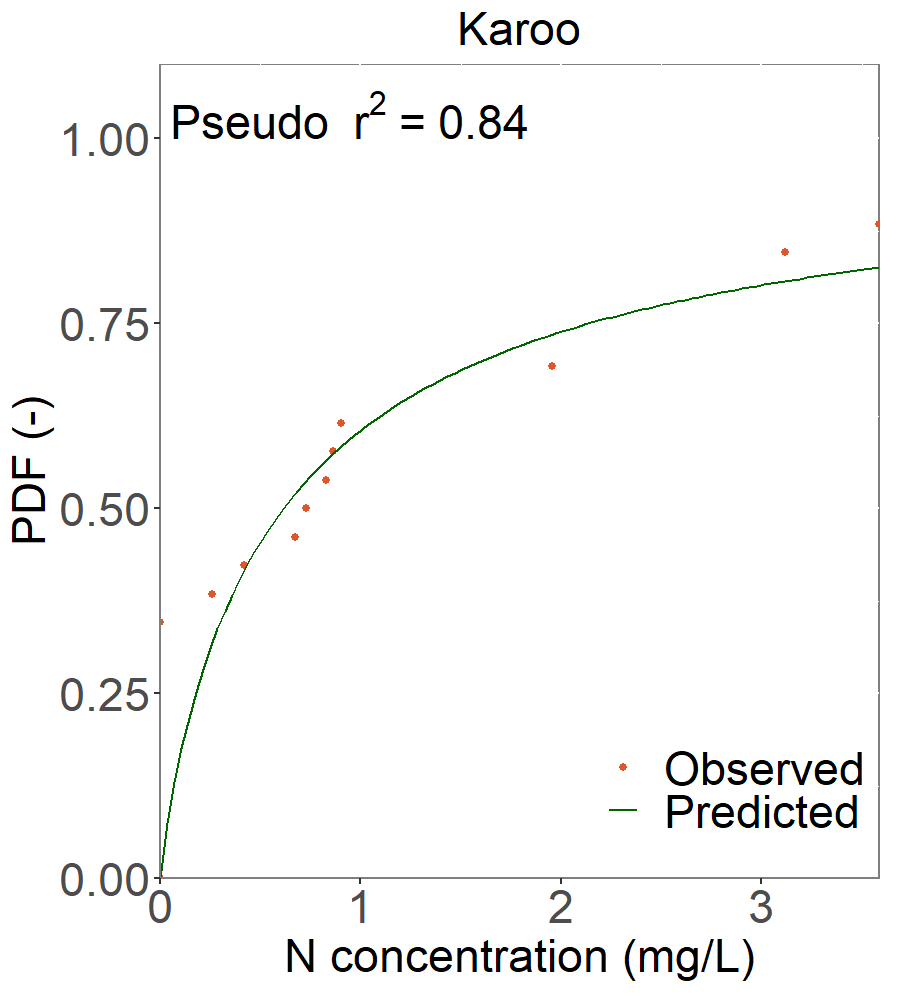

Supplement: Supplementary file 2 — es2c09333_si_002.zip [file es2c09333_si_002.zip › SSD_Ecoregion/Karoo.tif]

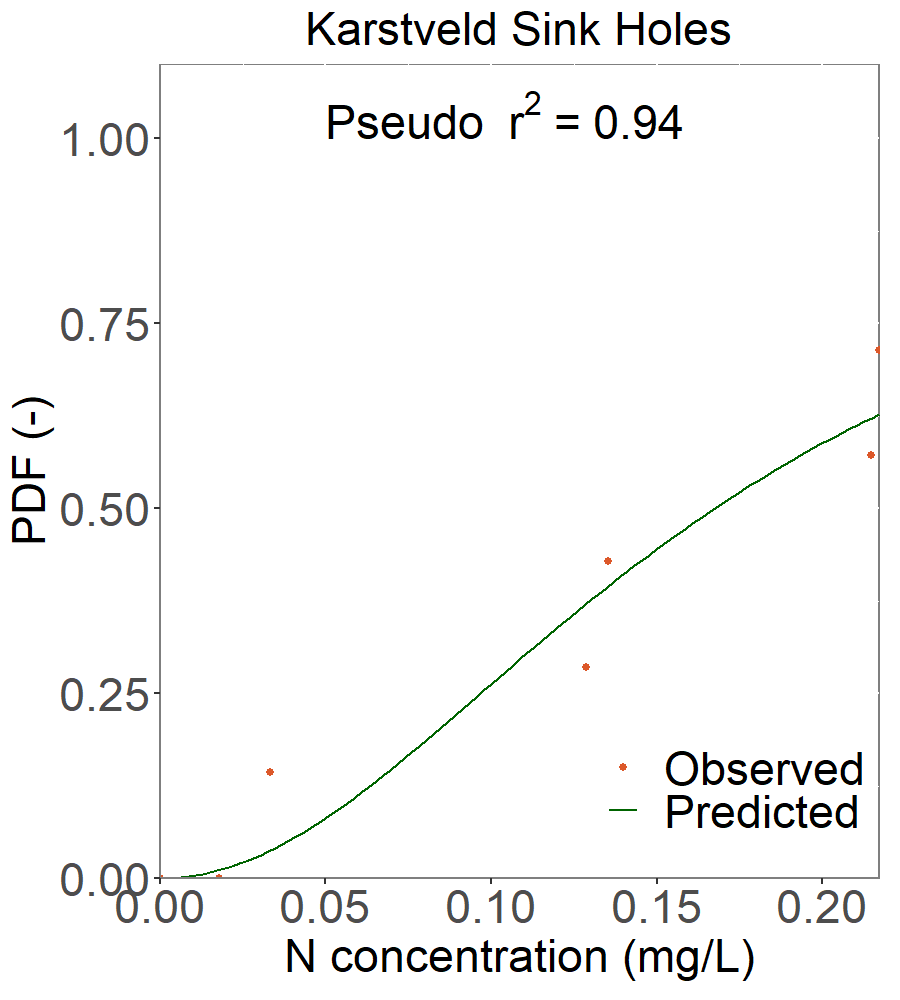

Supplement: Supplementary file 2 — es2c09333_si_002.zip [file es2c09333_si_002.zip › SSD_Ecoregion/Karstveld Sink Holes.tif]

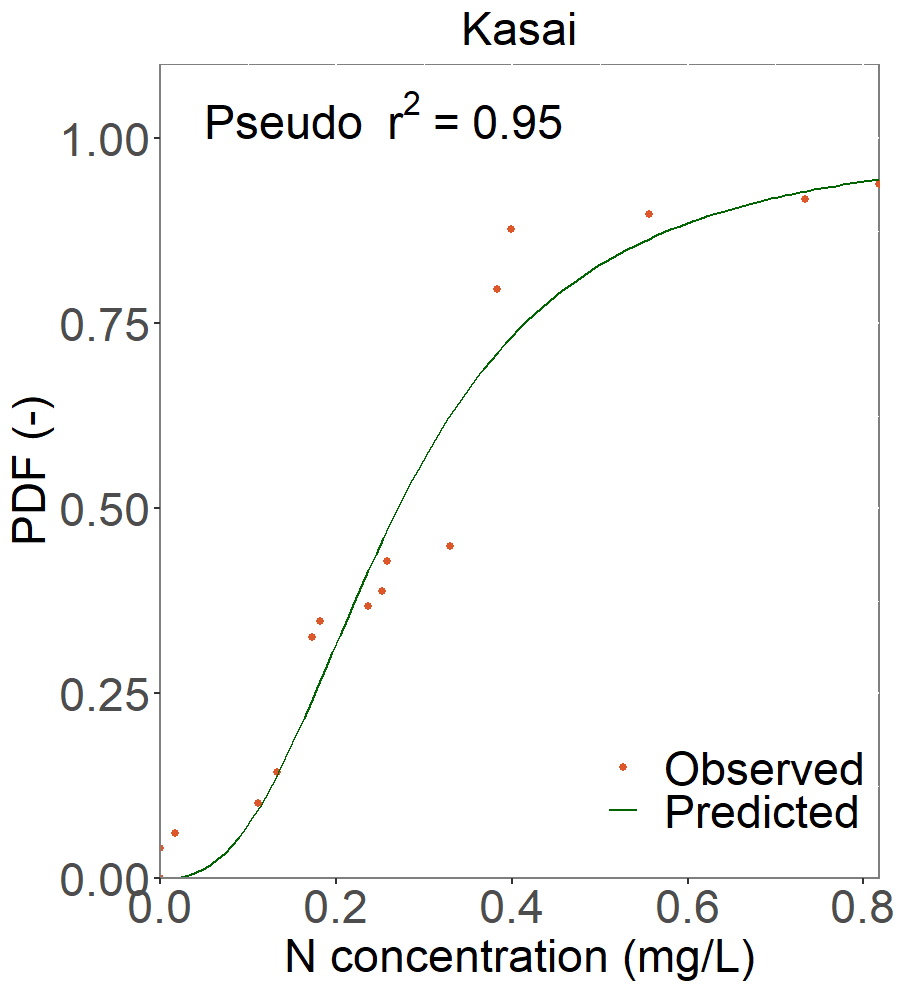

Supplement: Supplementary file 2 — es2c09333_si_002.zip [file es2c09333_si_002.zip › SSD_Ecoregion/Kasai.tif]

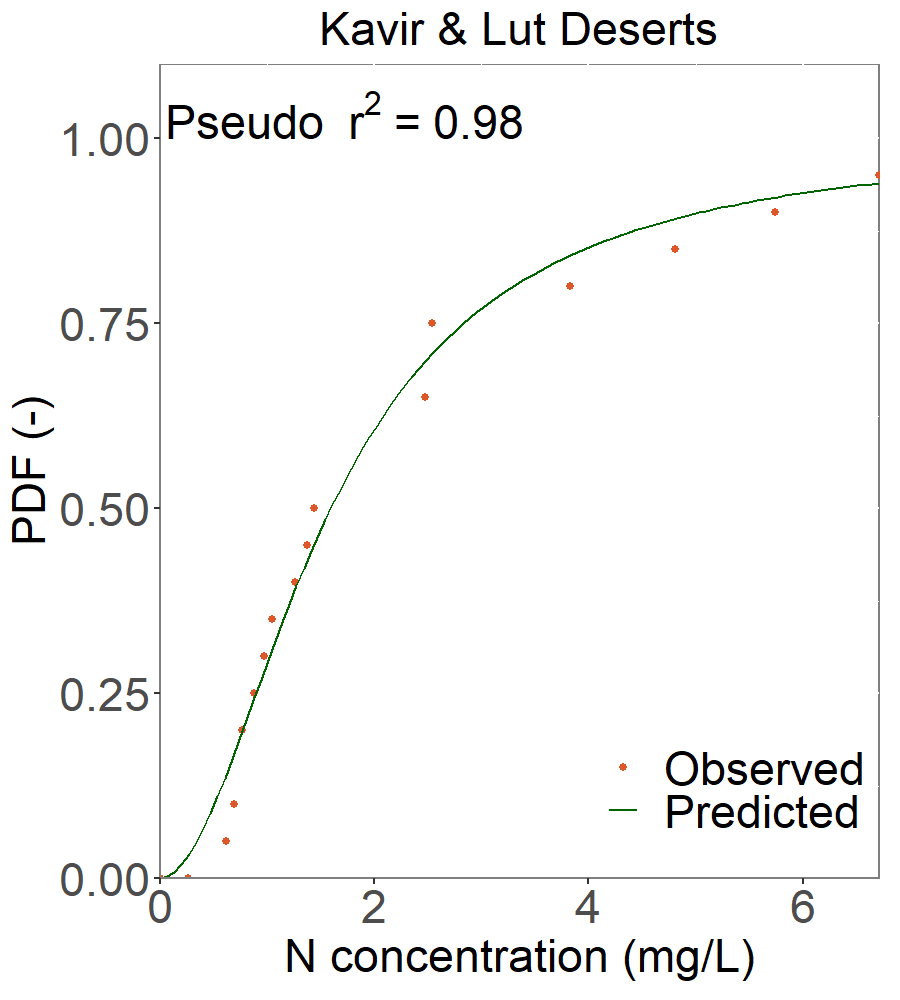

Supplement: Supplementary file 2 — es2c09333_si_002.zip [file es2c09333_si_002.zip › SSD_Ecoregion/Kavir & Lut Deserts.tif]

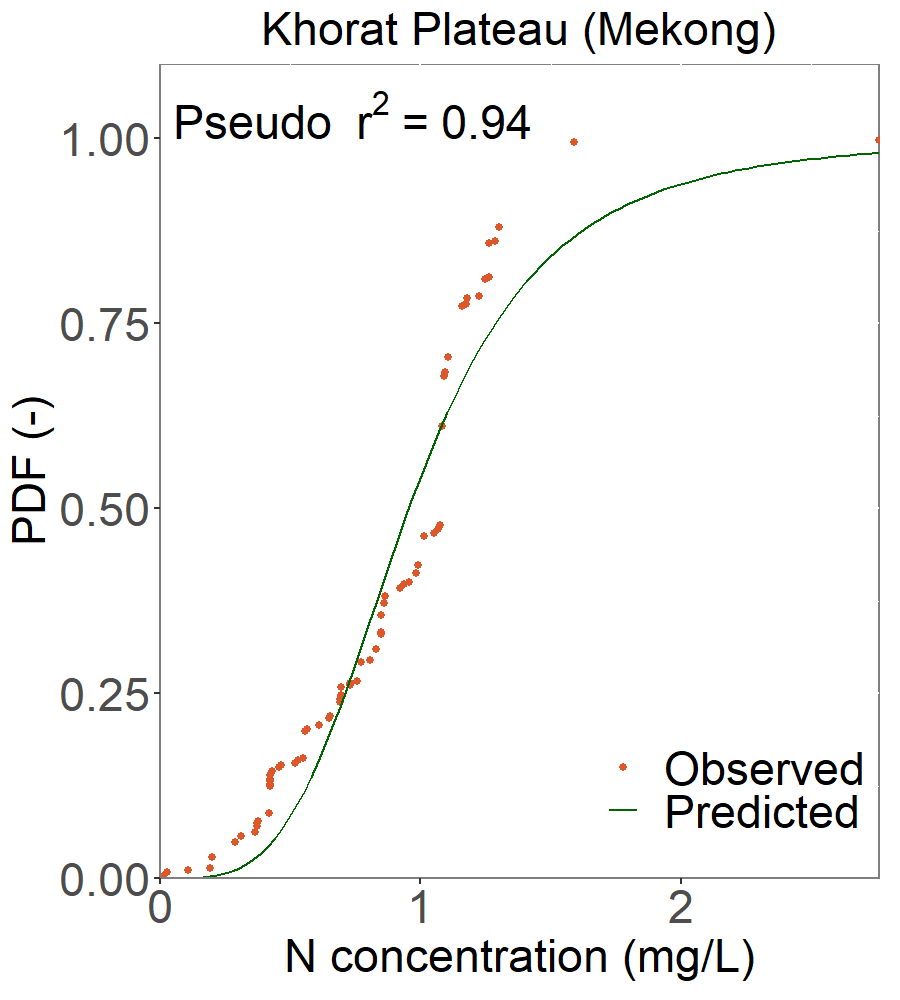

Supplement: Supplementary file 2 — es2c09333_si_002.zip [file es2c09333_si_002.zip › SSD_Ecoregion/Khorat Plateau (Mekong).tif]

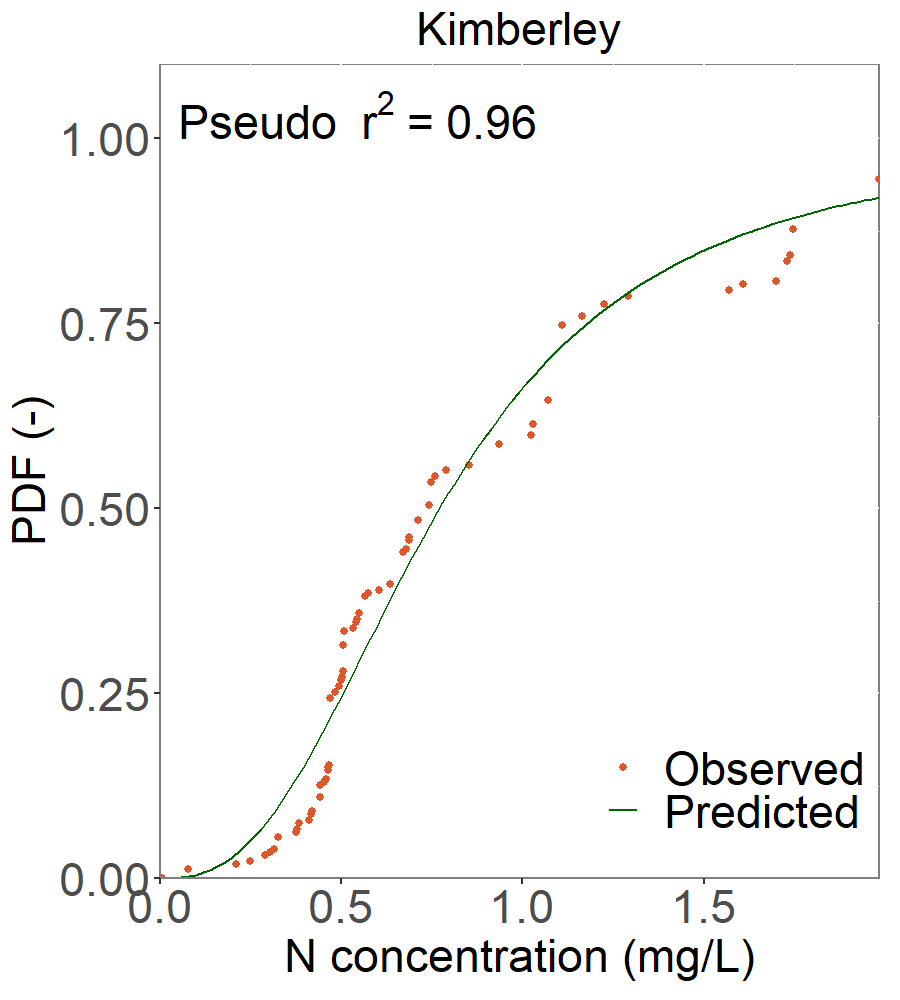

Supplement: Supplementary file 2 — es2c09333_si_002.zip [file es2c09333_si_002.zip › SSD_Ecoregion/Kimberley.tif]

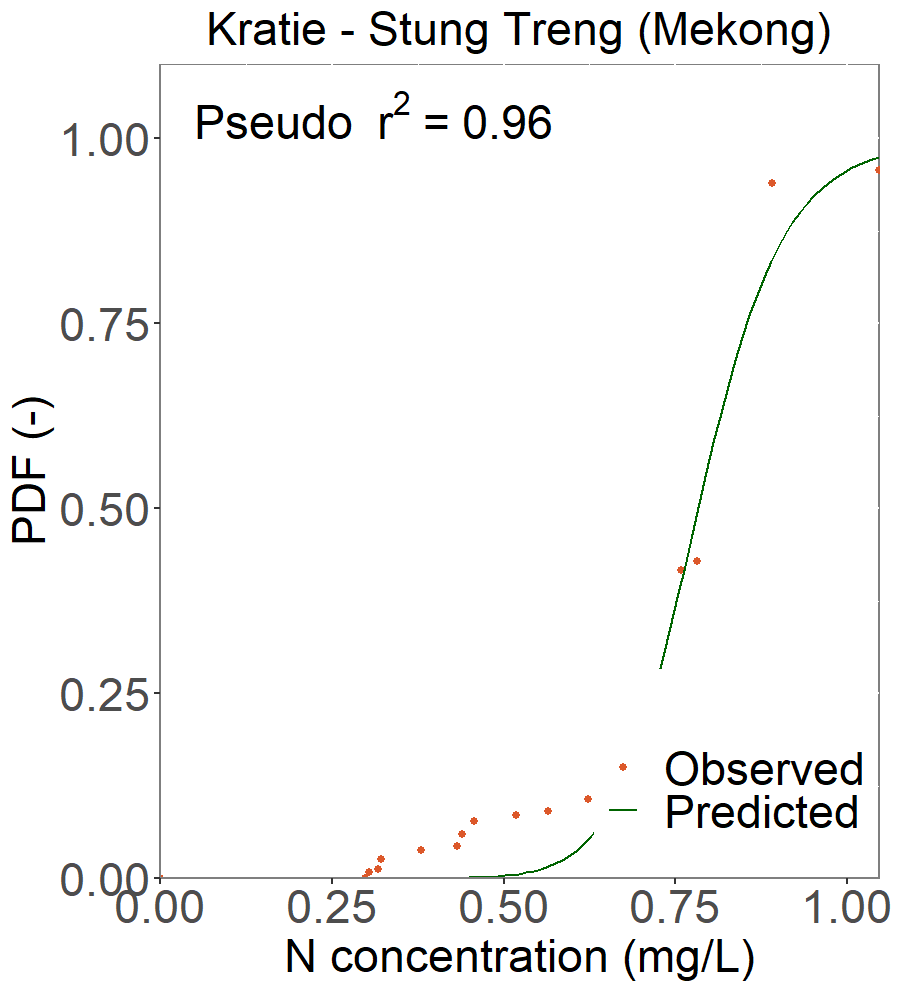

Supplement: Supplementary file 2 — es2c09333_si_002.zip [file es2c09333_si_002.zip › SSD_Ecoregion/Kratie - Stung Treng (Mekong).tif]

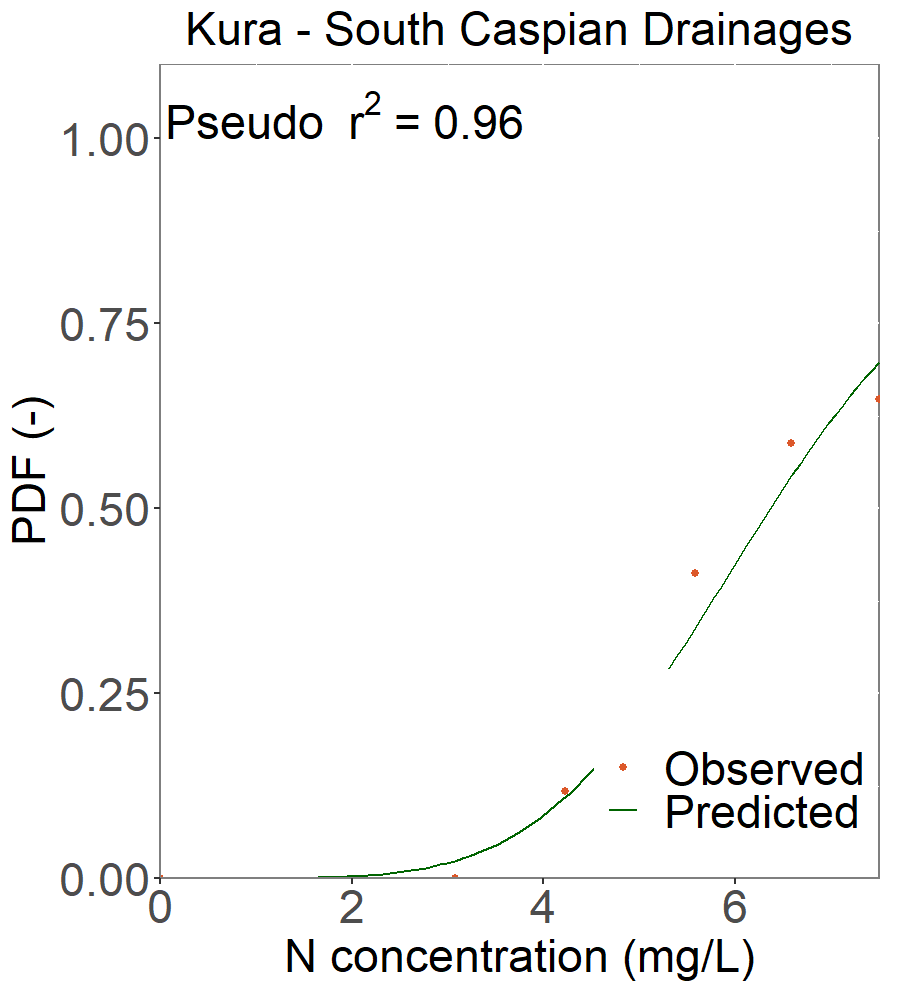

Supplement: Supplementary file 2 — es2c09333_si_002.zip [file es2c09333_si_002.zip › SSD_Ecoregion/Kura - South Caspian Drainages.tif]

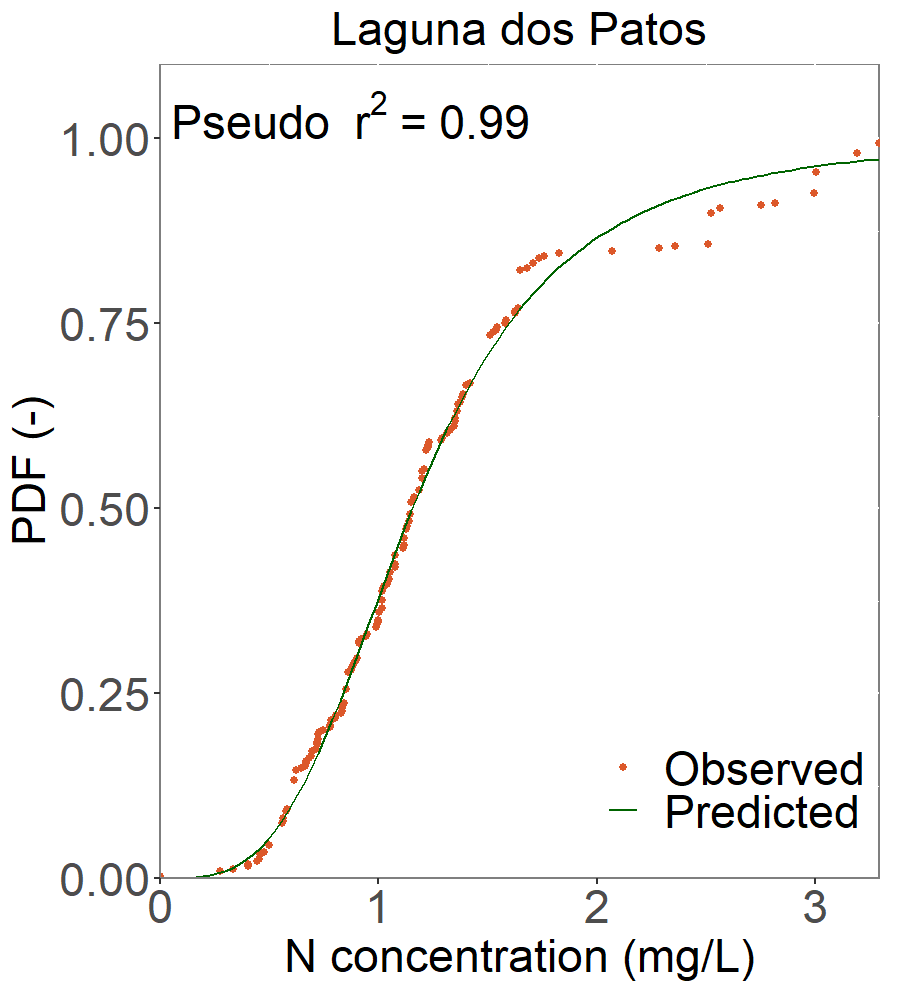

Supplement: Supplementary file 2 — es2c09333_si_002.zip [file es2c09333_si_002.zip › SSD_Ecoregion/Laguna dos Patos.tif]

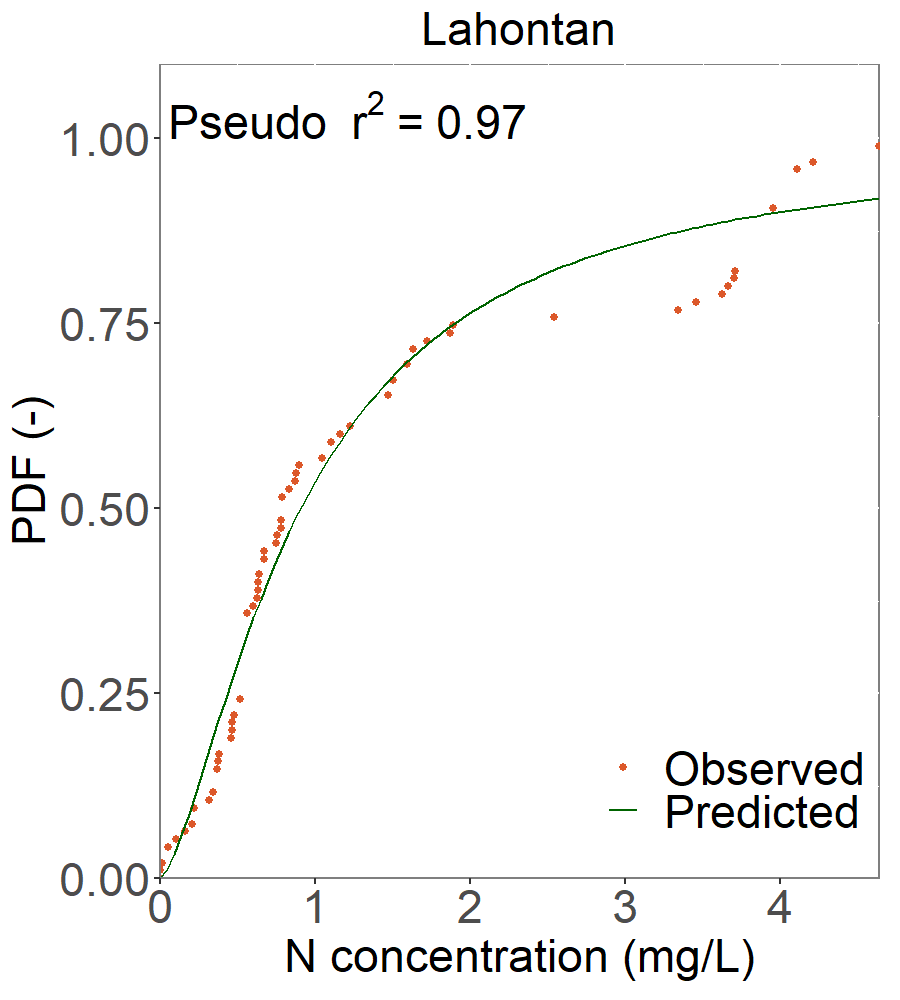

Supplement: Supplementary file 2 — es2c09333_si_002.zip [file es2c09333_si_002.zip › SSD_Ecoregion/Lahontan.tif]

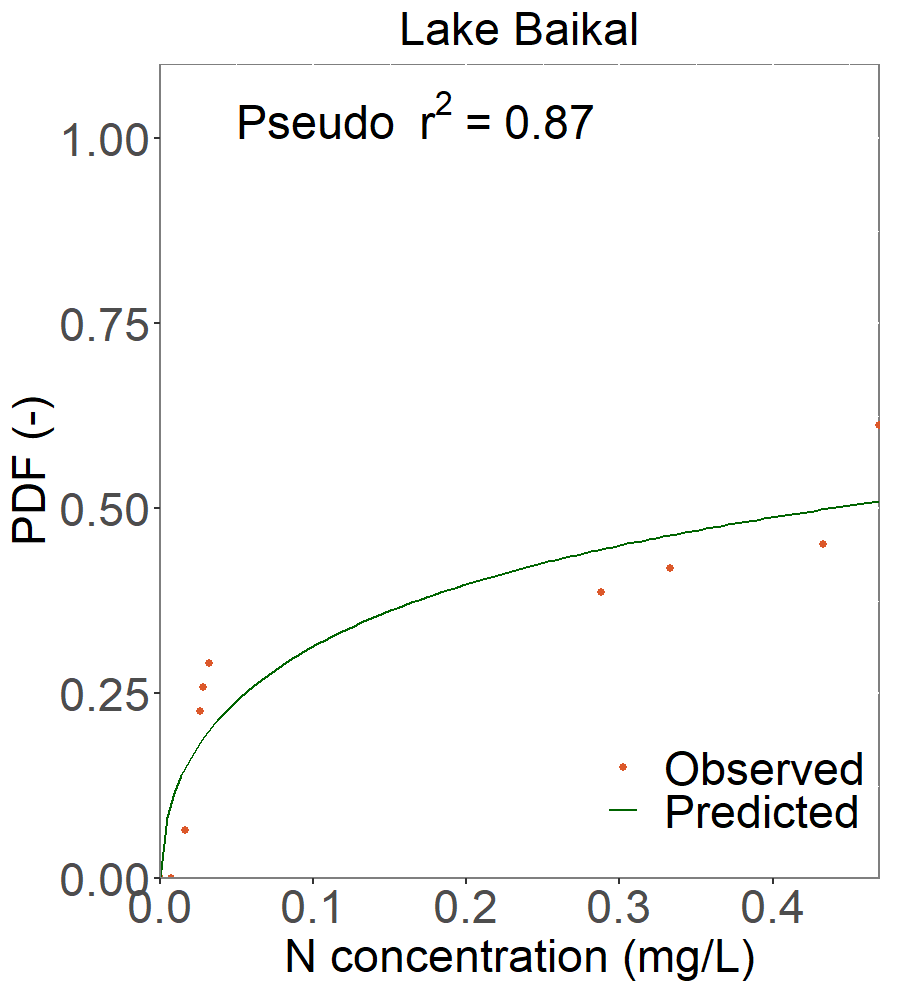

Supplement: Supplementary file 2 — es2c09333_si_002.zip [file es2c09333_si_002.zip › SSD_Ecoregion/Lake Baikal.tif]

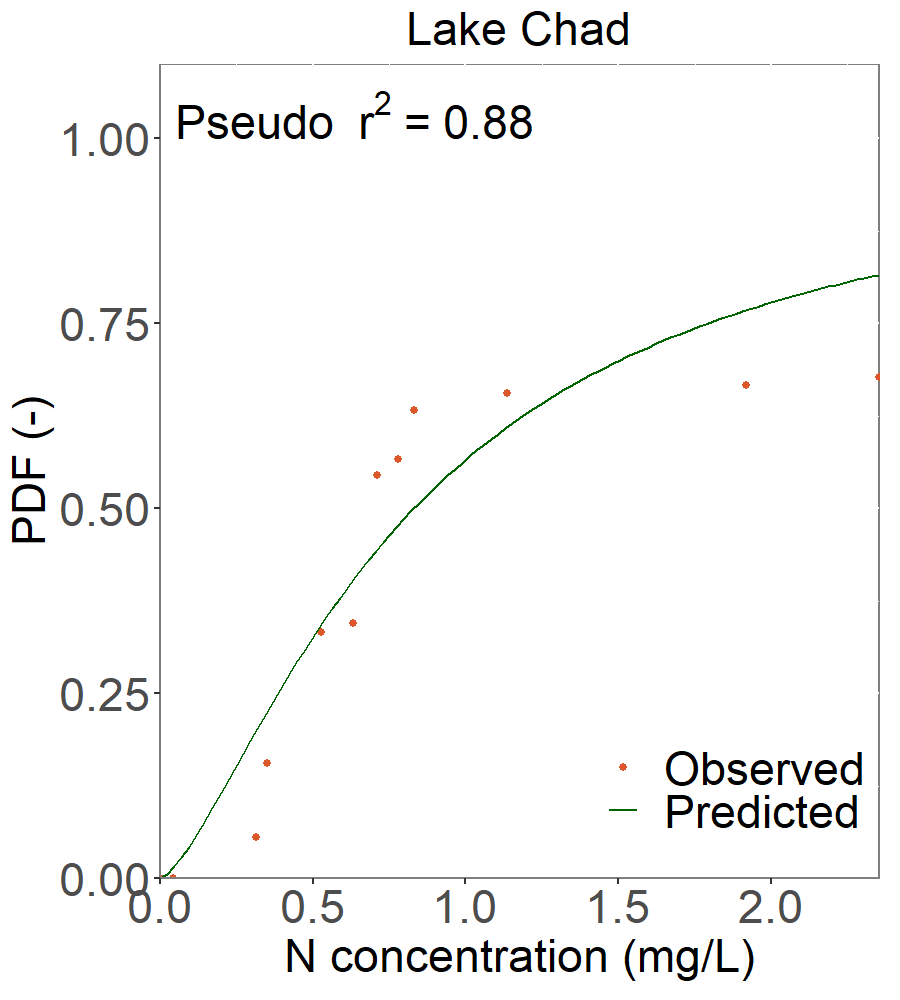

Supplement: Supplementary file 2 — es2c09333_si_002.zip [file es2c09333_si_002.zip › SSD_Ecoregion/Lake Chad.tif]

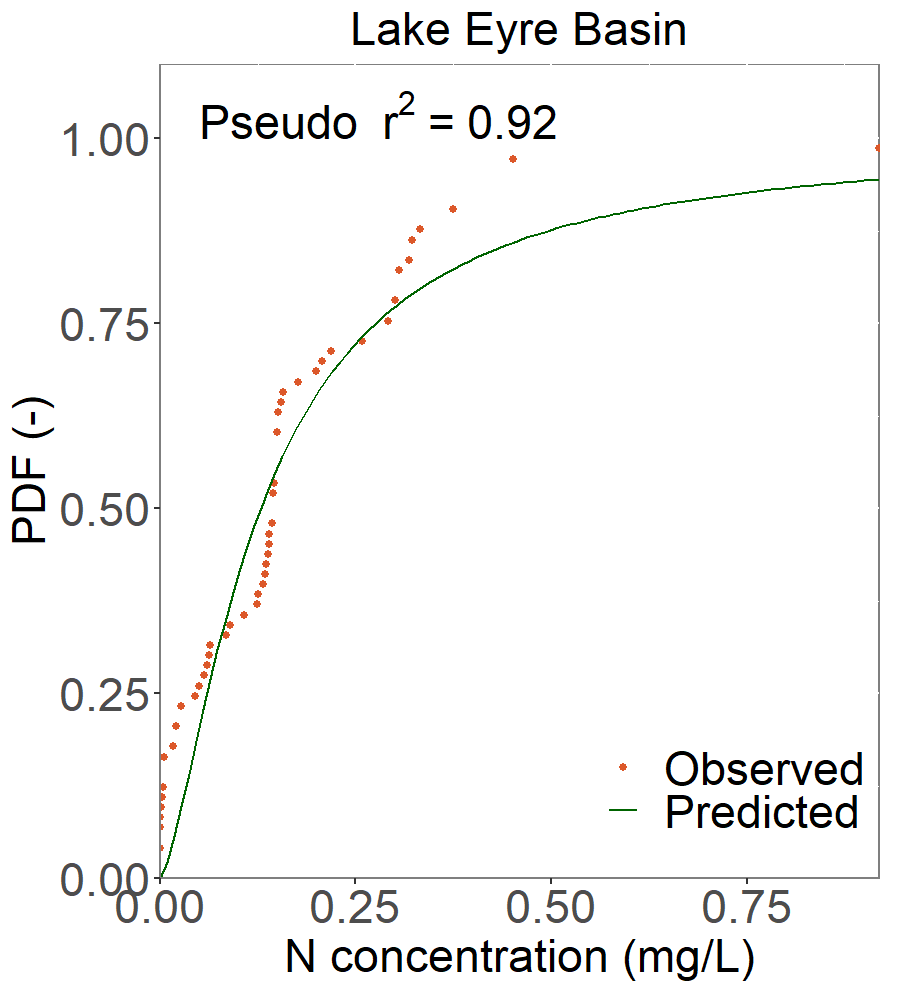

Supplement: Supplementary file 2 — es2c09333_si_002.zip [file es2c09333_si_002.zip › SSD_Ecoregion/Lake Eyre Basin.tif]

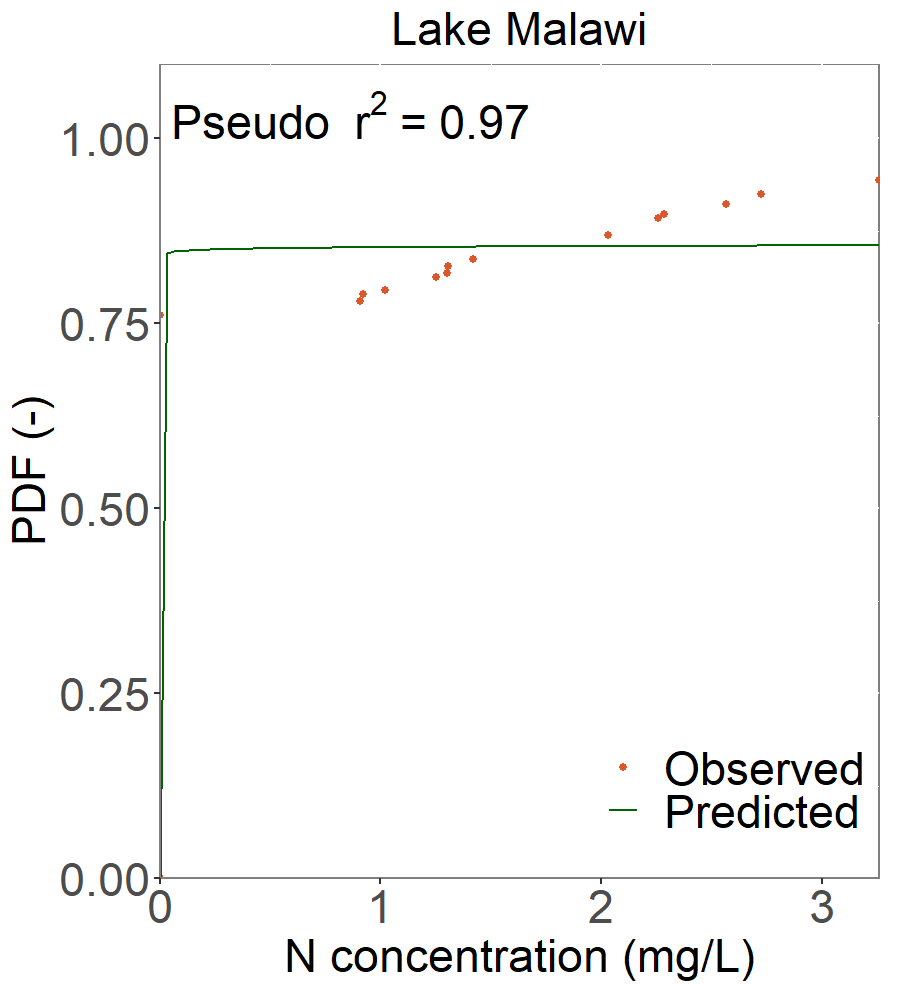

Supplement: Supplementary file 2 — es2c09333_si_002.zip [file es2c09333_si_002.zip › SSD_Ecoregion/Lake Malawi.tif]

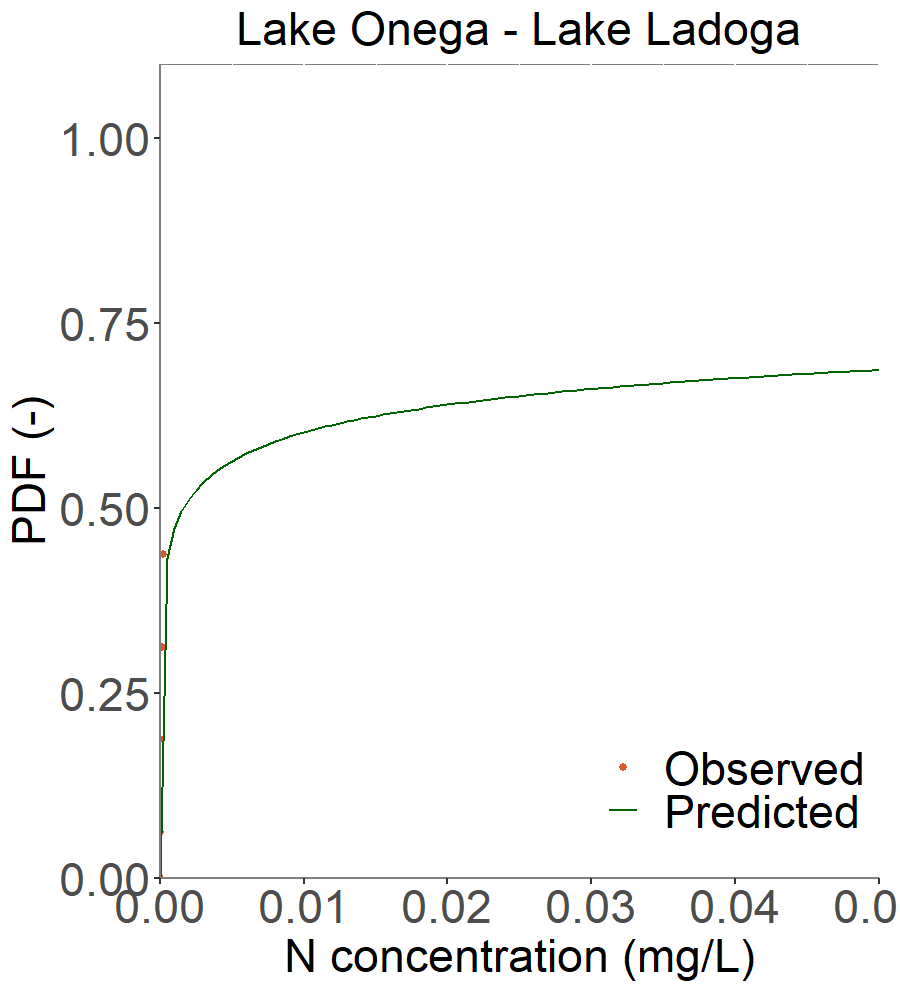

Supplement: Supplementary file 2 — es2c09333_si_002.zip [file es2c09333_si_002.zip › SSD_Ecoregion/Lake Onega - Lake Ladoga.tif]

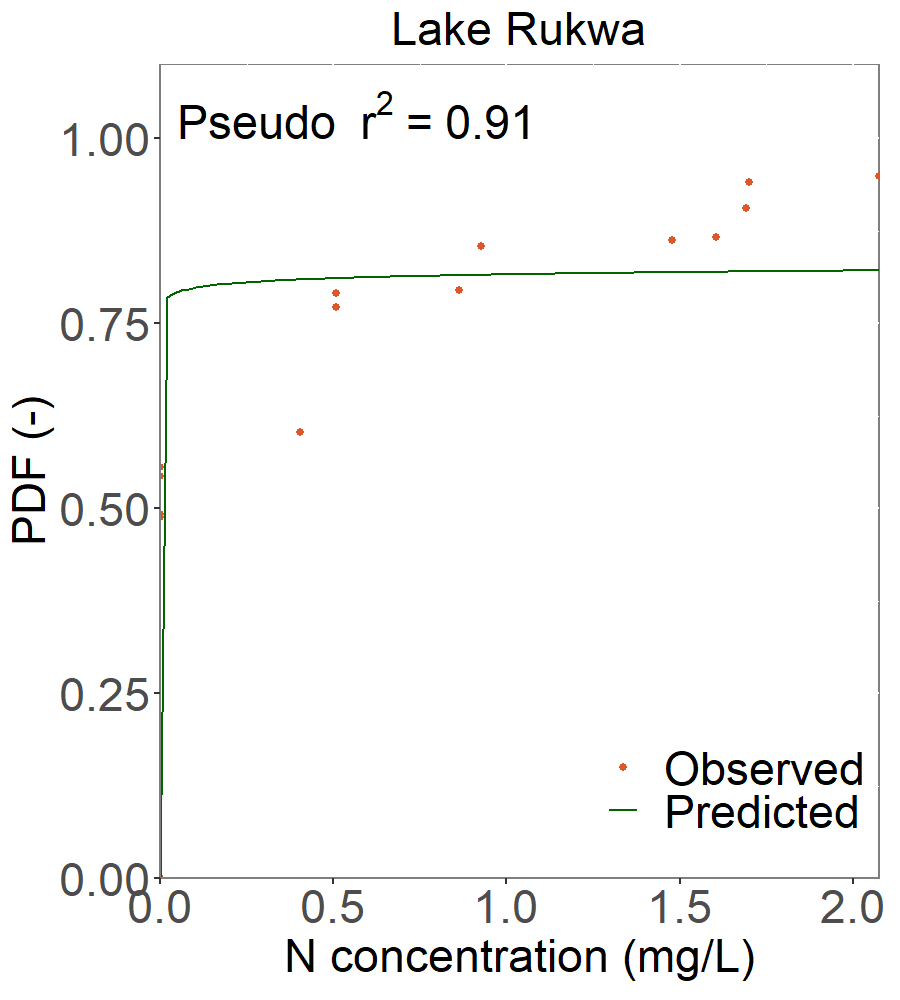

Supplement: Supplementary file 2 — es2c09333_si_002.zip [file es2c09333_si_002.zip › SSD_Ecoregion/Lake Rukwa.tif]

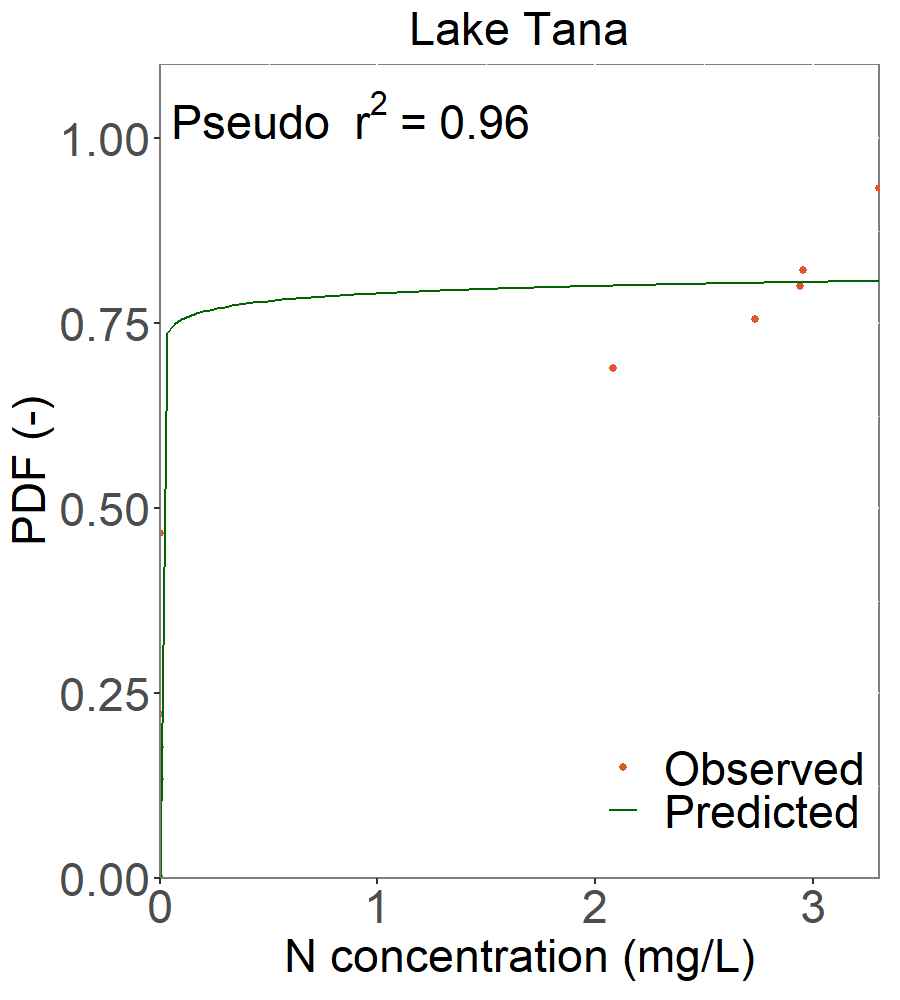

Supplement: Supplementary file 2 — es2c09333_si_002.zip [file es2c09333_si_002.zip › SSD_Ecoregion/Lake Tana.tif]

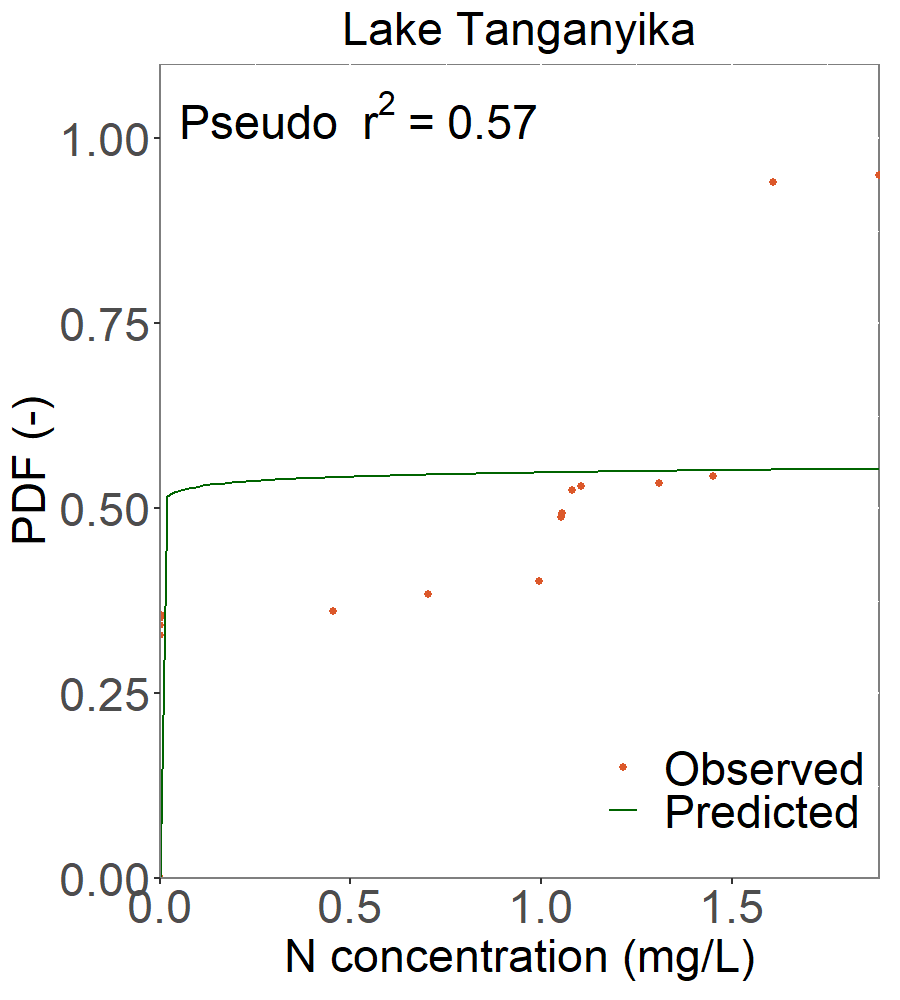

Supplement: Supplementary file 2 — es2c09333_si_002.zip [file es2c09333_si_002.zip › SSD_Ecoregion/Lake Tanganyika.tif]

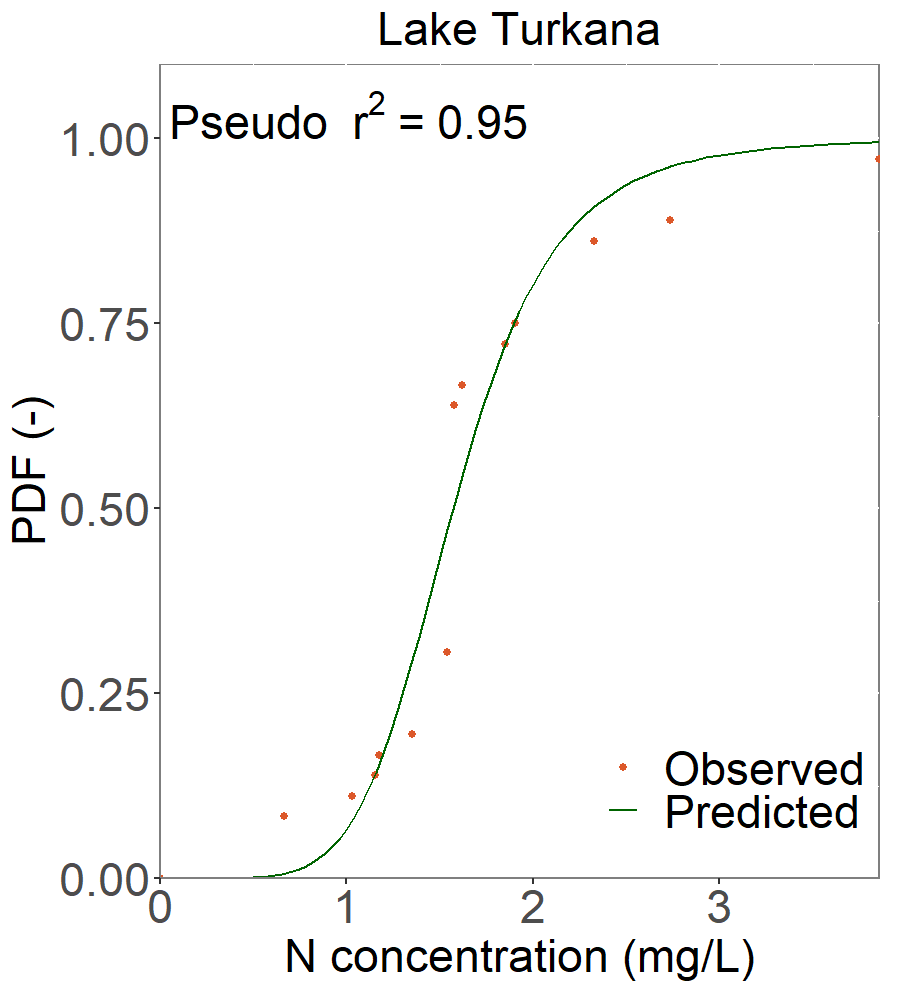

Supplement: Supplementary file 2 — es2c09333_si_002.zip [file es2c09333_si_002.zip › SSD_Ecoregion/Lake Turkana.tif]

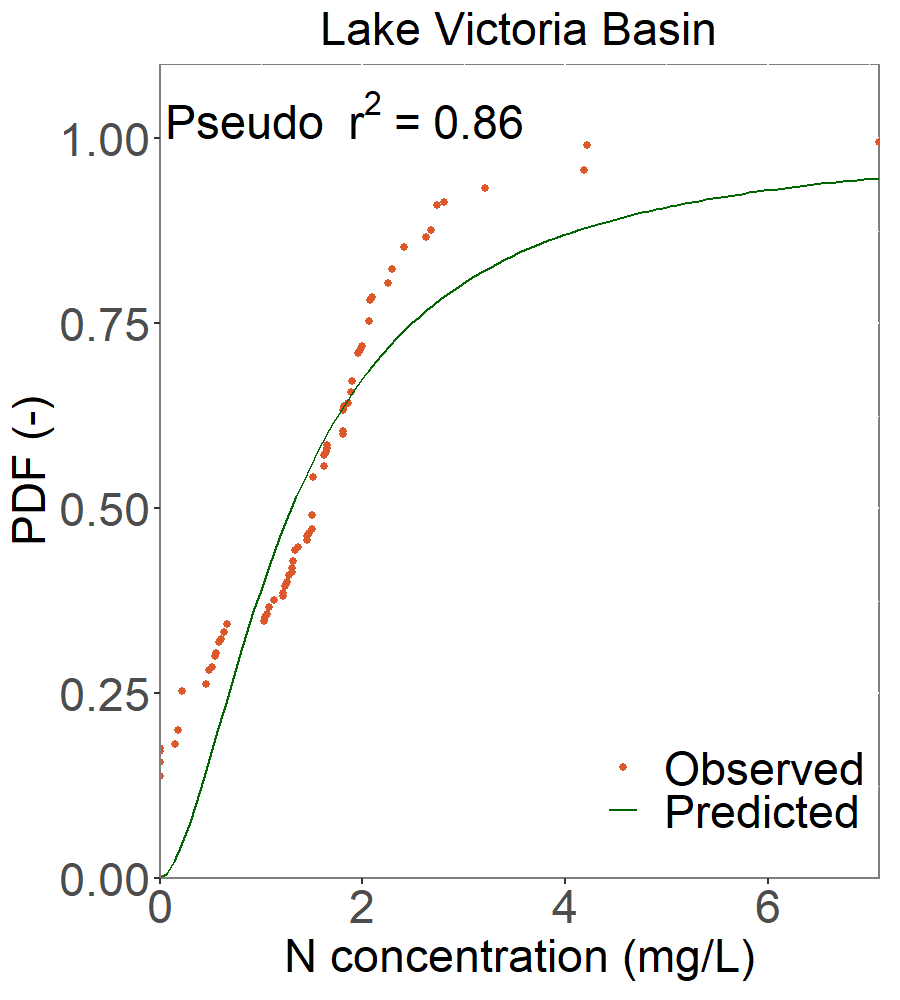

Supplement: Supplementary file 2 — es2c09333_si_002.zip [file es2c09333_si_002.zip › SSD_Ecoregion/Lake Victoria Basin.tif]

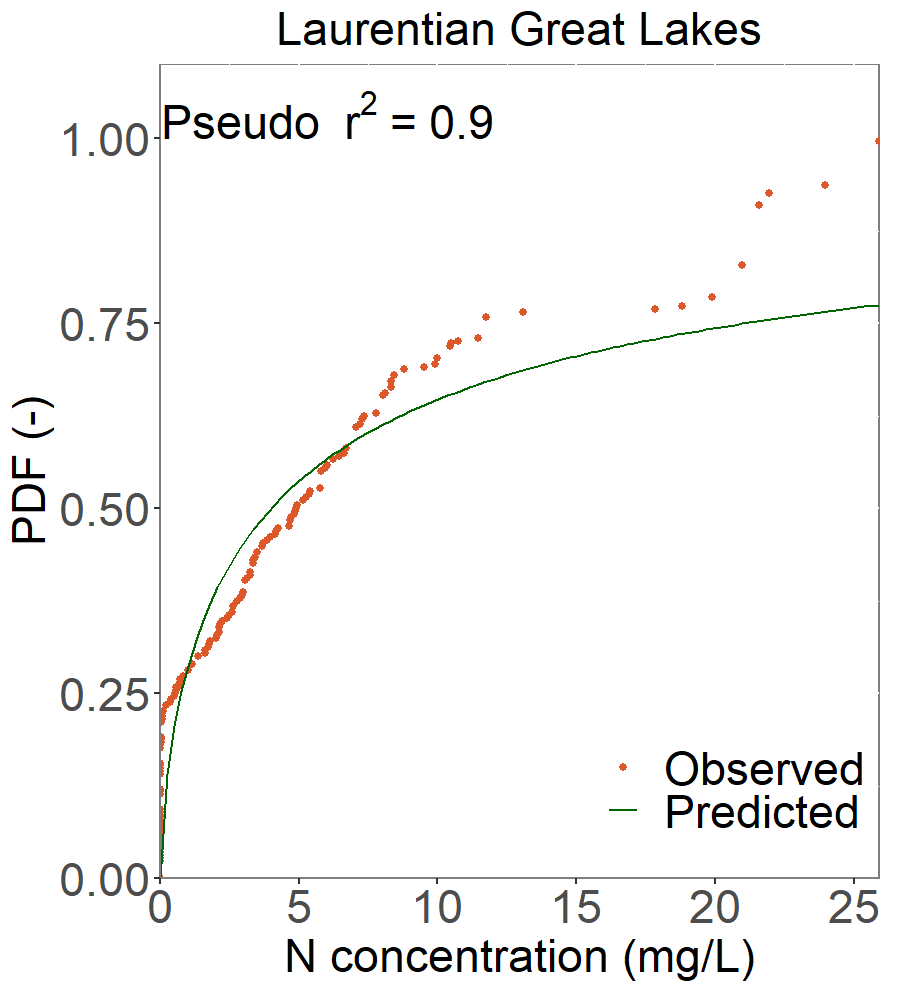

Supplement: Supplementary file 2 — es2c09333_si_002.zip [file es2c09333_si_002.zip › SSD_Ecoregion/Laurentian Great Lakes.tif]

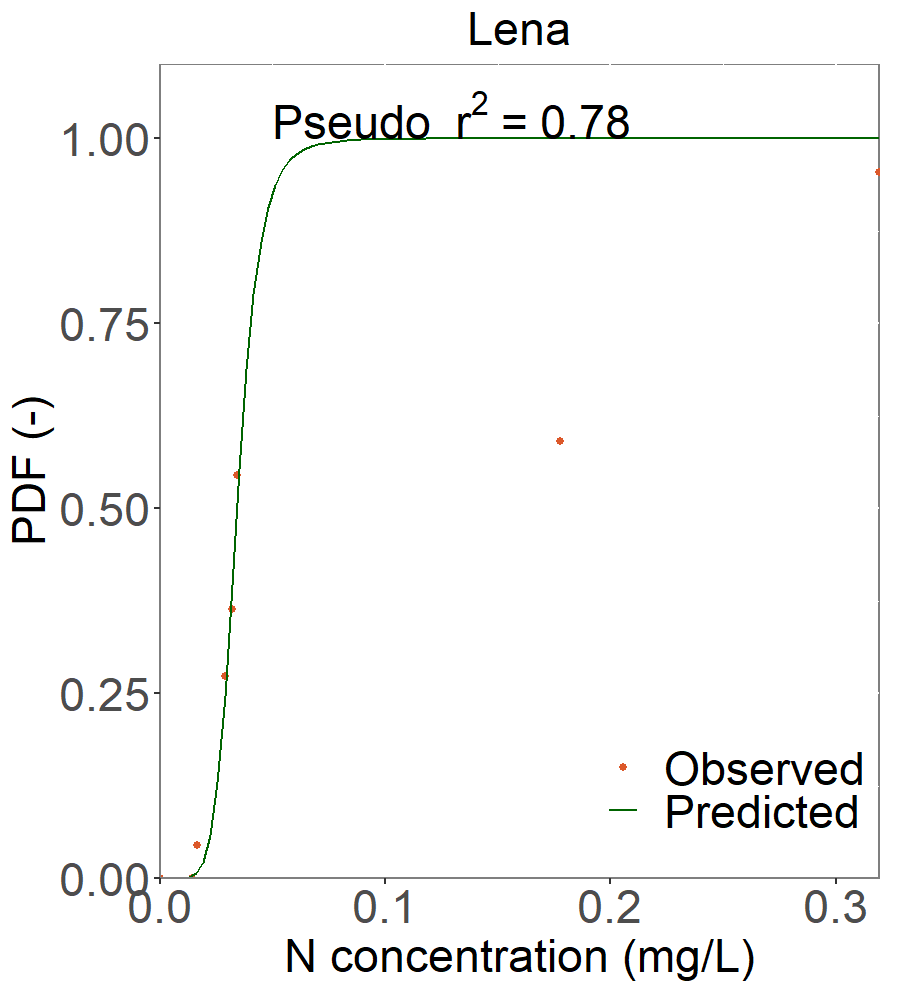

Supplement: Supplementary file 2 — es2c09333_si_002.zip [file es2c09333_si_002.zip › SSD_Ecoregion/Lena.tif]

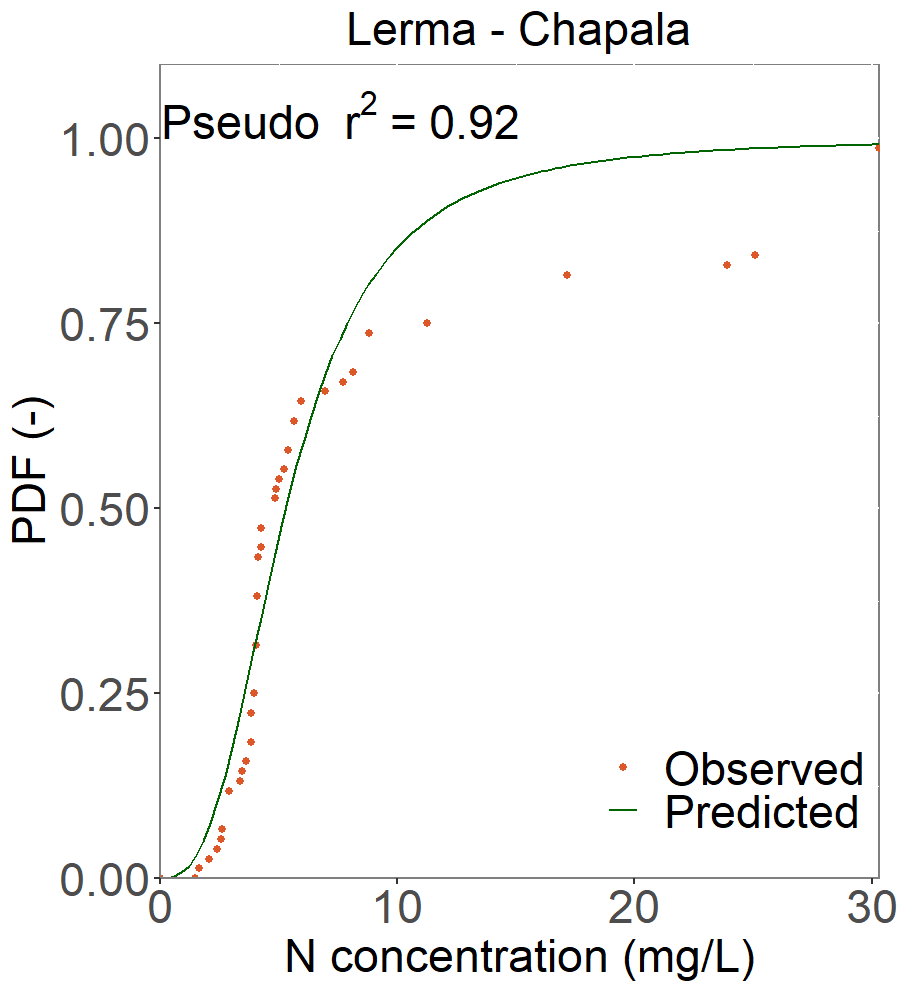

Supplement: Supplementary file 2 — es2c09333_si_002.zip [file es2c09333_si_002.zip › SSD_Ecoregion/Lerma - Chapala.tif]

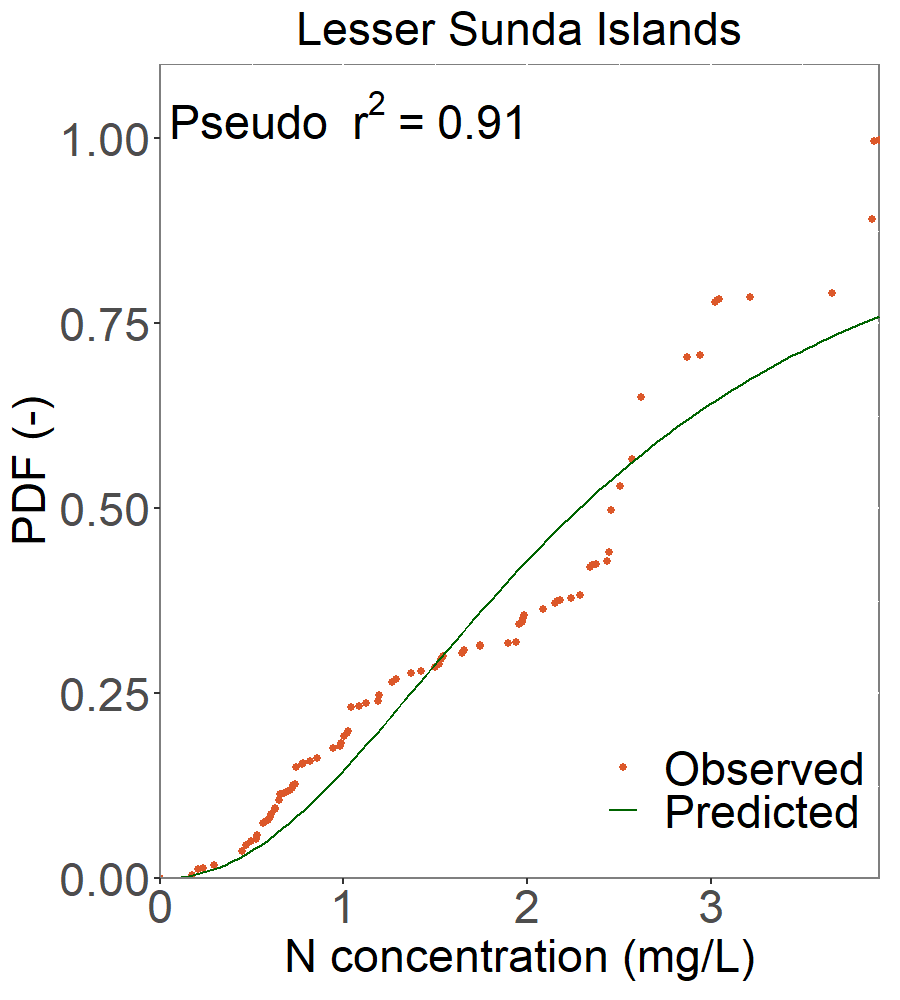

Supplement: Supplementary file 2 — es2c09333_si_002.zip [file es2c09333_si_002.zip › SSD_Ecoregion/Lesser Sunda Islands.tif]

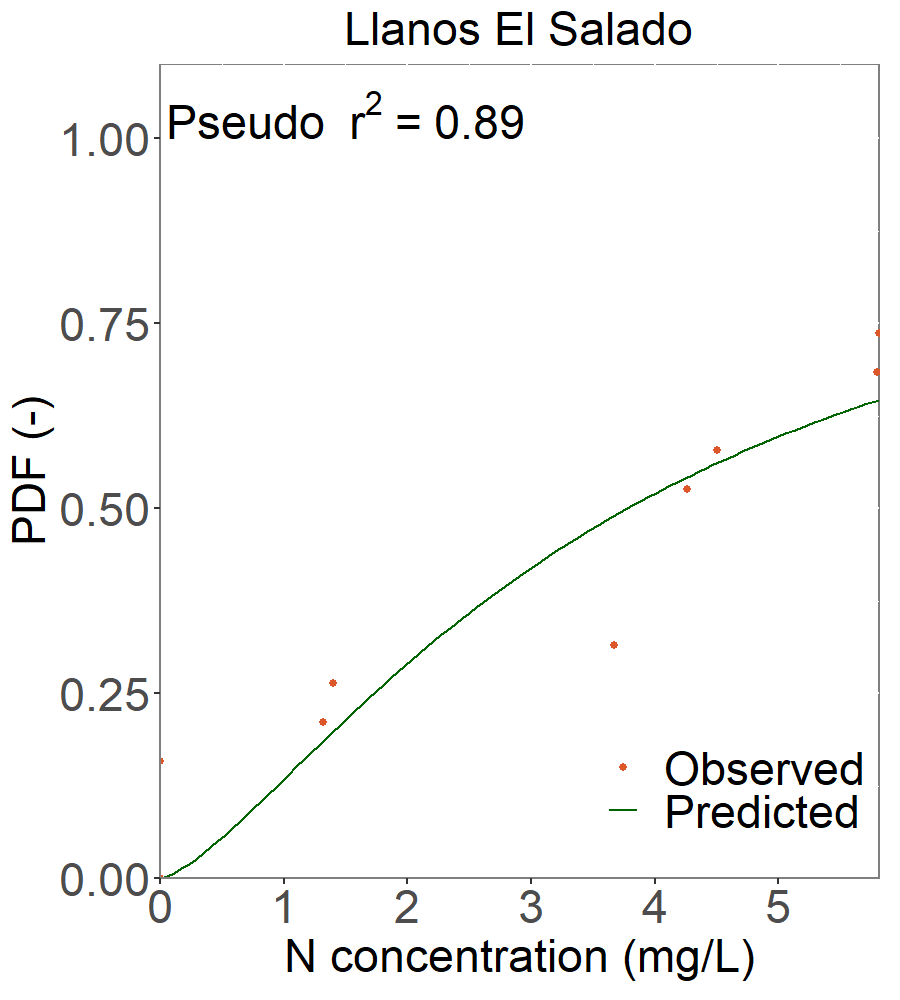

Supplement: Supplementary file 2 — es2c09333_si_002.zip [file es2c09333_si_002.zip › SSD_Ecoregion/Llanos El Salado.tif]

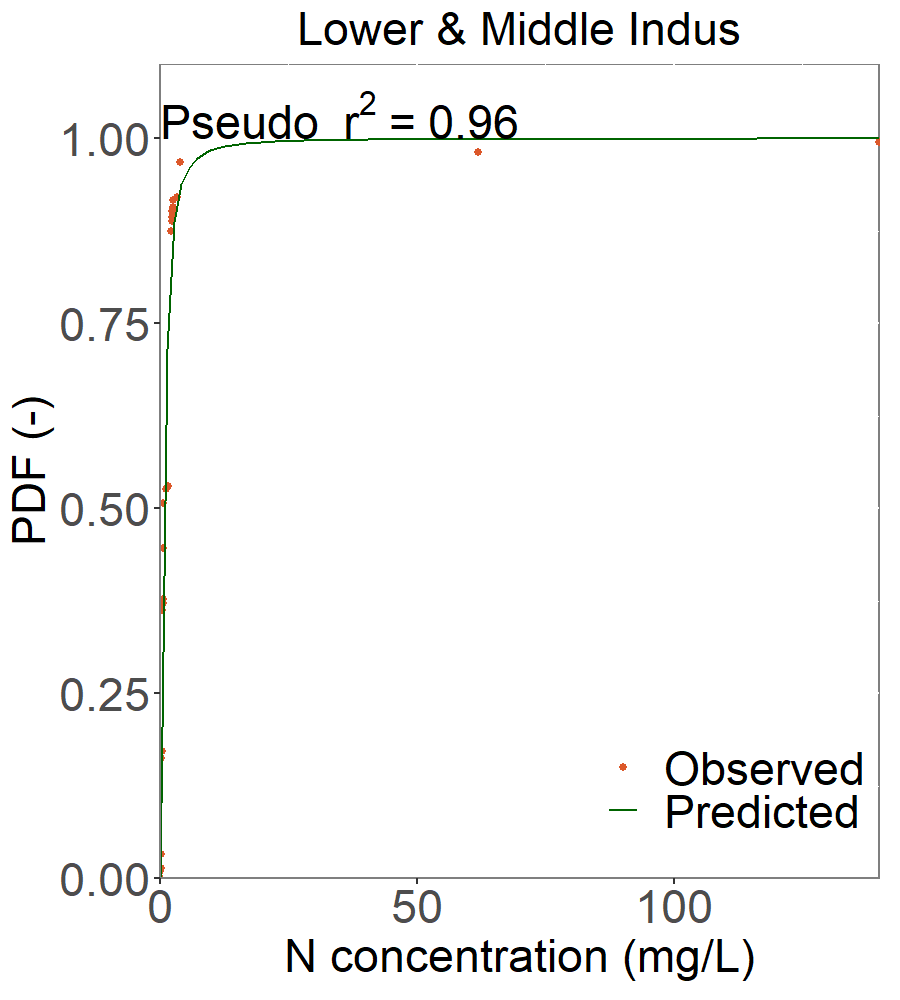

Supplement: Supplementary file 2 — es2c09333_si_002.zip [file es2c09333_si_002.zip › SSD_Ecoregion/Lower & Middle Indus.tif]

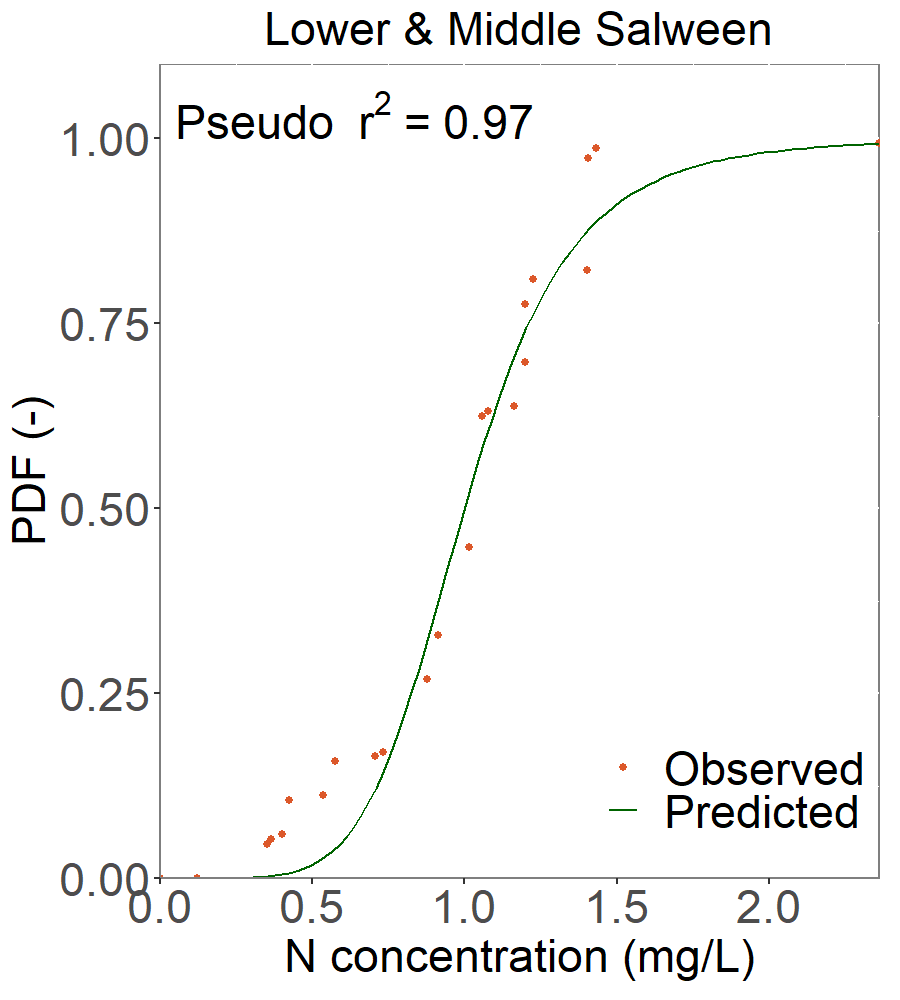

Supplement: Supplementary file 2 — es2c09333_si_002.zip [file es2c09333_si_002.zip › SSD_Ecoregion/Lower & Middle Salween.tif]

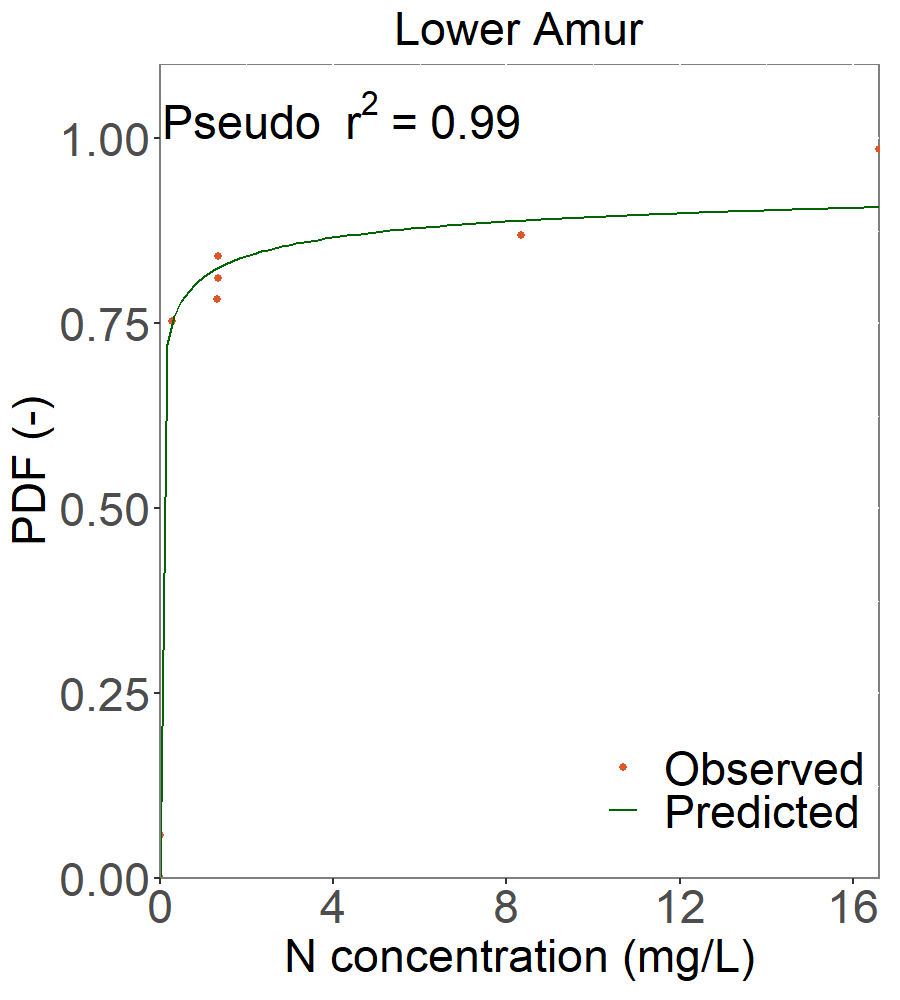

Supplement: Supplementary file 2 — es2c09333_si_002.zip [file es2c09333_si_002.zip › SSD_Ecoregion/Lower Amur.tif]

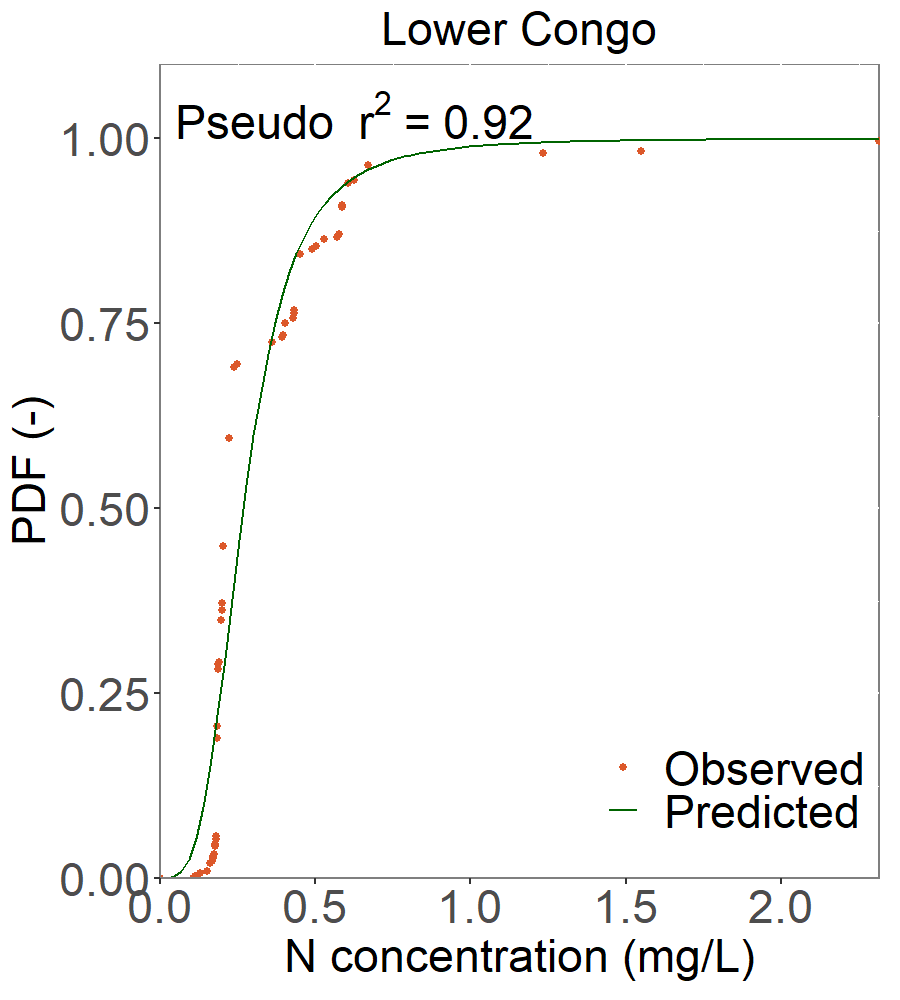

Supplement: Supplementary file 2 — es2c09333_si_002.zip [file es2c09333_si_002.zip › SSD_Ecoregion/Lower Congo.tif]

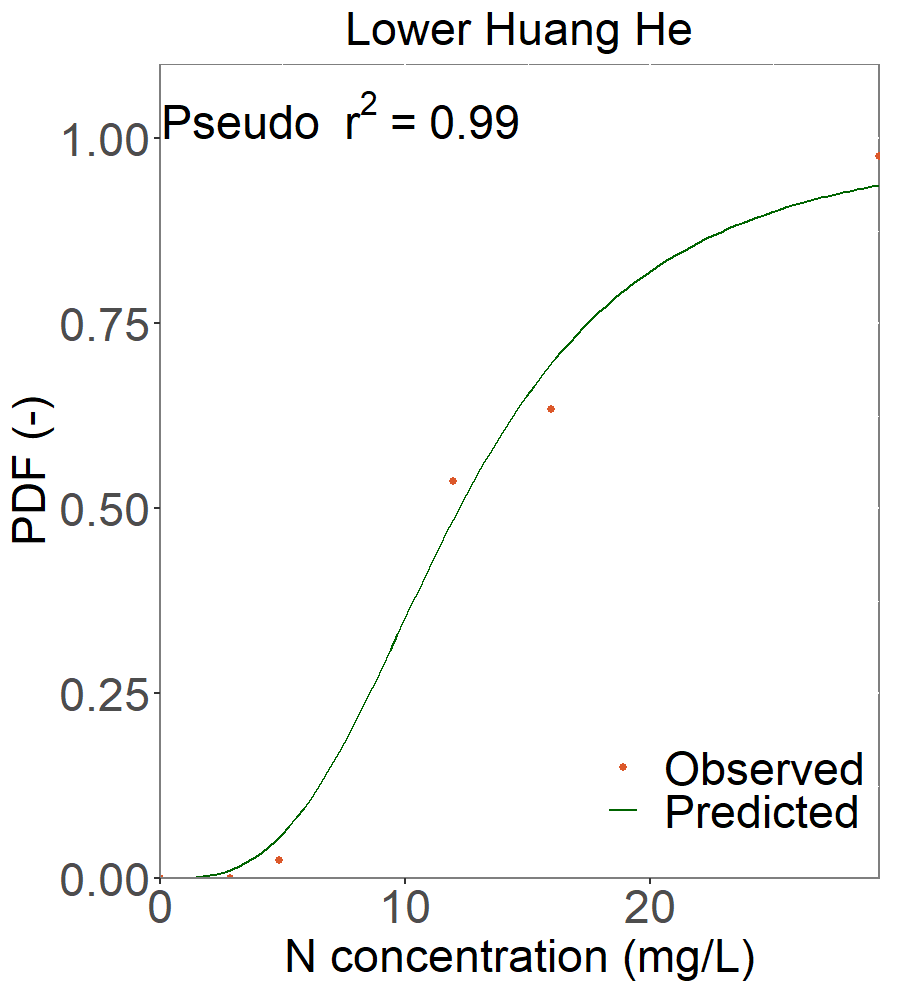

Supplement: Supplementary file 2 — es2c09333_si_002.zip [file es2c09333_si_002.zip › SSD_Ecoregion/Lower Huang He.tif]

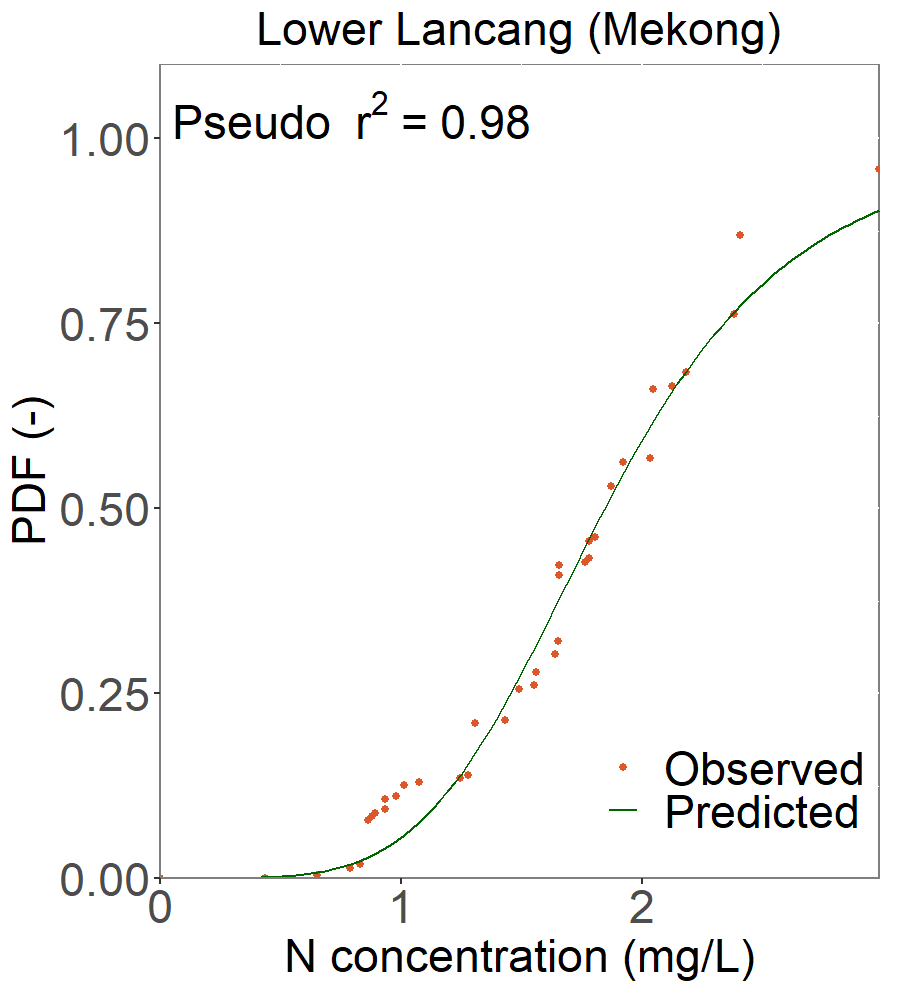

Supplement: Supplementary file 2 — es2c09333_si_002.zip [file es2c09333_si_002.zip › SSD_Ecoregion/Lower Lancang (Mekong).tif]

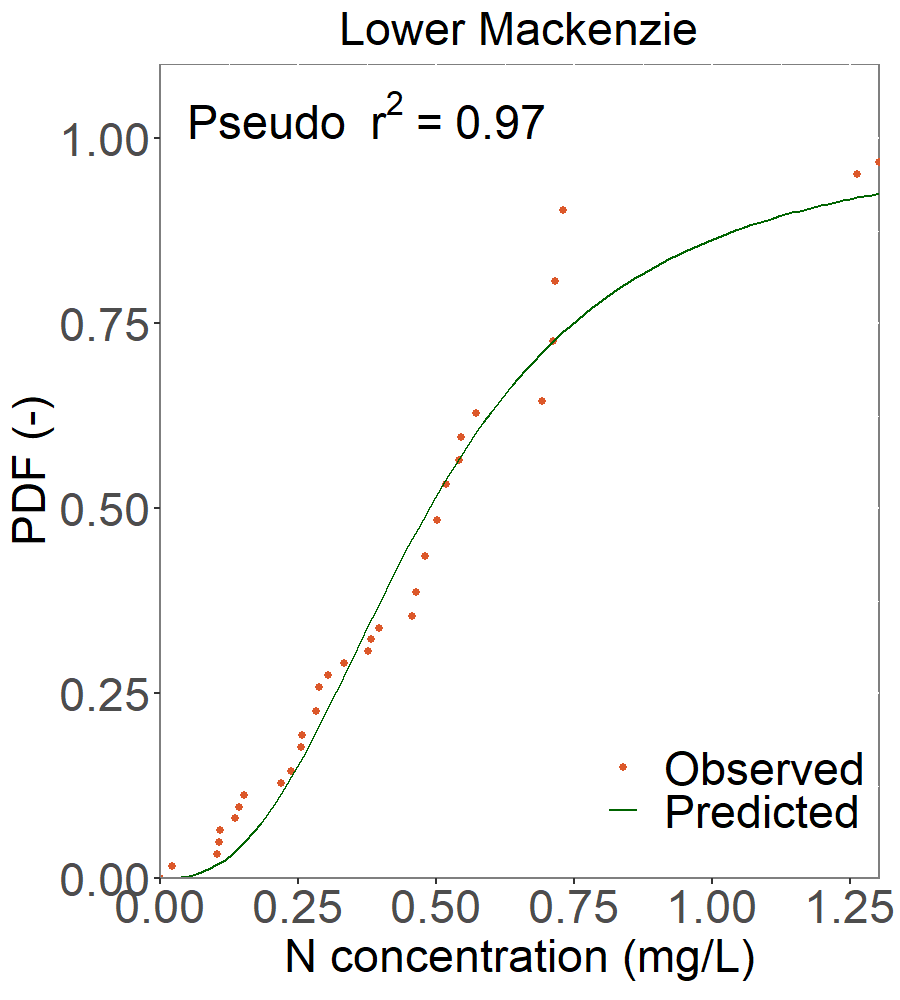

Supplement: Supplementary file 2 — es2c09333_si_002.zip [file es2c09333_si_002.zip › SSD_Ecoregion/Lower Mackenzie.tif]

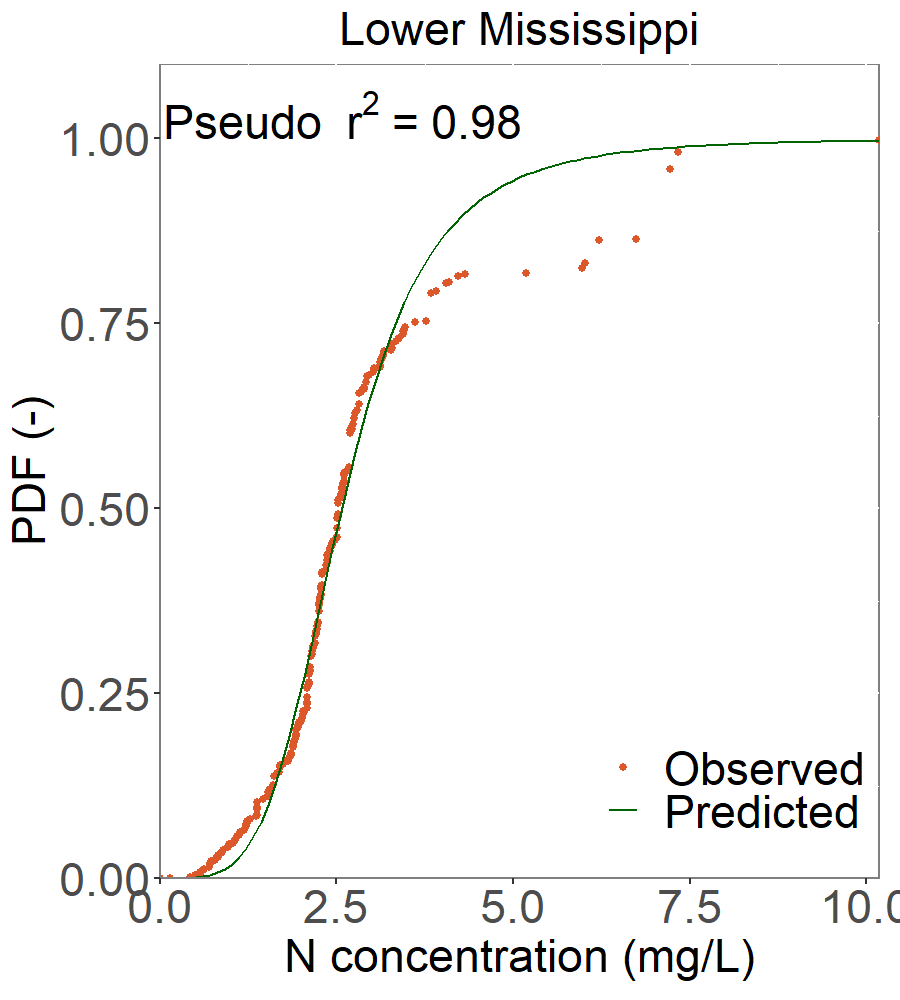

Supplement: Supplementary file 2 — es2c09333_si_002.zip [file es2c09333_si_002.zip › SSD_Ecoregion/Lower Mississippi.tif]

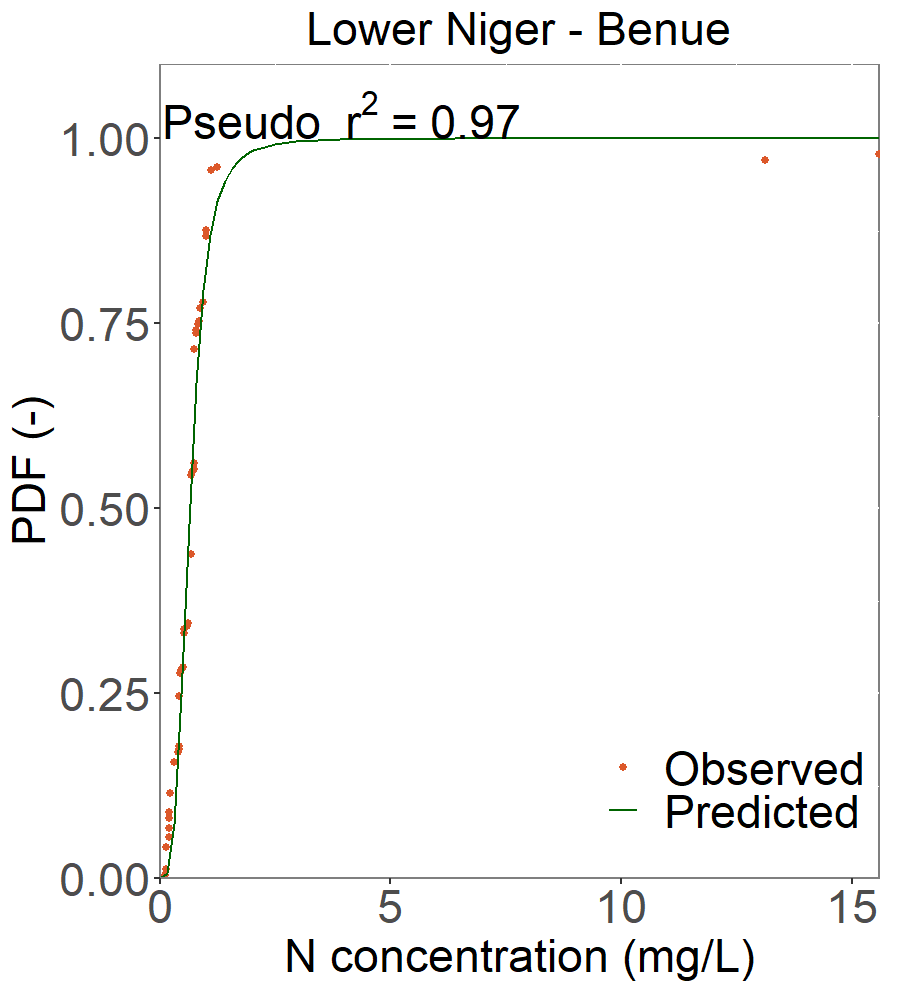

Supplement: Supplementary file 2 — es2c09333_si_002.zip [file es2c09333_si_002.zip › SSD_Ecoregion/Lower Niger - Benue.tif]

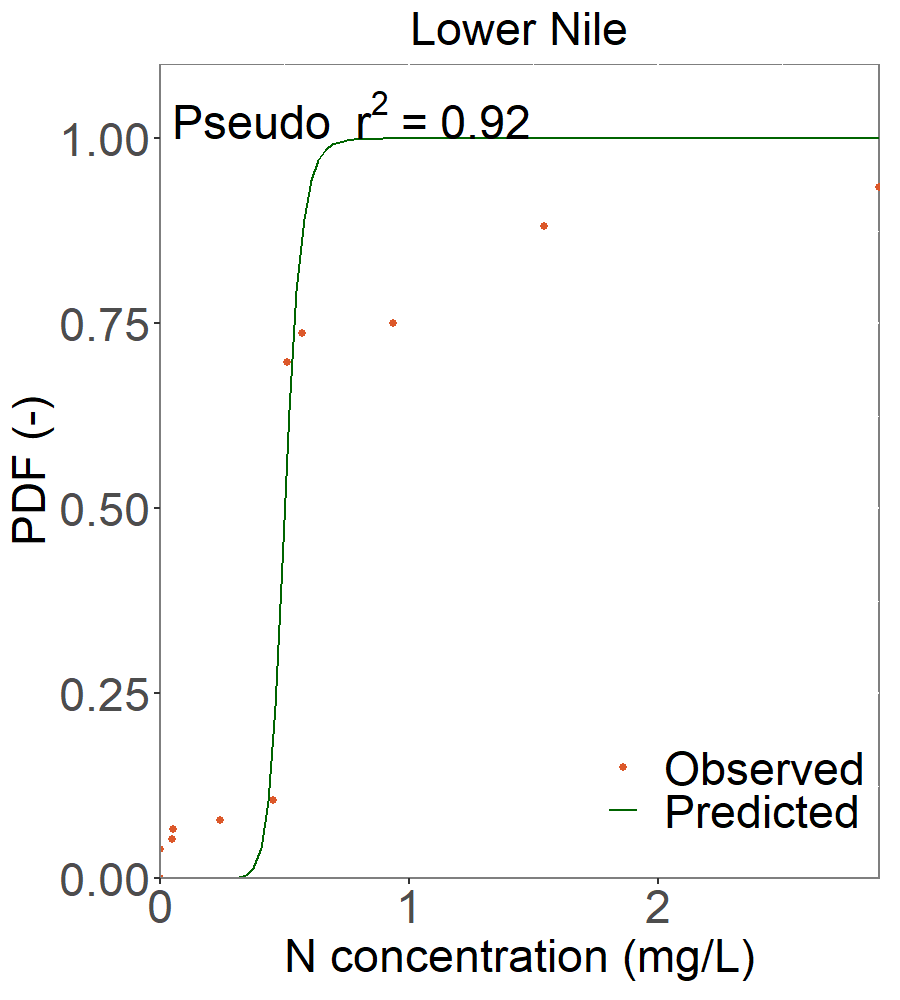

Supplement: Supplementary file 2 — es2c09333_si_002.zip [file es2c09333_si_002.zip › SSD_Ecoregion/Lower Nile.tif]

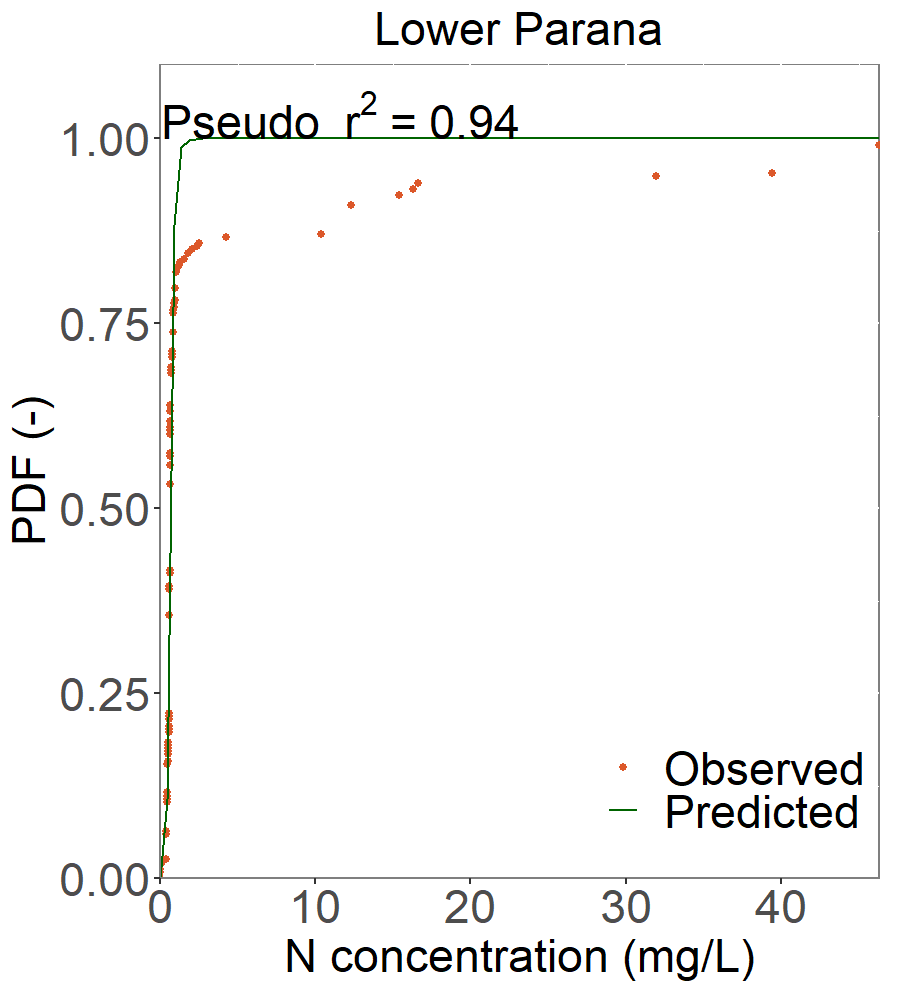

Supplement: Supplementary file 2 — es2c09333_si_002.zip [file es2c09333_si_002.zip › SSD_Ecoregion/Lower Parana.tif]

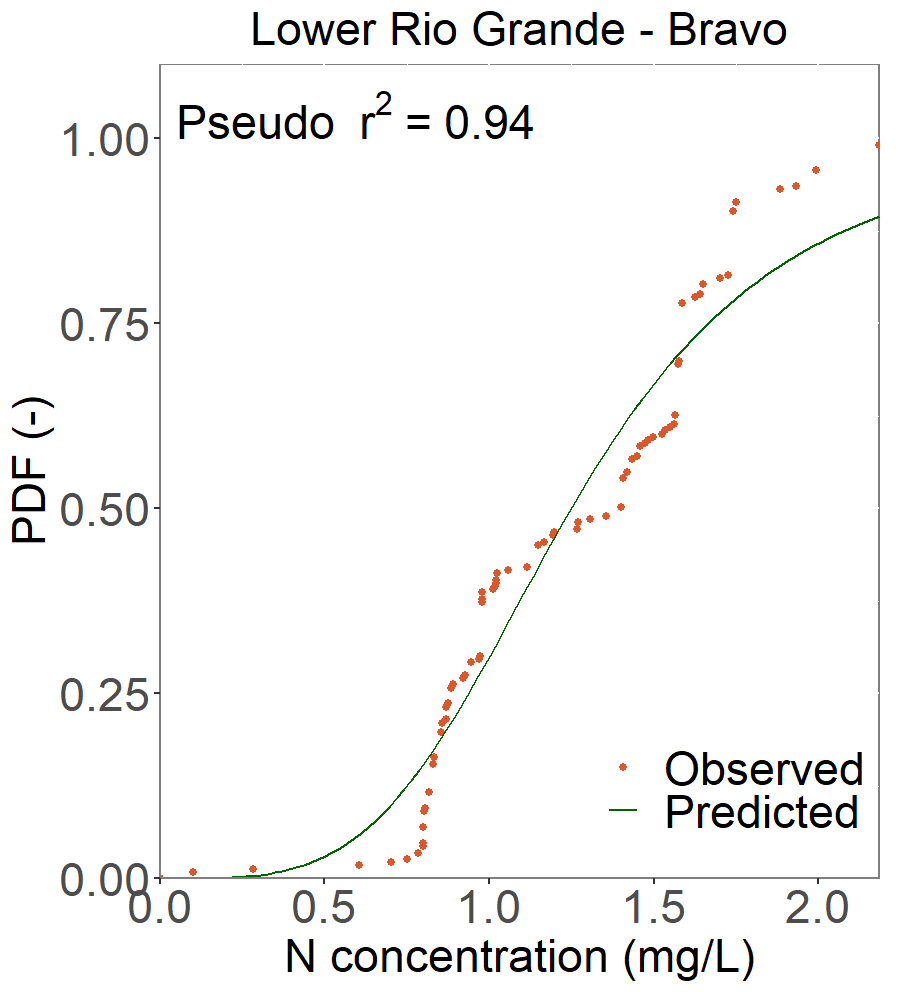

Supplement: Supplementary file 2 — es2c09333_si_002.zip [file es2c09333_si_002.zip › SSD_Ecoregion/Lower Rio Grande - Bravo.tif]

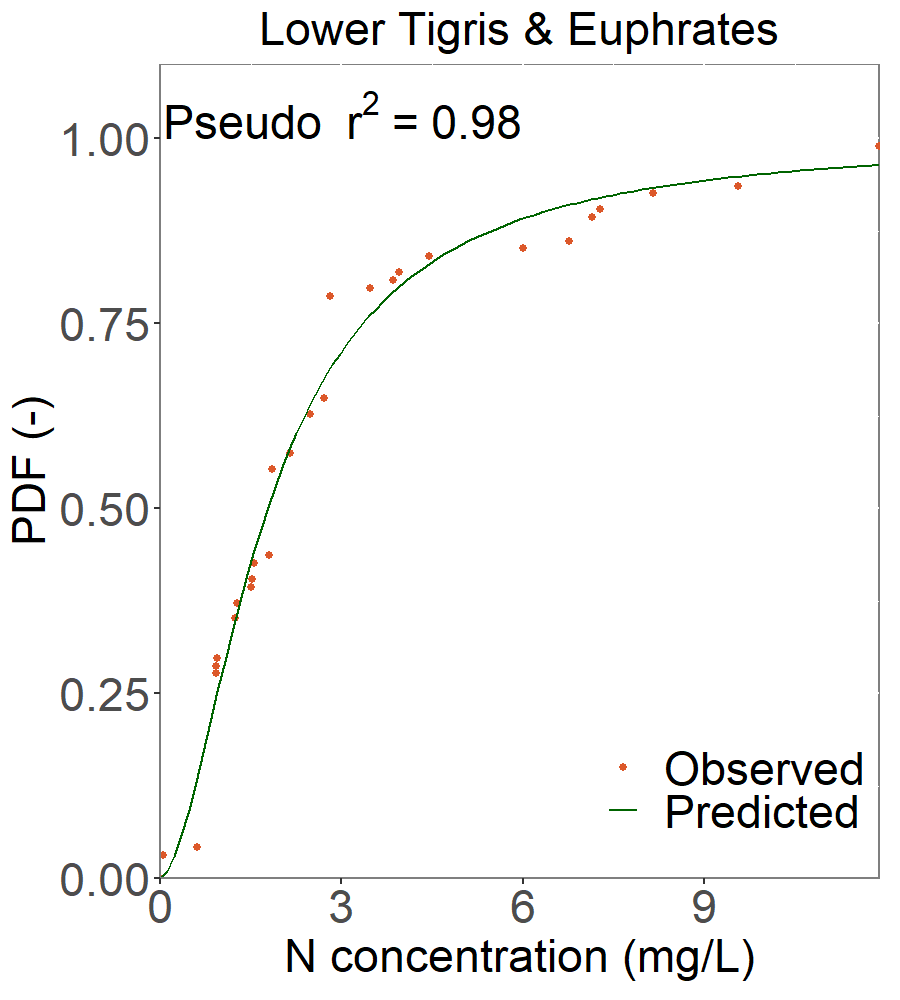

Supplement: Supplementary file 2 — es2c09333_si_002.zip [file es2c09333_si_002.zip › SSD_Ecoregion/Lower Tigris & Euphrates.tif]

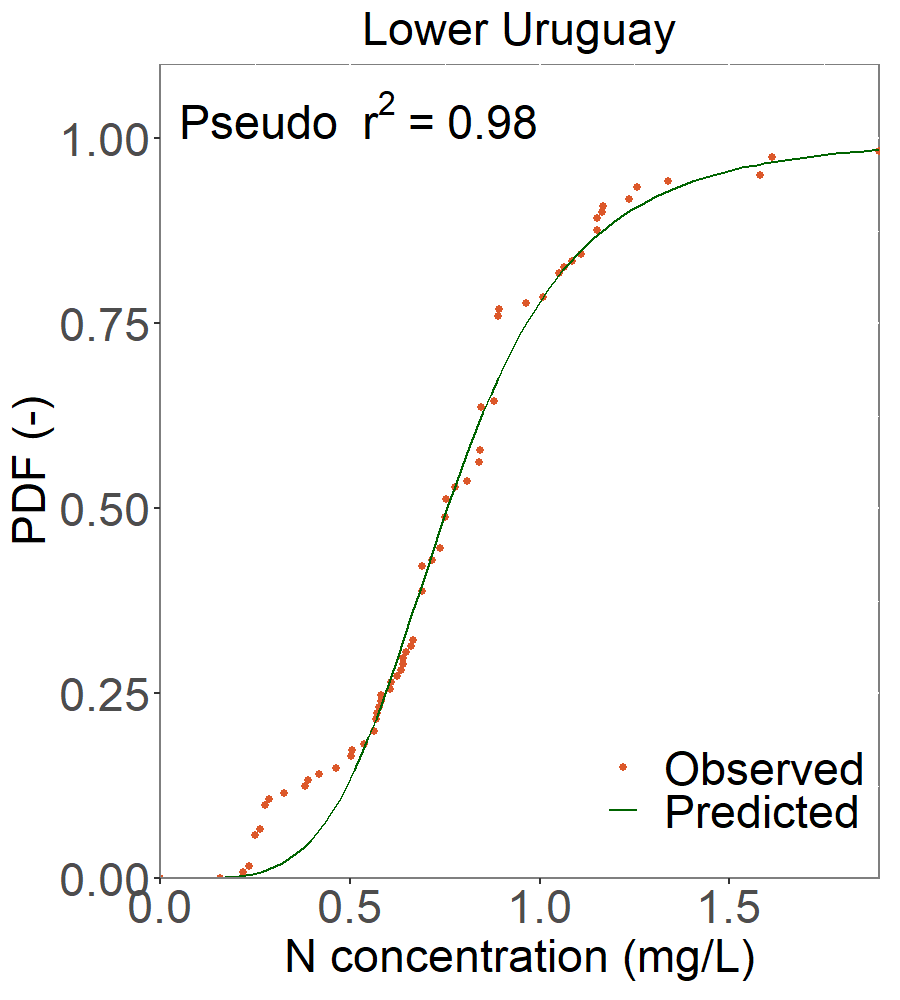

Supplement: Supplementary file 2 — es2c09333_si_002.zip [file es2c09333_si_002.zip › SSD_Ecoregion/Lower Uruguay.tif]

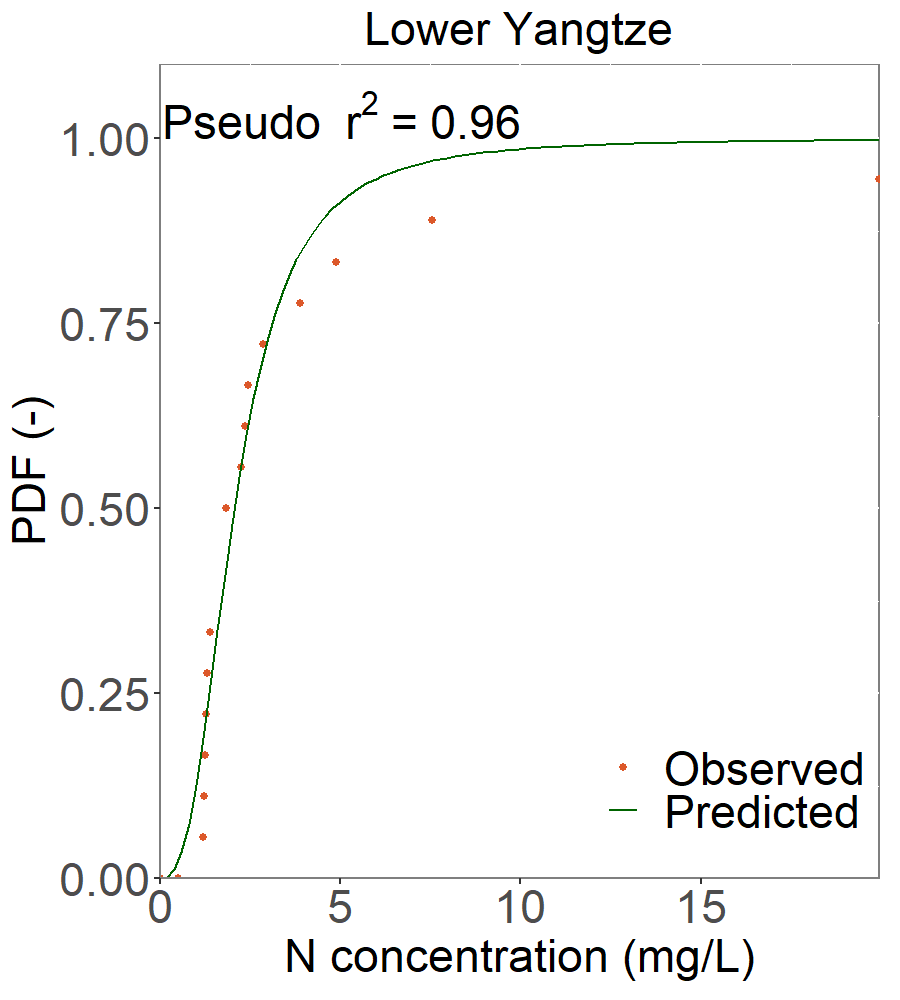

Supplement: Supplementary file 2 — es2c09333_si_002.zip [file es2c09333_si_002.zip › SSD_Ecoregion/Lower Yangtze.tif]

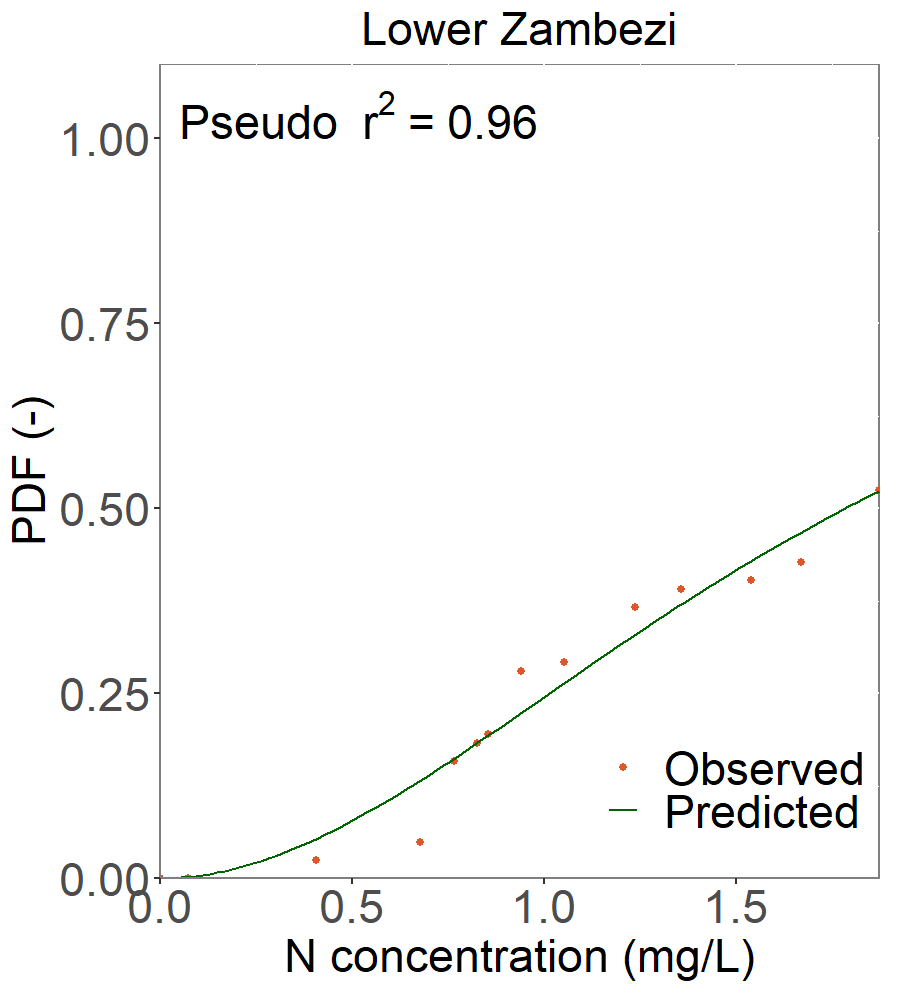

Supplement: Supplementary file 2 — es2c09333_si_002.zip [file es2c09333_si_002.zip › SSD_Ecoregion/Lower Zambezi.tif]

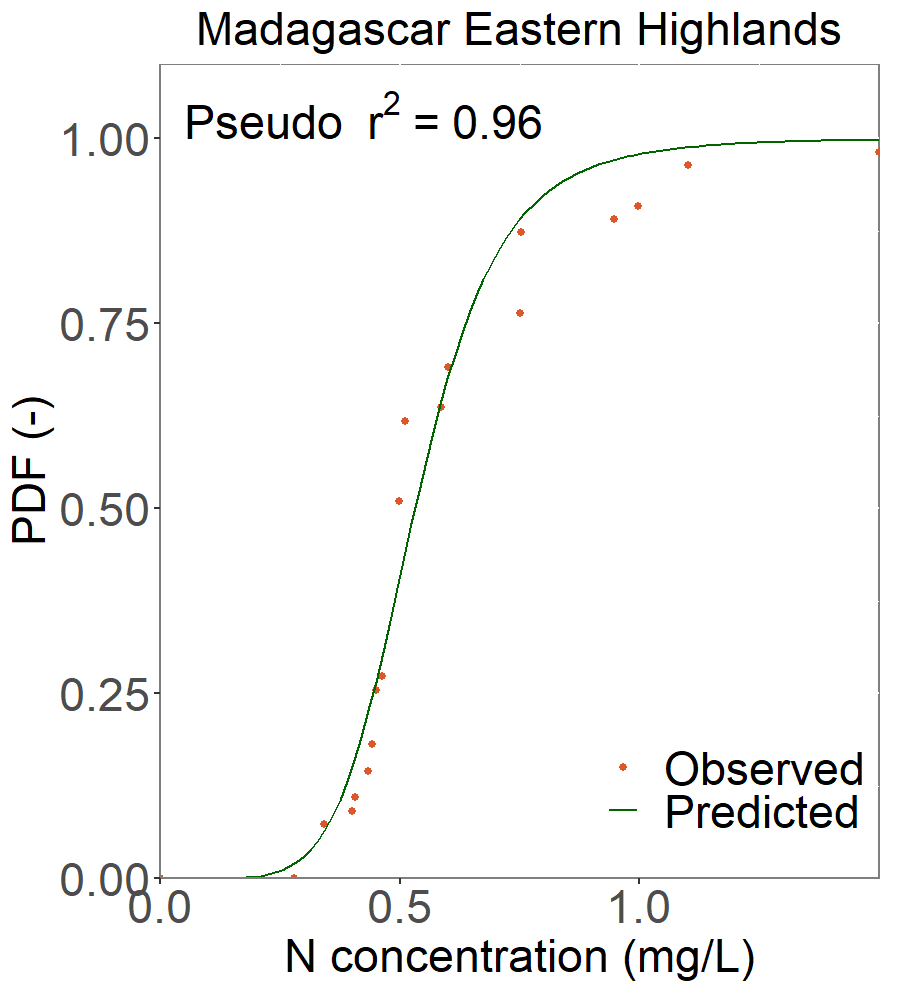

Supplement: Supplementary file 2 — es2c09333_si_002.zip [file es2c09333_si_002.zip › SSD_Ecoregion/Madagascar Eastern Highlands.tif]

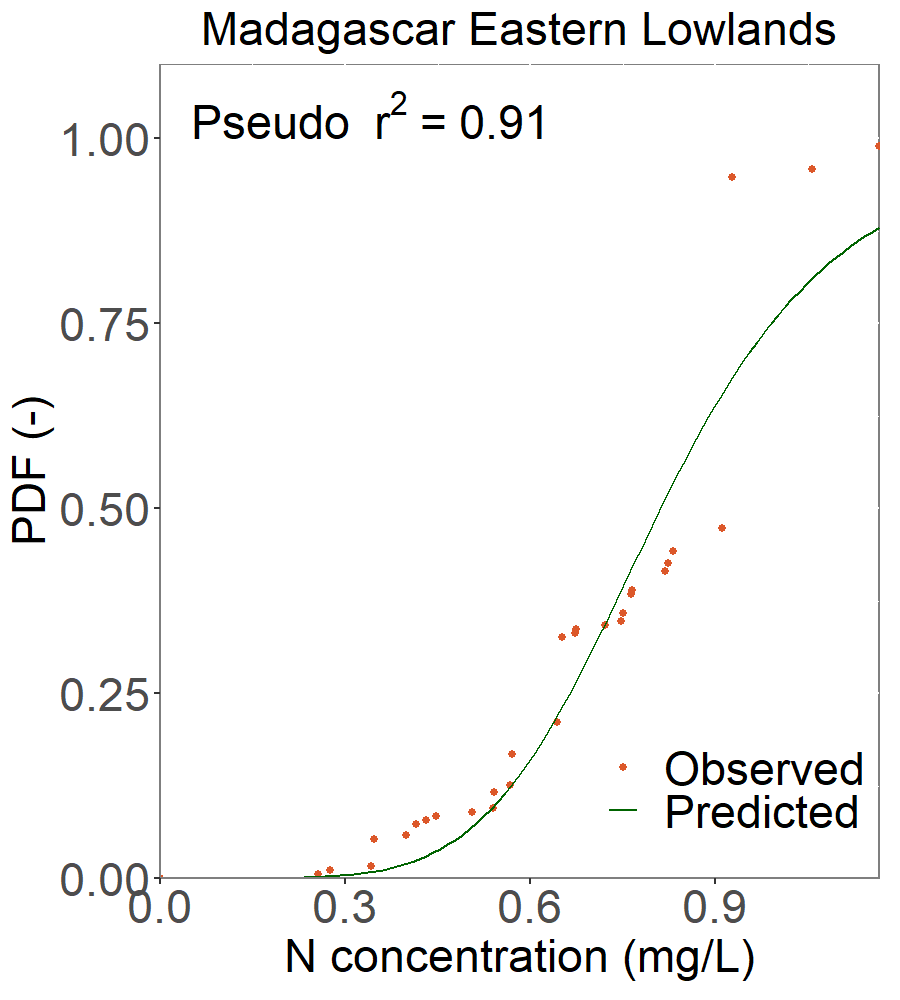

Supplement: Supplementary file 2 — es2c09333_si_002.zip [file es2c09333_si_002.zip › SSD_Ecoregion/Madagascar Eastern Lowlands.tif]

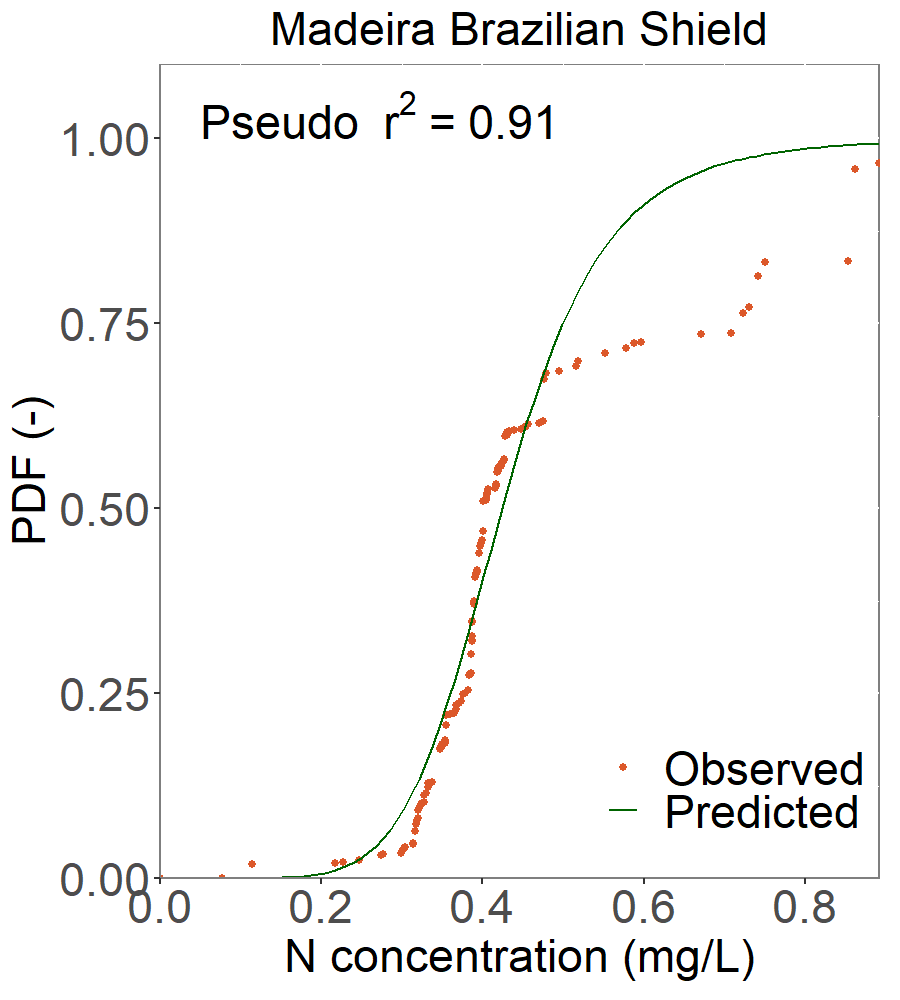

Supplement: Supplementary file 2 — es2c09333_si_002.zip [file es2c09333_si_002.zip › SSD_Ecoregion/Madeira Brazilian Shield.tif]

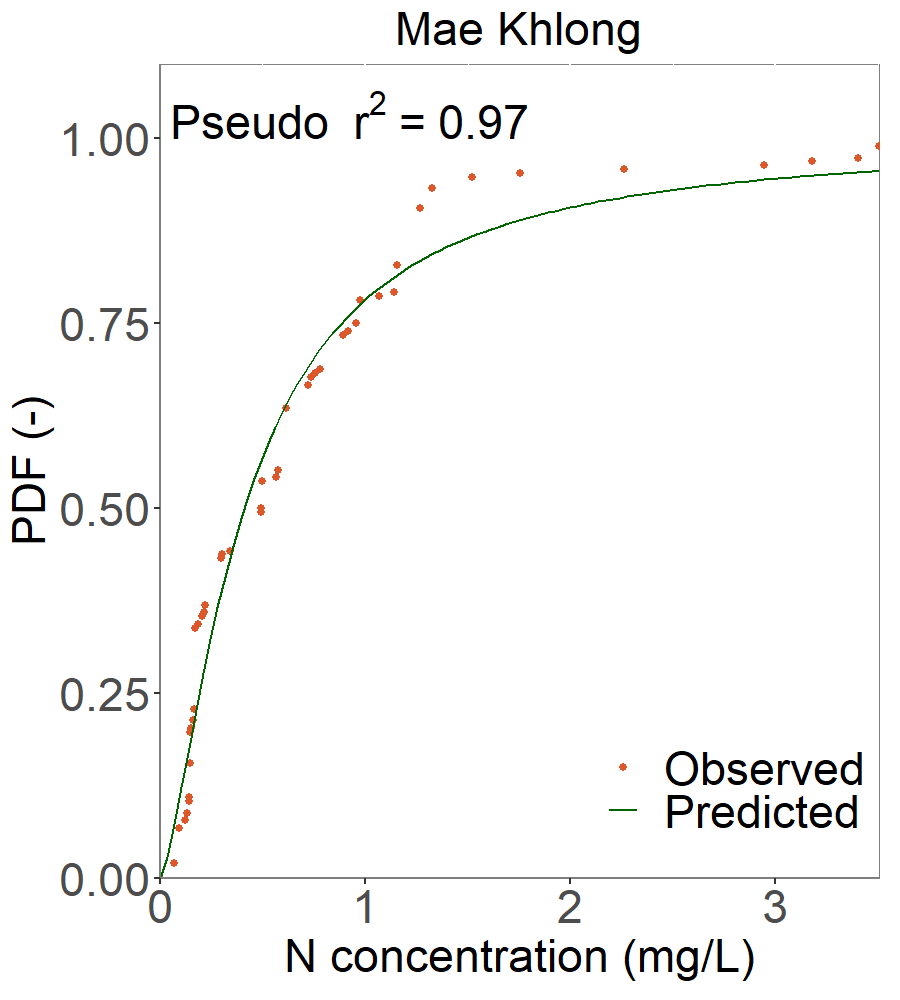

Supplement: Supplementary file 2 — es2c09333_si_002.zip [file es2c09333_si_002.zip › SSD_Ecoregion/Mae Khlong.tif]

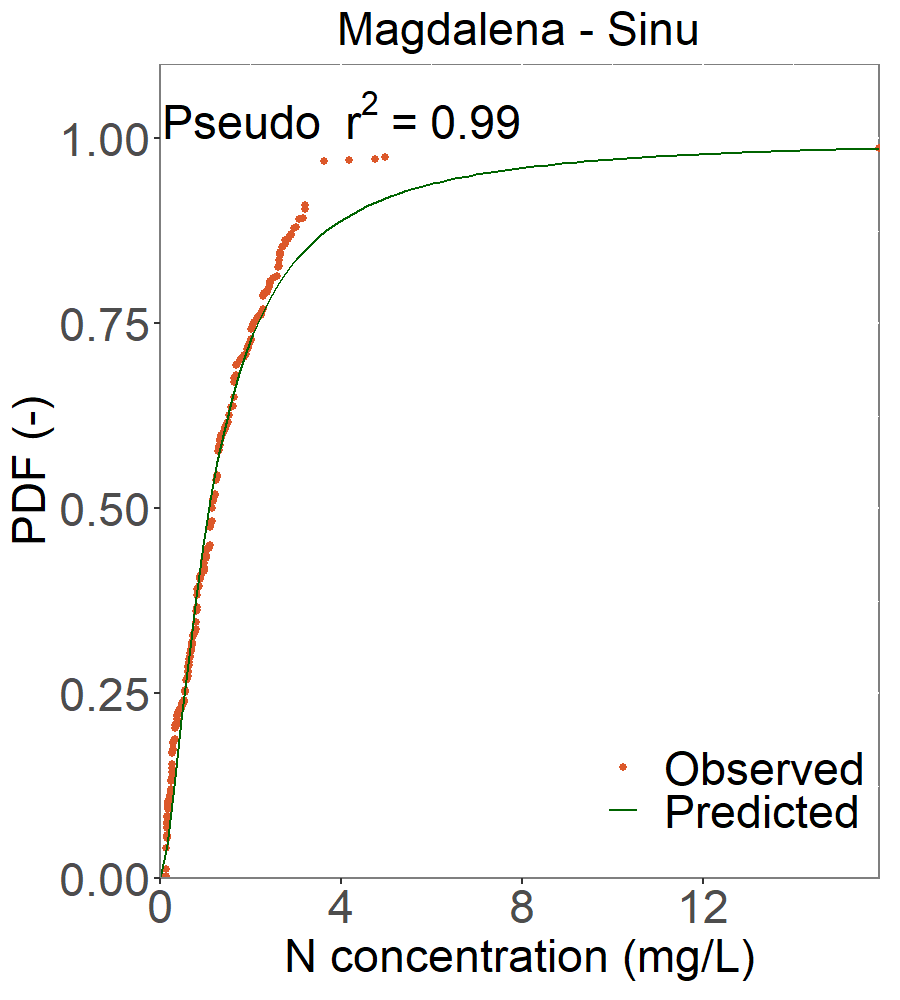

Supplement: Supplementary file 2 — es2c09333_si_002.zip [file es2c09333_si_002.zip › SSD_Ecoregion/Magdalena - Sinu.tif]

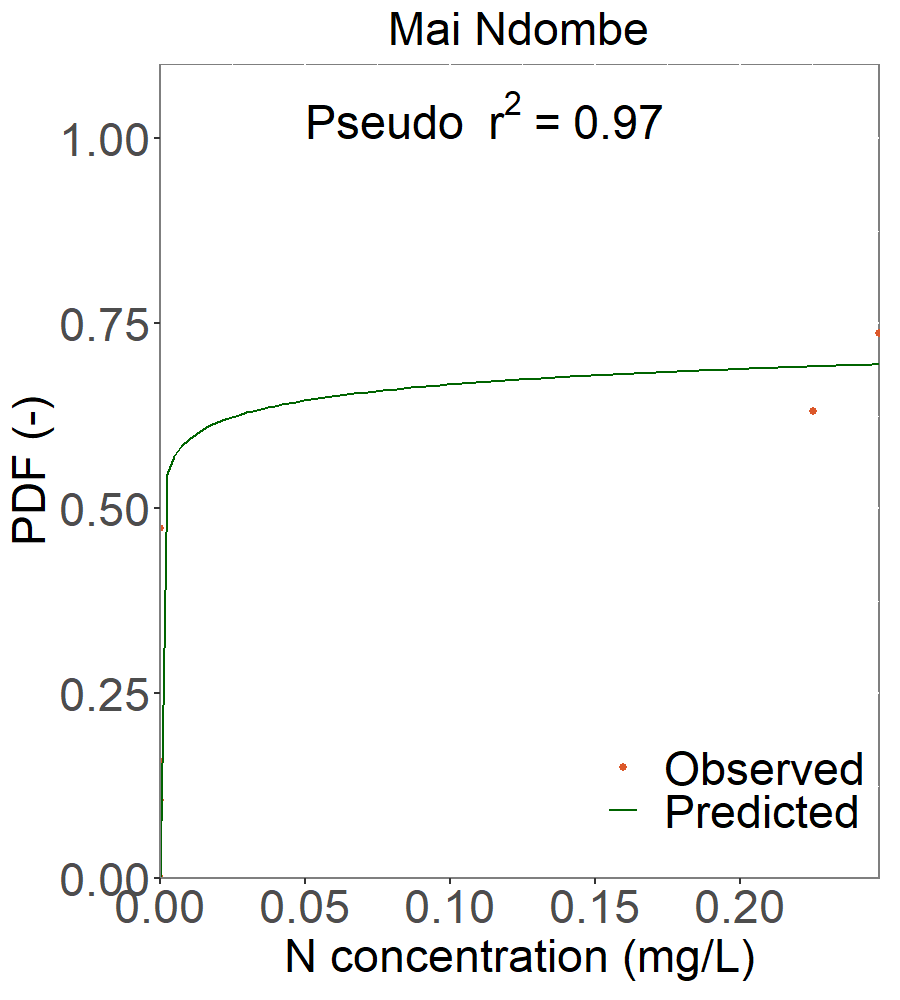

Supplement: Supplementary file 2 — es2c09333_si_002.zip [file es2c09333_si_002.zip › SSD_Ecoregion/Mai Ndombe.tif]

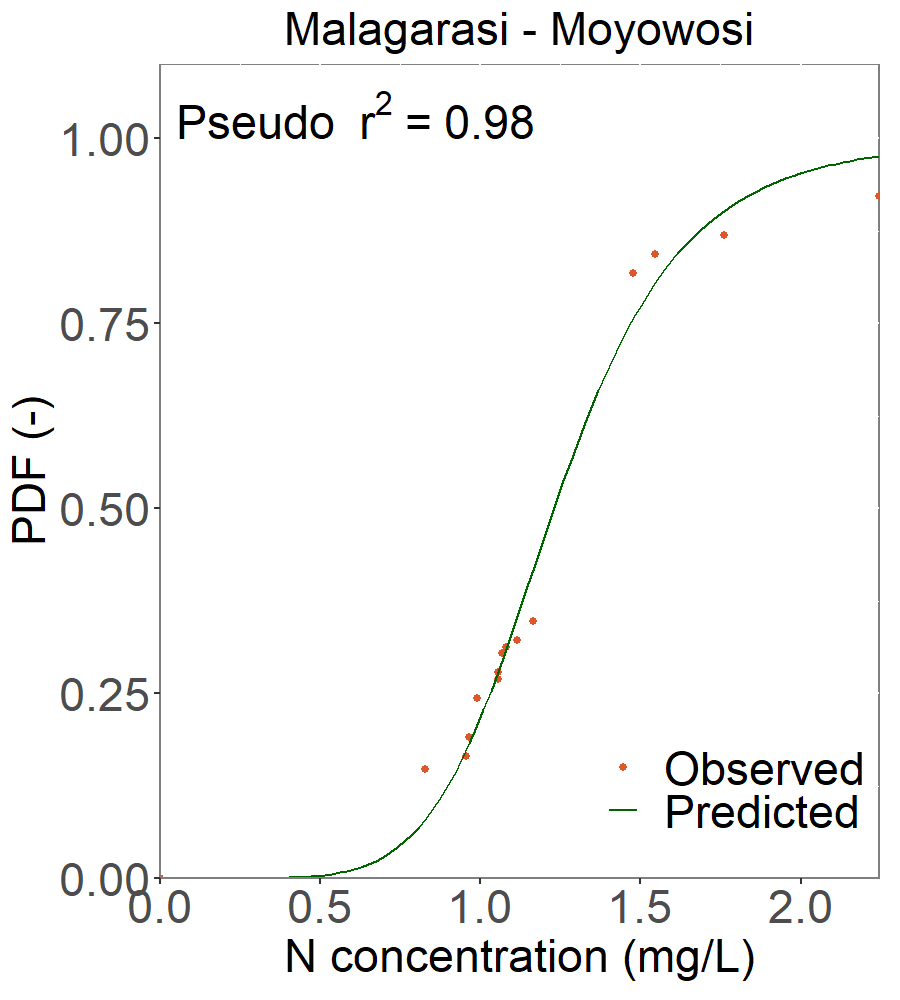

Supplement: Supplementary file 2 — es2c09333_si_002.zip [file es2c09333_si_002.zip › SSD_Ecoregion/Malagarasi - Moyowosi.tif]

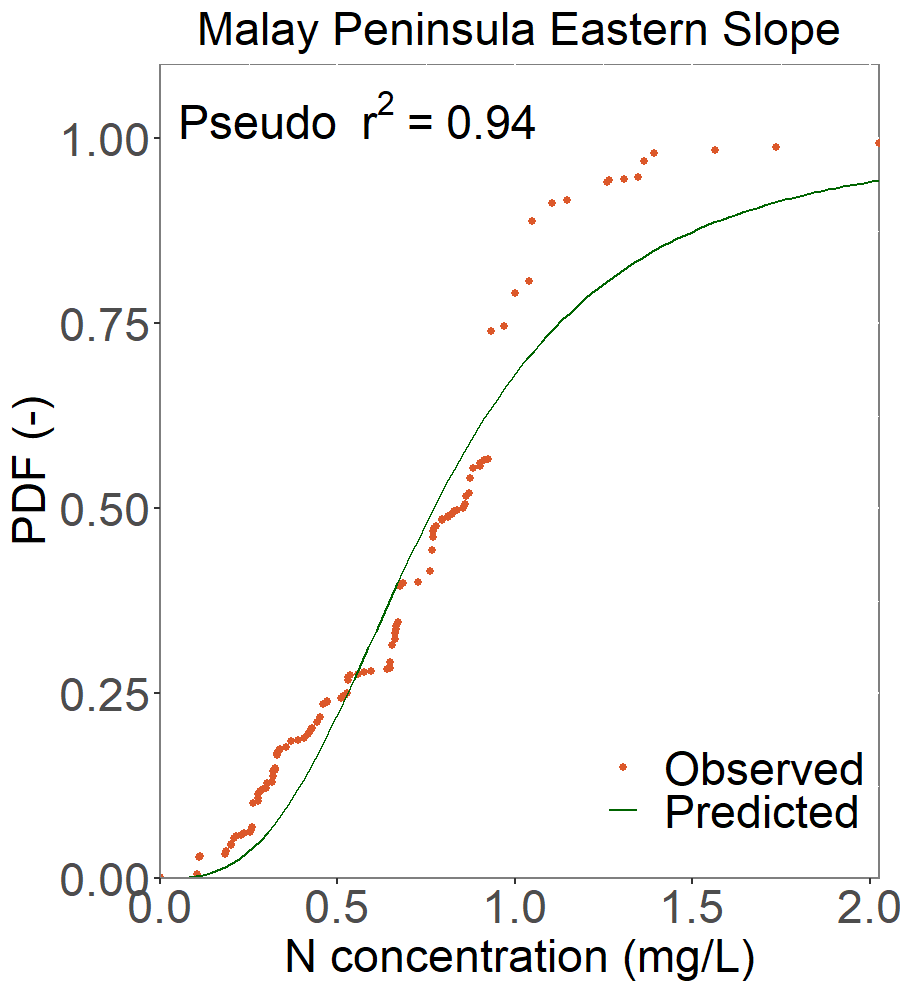

Supplement: Supplementary file 2 — es2c09333_si_002.zip [file es2c09333_si_002.zip › SSD_Ecoregion/Malay Peninsula Eastern Slope.tif]

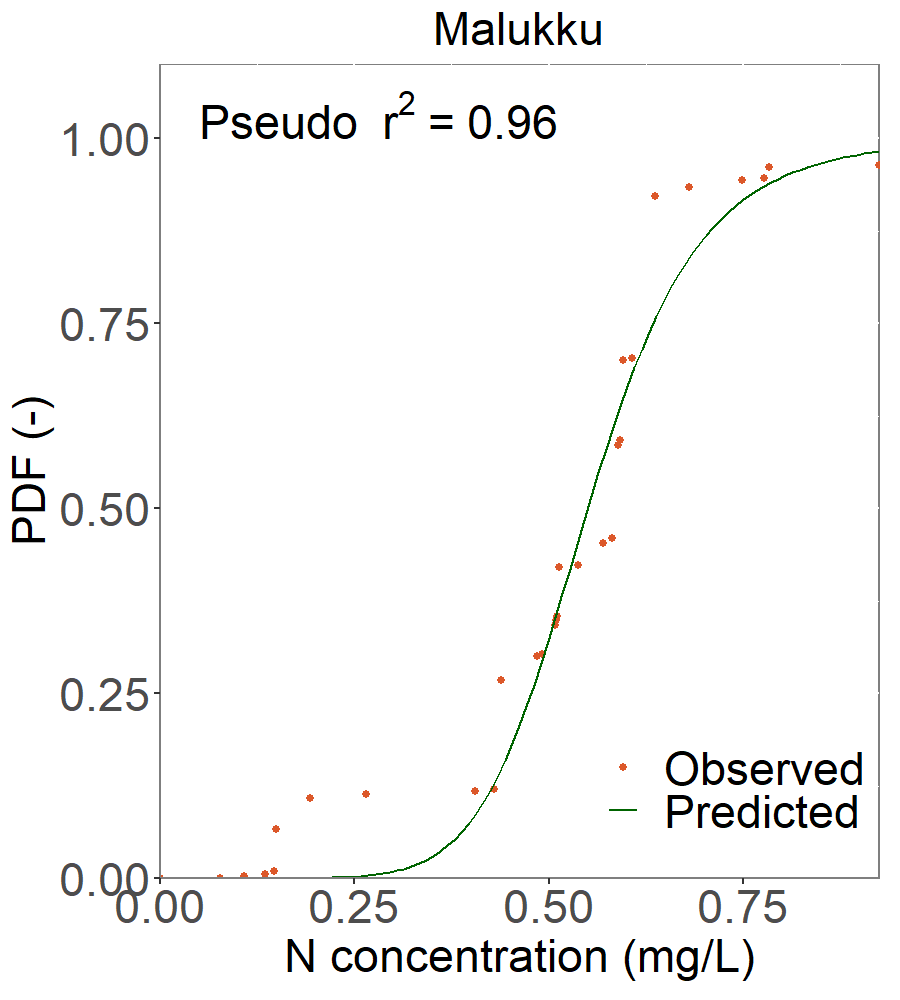

Supplement: Supplementary file 2 — es2c09333_si_002.zip [file es2c09333_si_002.zip › SSD_Ecoregion/Malukku.tif]

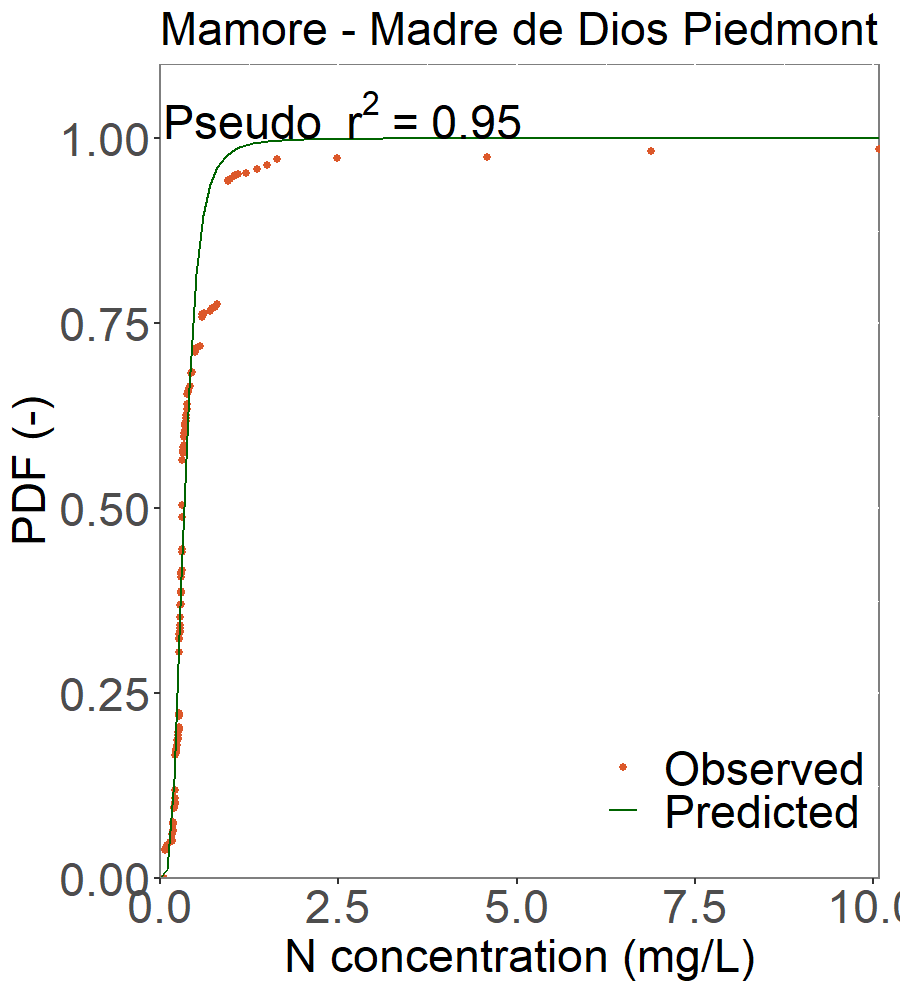

Supplement: Supplementary file 2 — es2c09333_si_002.zip [file es2c09333_si_002.zip › SSD_Ecoregion/Mamore - Madre de Dios Piedmont.tif]

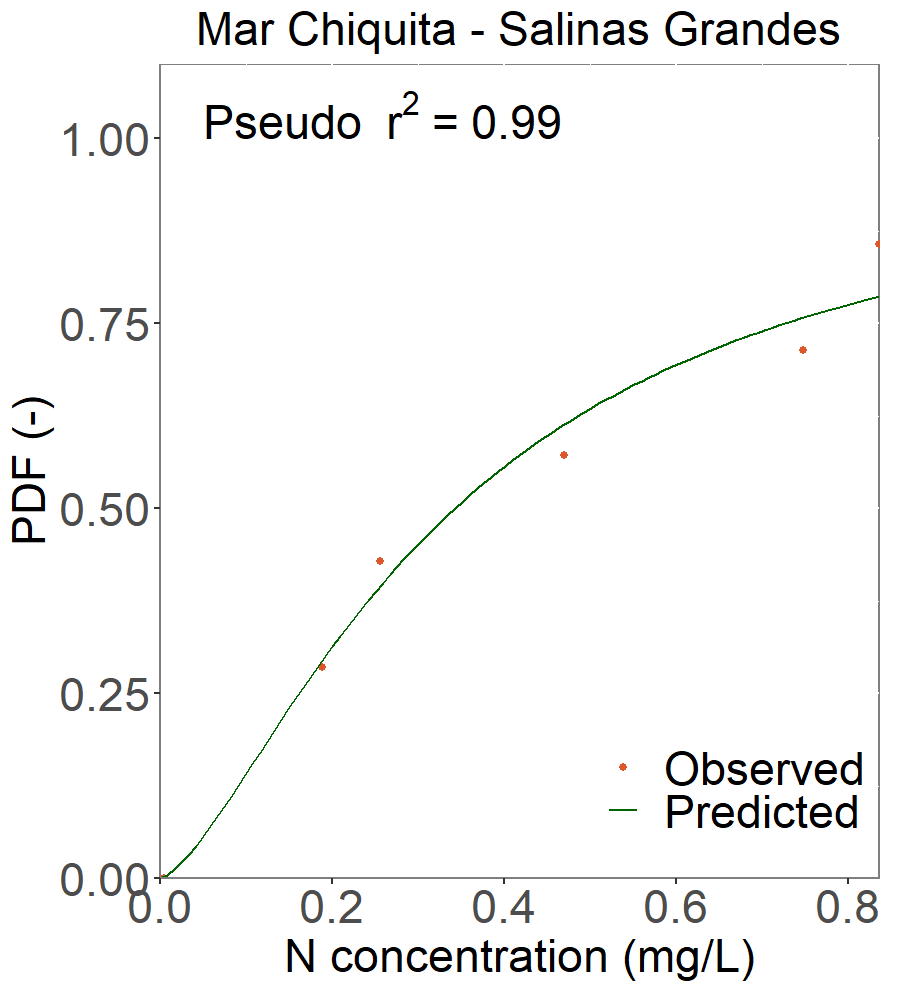

Supplement: Supplementary file 2 — es2c09333_si_002.zip [file es2c09333_si_002.zip › SSD_Ecoregion/Mar Chiquita - Salinas Grandes.tif]

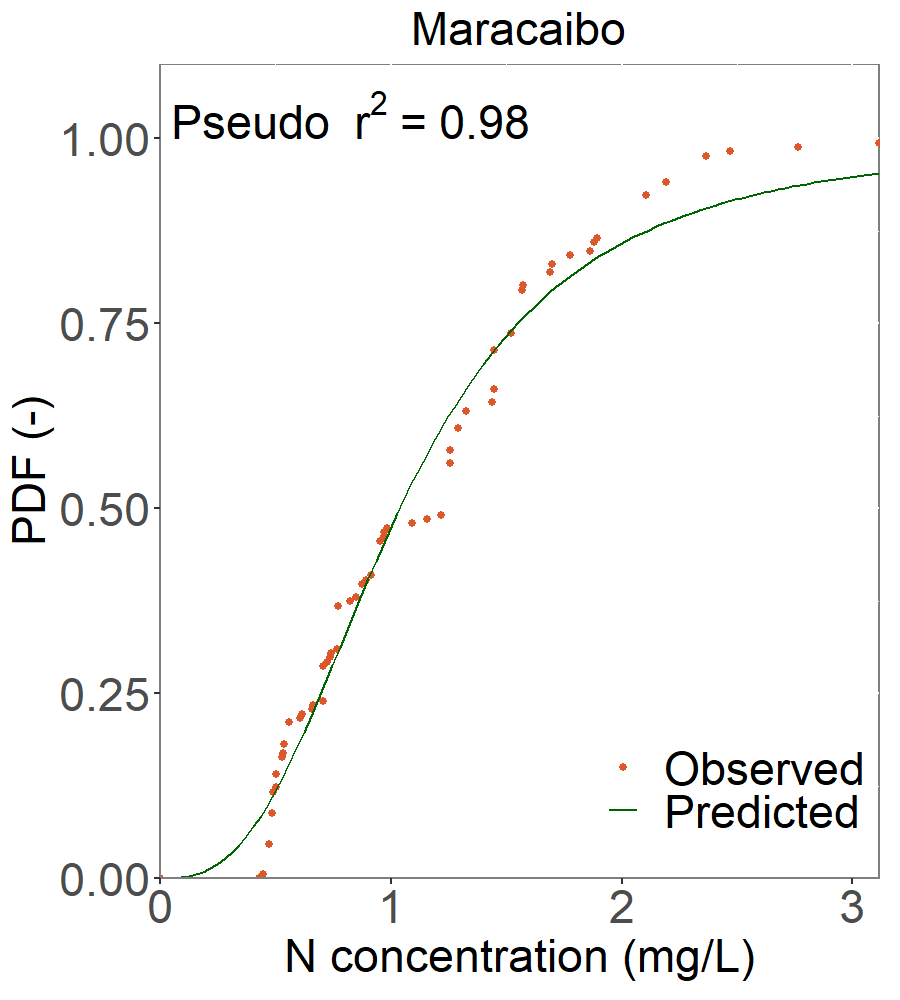

Supplement: Supplementary file 2 — es2c09333_si_002.zip [file es2c09333_si_002.zip › SSD_Ecoregion/Maracaibo.tif]

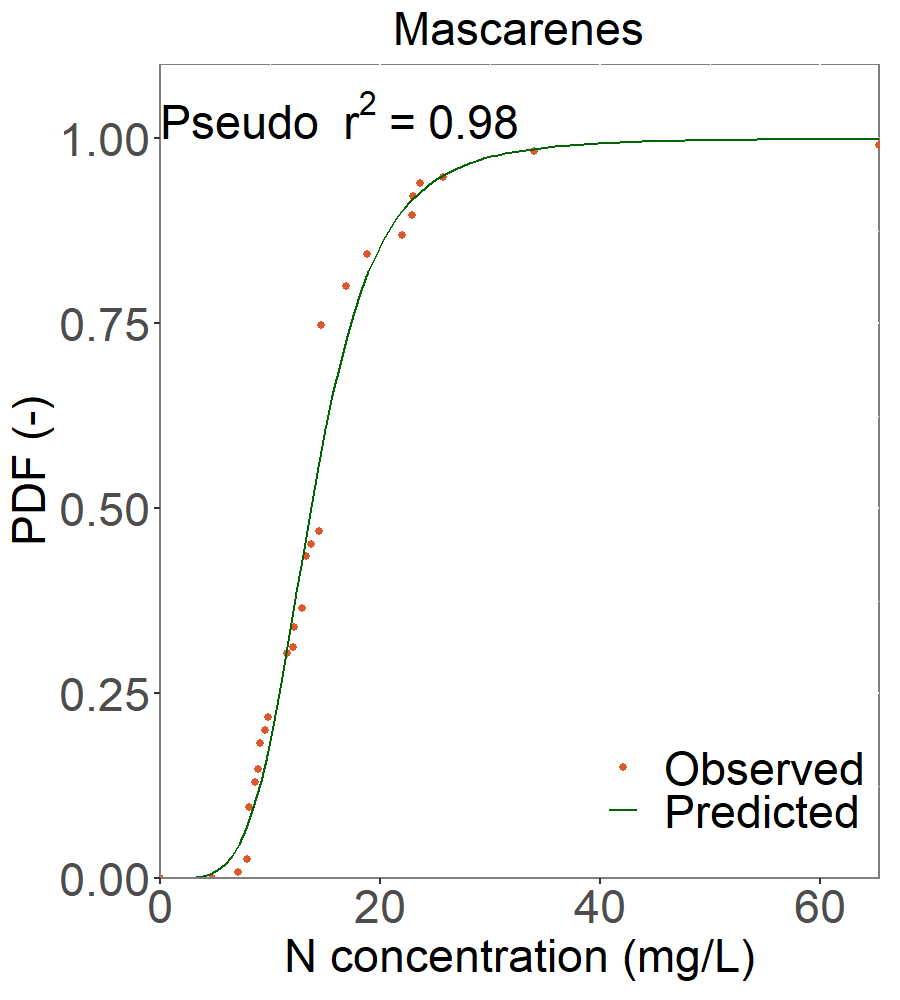

Supplement: Supplementary file 2 — es2c09333_si_002.zip [file es2c09333_si_002.zip › SSD_Ecoregion/Mascarenes.tif]

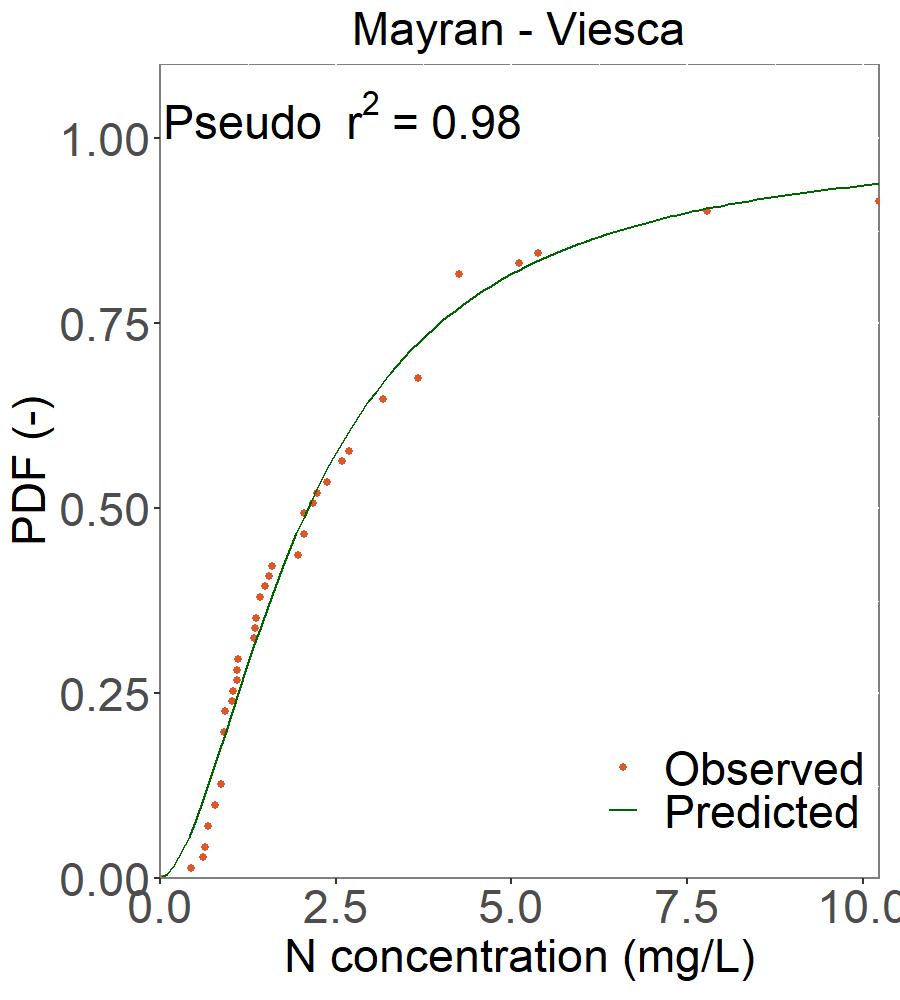

Supplement: Supplementary file 2 — es2c09333_si_002.zip [file es2c09333_si_002.zip › SSD_Ecoregion/Mayran - Viesca.tif]

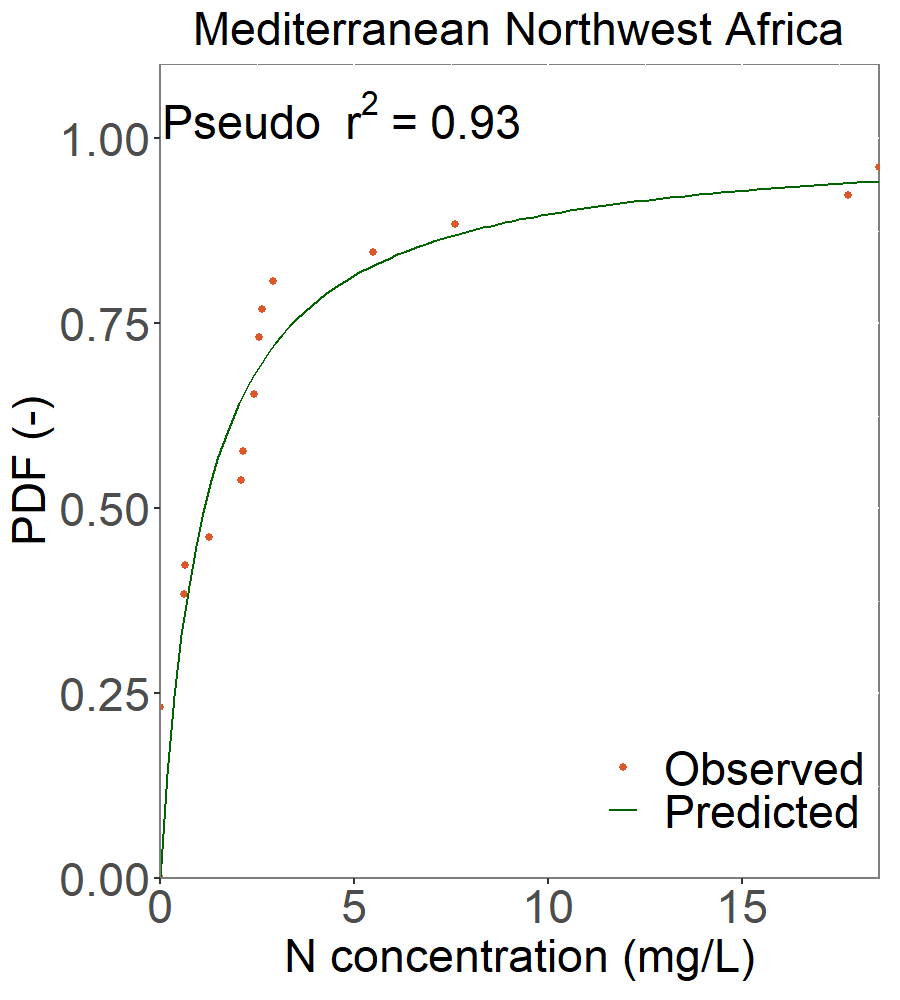

Supplement: Supplementary file 2 — es2c09333_si_002.zip [file es2c09333_si_002.zip › SSD_Ecoregion/Mediterranean Northwest Africa.tif]

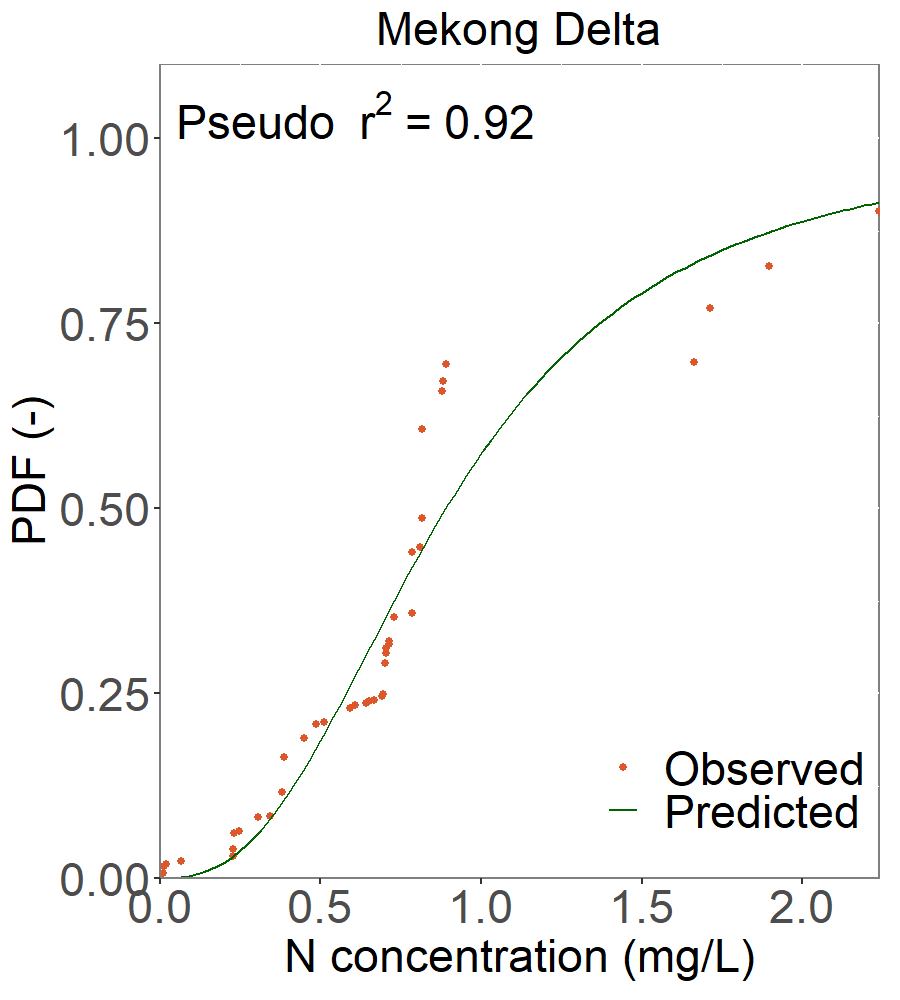

Supplement: Supplementary file 2 — es2c09333_si_002.zip [file es2c09333_si_002.zip › SSD_Ecoregion/Mekong Delta.tif]

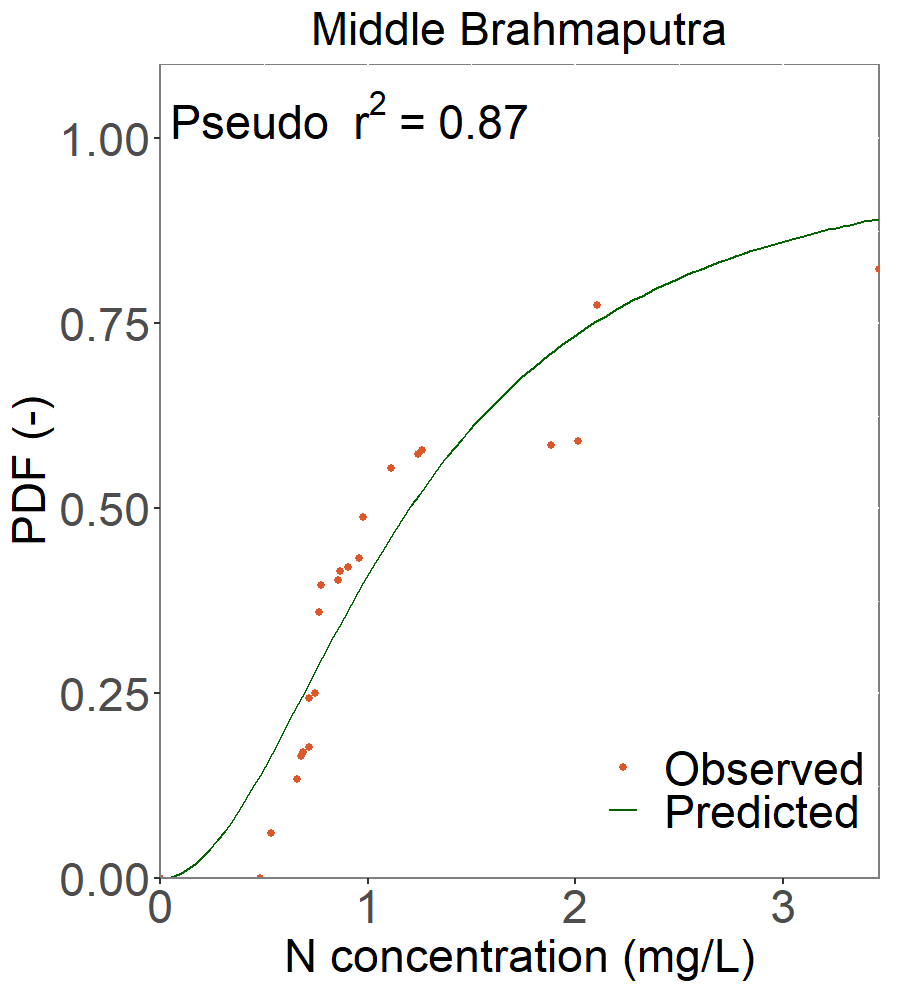

Supplement: Supplementary file 2 — es2c09333_si_002.zip [file es2c09333_si_002.zip › SSD_Ecoregion/Middle Brahmaputra.tif]

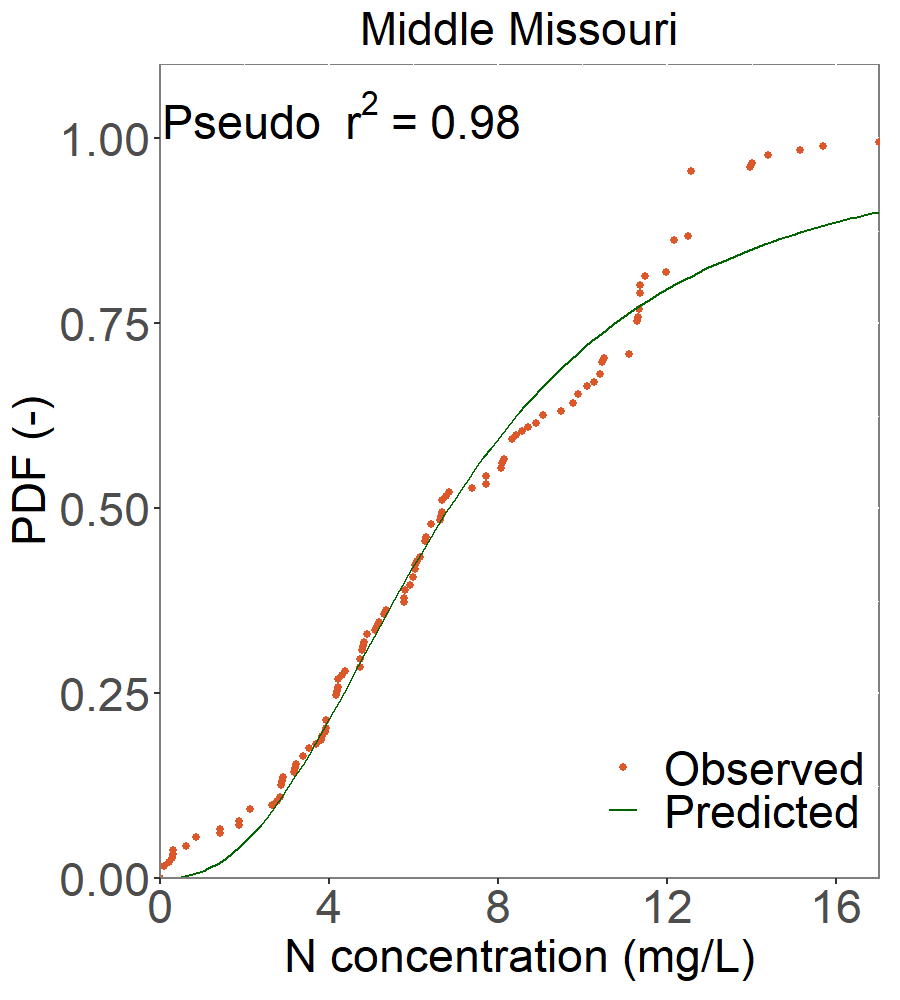

Supplement: Supplementary file 2 — es2c09333_si_002.zip [file es2c09333_si_002.zip › SSD_Ecoregion/Middle Missouri.tif]

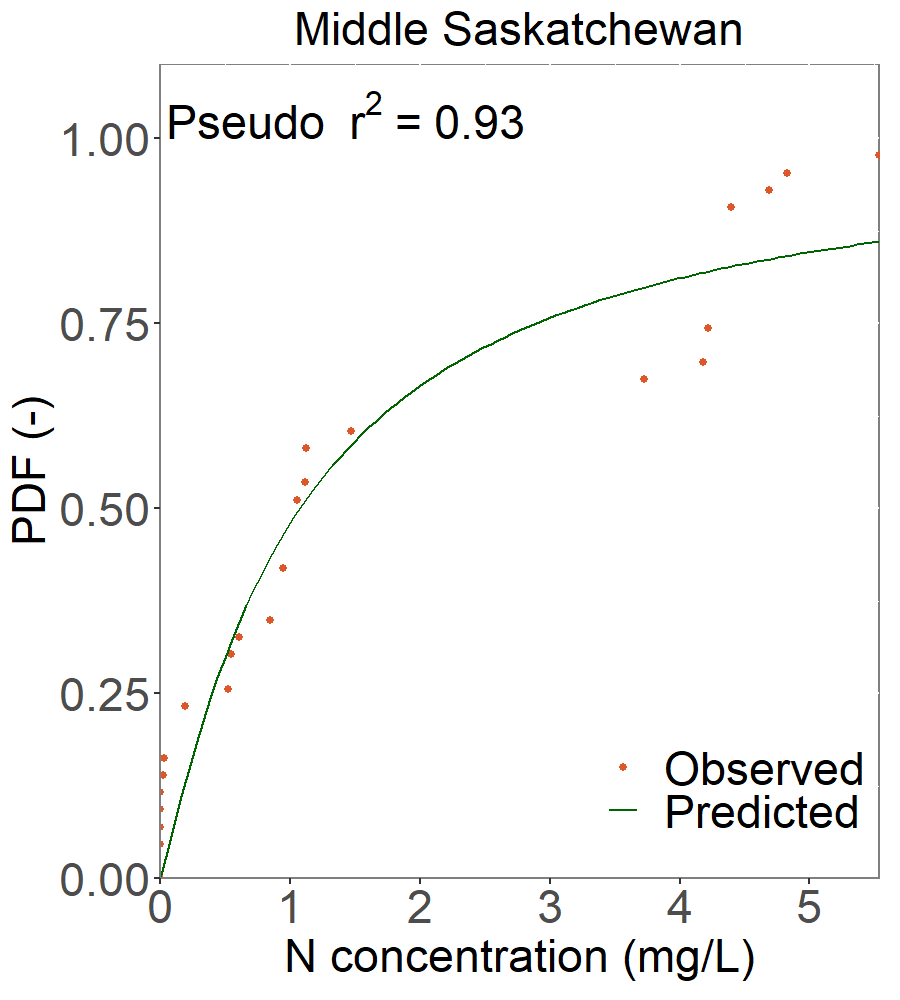

Supplement: Supplementary file 2 — es2c09333_si_002.zip [file es2c09333_si_002.zip › SSD_Ecoregion/Middle Saskatchewan.tif]

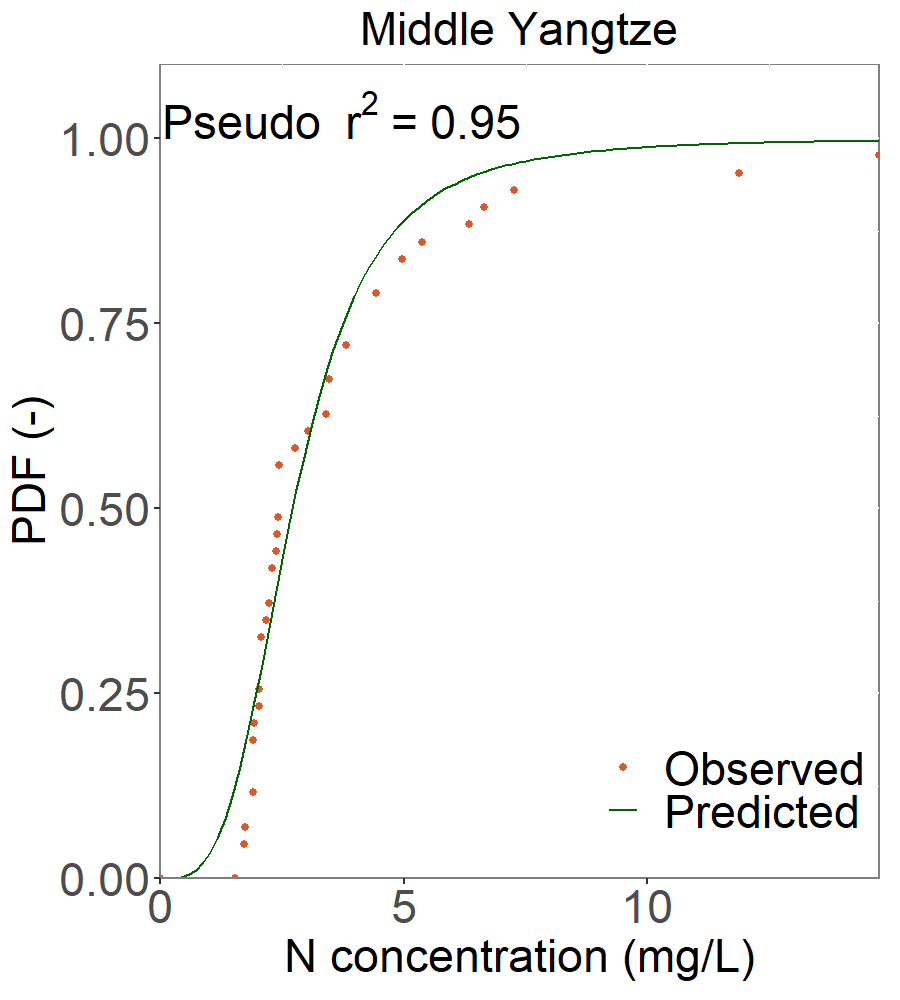

Supplement: Supplementary file 2 — es2c09333_si_002.zip [file es2c09333_si_002.zip › SSD_Ecoregion/Middle Yangtze.tif]

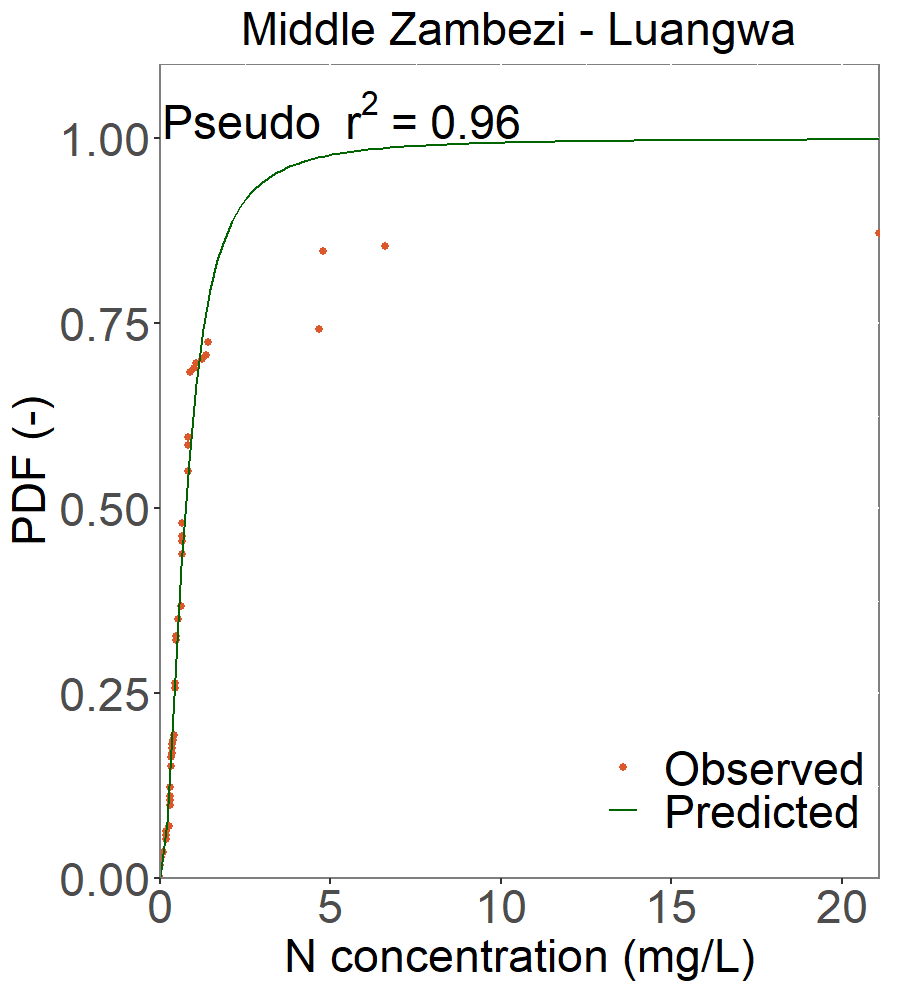

Supplement: Supplementary file 2 — es2c09333_si_002.zip [file es2c09333_si_002.zip › SSD_Ecoregion/Middle Zambezi - Luangwa.tif]

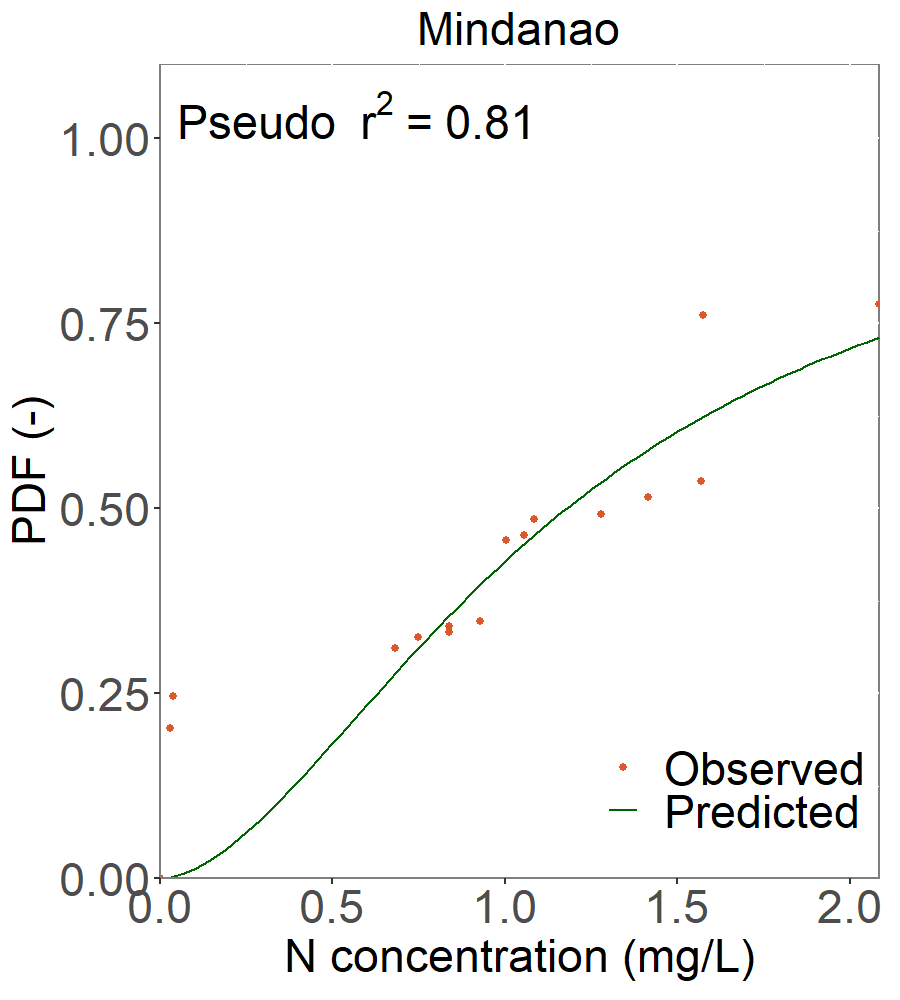

Supplement: Supplementary file 2 — es2c09333_si_002.zip [file es2c09333_si_002.zip › SSD_Ecoregion/Mindanao.tif]

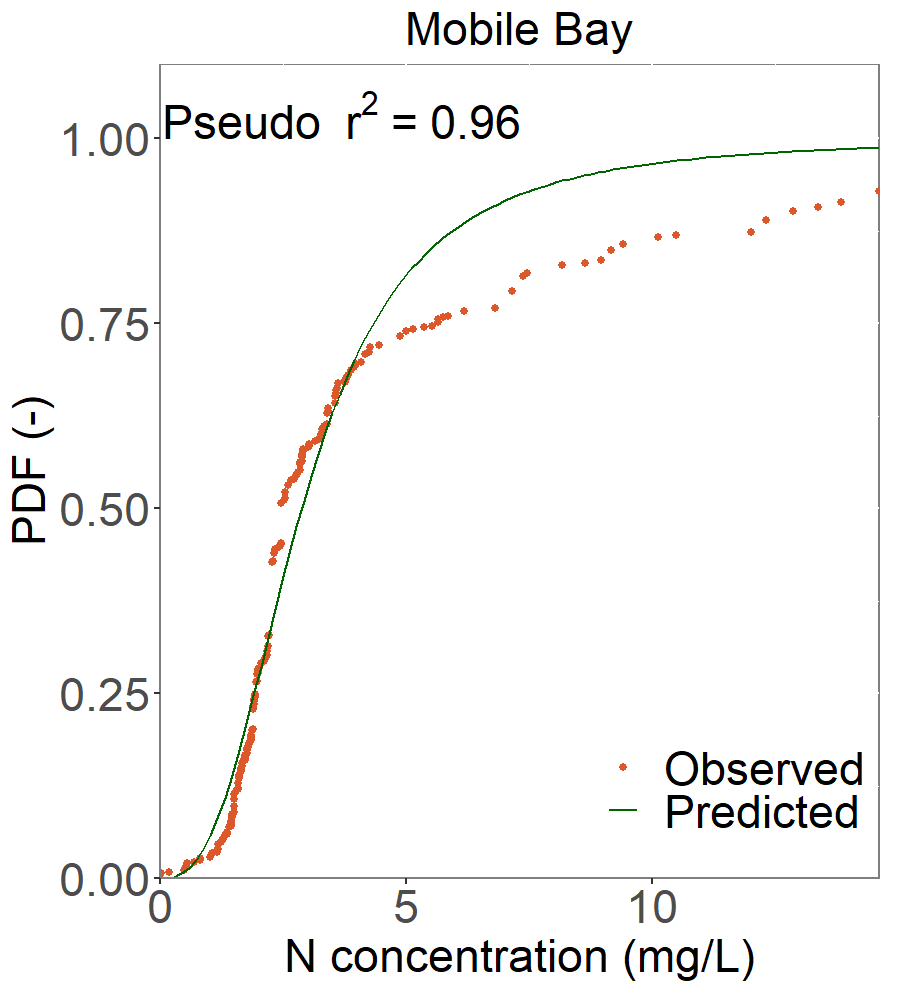

Supplement: Supplementary file 2 — es2c09333_si_002.zip [file es2c09333_si_002.zip › SSD_Ecoregion/Mobile Bay.tif]

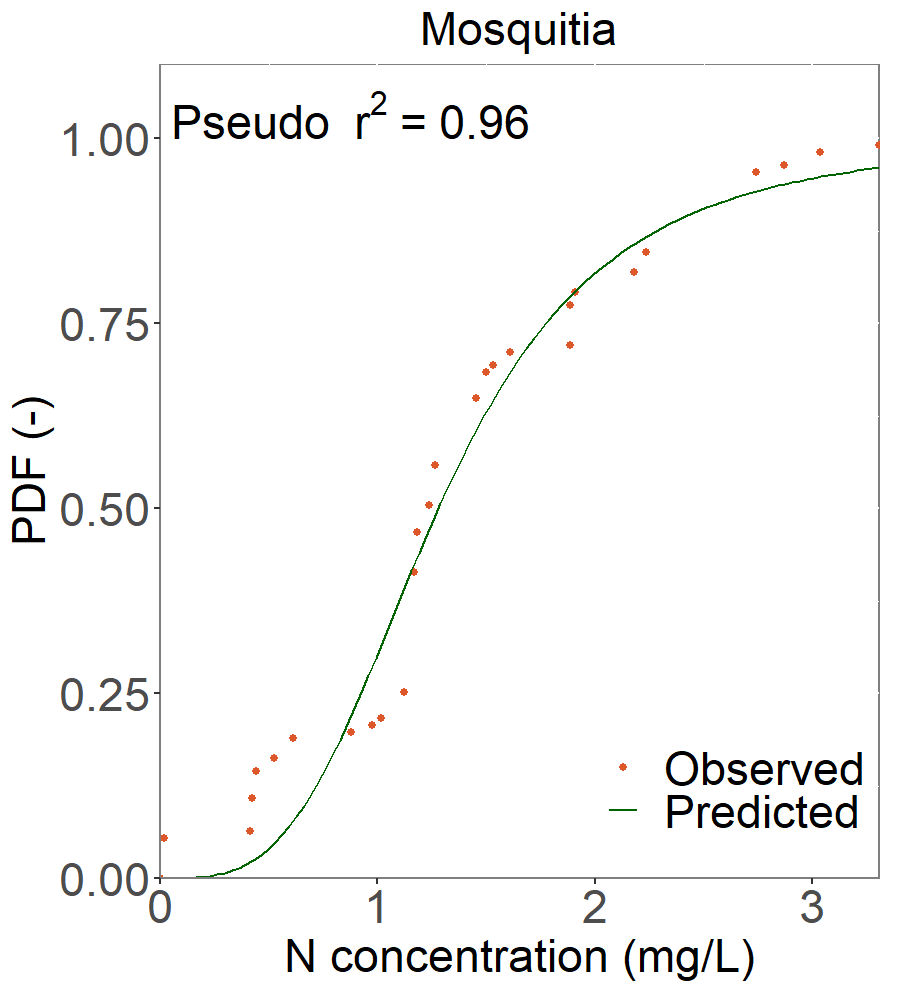

Supplement: Supplementary file 2 — es2c09333_si_002.zip [file es2c09333_si_002.zip › SSD_Ecoregion/Mosquitia.tif]

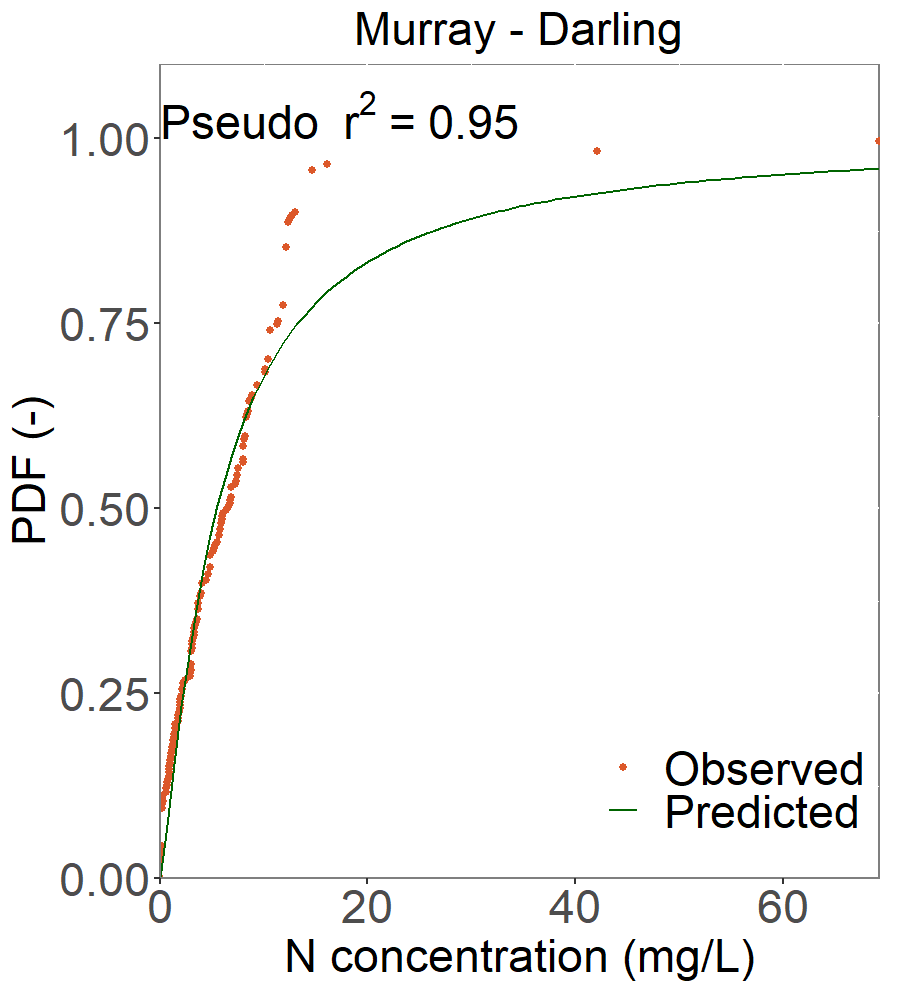

Supplement: Supplementary file 2 — es2c09333_si_002.zip [file es2c09333_si_002.zip › SSD_Ecoregion/Murray - Darling.tif]

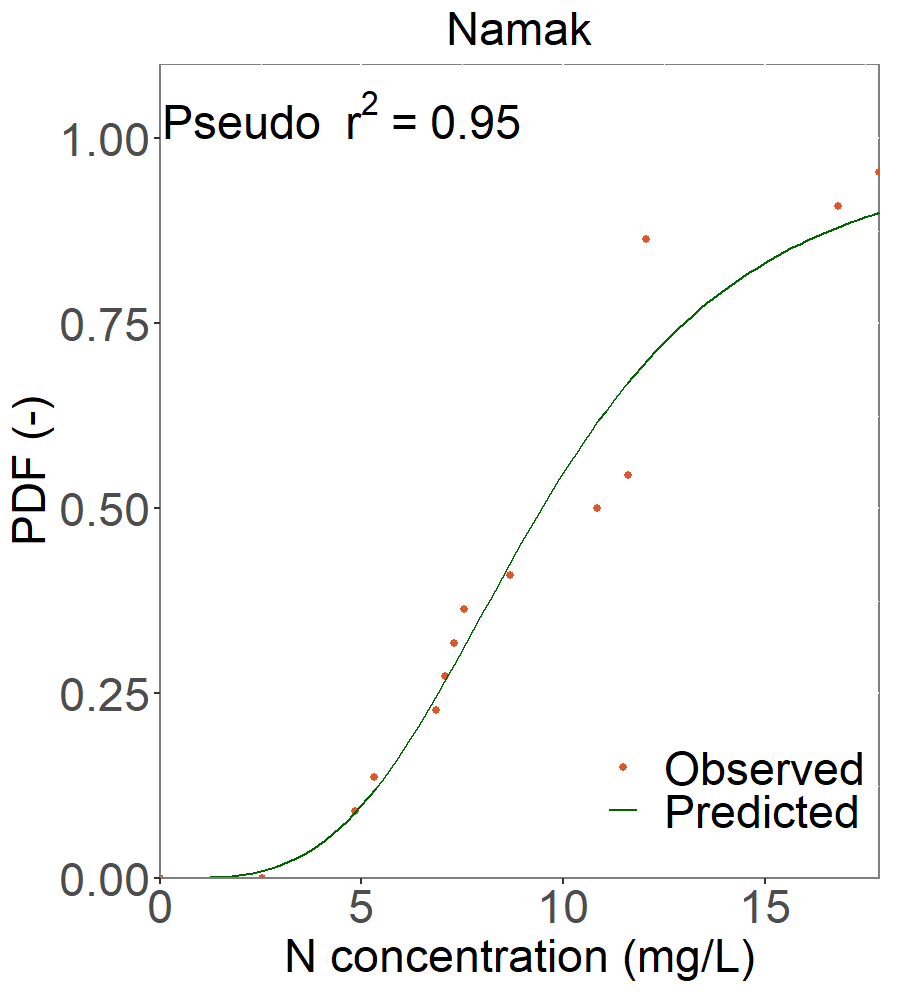

Supplement: Supplementary file 2 — es2c09333_si_002.zip [file es2c09333_si_002.zip › SSD_Ecoregion/Namak.tif]

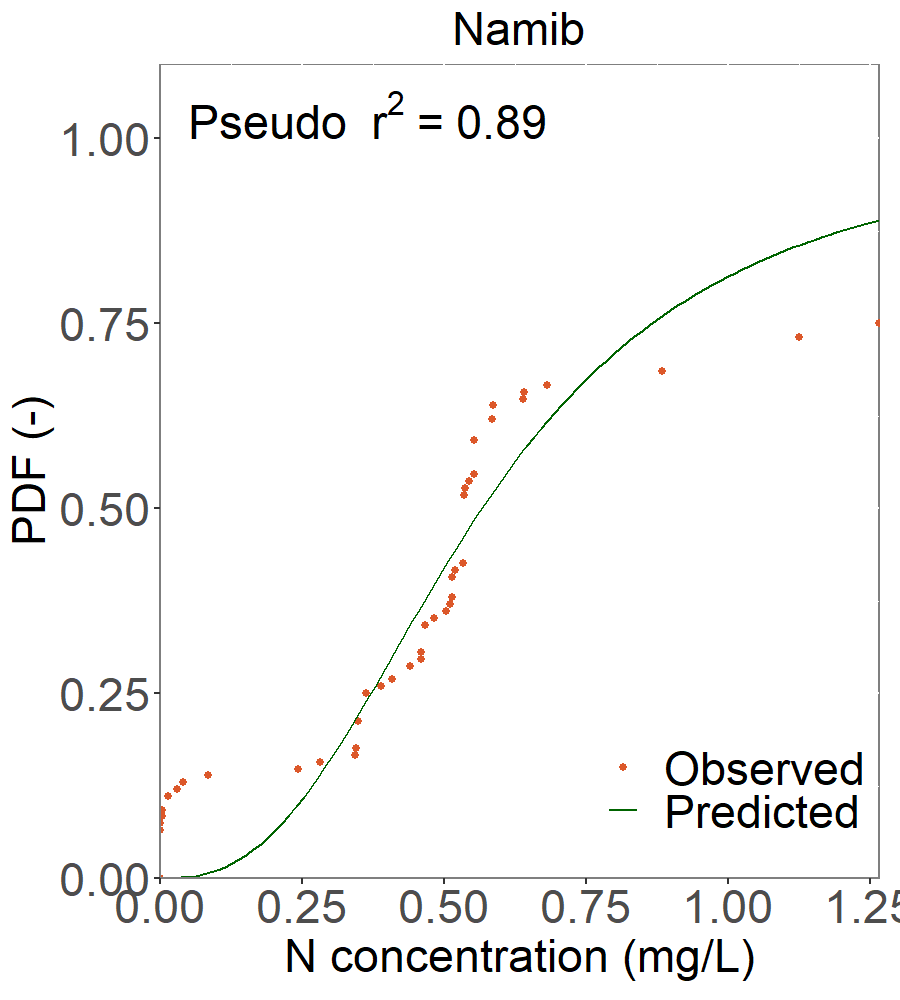

Supplement: Supplementary file 2 — es2c09333_si_002.zip [file es2c09333_si_002.zip › SSD_Ecoregion/Namib.tif]

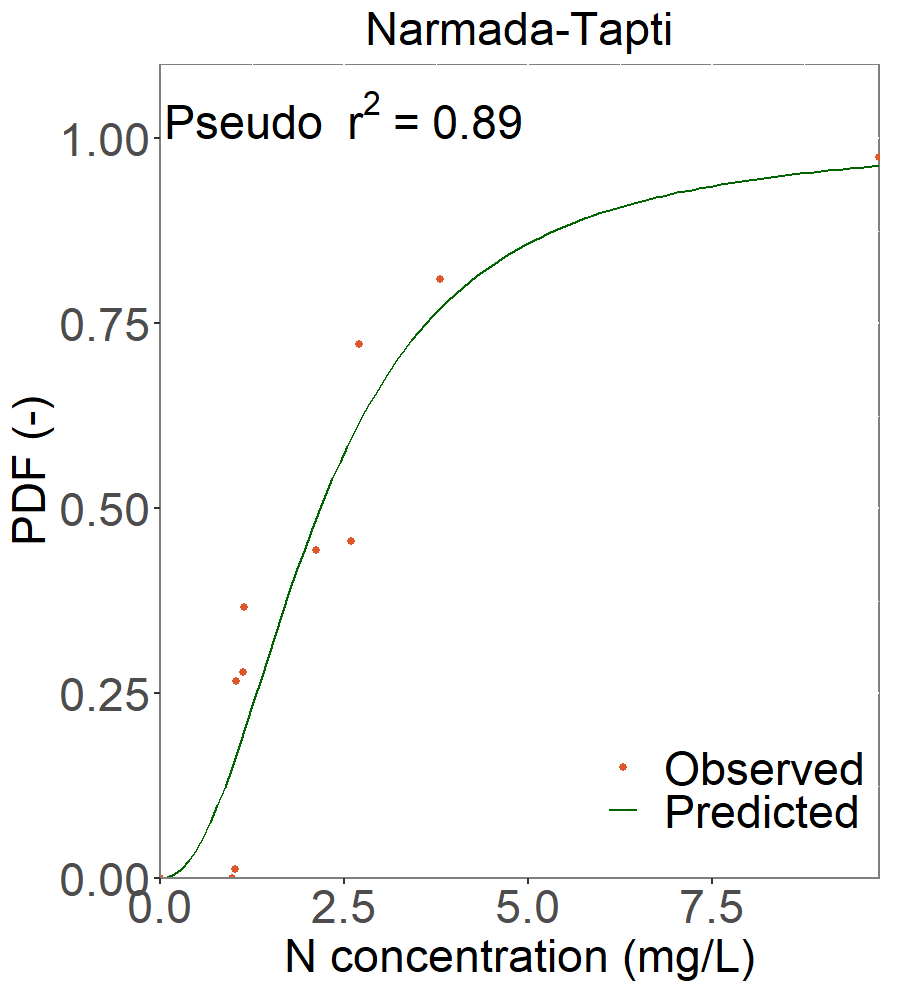

Supplement: Supplementary file 2 — es2c09333_si_002.zip [file es2c09333_si_002.zip › SSD_Ecoregion/Narmada-Tapti.tif]

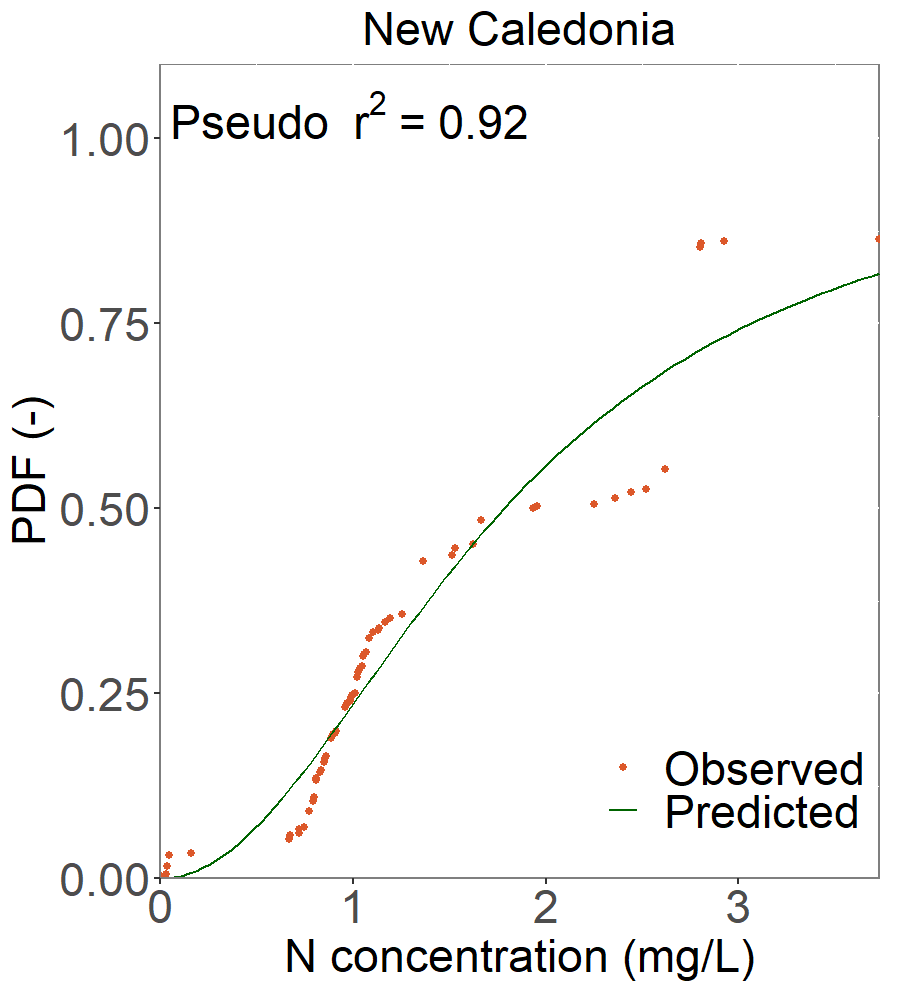

Supplement: Supplementary file 2 — es2c09333_si_002.zip [file es2c09333_si_002.zip › SSD_Ecoregion/New Caledonia.tif]

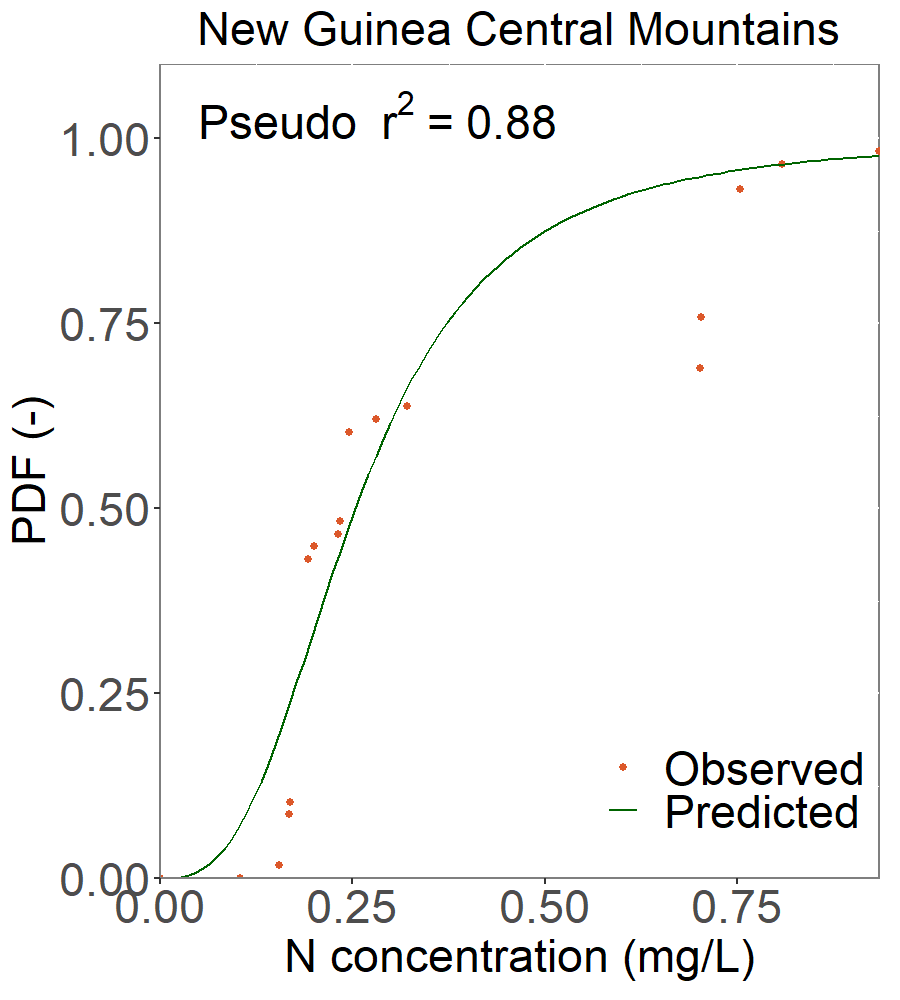

Supplement: Supplementary file 2 — es2c09333_si_002.zip [file es2c09333_si_002.zip › SSD_Ecoregion/New Guinea Central Mountains.tif]

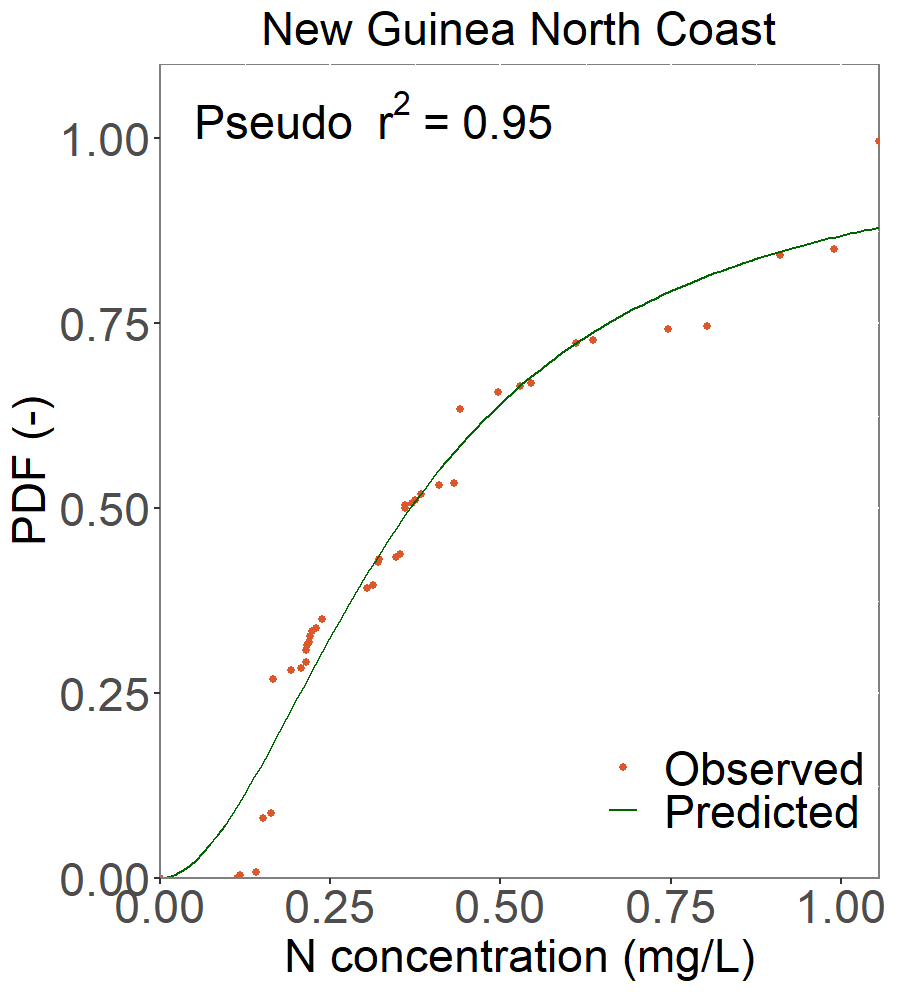

Supplement: Supplementary file 2 — es2c09333_si_002.zip [file es2c09333_si_002.zip › SSD_Ecoregion/New Guinea North Coast.tif]

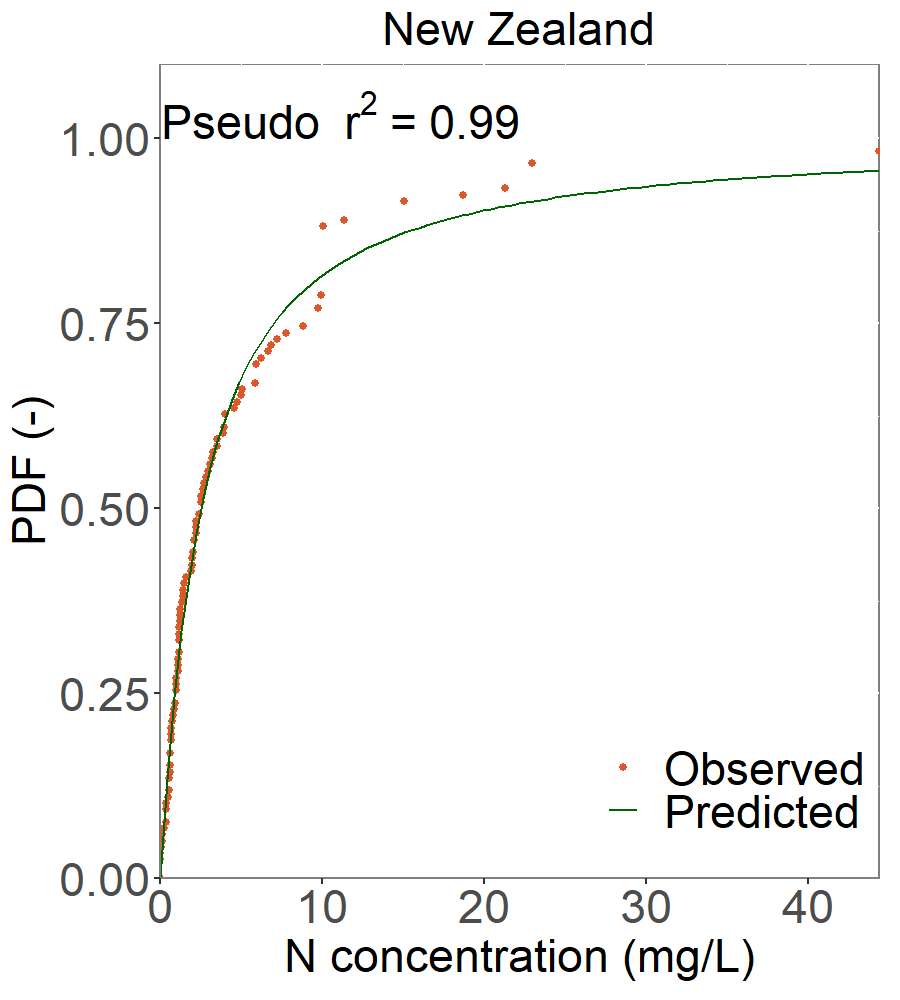

Supplement: Supplementary file 2 — es2c09333_si_002.zip [file es2c09333_si_002.zip › SSD_Ecoregion/New Zealand.tif]

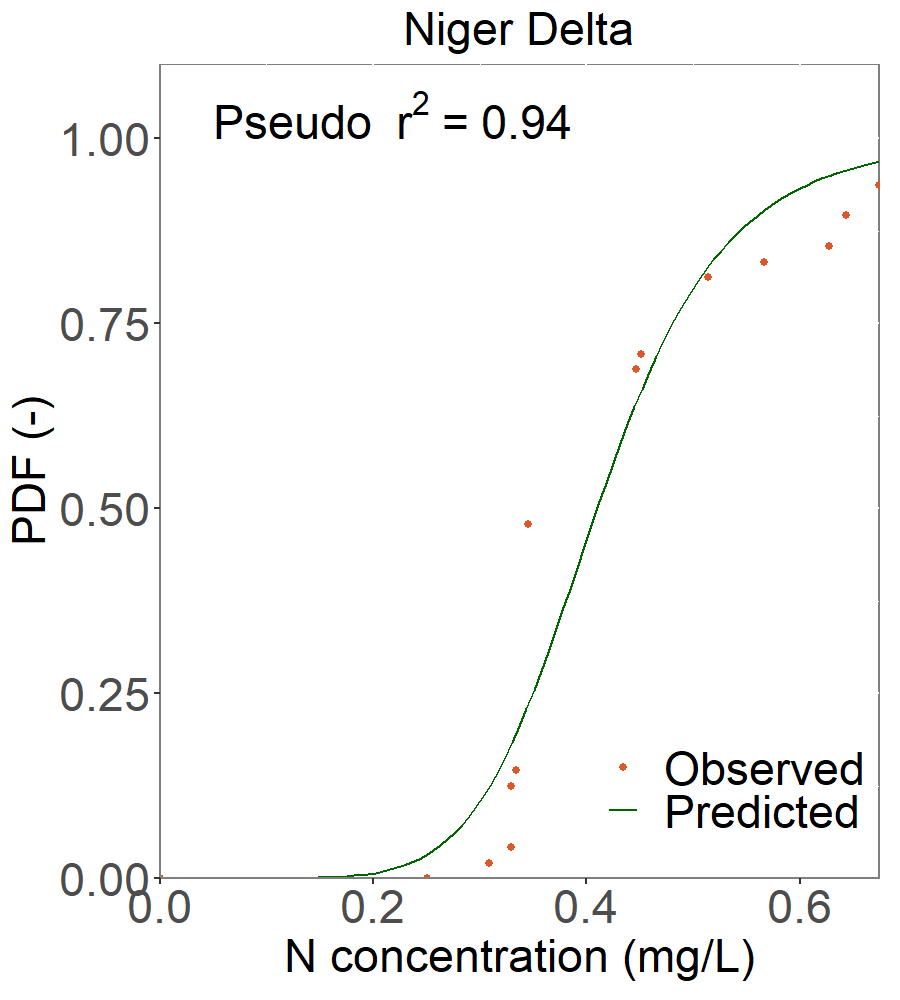

Supplement: Supplementary file 2 — es2c09333_si_002.zip [file es2c09333_si_002.zip › SSD_Ecoregion/Niger Delta.tif]

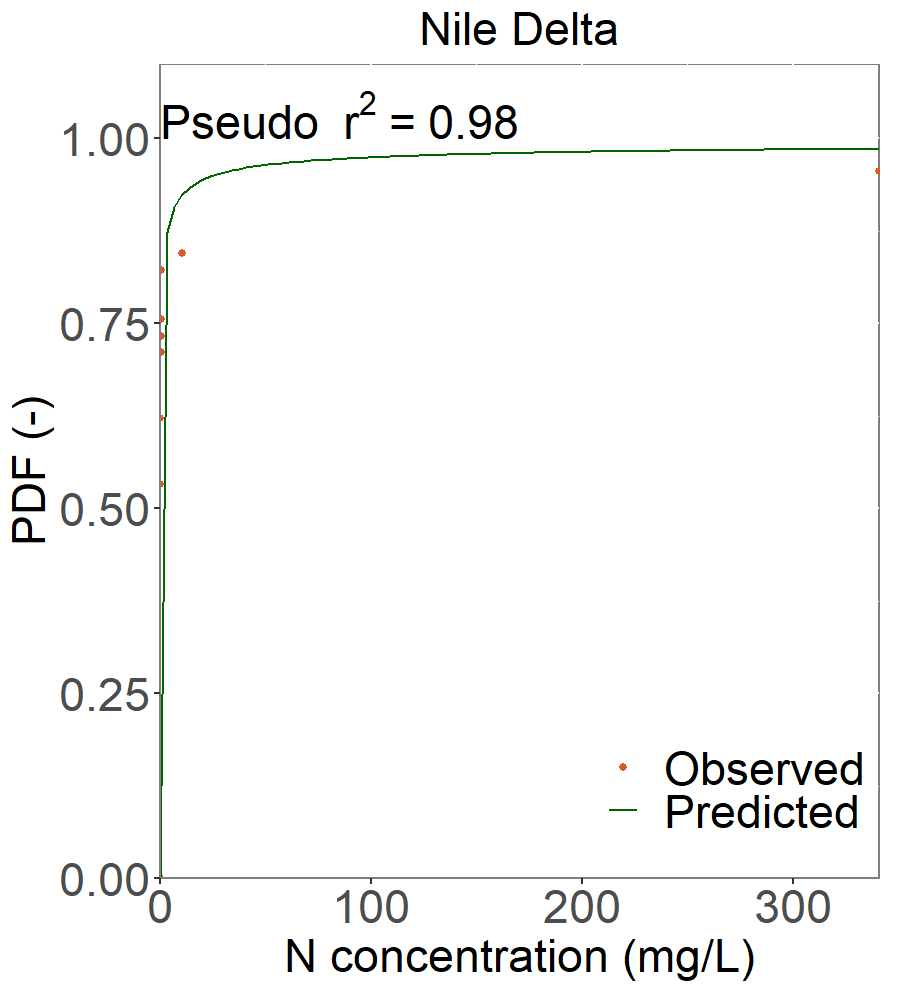

Supplement: Supplementary file 2 — es2c09333_si_002.zip [file es2c09333_si_002.zip › SSD_Ecoregion/Nile Delta.tif]

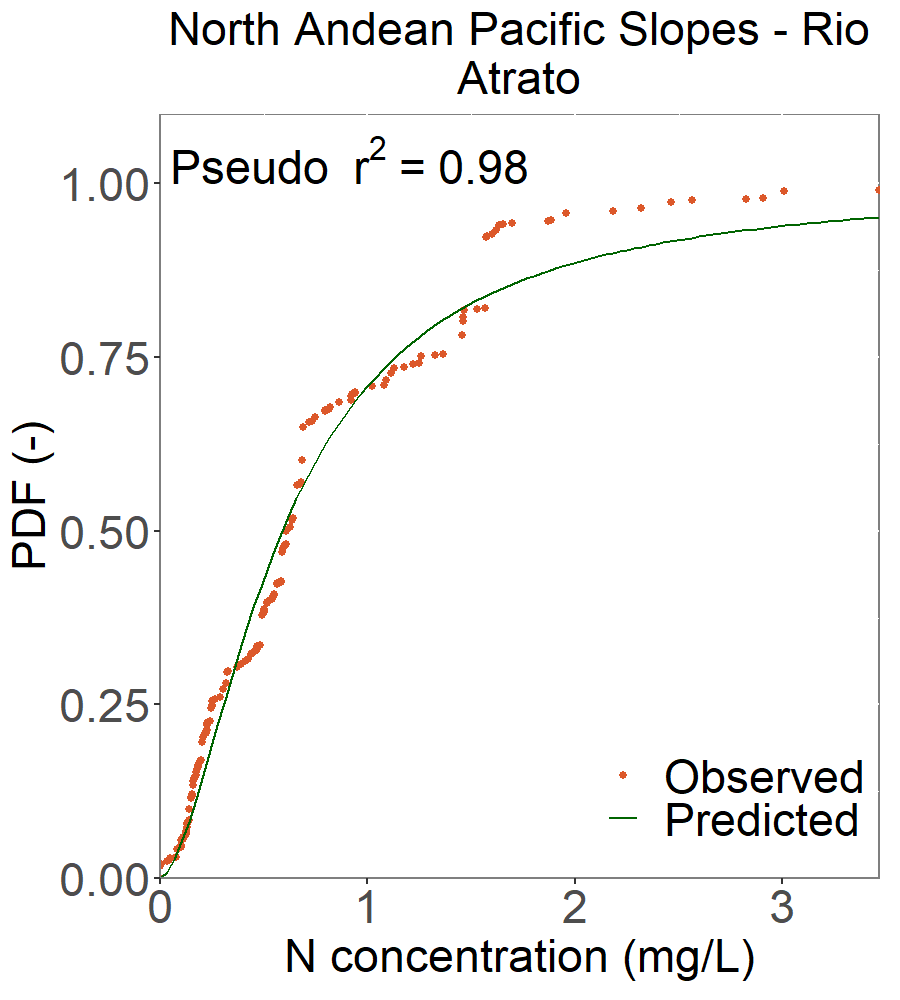

Supplement: Supplementary file 2 — es2c09333_si_002.zip [file es2c09333_si_002.zip › SSD_Ecoregion/North Andean Pacific Slopes - Rio Atrato.tif]

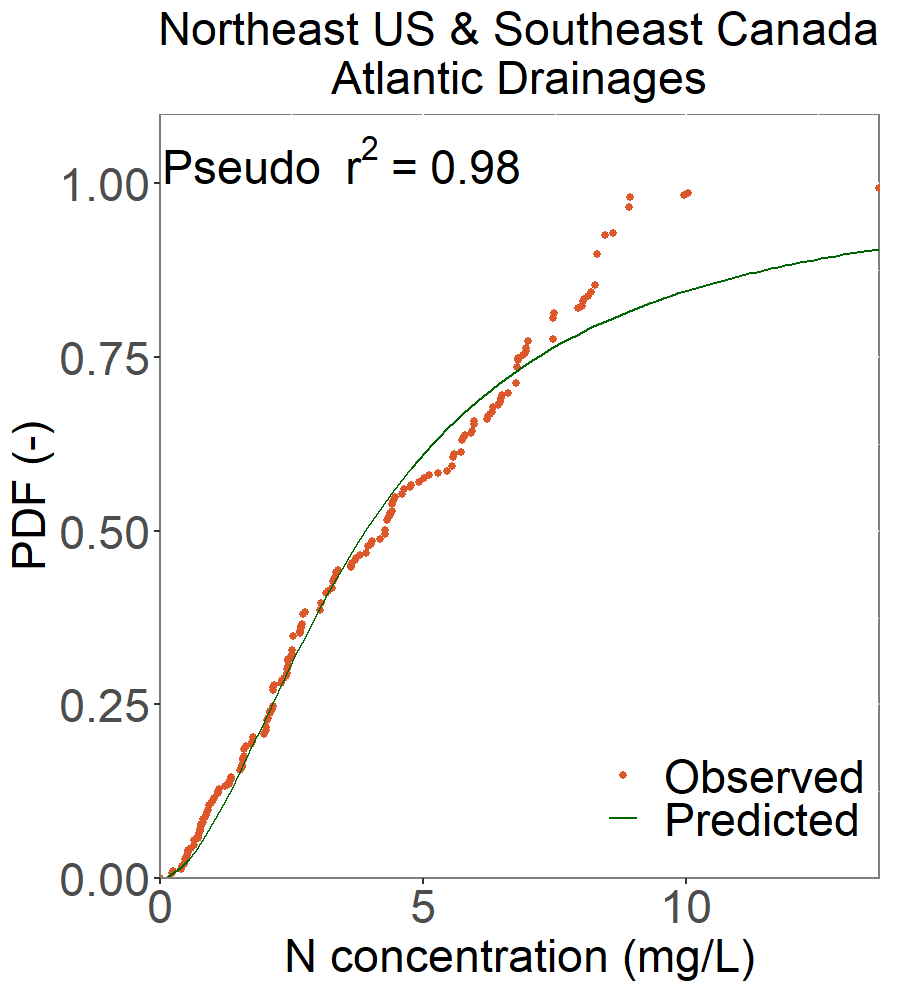

Supplement: Supplementary file 2 — es2c09333_si_002.zip [file es2c09333_si_002.zip › SSD_Ecoregion/Northeast US & Southeast Canada Atlantic Drainages.tif]

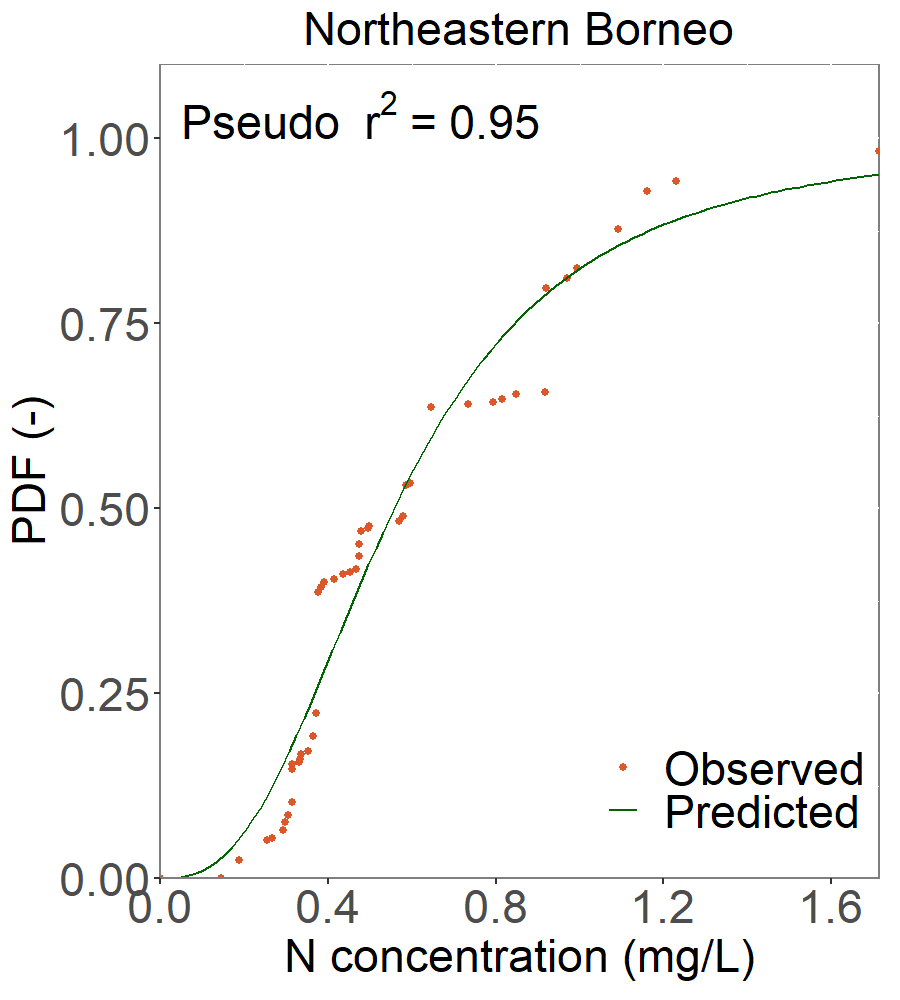

Supplement: Supplementary file 2 — es2c09333_si_002.zip [file es2c09333_si_002.zip › SSD_Ecoregion/Northeastern Borneo.tif]

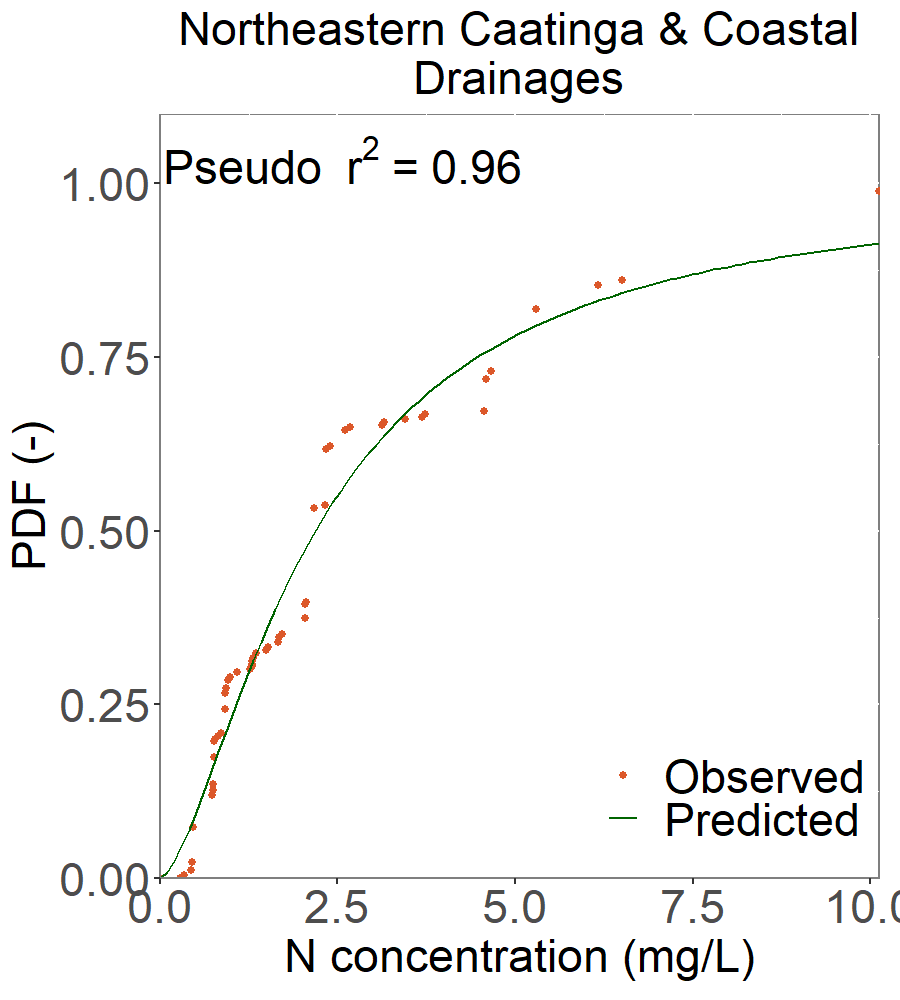

Supplement: Supplementary file 2 — es2c09333_si_002.zip [file es2c09333_si_002.zip › SSD_Ecoregion/Northeastern Caatinga & Coastal Drainages.tif]

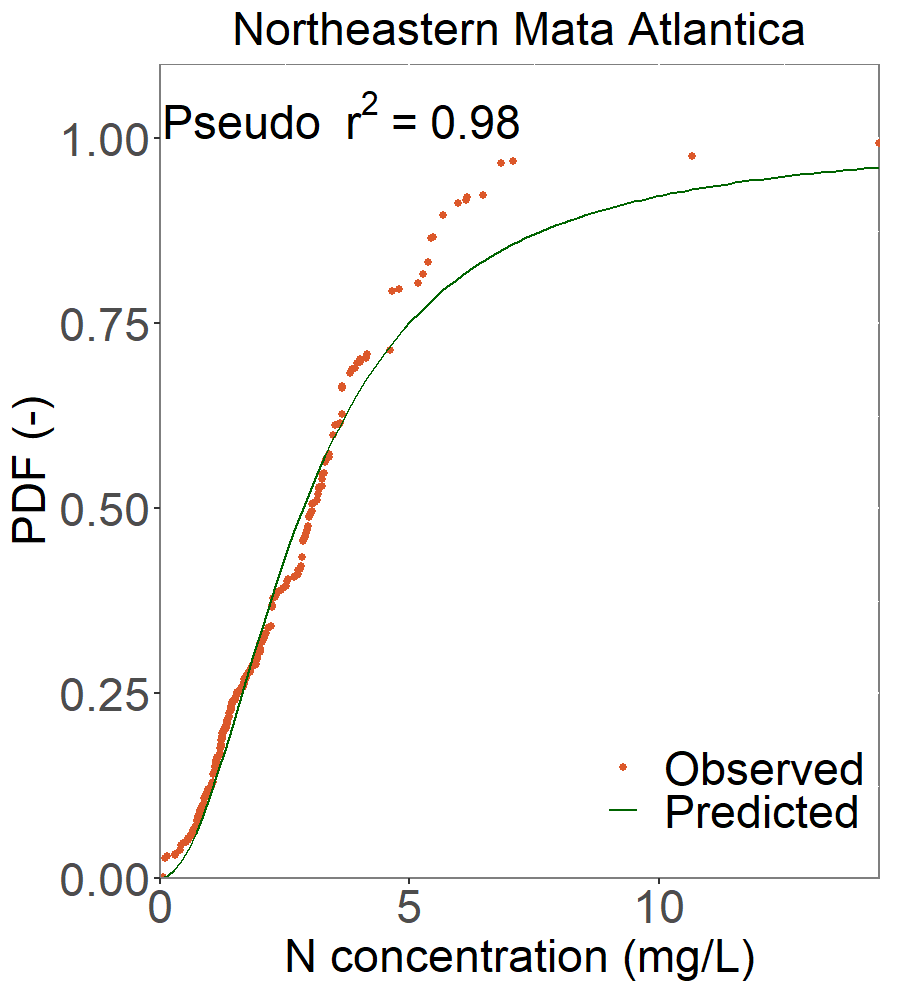

Supplement: Supplementary file 2 — es2c09333_si_002.zip [file es2c09333_si_002.zip › SSD_Ecoregion/Northeastern Mata Atlantica.tif]

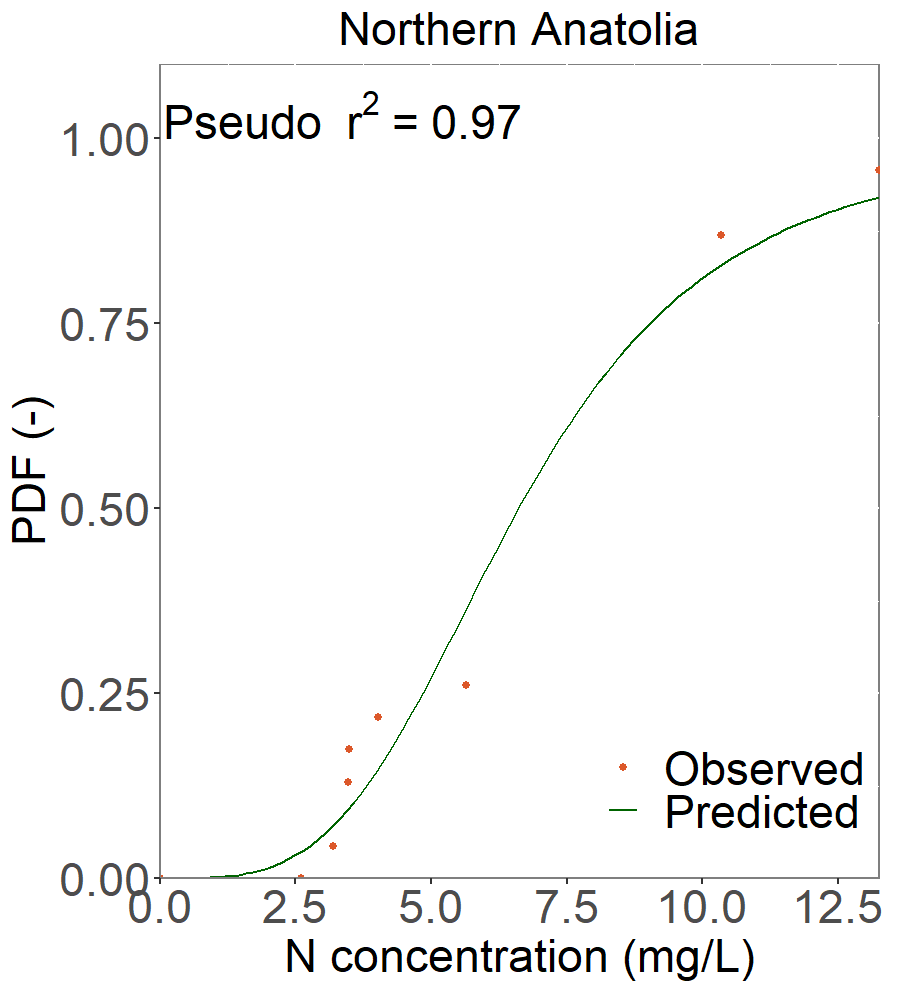

Supplement: Supplementary file 2 — es2c09333_si_002.zip [file es2c09333_si_002.zip › SSD_Ecoregion/Northern Anatolia.tif]

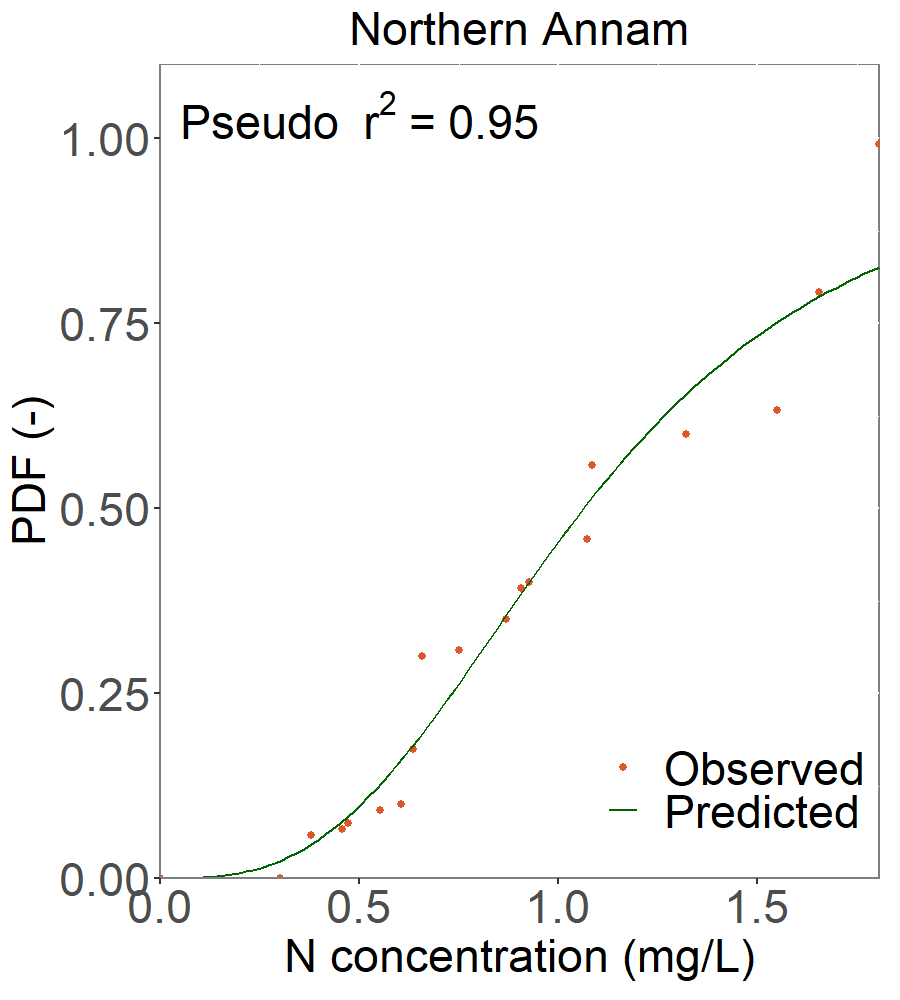

Supplement: Supplementary file 2 — es2c09333_si_002.zip [file es2c09333_si_002.zip › SSD_Ecoregion/Northern Annam.tif]
