# Supplementary figures and images for: Effects of Nitrogen Emissions on Fish Species Richness across the World’s Freshwater Ecoregions (part 1 of 3)
Source: Environ Sci Technol. 2023 May 22;57(22):8347–54. doi: 10.1021/acs.est.2c09333 (PMC10249400; doi:10.1021/acs.est.2c09333)

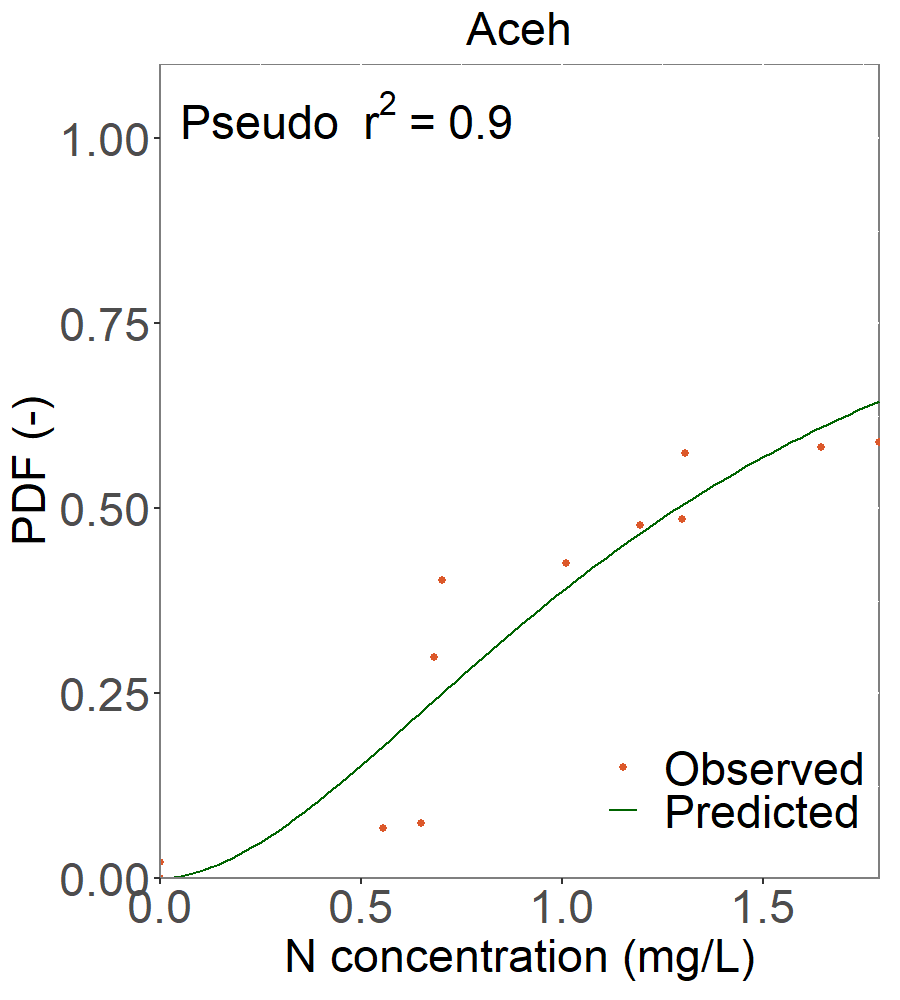

Supplement: Supplementary file 2 — es2c09333_si_002.zip [file es2c09333_si_002.zip › SSD_Ecoregion/Aceh.tif]

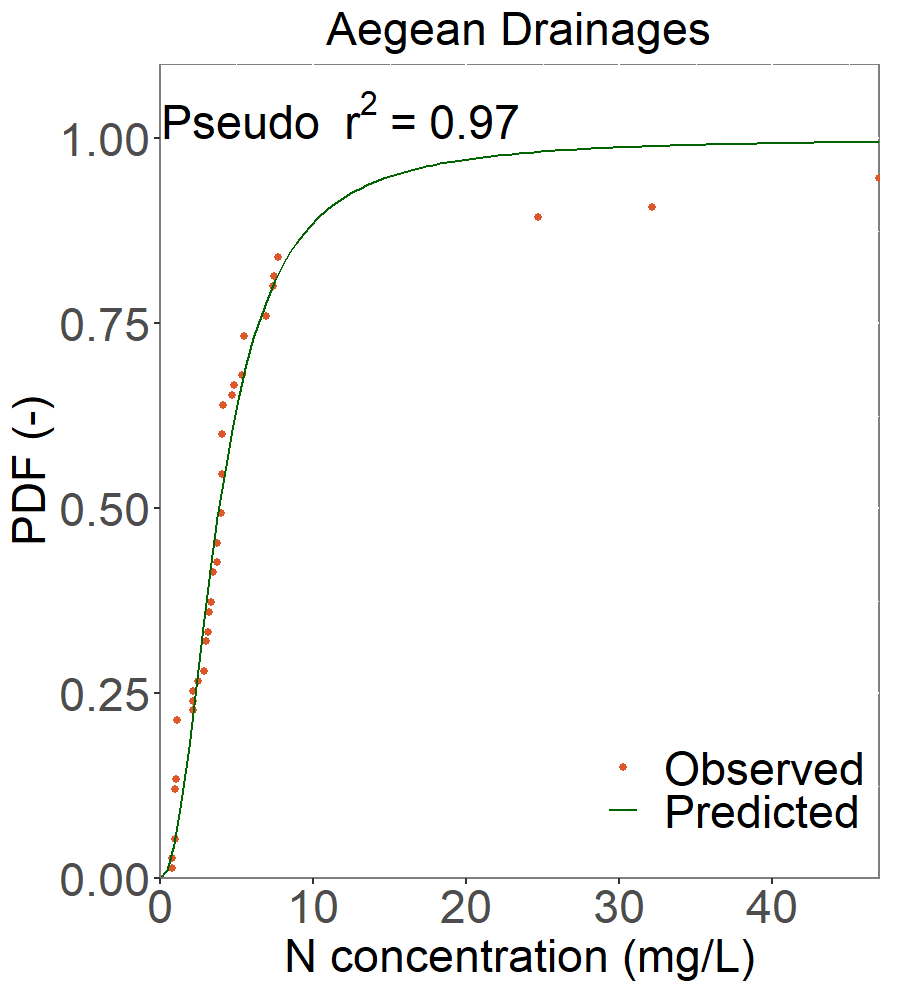

Supplement: Supplementary file 2 — es2c09333_si_002.zip [file es2c09333_si_002.zip › SSD_Ecoregion/Aegean Drainages.tif]

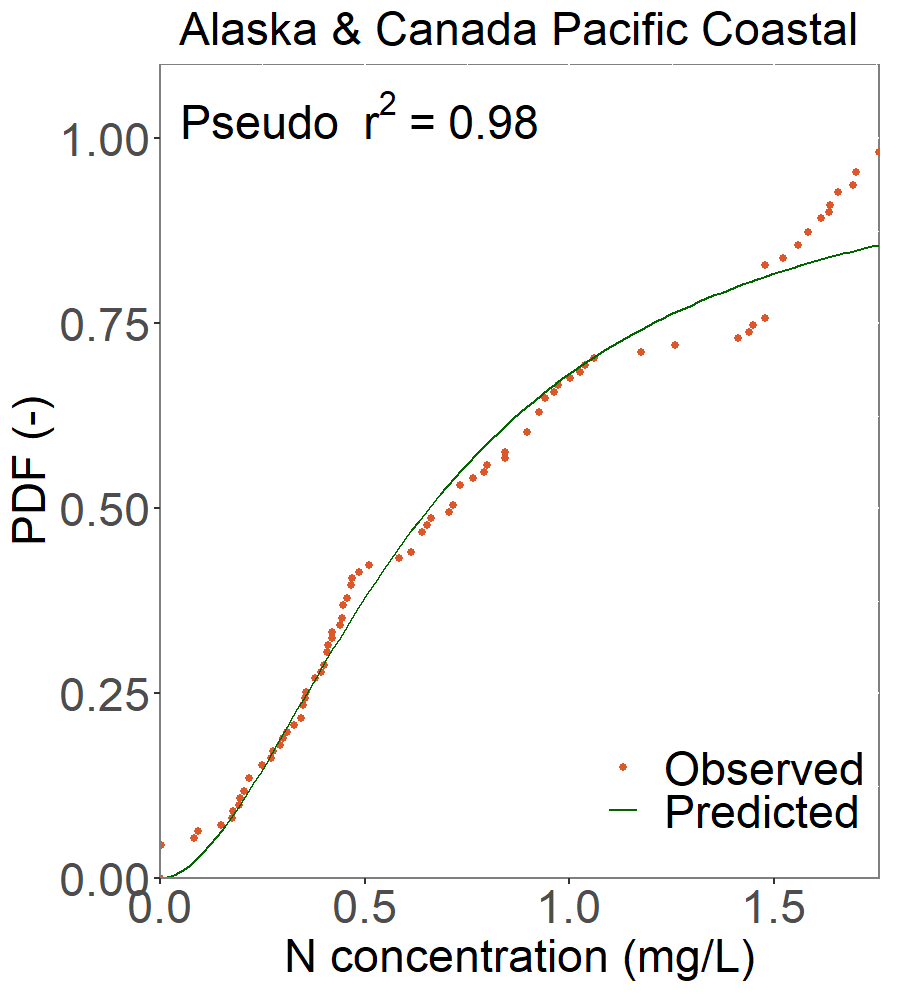

Supplement: Supplementary file 2 — es2c09333_si_002.zip [file es2c09333_si_002.zip › SSD_Ecoregion/Alaska & Canada Pacific Coastal.tif]

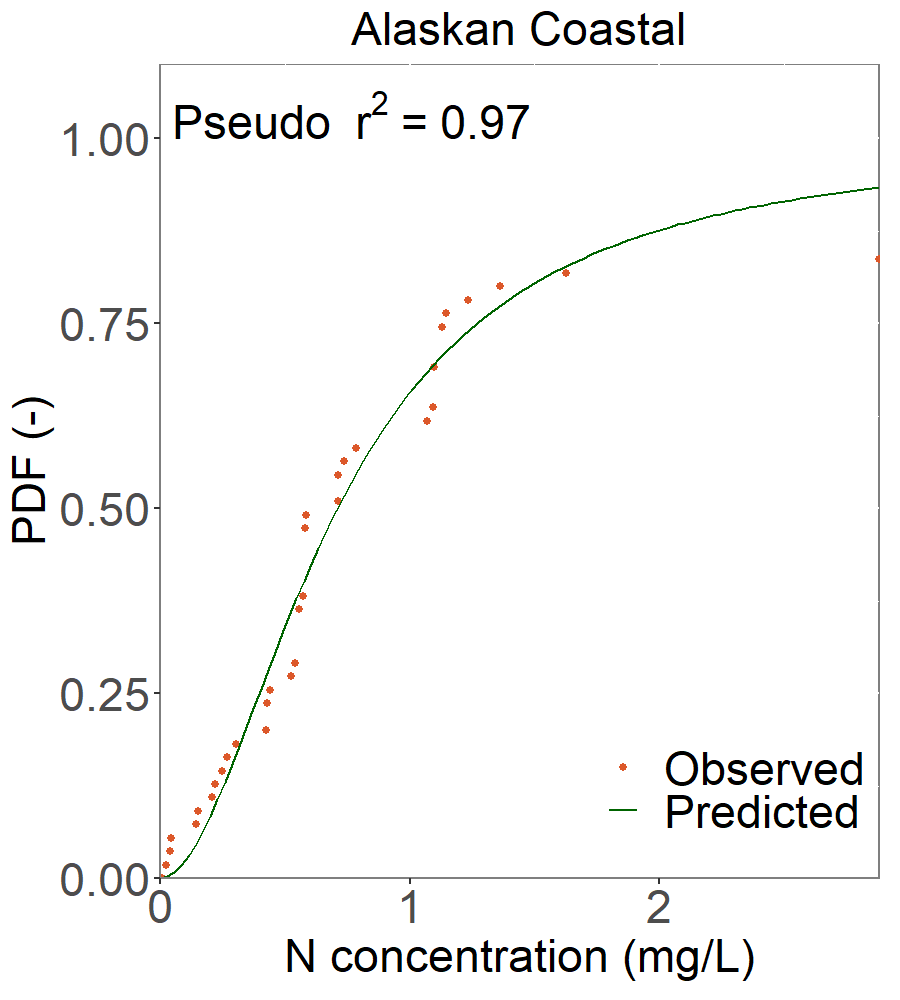

Supplement: Supplementary file 2 — es2c09333_si_002.zip [file es2c09333_si_002.zip › SSD_Ecoregion/Alaskan Coastal.tif]

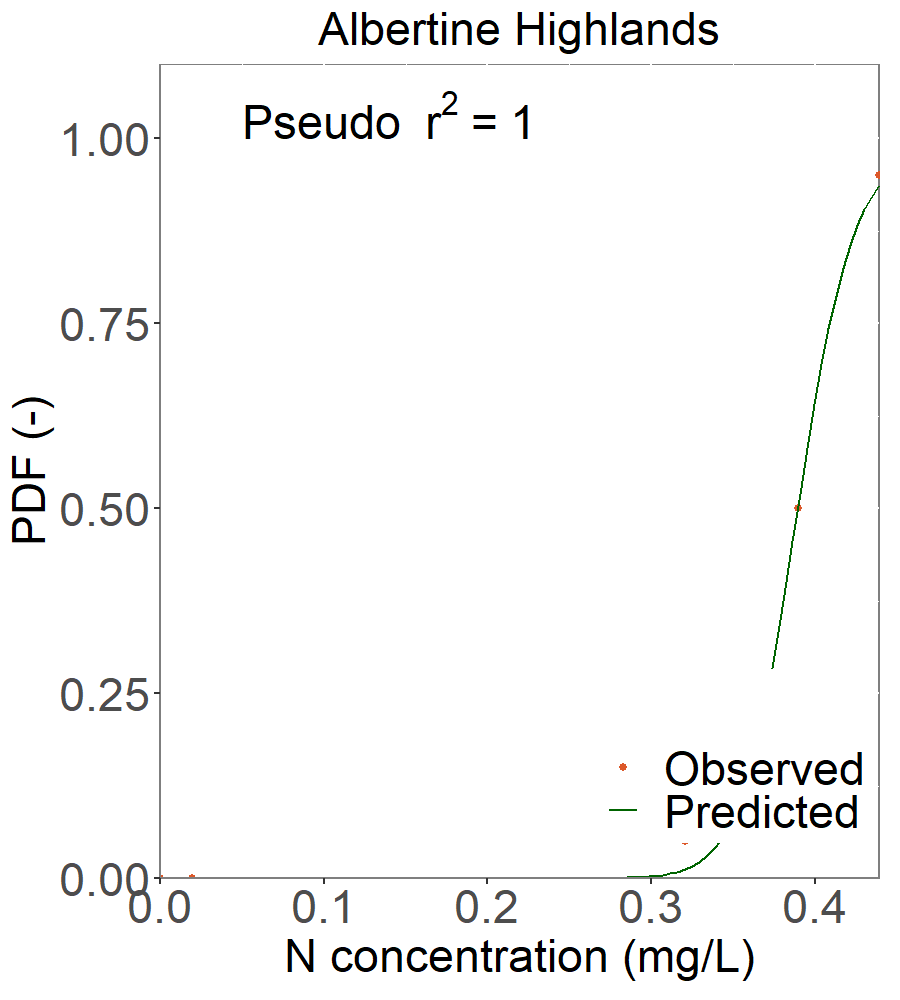

Supplement: Supplementary file 2 — es2c09333_si_002.zip [file es2c09333_si_002.zip › SSD_Ecoregion/Albertine Highlands.tif]

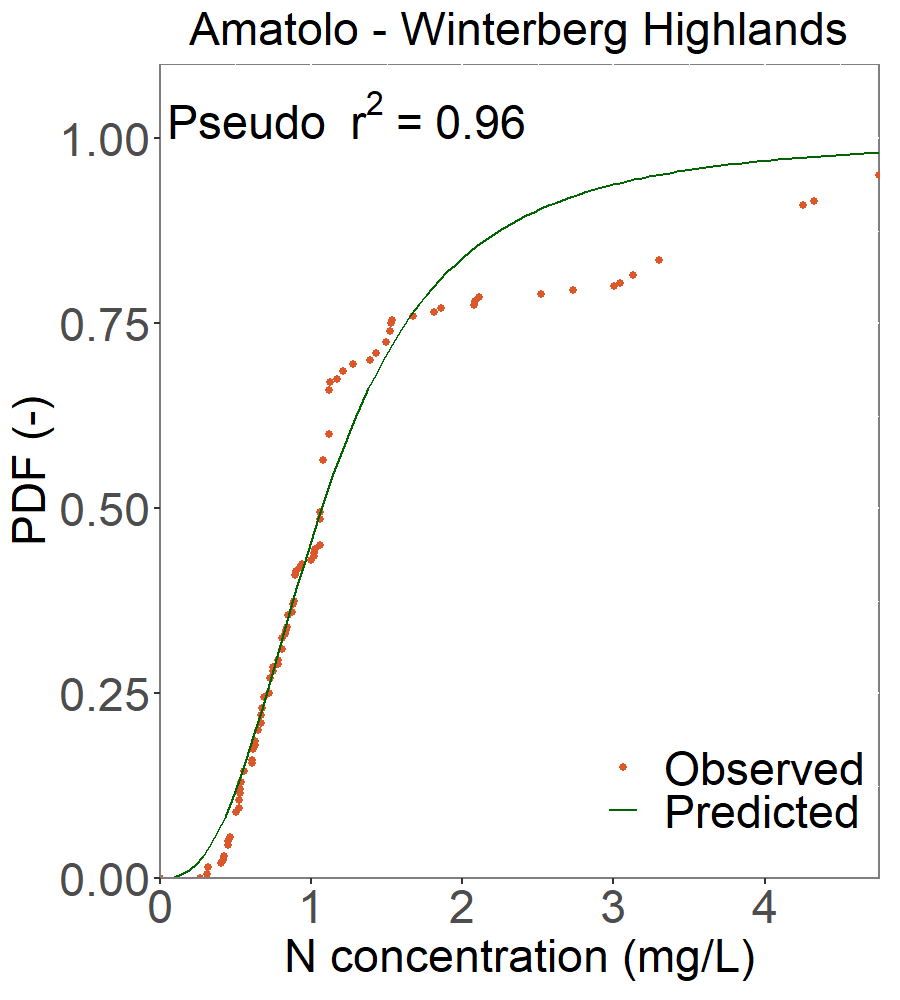

Supplement: Supplementary file 2 — es2c09333_si_002.zip [file es2c09333_si_002.zip › SSD_Ecoregion/Amatolo - Winterberg Highlands.tif]

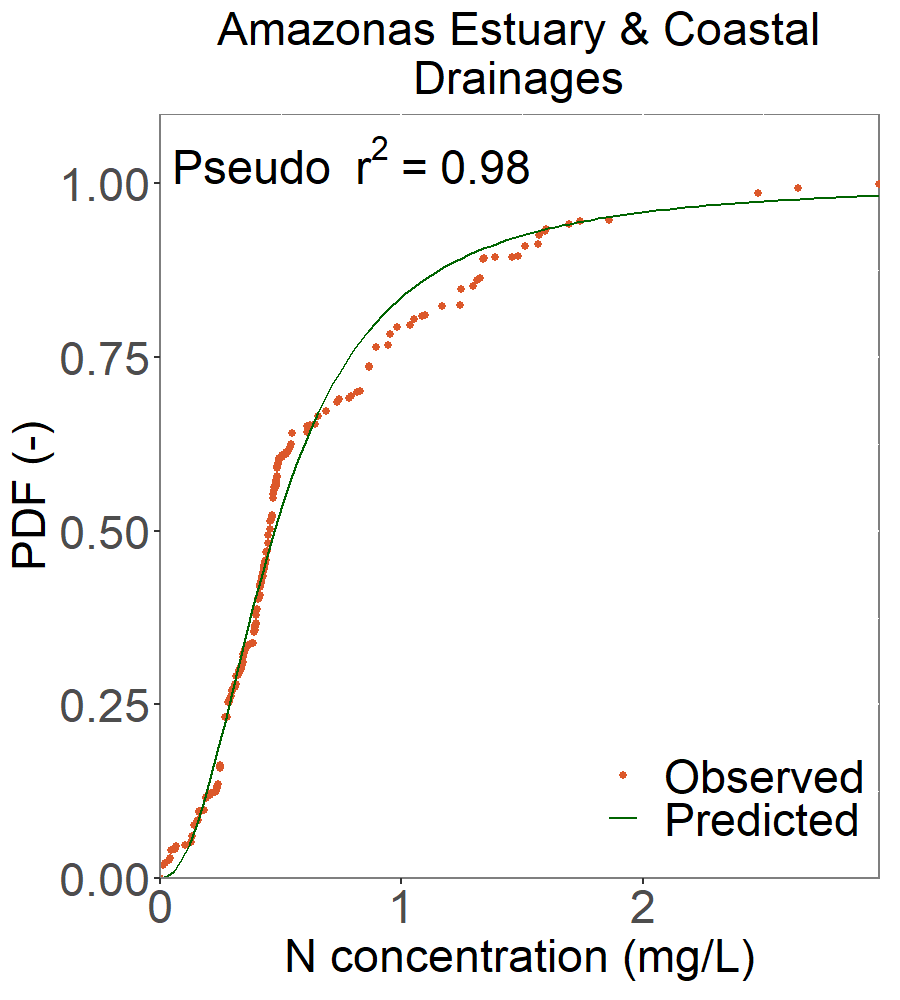

Supplement: Supplementary file 2 — es2c09333_si_002.zip [file es2c09333_si_002.zip › SSD_Ecoregion/Amazonas Estuary & Coastal Drainages.tif]

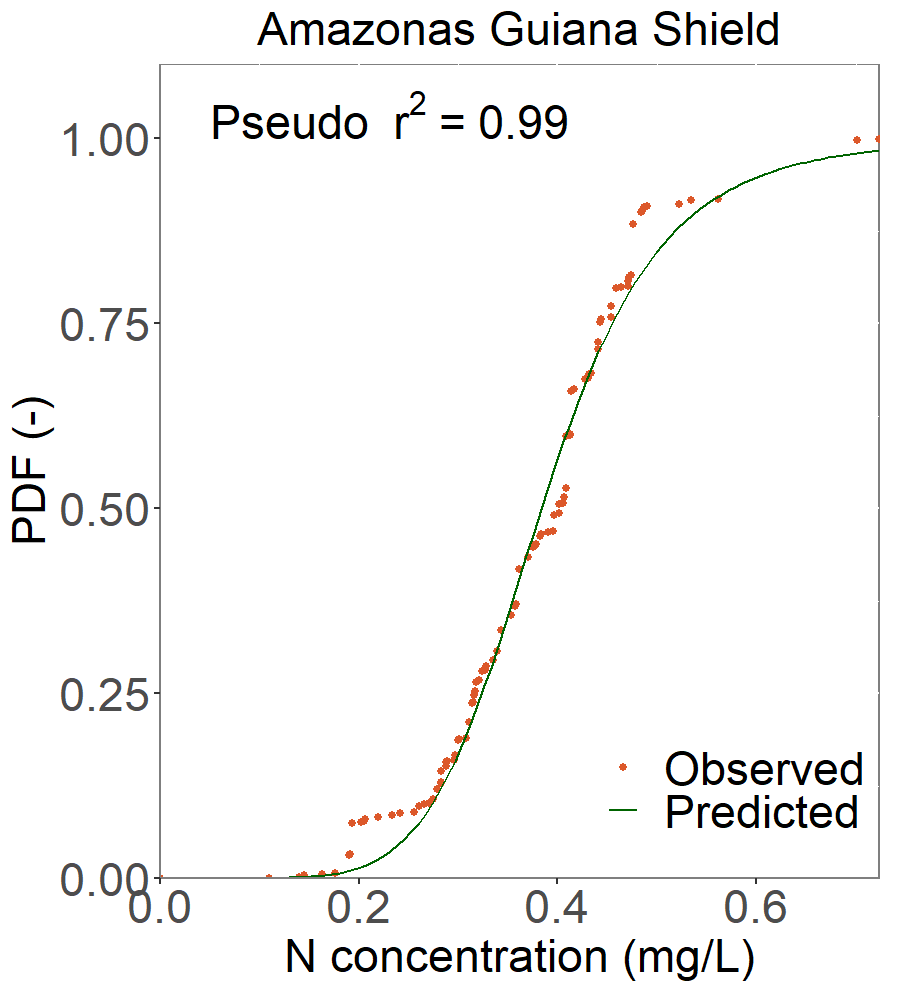

Supplement: Supplementary file 2 — es2c09333_si_002.zip [file es2c09333_si_002.zip › SSD_Ecoregion/Amazonas Guiana Shield.tif]

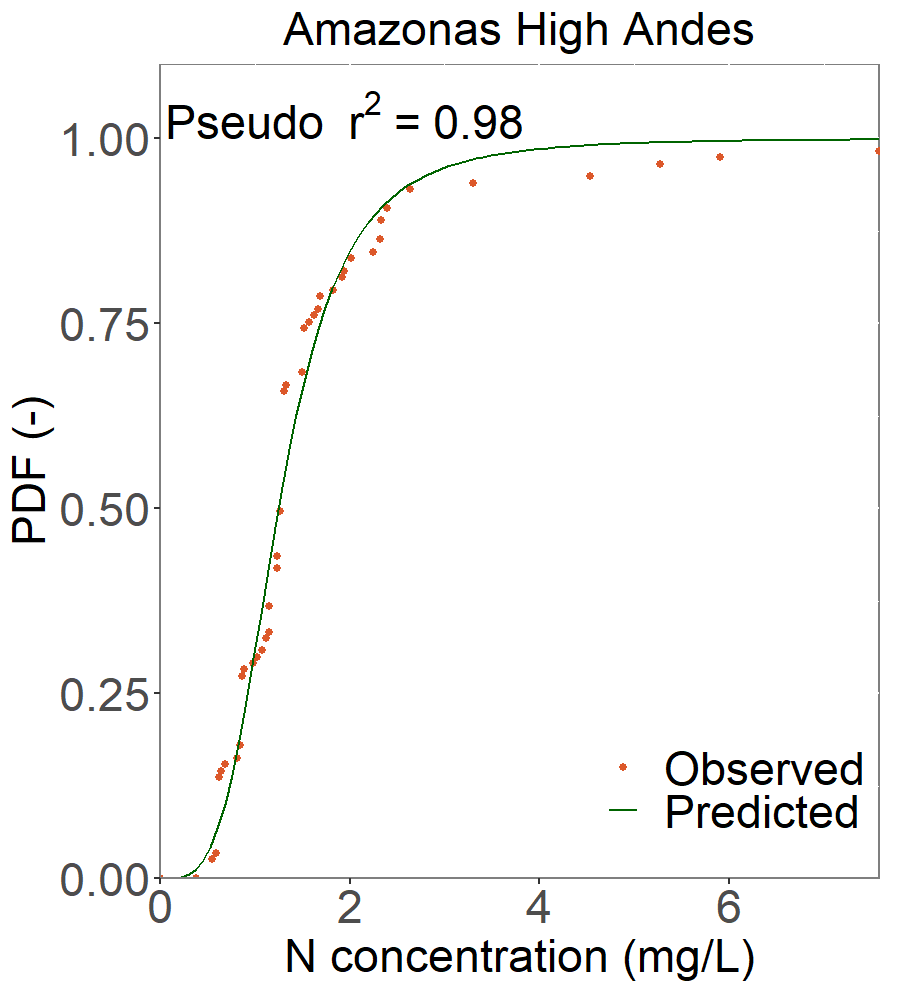

Supplement: Supplementary file 2 — es2c09333_si_002.zip [file es2c09333_si_002.zip › SSD_Ecoregion/Amazonas High Andes.tif]

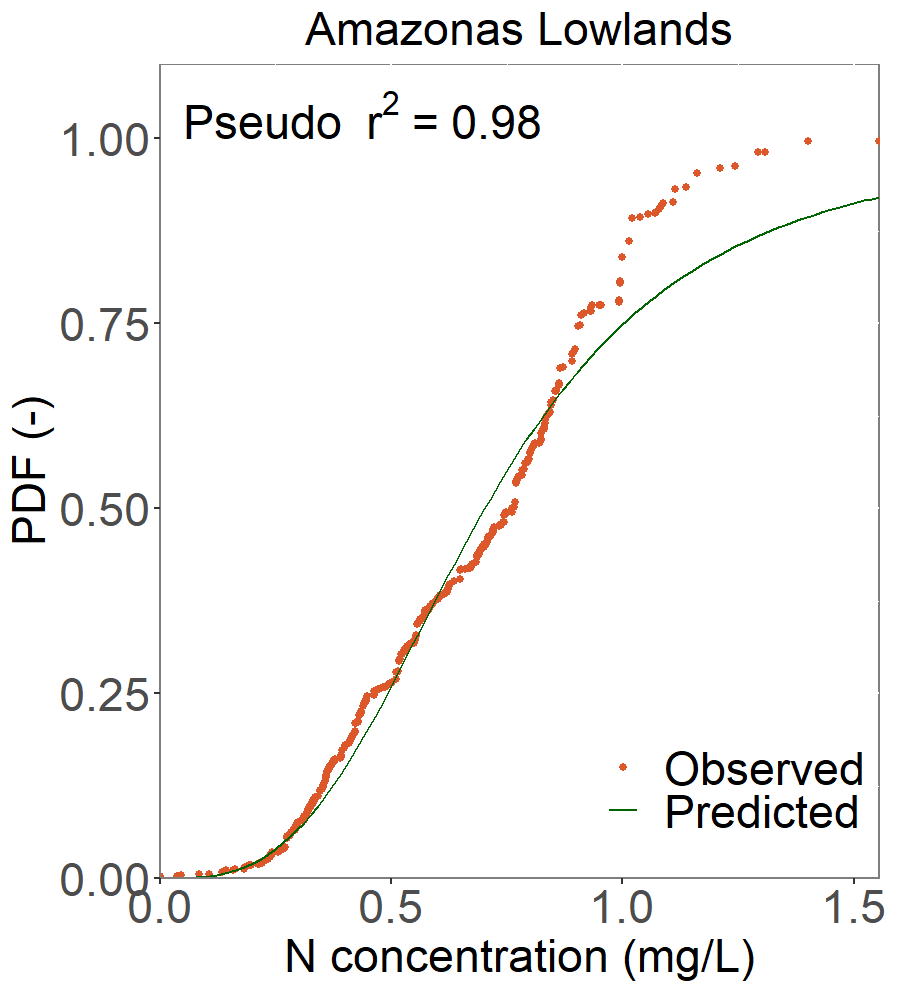

Supplement: Supplementary file 2 — es2c09333_si_002.zip [file es2c09333_si_002.zip › SSD_Ecoregion/Amazonas Lowlands.tif]

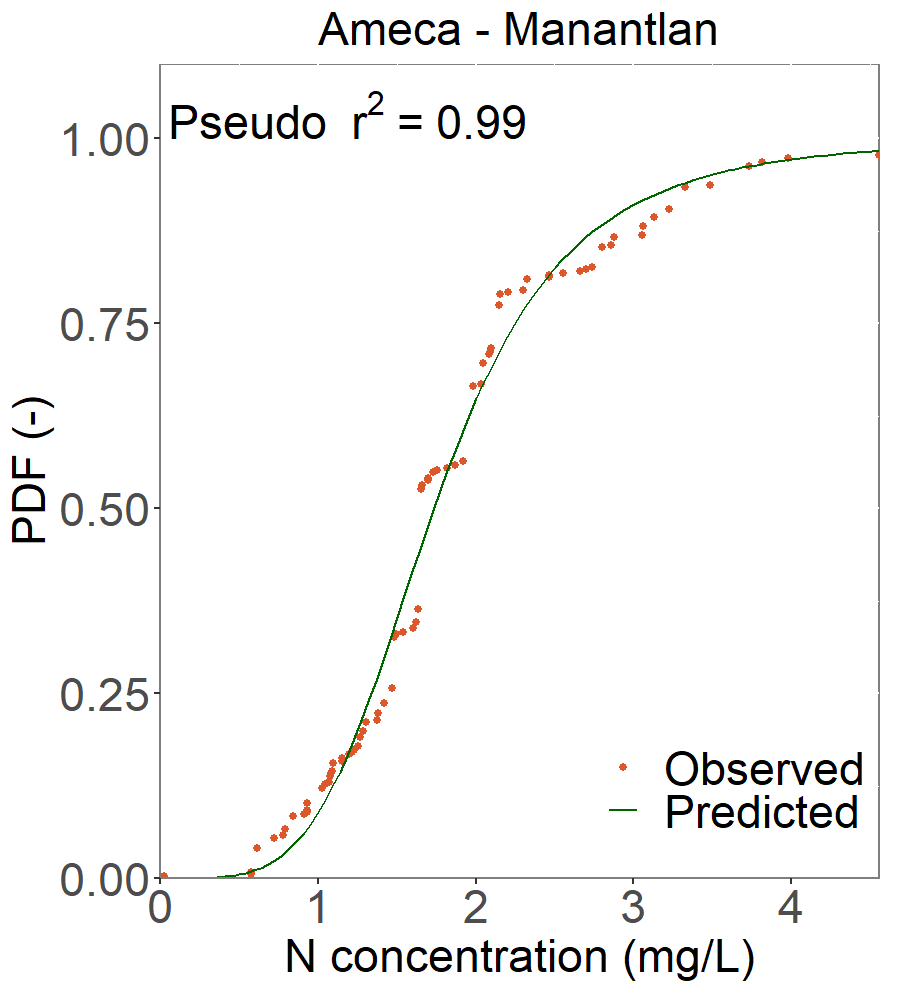

Supplement: Supplementary file 2 — es2c09333_si_002.zip [file es2c09333_si_002.zip › SSD_Ecoregion/Ameca - Manantlan.tif]

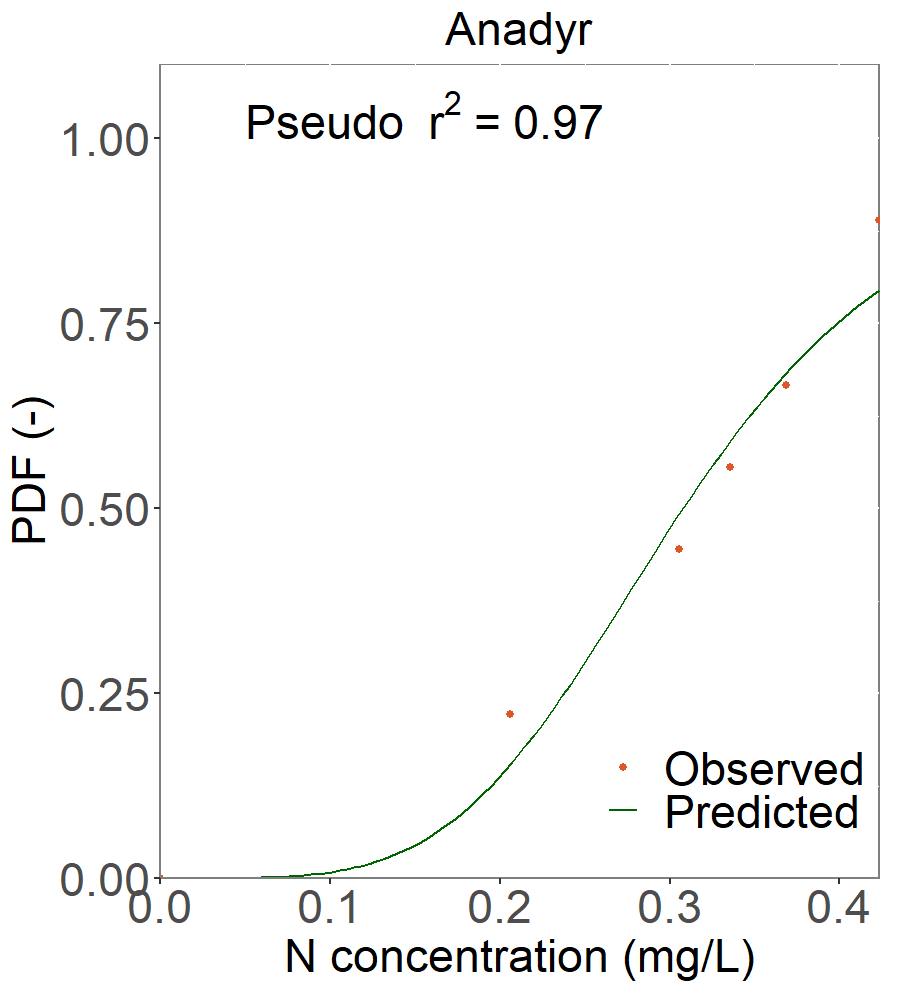

Supplement: Supplementary file 2 — es2c09333_si_002.zip [file es2c09333_si_002.zip › SSD_Ecoregion/Anadyr.tif]

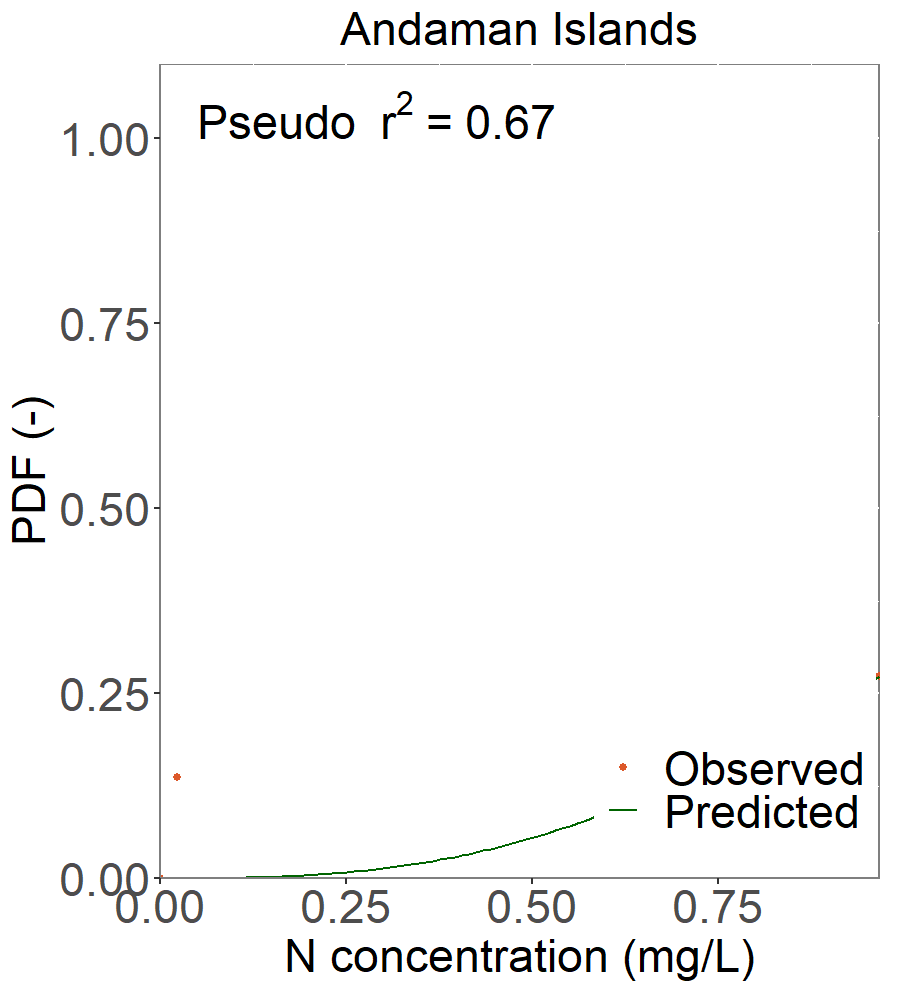

Supplement: Supplementary file 2 — es2c09333_si_002.zip [file es2c09333_si_002.zip › SSD_Ecoregion/Andaman Islands.tif]

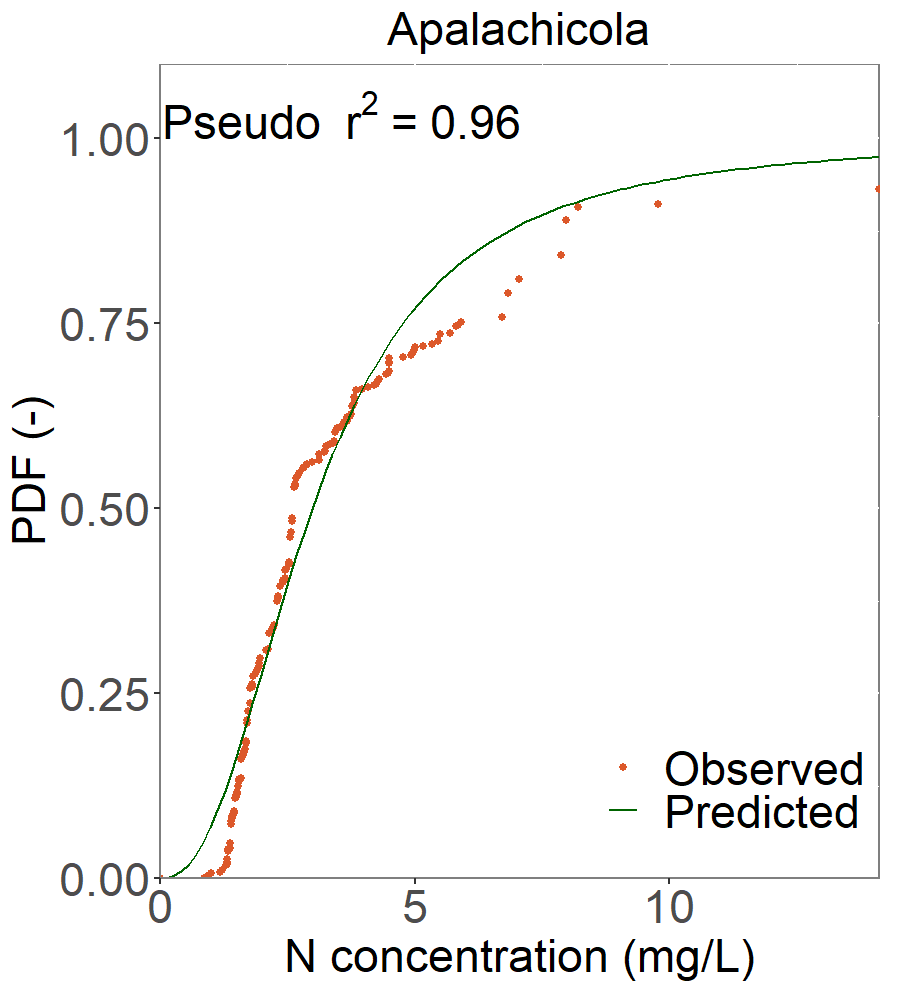

Supplement: Supplementary file 2 — es2c09333_si_002.zip [file es2c09333_si_002.zip › SSD_Ecoregion/Apalachicola.tif]

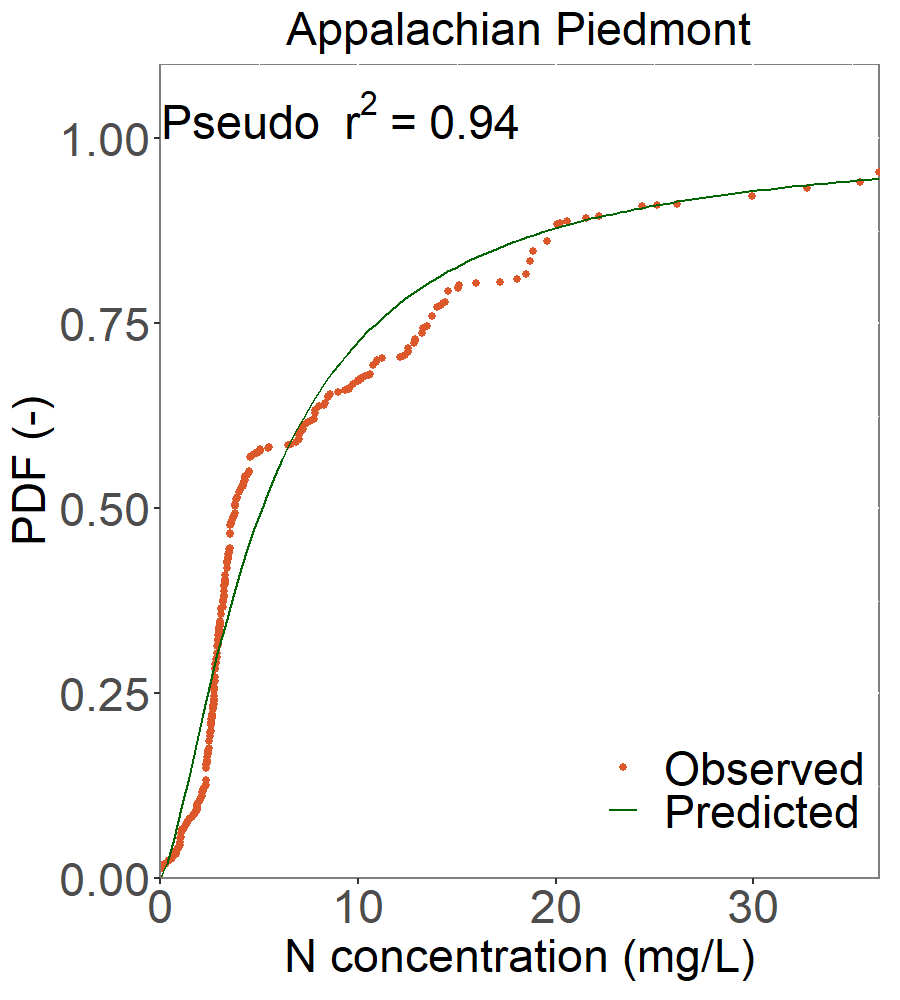

Supplement: Supplementary file 2 — es2c09333_si_002.zip [file es2c09333_si_002.zip › SSD_Ecoregion/Appalachian Piedmont.tif]

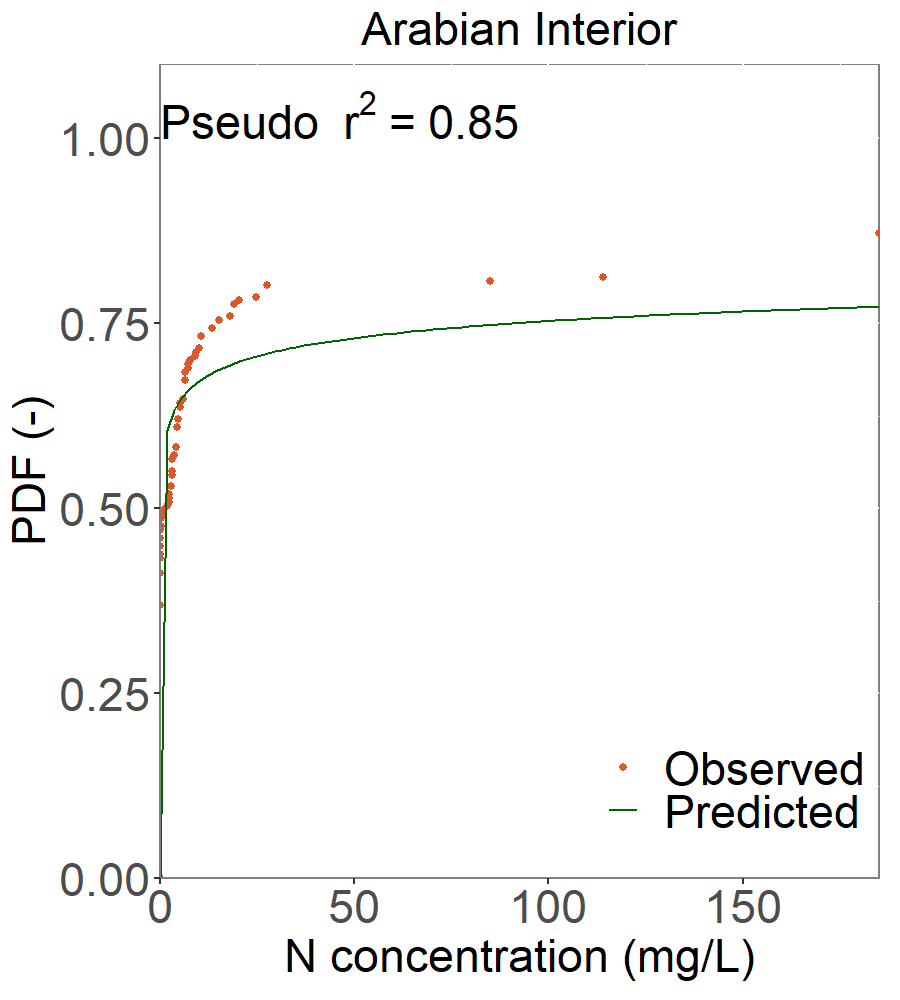

Supplement: Supplementary file 2 — es2c09333_si_002.zip [file es2c09333_si_002.zip › SSD_Ecoregion/Arabian Interior.tif]

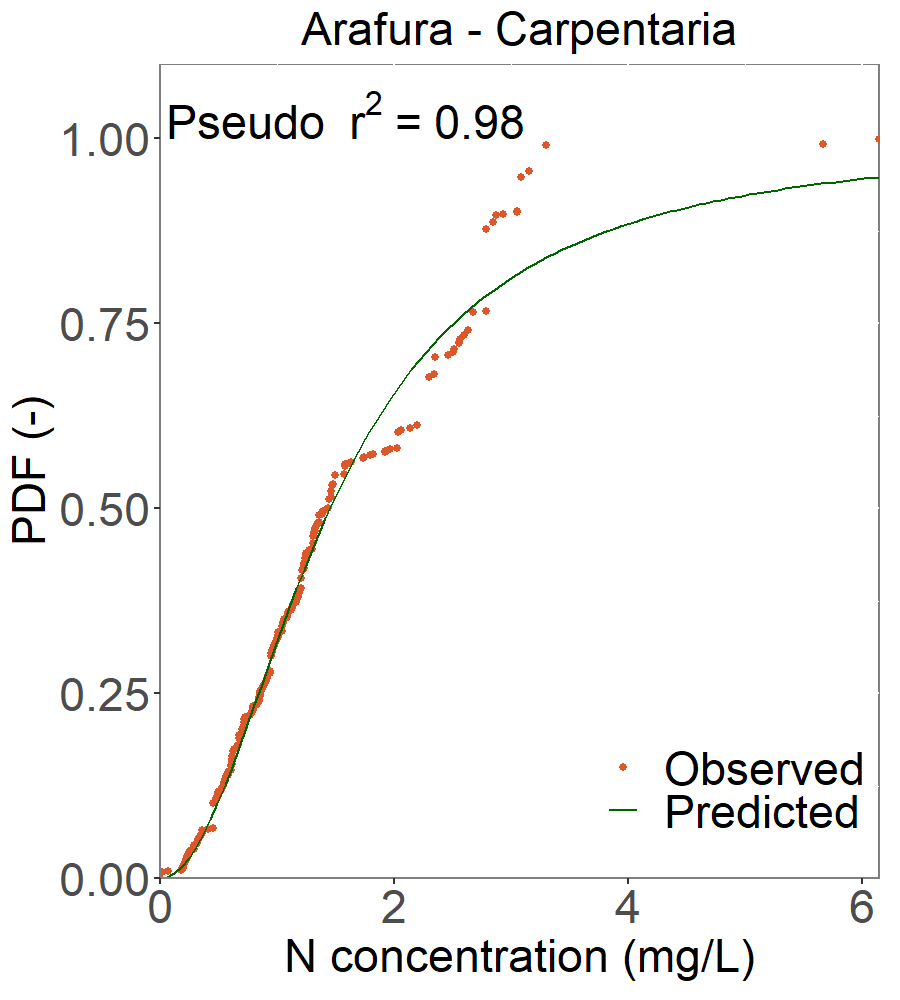

Supplement: Supplementary file 2 — es2c09333_si_002.zip [file es2c09333_si_002.zip › SSD_Ecoregion/Arafura - Carpentaria.tif]

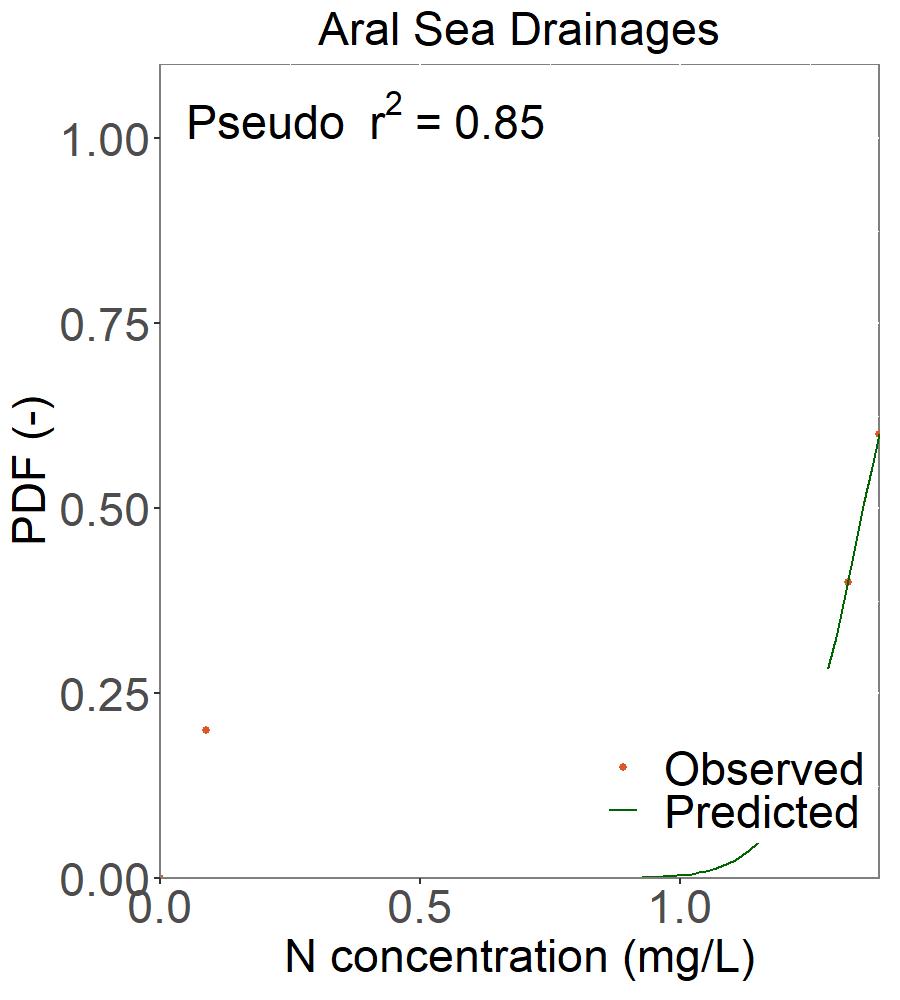

Supplement: Supplementary file 2 — es2c09333_si_002.zip [file es2c09333_si_002.zip › SSD_Ecoregion/Aral Sea Drainages.tif]

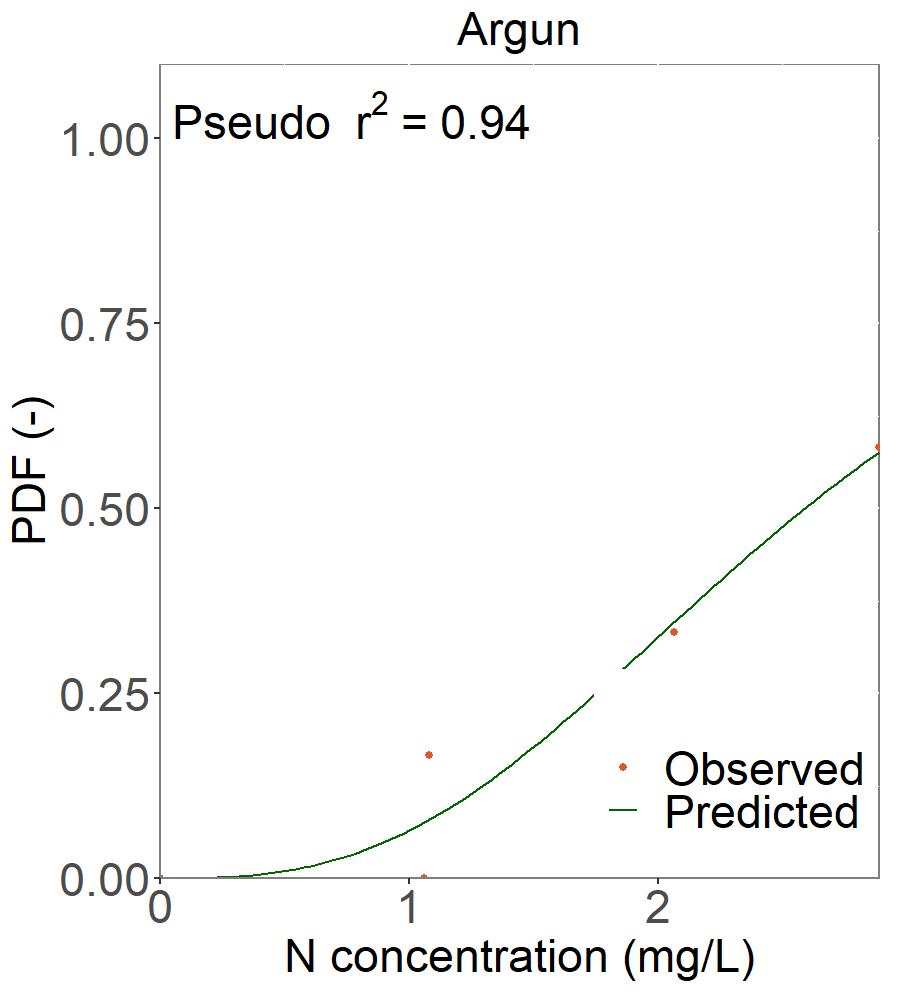

Supplement: Supplementary file 2 — es2c09333_si_002.zip [file es2c09333_si_002.zip › SSD_Ecoregion/Argun.tif]

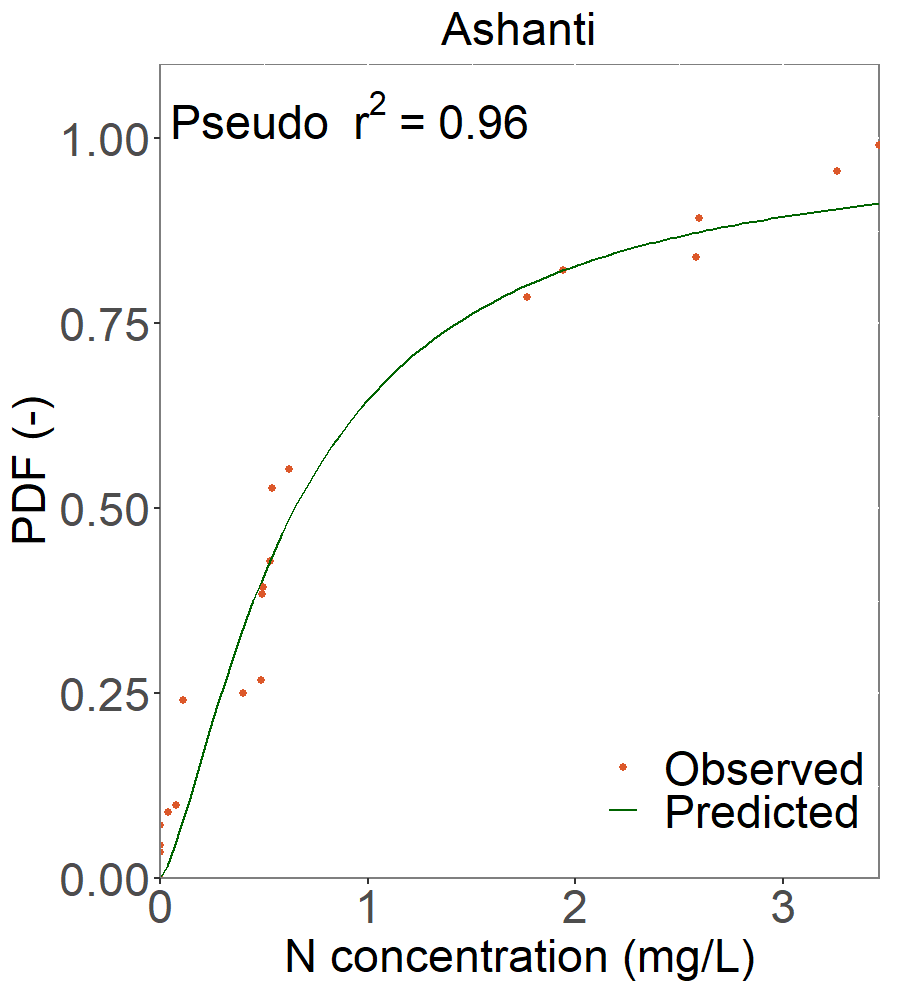

Supplement: Supplementary file 2 — es2c09333_si_002.zip [file es2c09333_si_002.zip › SSD_Ecoregion/Ashanti.tif]

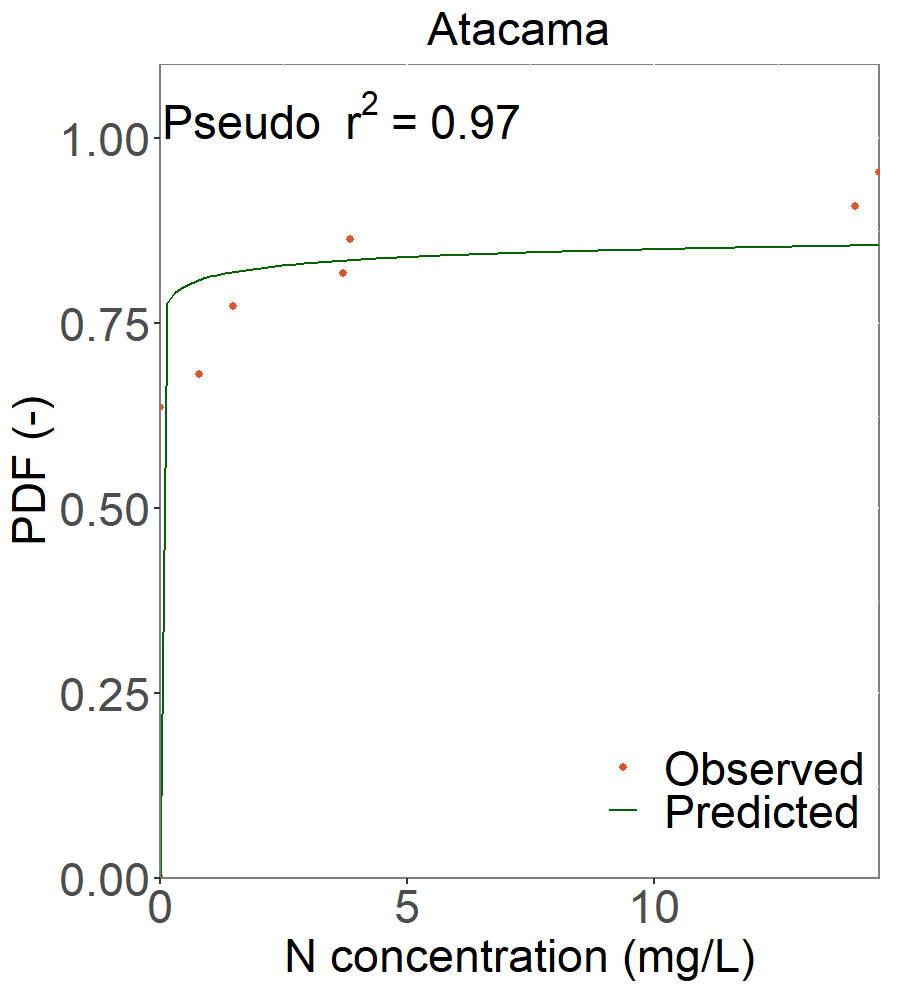

Supplement: Supplementary file 2 — es2c09333_si_002.zip [file es2c09333_si_002.zip › SSD_Ecoregion/Atacama.tif]

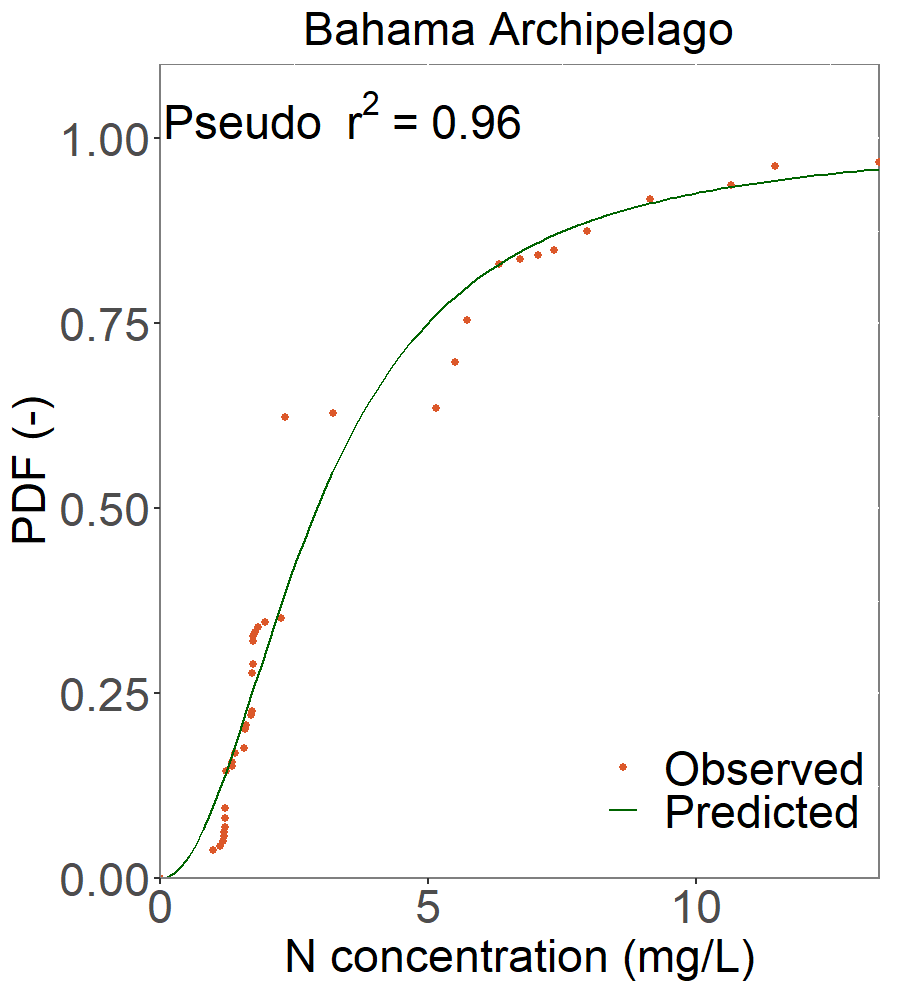

Supplement: Supplementary file 2 — es2c09333_si_002.zip [file es2c09333_si_002.zip › SSD_Ecoregion/Bahama Archipelago.tif]

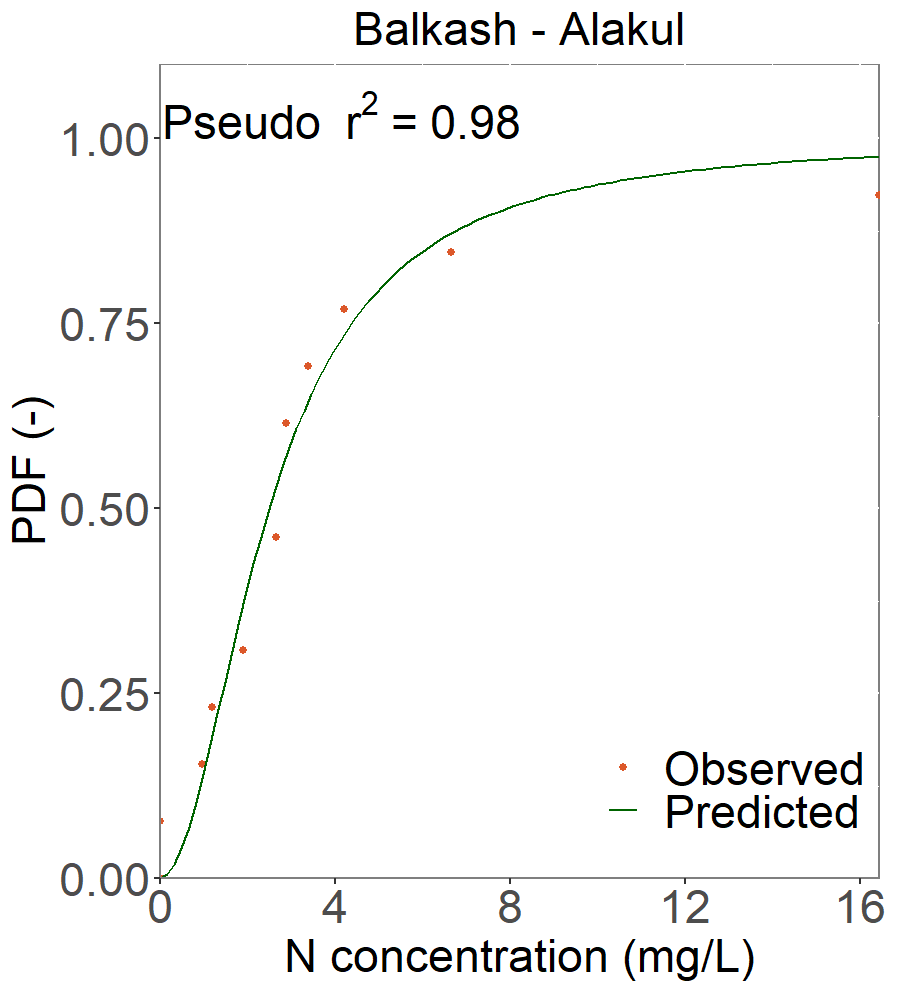

Supplement: Supplementary file 2 — es2c09333_si_002.zip [file es2c09333_si_002.zip › SSD_Ecoregion/Balkash - Alakul.tif]

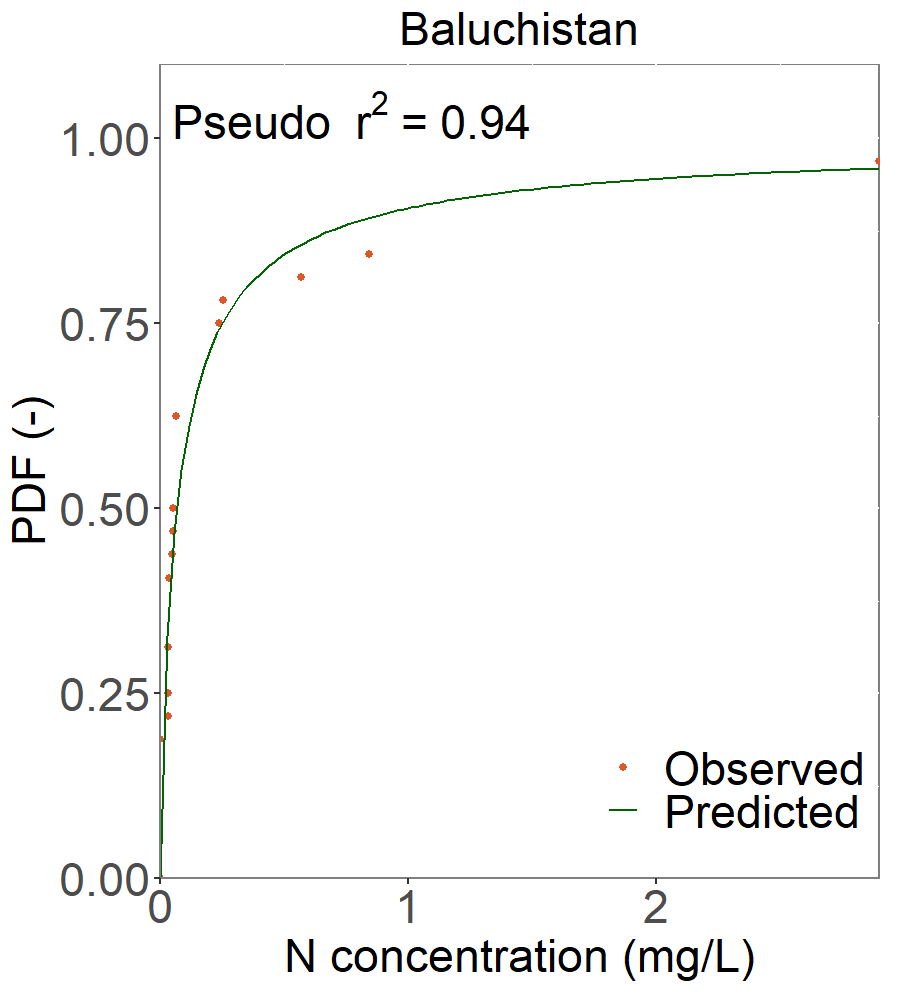

Supplement: Supplementary file 2 — es2c09333_si_002.zip [file es2c09333_si_002.zip › SSD_Ecoregion/Baluchistan.tif]

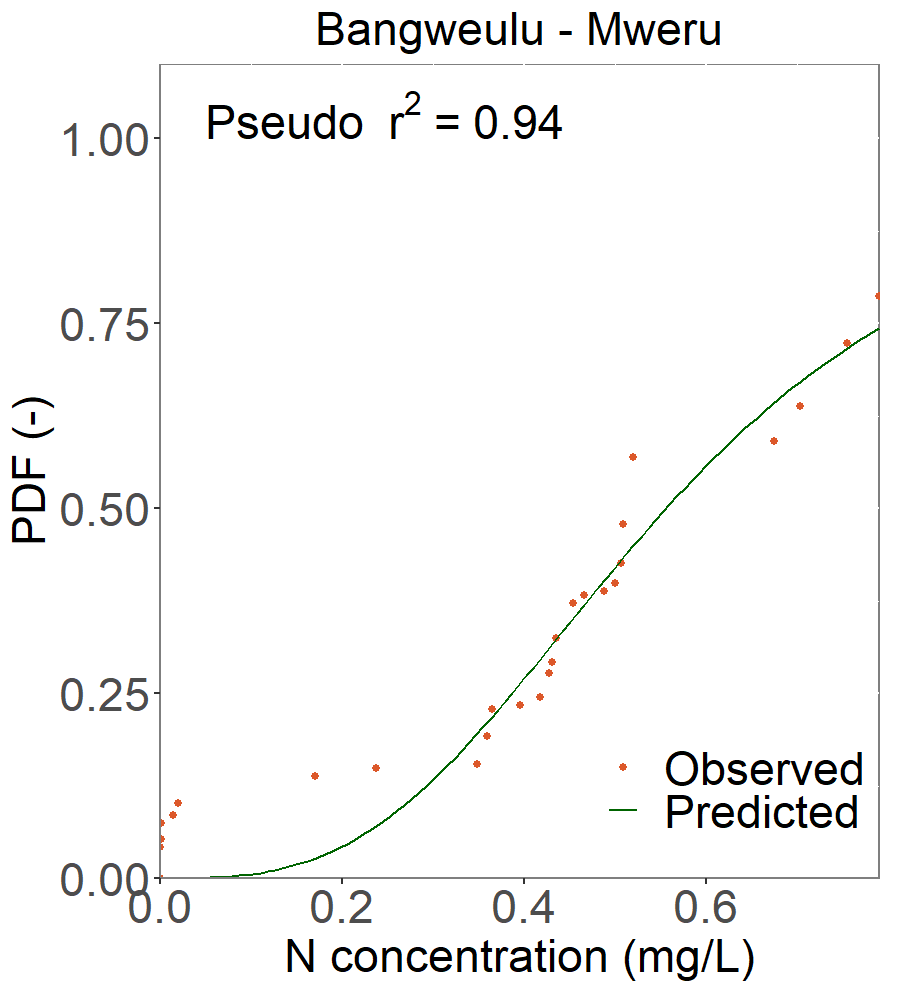

Supplement: Supplementary file 2 — es2c09333_si_002.zip [file es2c09333_si_002.zip › SSD_Ecoregion/Bangweulu - Mweru.tif]

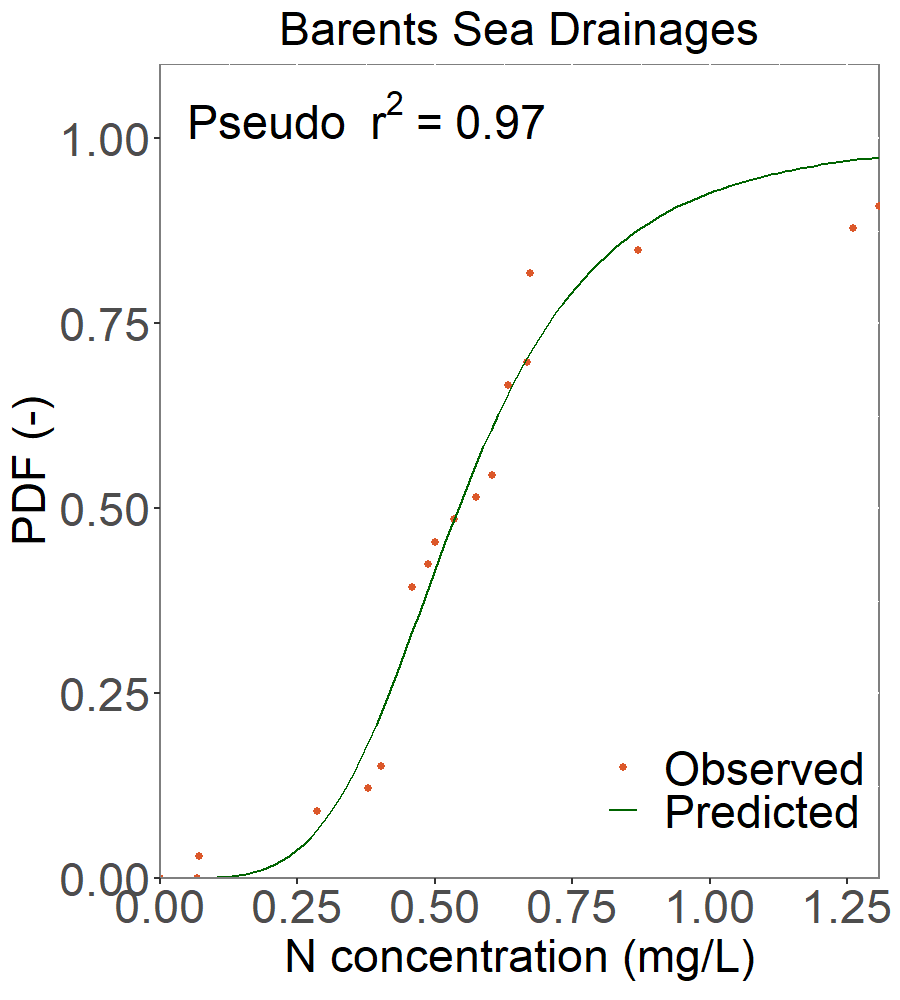

Supplement: Supplementary file 2 — es2c09333_si_002.zip [file es2c09333_si_002.zip › SSD_Ecoregion/Barents Sea Drainages.tif]

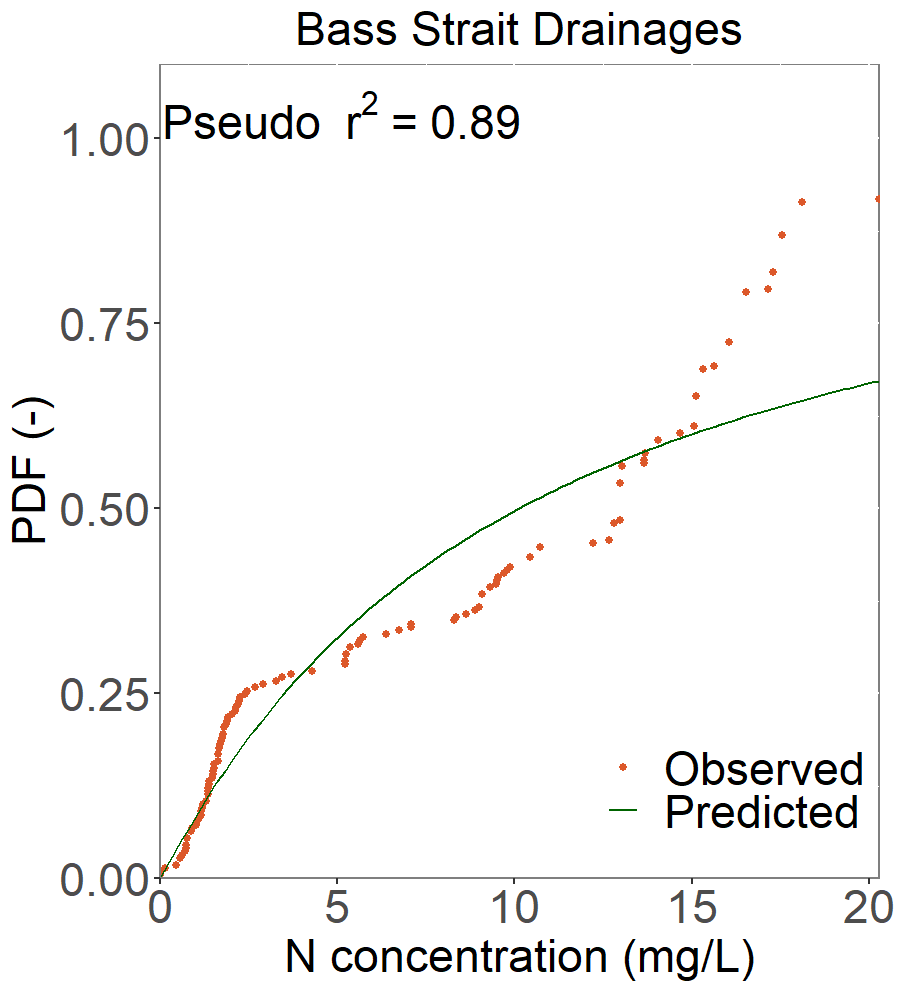

Supplement: Supplementary file 2 — es2c09333_si_002.zip [file es2c09333_si_002.zip › SSD_Ecoregion/Bass Strait Drainages.tif]

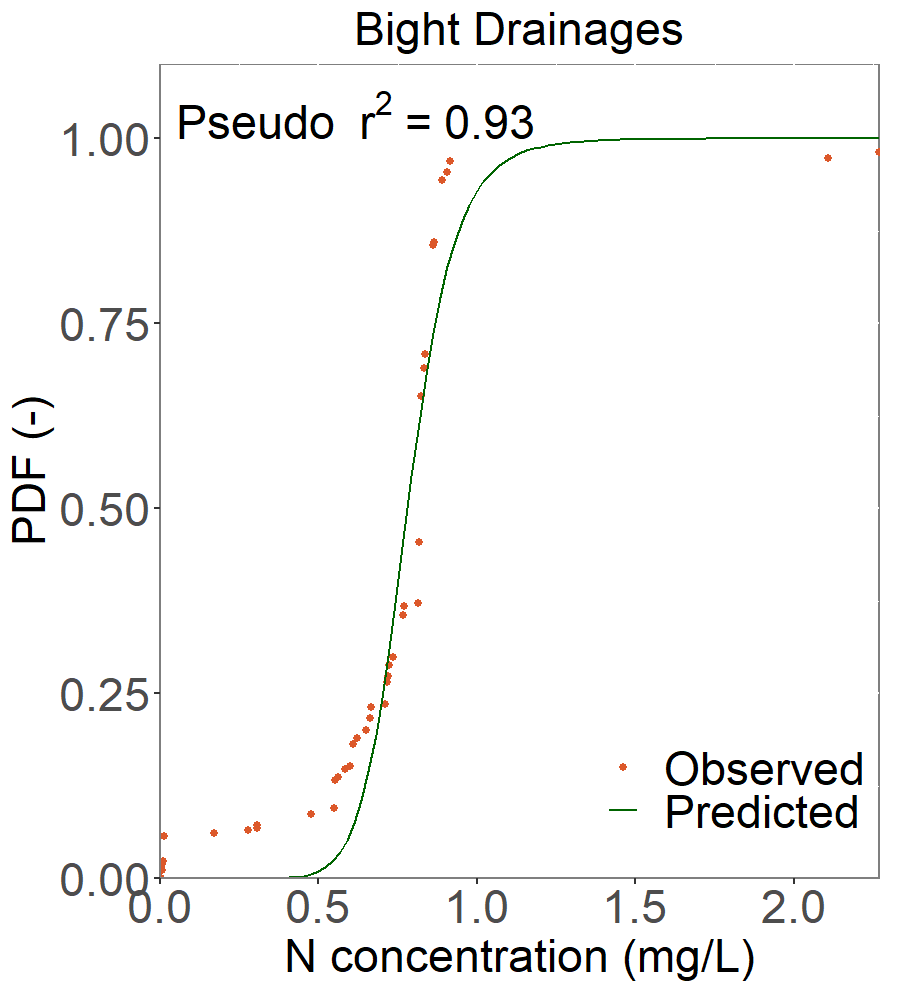

Supplement: Supplementary file 2 — es2c09333_si_002.zip [file es2c09333_si_002.zip › SSD_Ecoregion/Bight Drainages.tif]

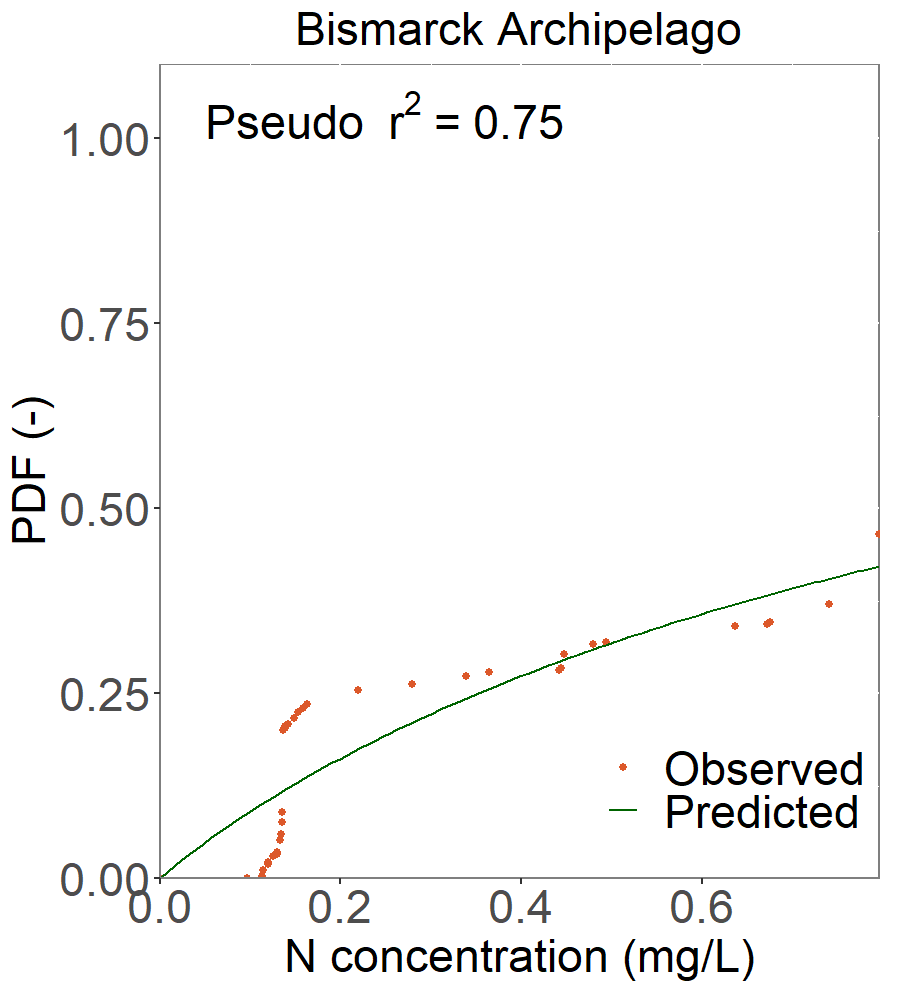

Supplement: Supplementary file 2 — es2c09333_si_002.zip [file es2c09333_si_002.zip › SSD_Ecoregion/Bismarck Archipelago.tif]

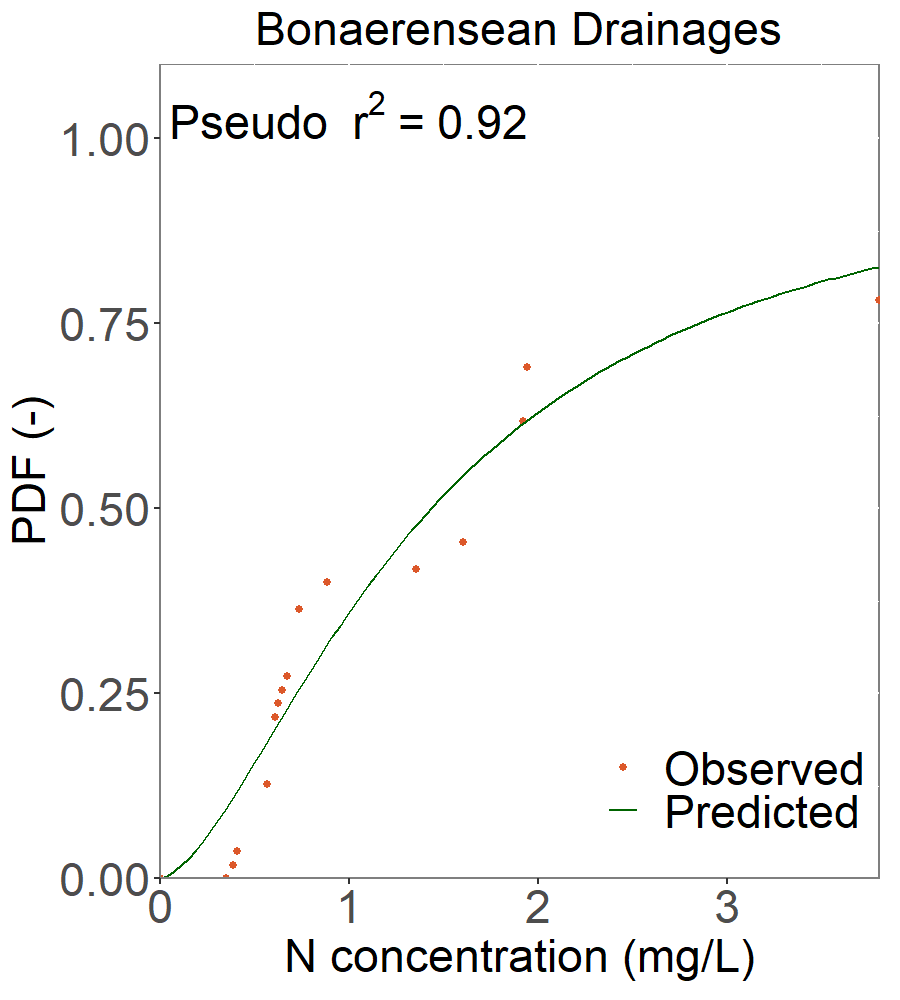

Supplement: Supplementary file 2 — es2c09333_si_002.zip [file es2c09333_si_002.zip › SSD_Ecoregion/Bonaerensean Drainages.tif]

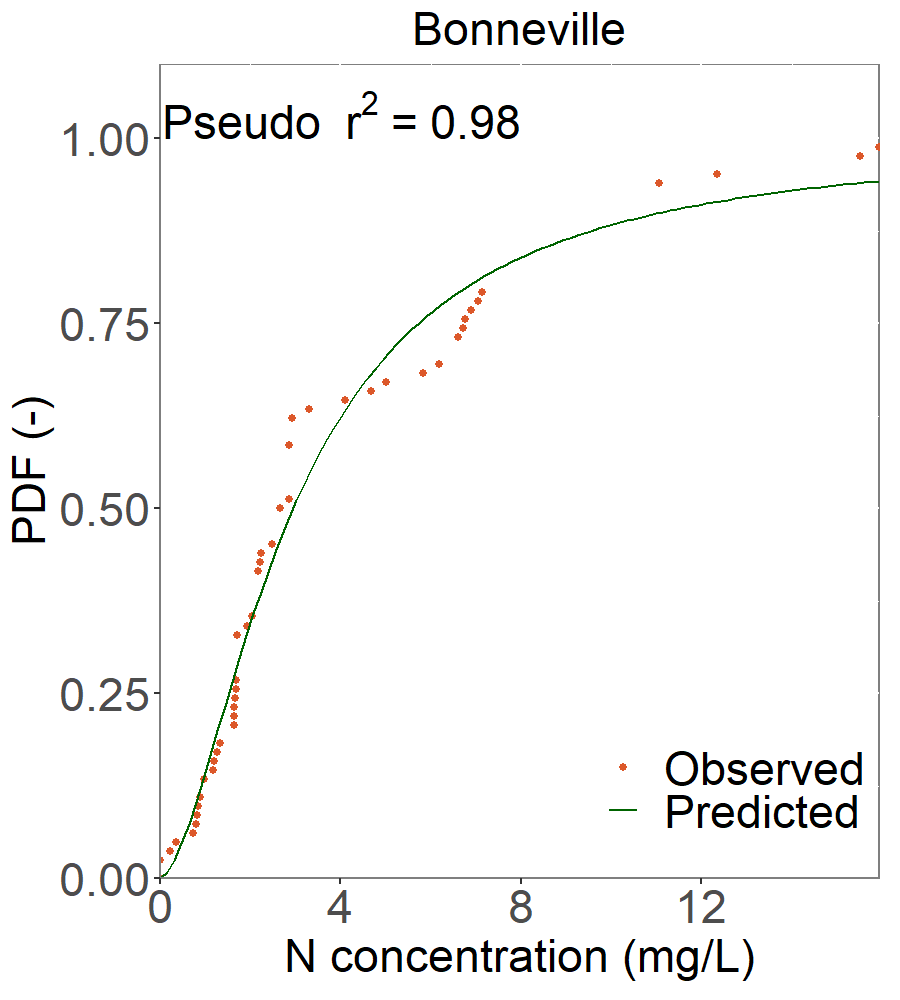

Supplement: Supplementary file 2 — es2c09333_si_002.zip [file es2c09333_si_002.zip › SSD_Ecoregion/Bonneville.tif]

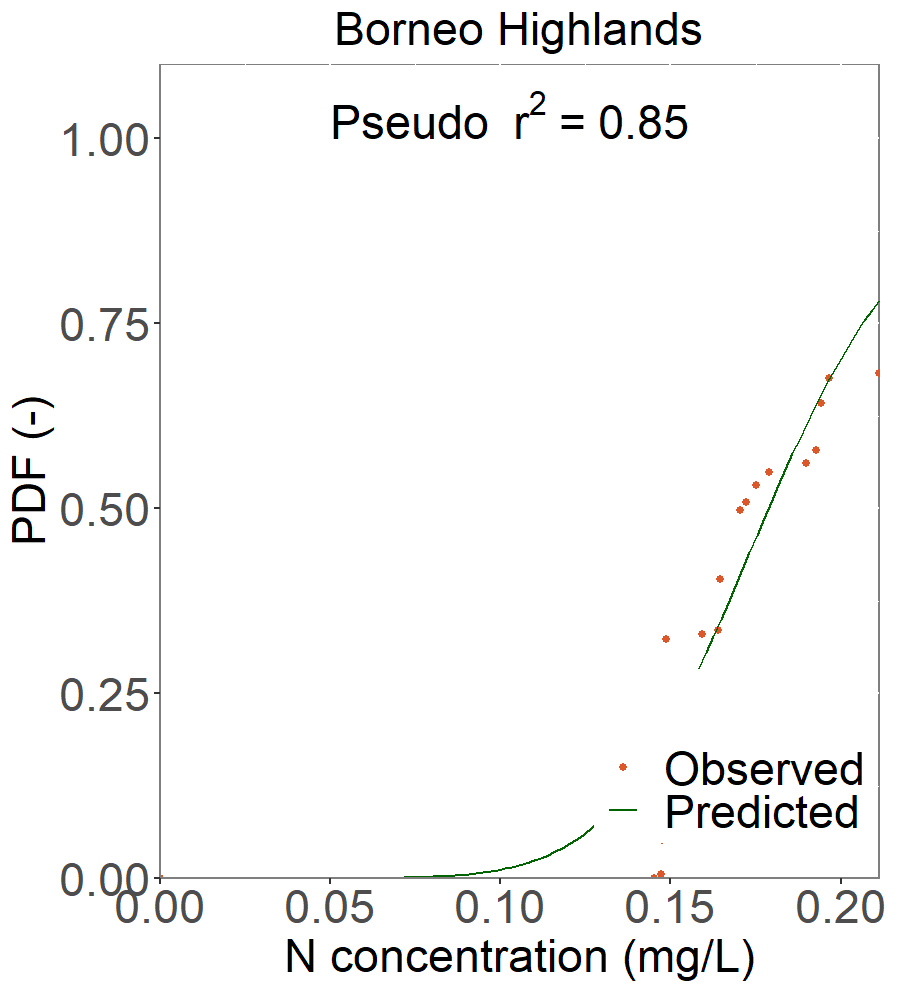

Supplement: Supplementary file 2 — es2c09333_si_002.zip [file es2c09333_si_002.zip › SSD_Ecoregion/Borneo Highlands.tif]

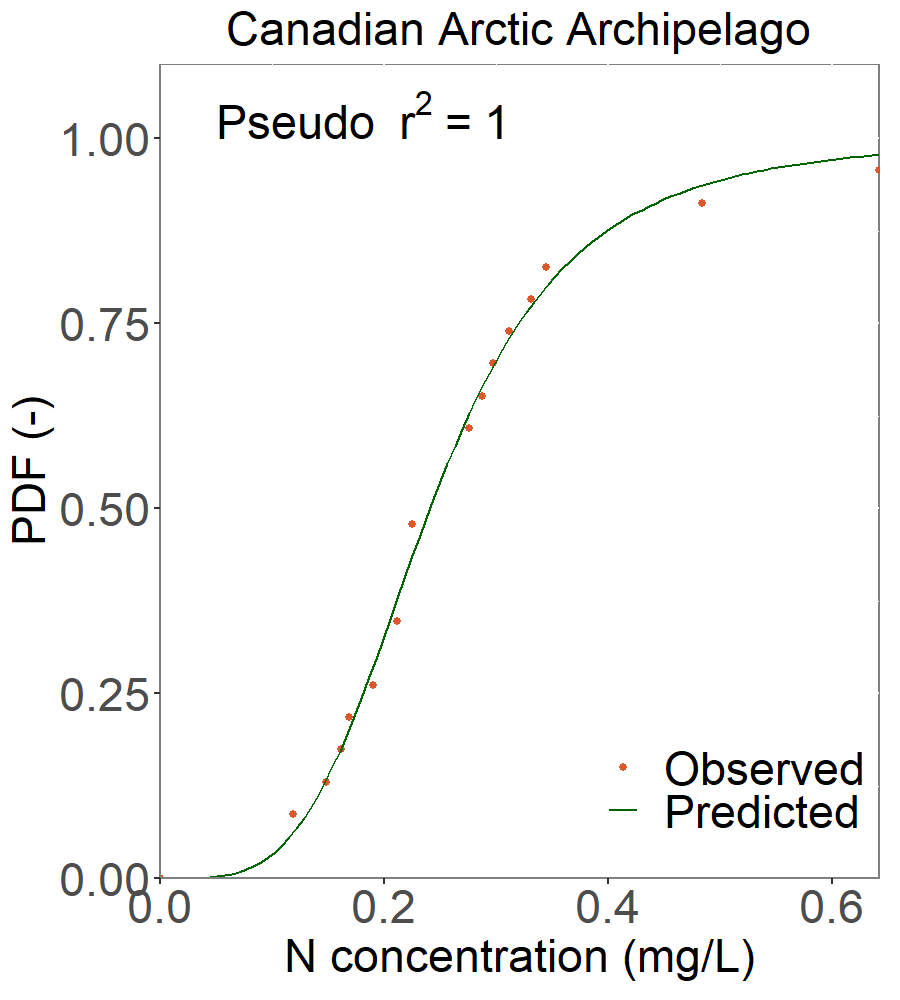

Supplement: Supplementary file 2 — es2c09333_si_002.zip [file es2c09333_si_002.zip › SSD_Ecoregion/Canadian Arctic Archipelago.tif]

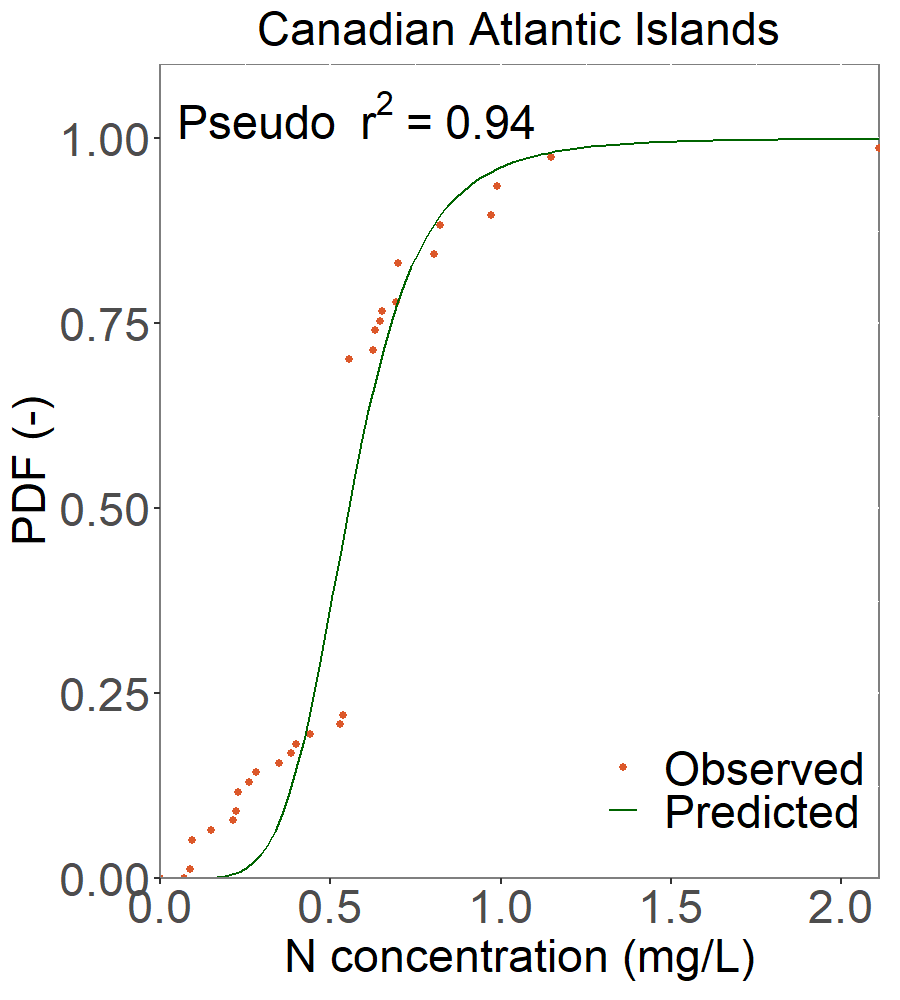

Supplement: Supplementary file 2 — es2c09333_si_002.zip [file es2c09333_si_002.zip › SSD_Ecoregion/Canadian Atlantic Islands.tif]

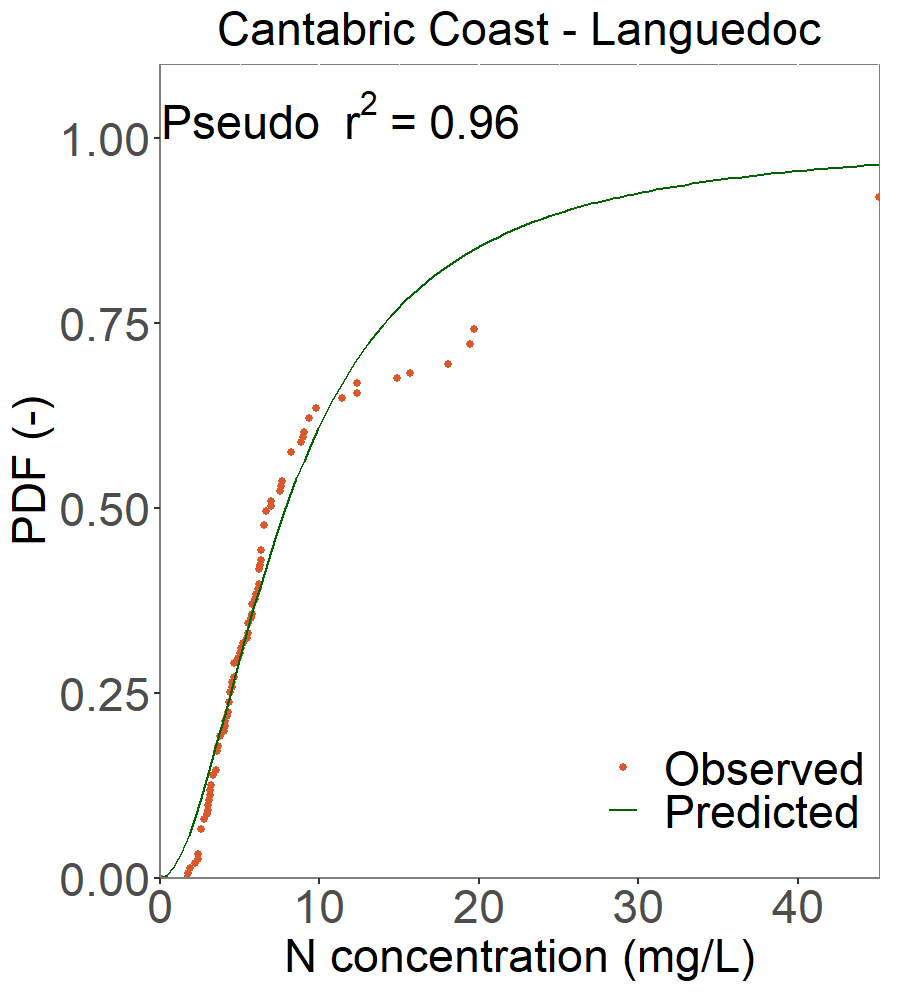

Supplement: Supplementary file 2 — es2c09333_si_002.zip [file es2c09333_si_002.zip › SSD_Ecoregion/Cantabric Coast - Languedoc.tif]

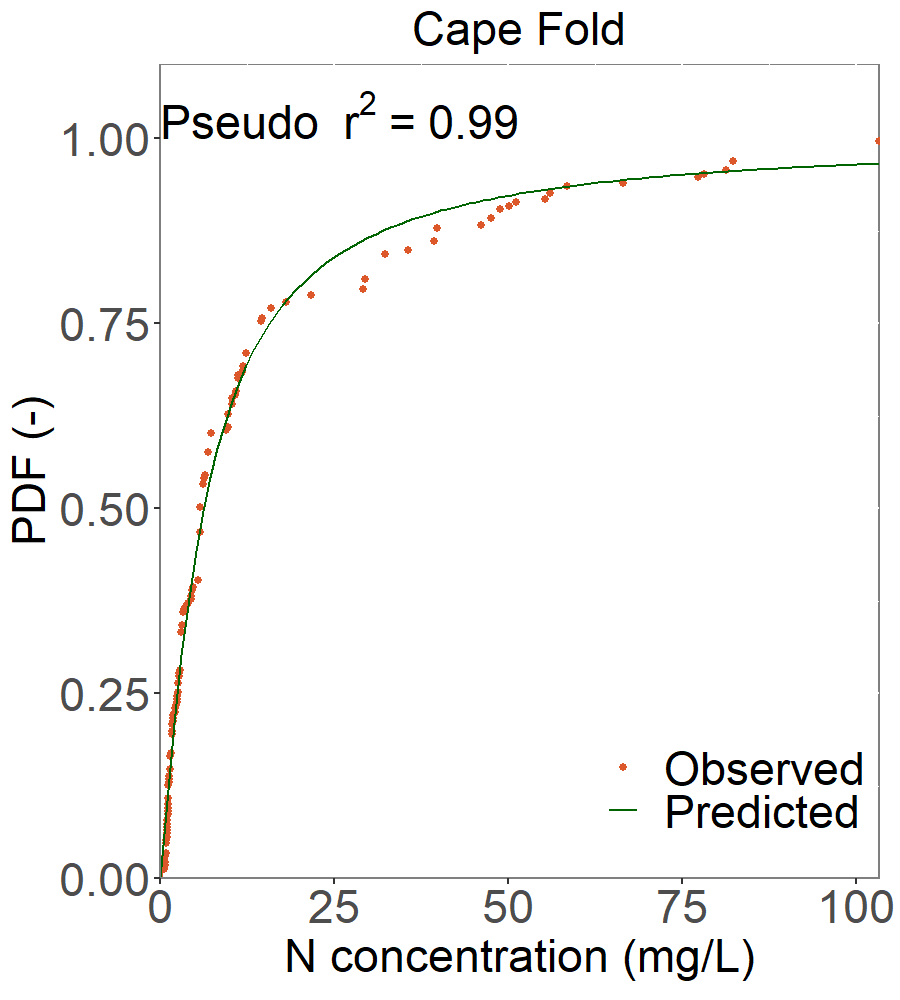

Supplement: Supplementary file 2 — es2c09333_si_002.zip [file es2c09333_si_002.zip › SSD_Ecoregion/Cape Fold.tif]

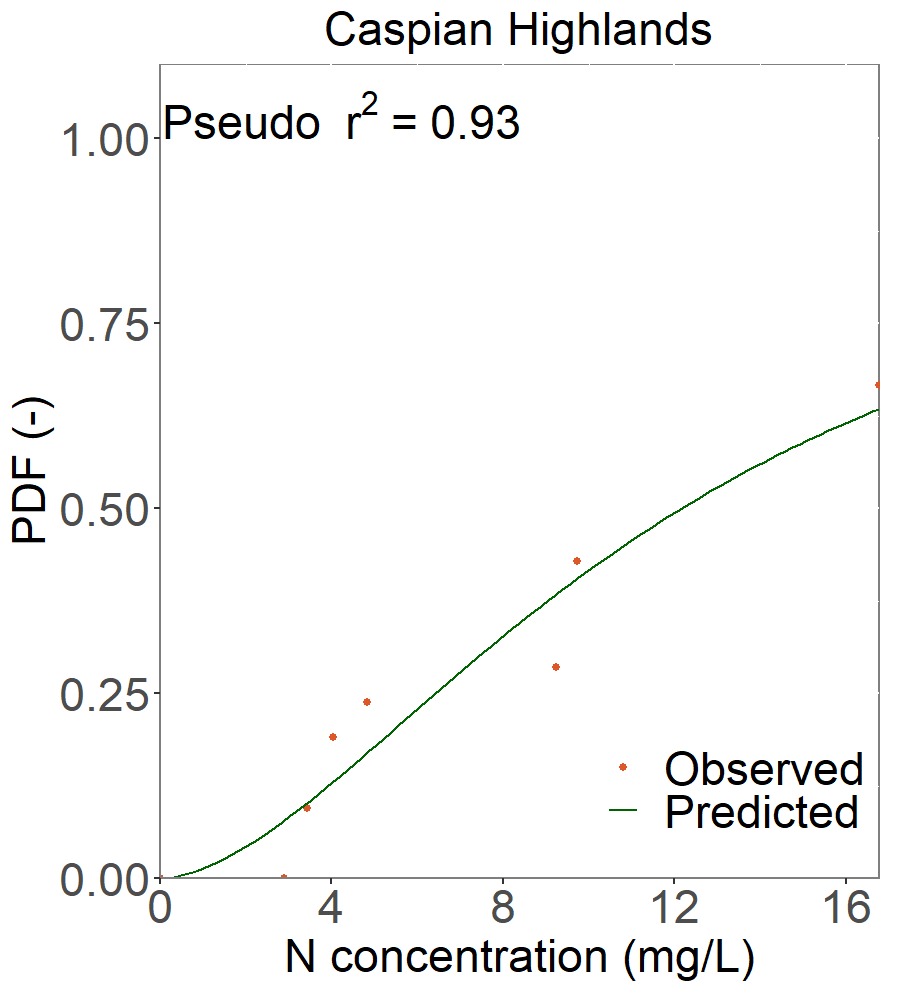

Supplement: Supplementary file 2 — es2c09333_si_002.zip [file es2c09333_si_002.zip › SSD_Ecoregion/Caspian Highlands.tif]

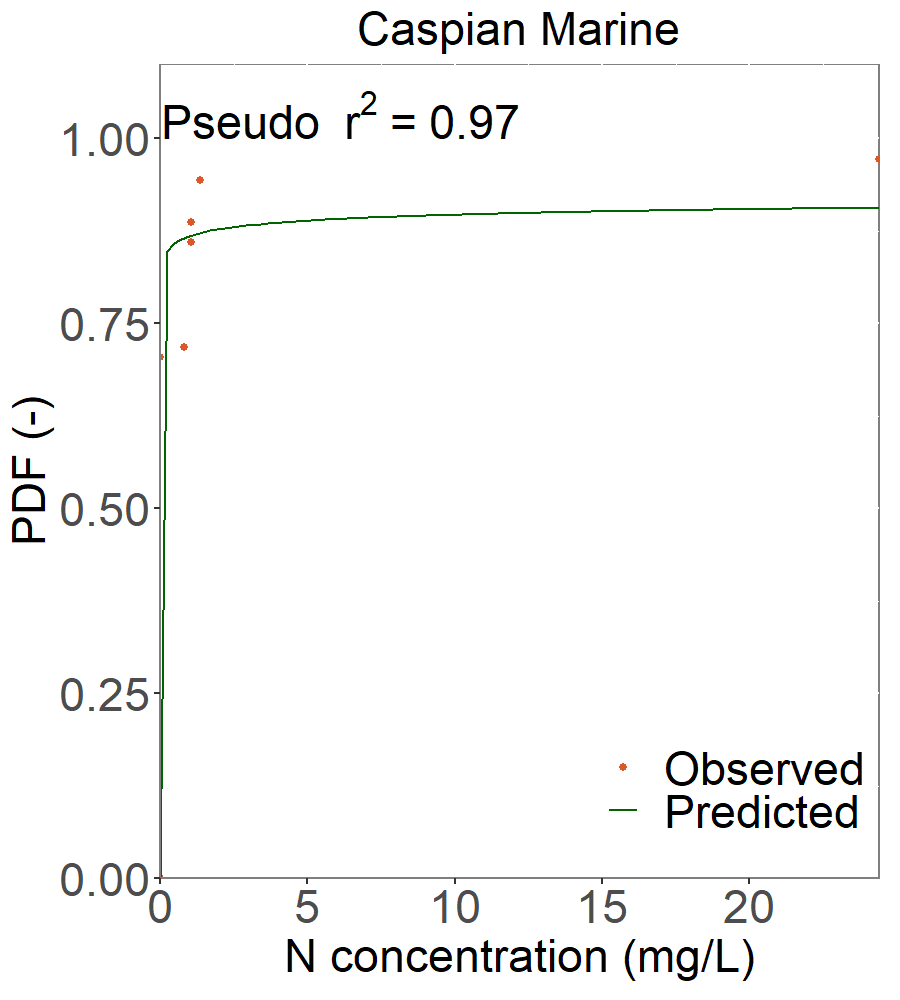

Supplement: Supplementary file 2 — es2c09333_si_002.zip [file es2c09333_si_002.zip › SSD_Ecoregion/Caspian Marine.tif]

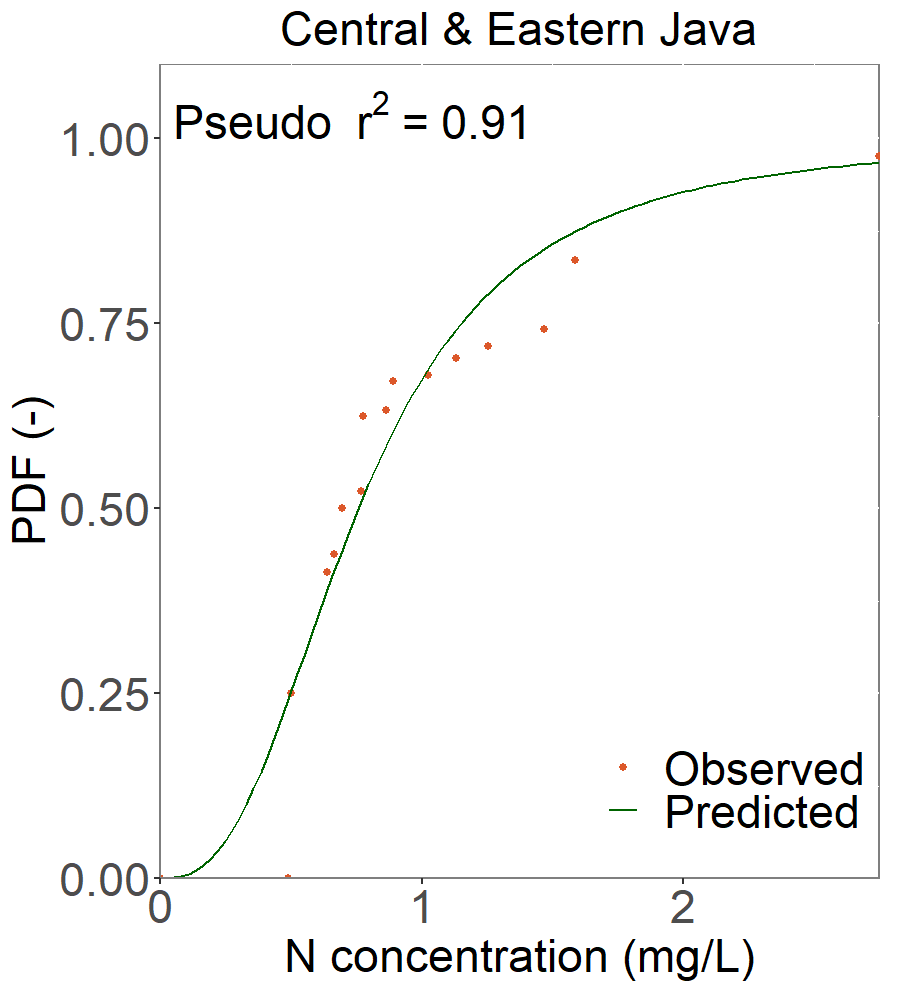

Supplement: Supplementary file 2 — es2c09333_si_002.zip [file es2c09333_si_002.zip › SSD_Ecoregion/Central & Eastern Java.tif]

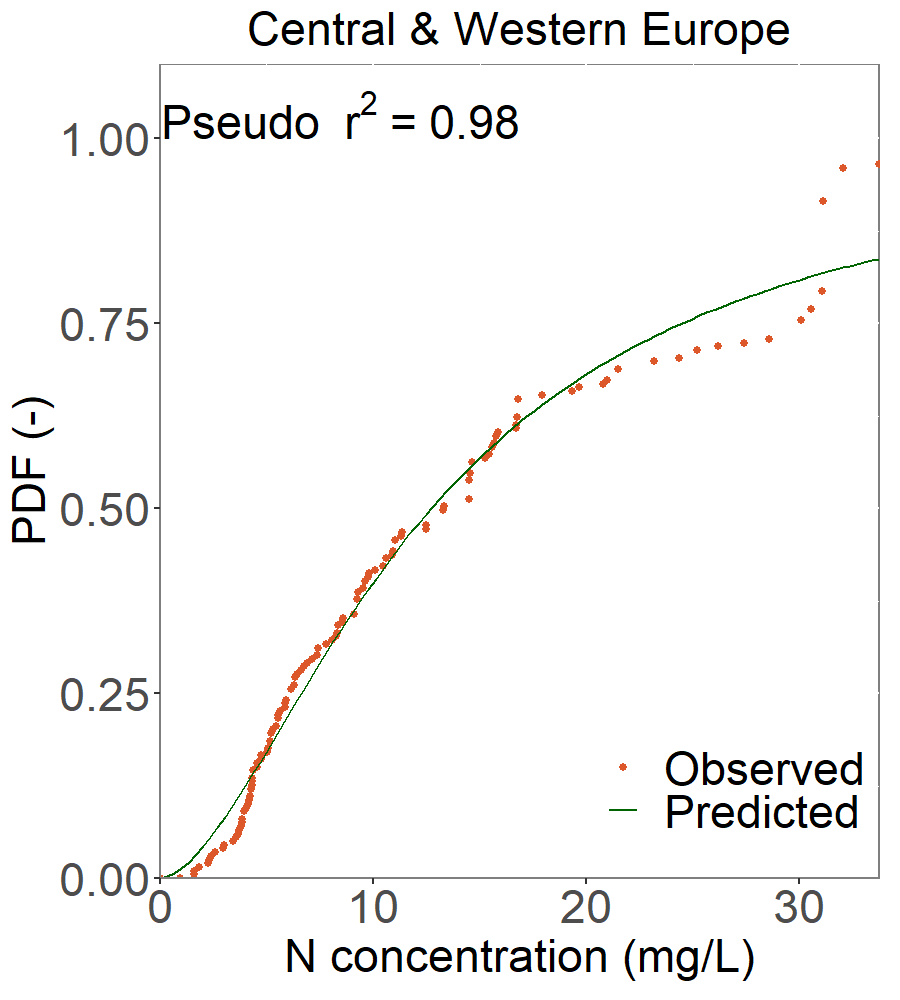

Supplement: Supplementary file 2 — es2c09333_si_002.zip [file es2c09333_si_002.zip › SSD_Ecoregion/Central & Western Europe.tif]

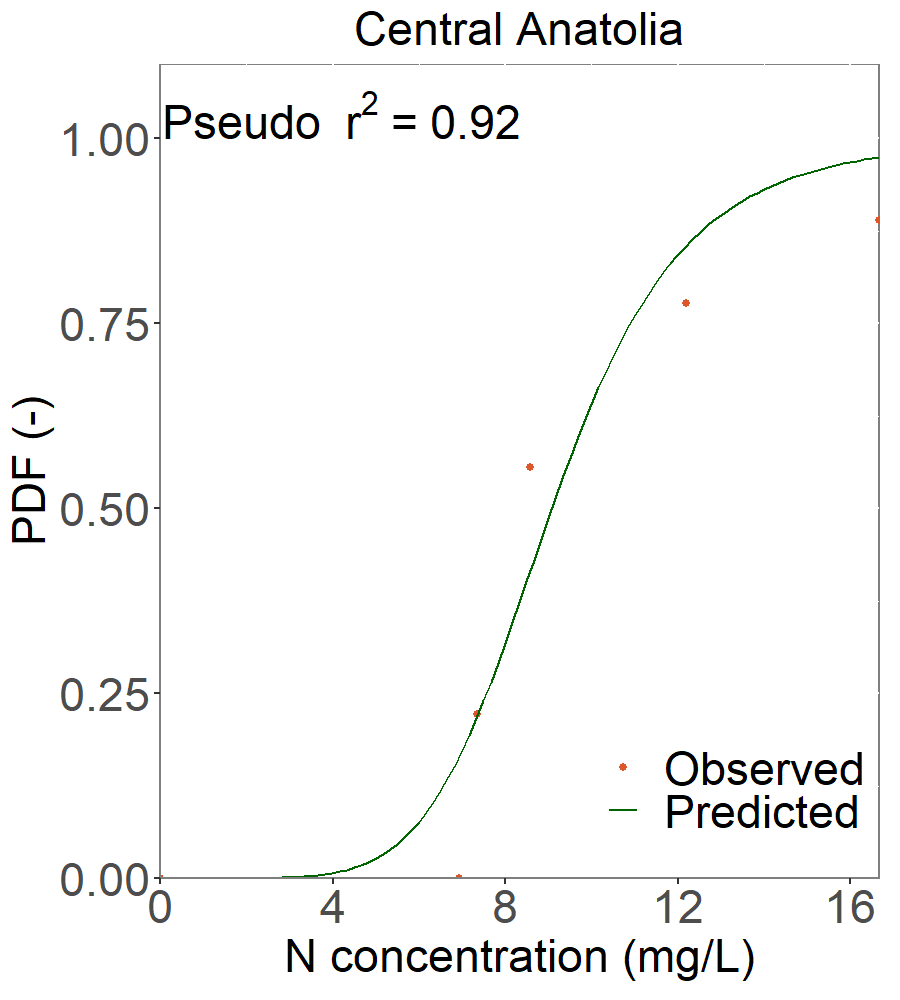

Supplement: Supplementary file 2 — es2c09333_si_002.zip [file es2c09333_si_002.zip › SSD_Ecoregion/Central Anatolia.tif]

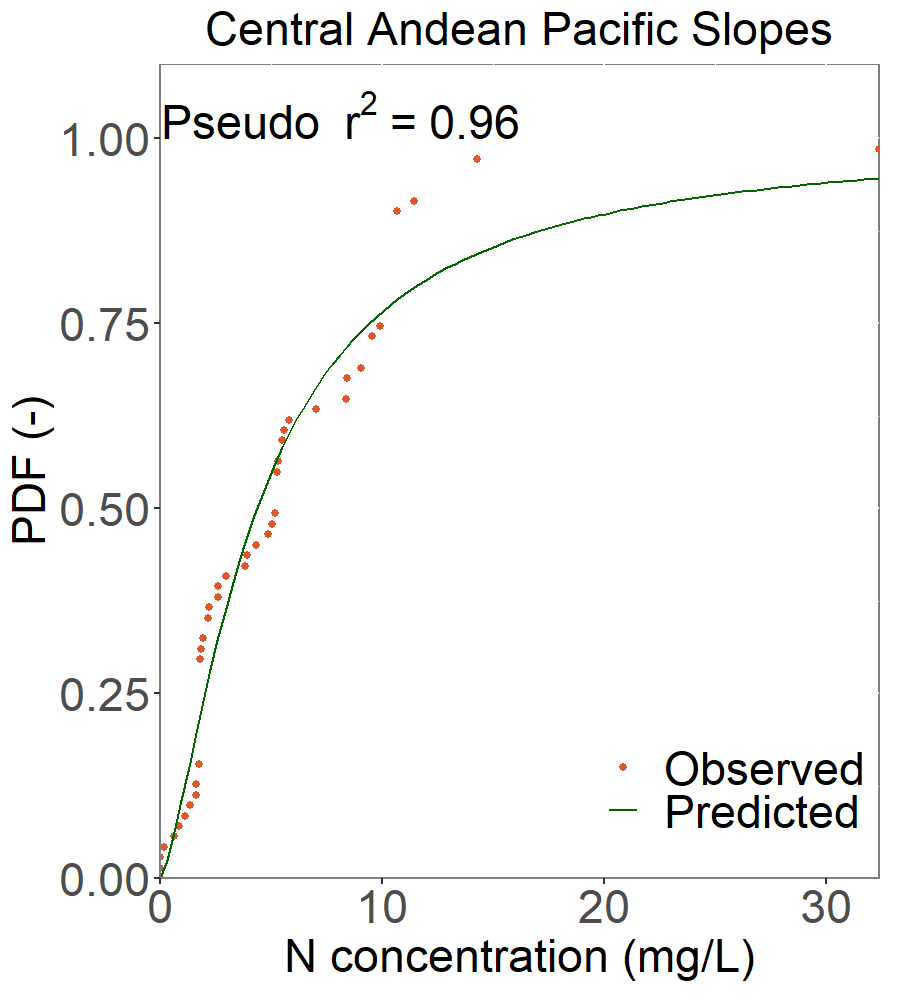

Supplement: Supplementary file 2 — es2c09333_si_002.zip [file es2c09333_si_002.zip › SSD_Ecoregion/Central Andean Pacific Slopes.tif]

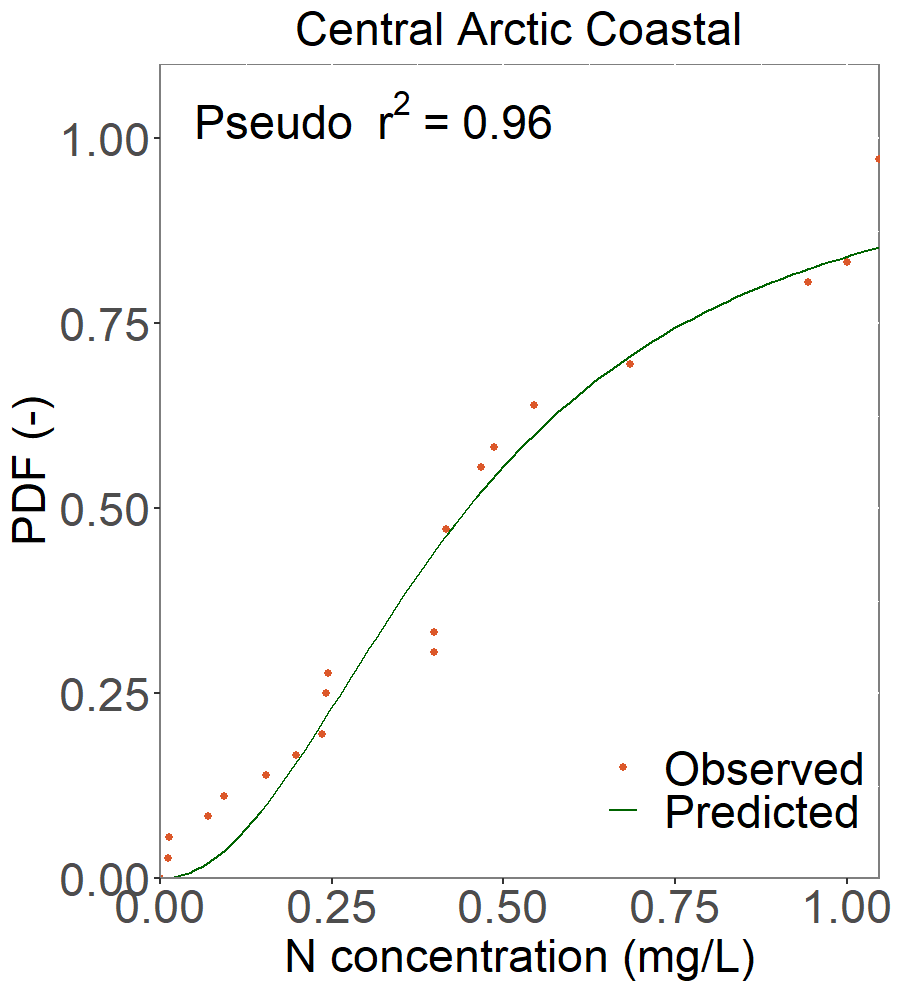

Supplement: Supplementary file 2 — es2c09333_si_002.zip [file es2c09333_si_002.zip › SSD_Ecoregion/Central Arctic Coastal.tif]

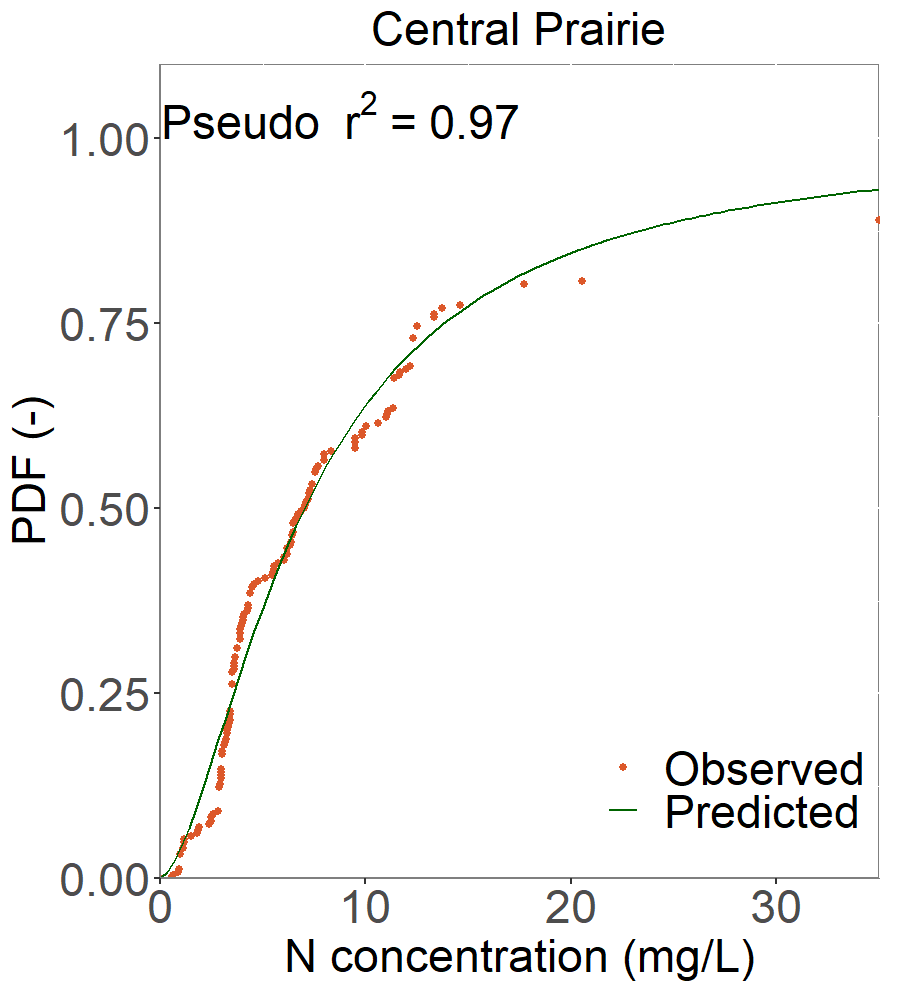

Supplement: Supplementary file 2 — es2c09333_si_002.zip [file es2c09333_si_002.zip › SSD_Ecoregion/Central Prairie.tif]

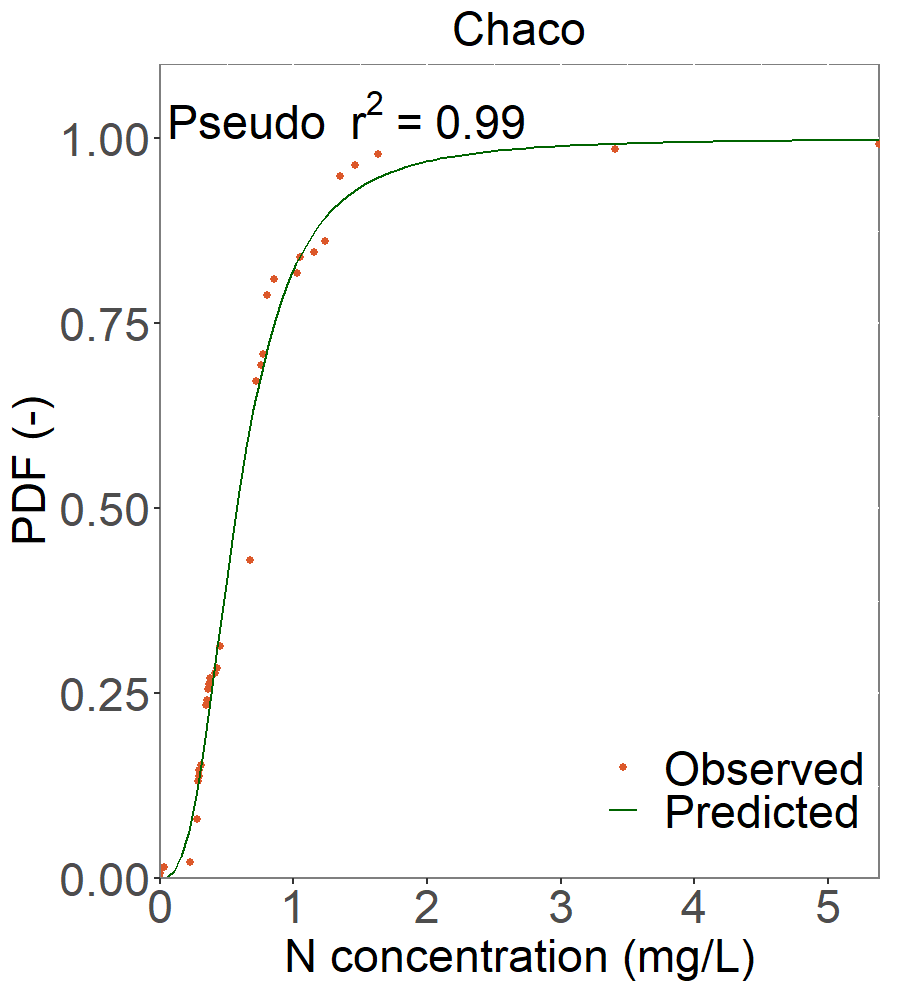

Supplement: Supplementary file 2 — es2c09333_si_002.zip [file es2c09333_si_002.zip › SSD_Ecoregion/Chaco.tif]

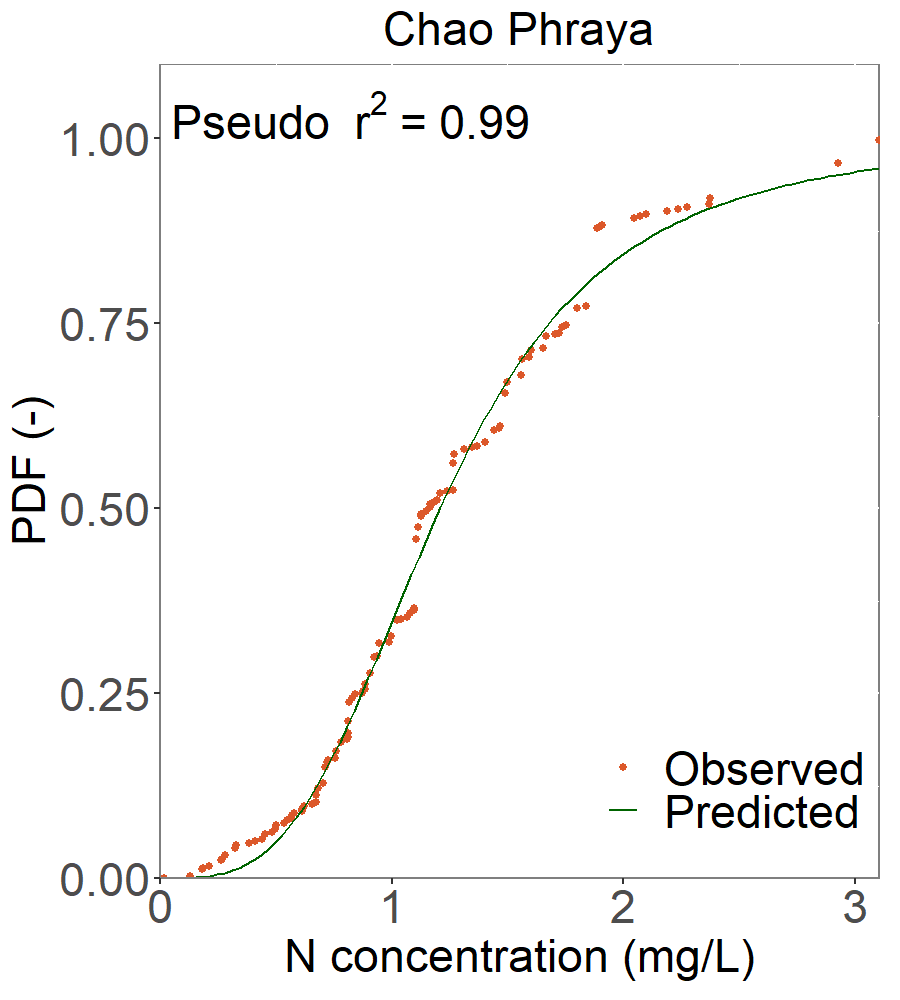

Supplement: Supplementary file 2 — es2c09333_si_002.zip [file es2c09333_si_002.zip › SSD_Ecoregion/Chao Phraya.tif]

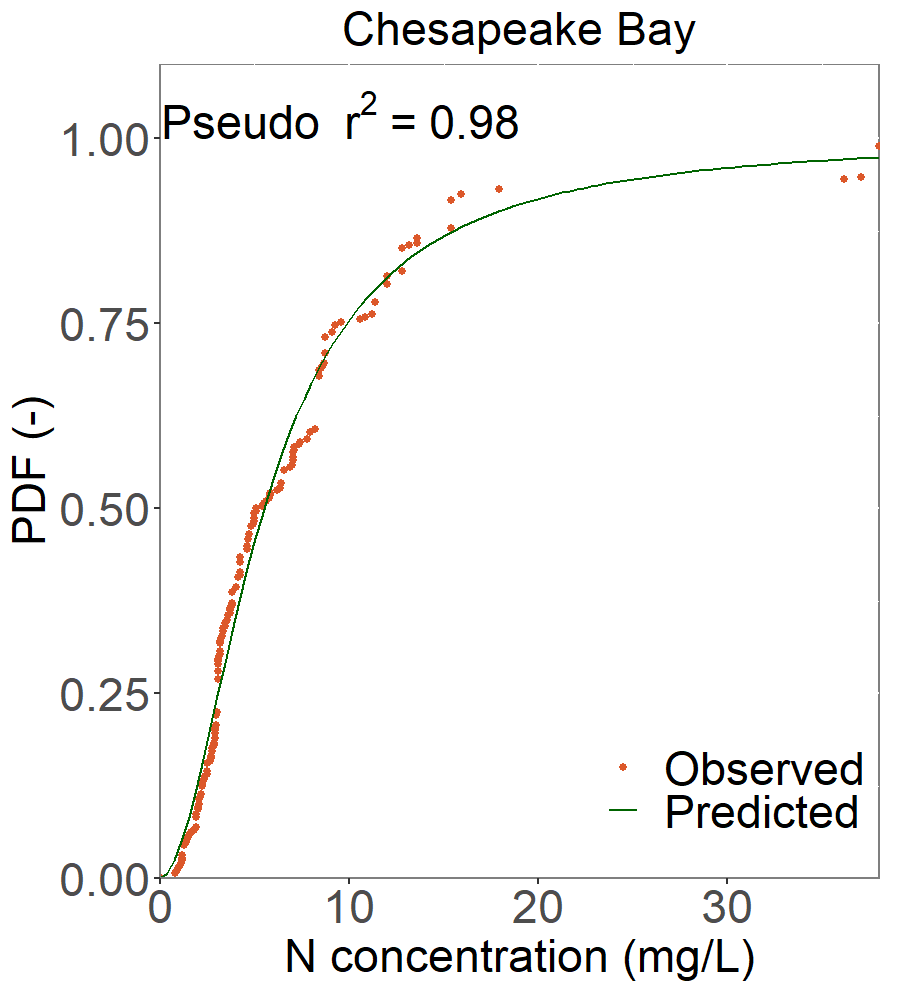

Supplement: Supplementary file 2 — es2c09333_si_002.zip [file es2c09333_si_002.zip › SSD_Ecoregion/Chesapeake Bay.tif]

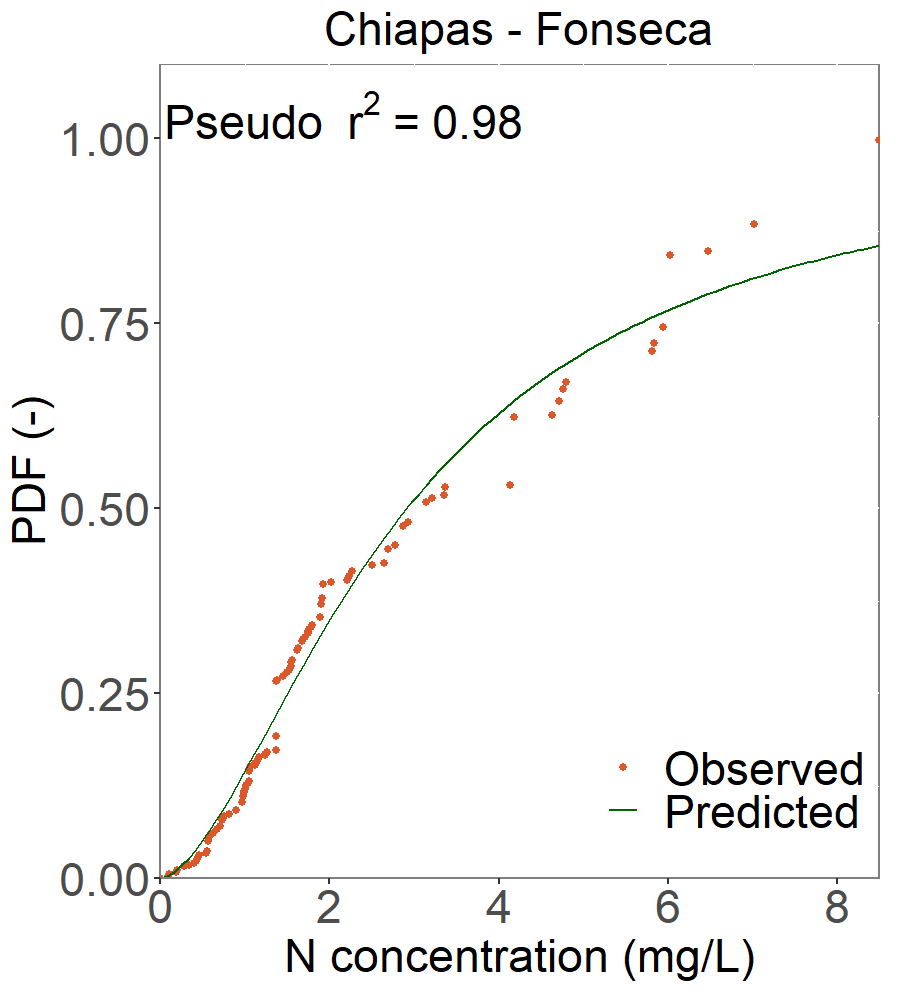

Supplement: Supplementary file 2 — es2c09333_si_002.zip [file es2c09333_si_002.zip › SSD_Ecoregion/Chiapas - Fonseca.tif]

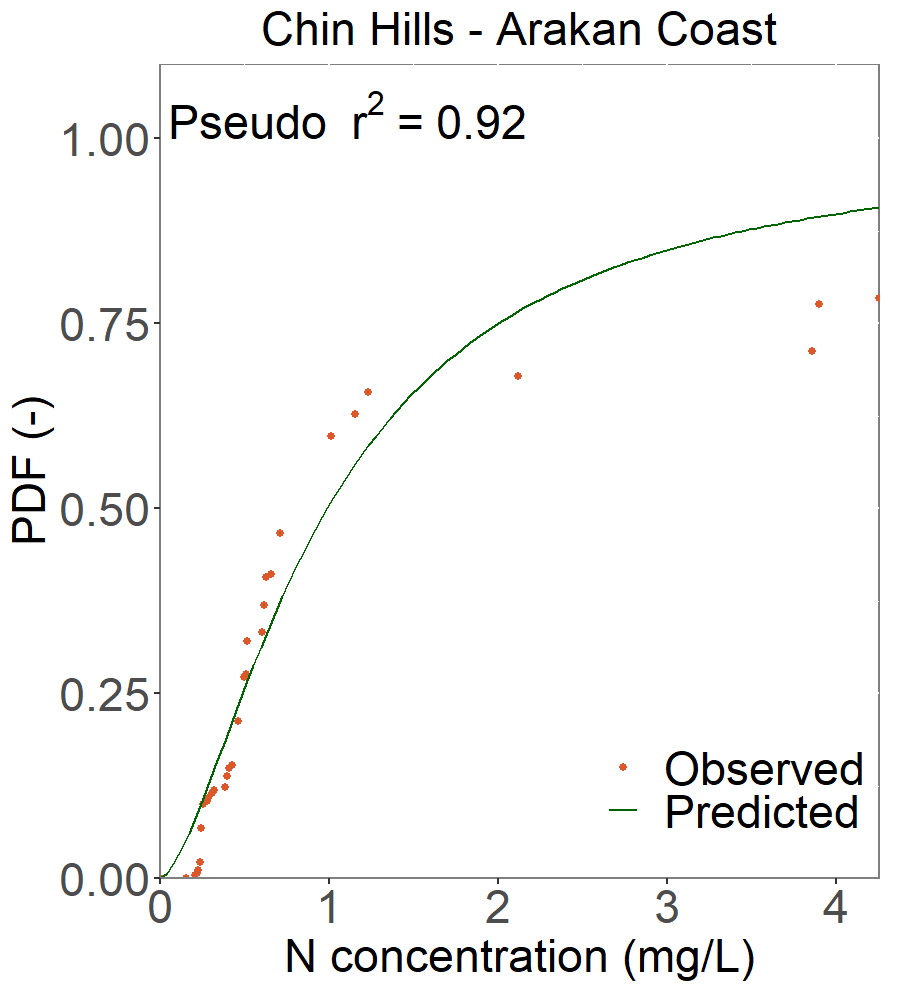

Supplement: Supplementary file 2 — es2c09333_si_002.zip [file es2c09333_si_002.zip › SSD_Ecoregion/Chin Hills - Arakan Coast.tif]

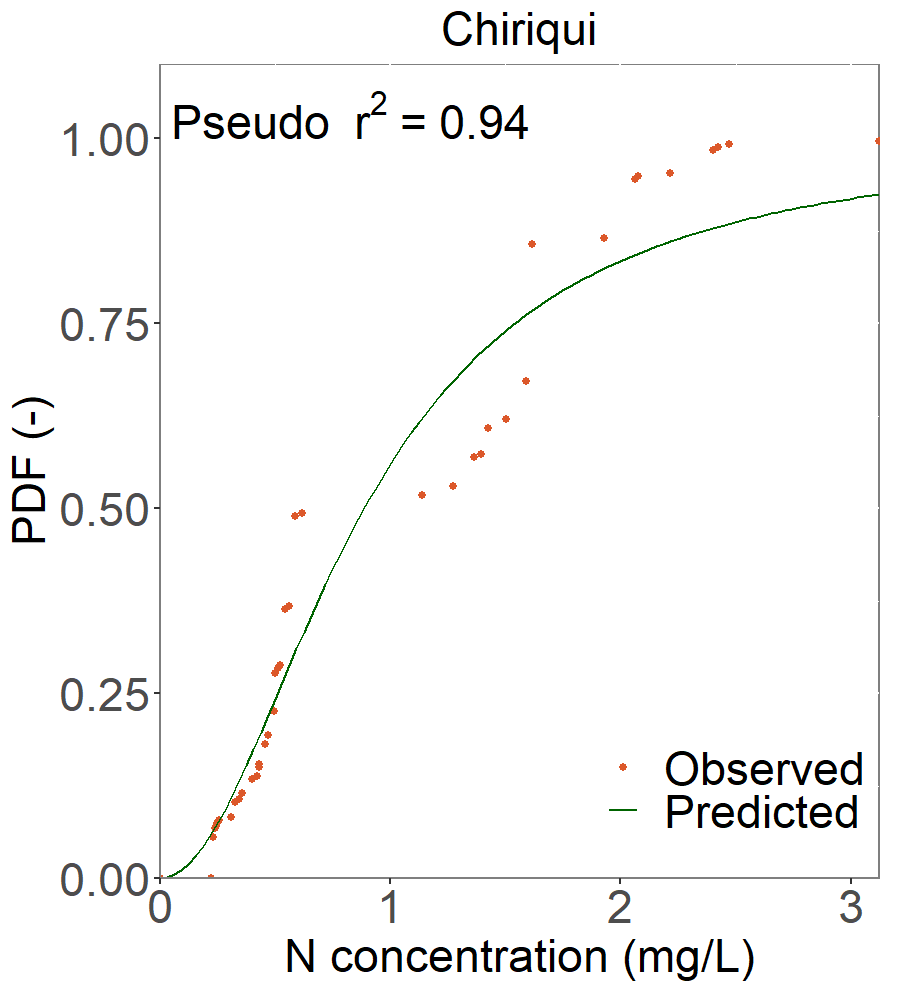

Supplement: Supplementary file 2 — es2c09333_si_002.zip [file es2c09333_si_002.zip › SSD_Ecoregion/Chiriqui.tif]

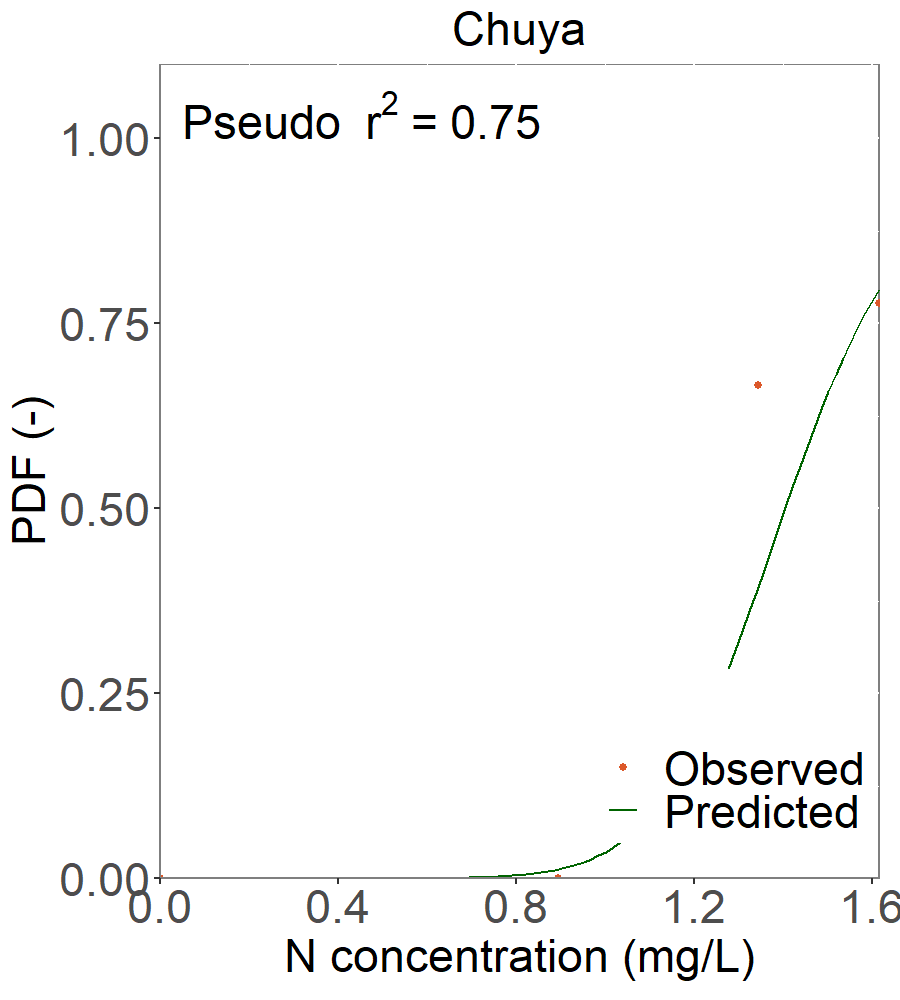

Supplement: Supplementary file 2 — es2c09333_si_002.zip [file es2c09333_si_002.zip › SSD_Ecoregion/Chuya.tif]

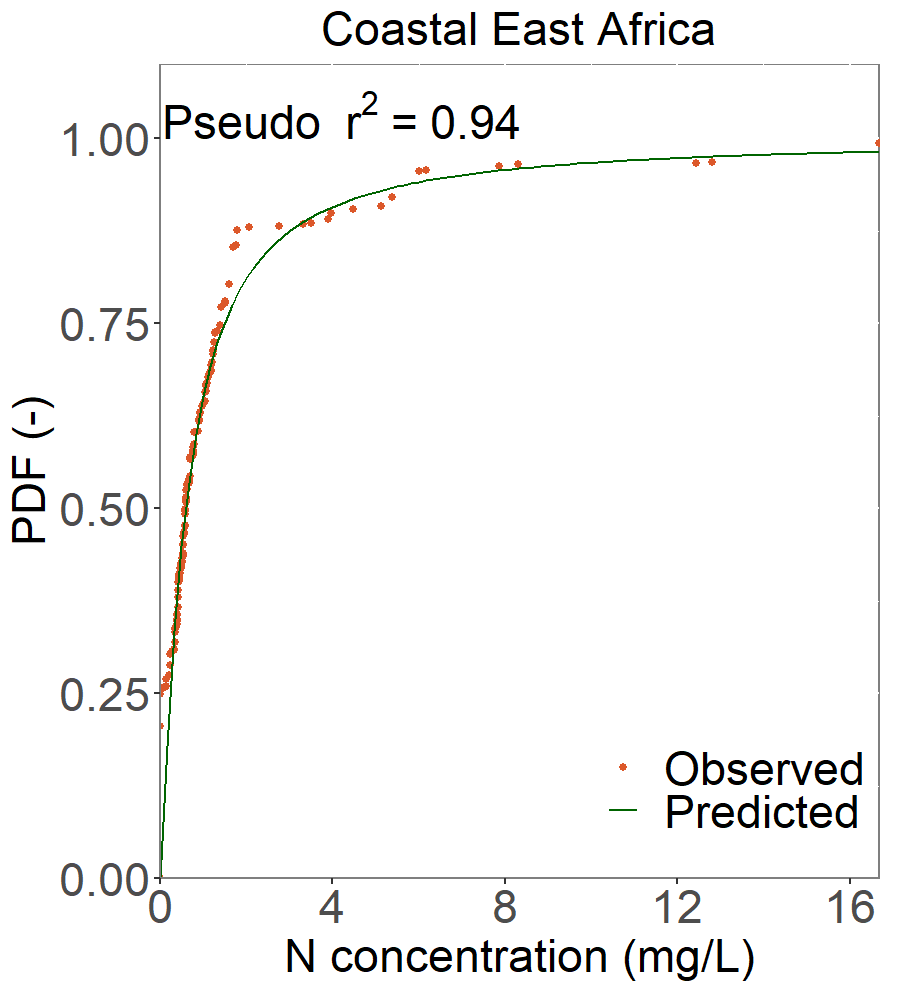

Supplement: Supplementary file 2 — es2c09333_si_002.zip [file es2c09333_si_002.zip › SSD_Ecoregion/Coastal East Africa.tif]

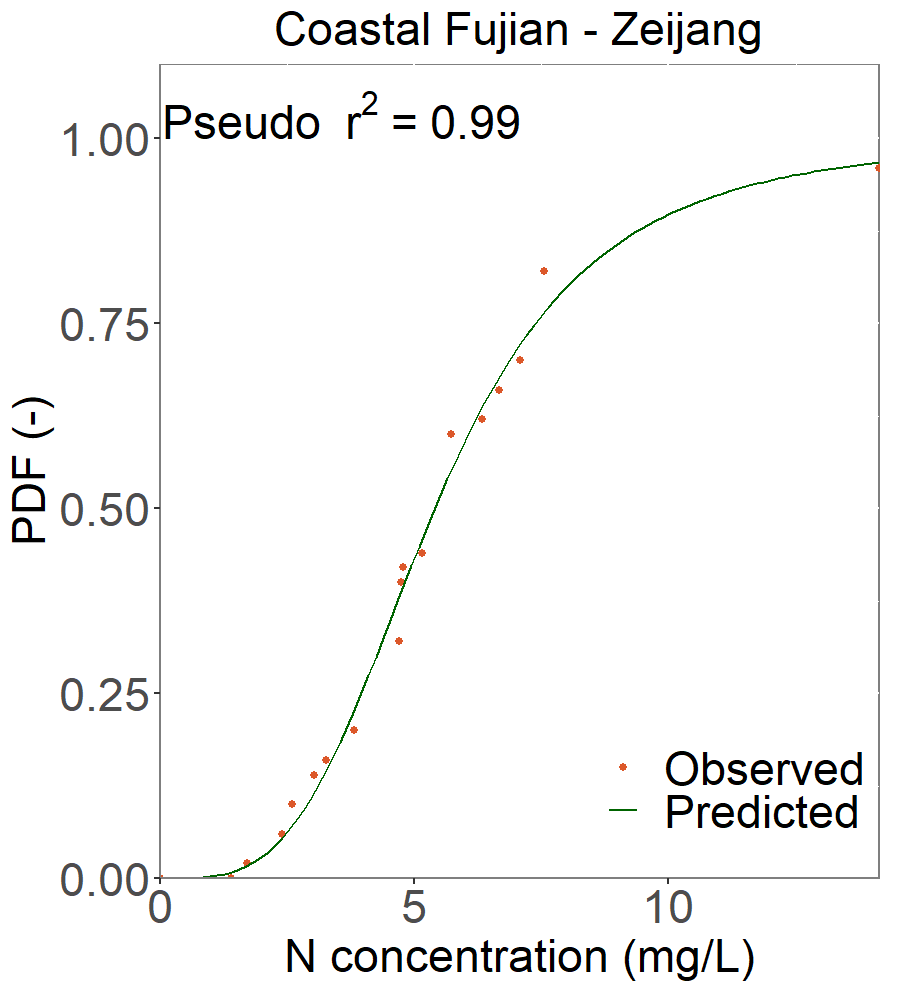

Supplement: Supplementary file 2 — es2c09333_si_002.zip [file es2c09333_si_002.zip › SSD_Ecoregion/Coastal Fujian - Zeijang.tif]

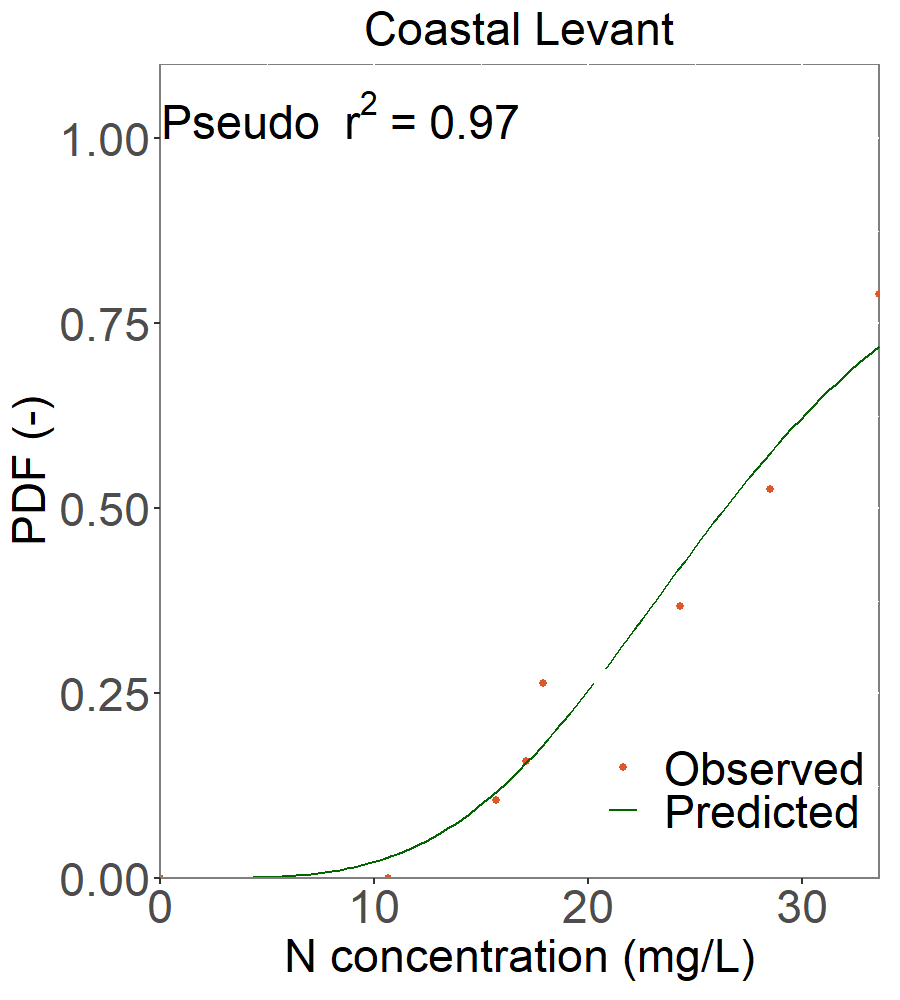

Supplement: Supplementary file 2 — es2c09333_si_002.zip [file es2c09333_si_002.zip › SSD_Ecoregion/Coastal Levant.tif]

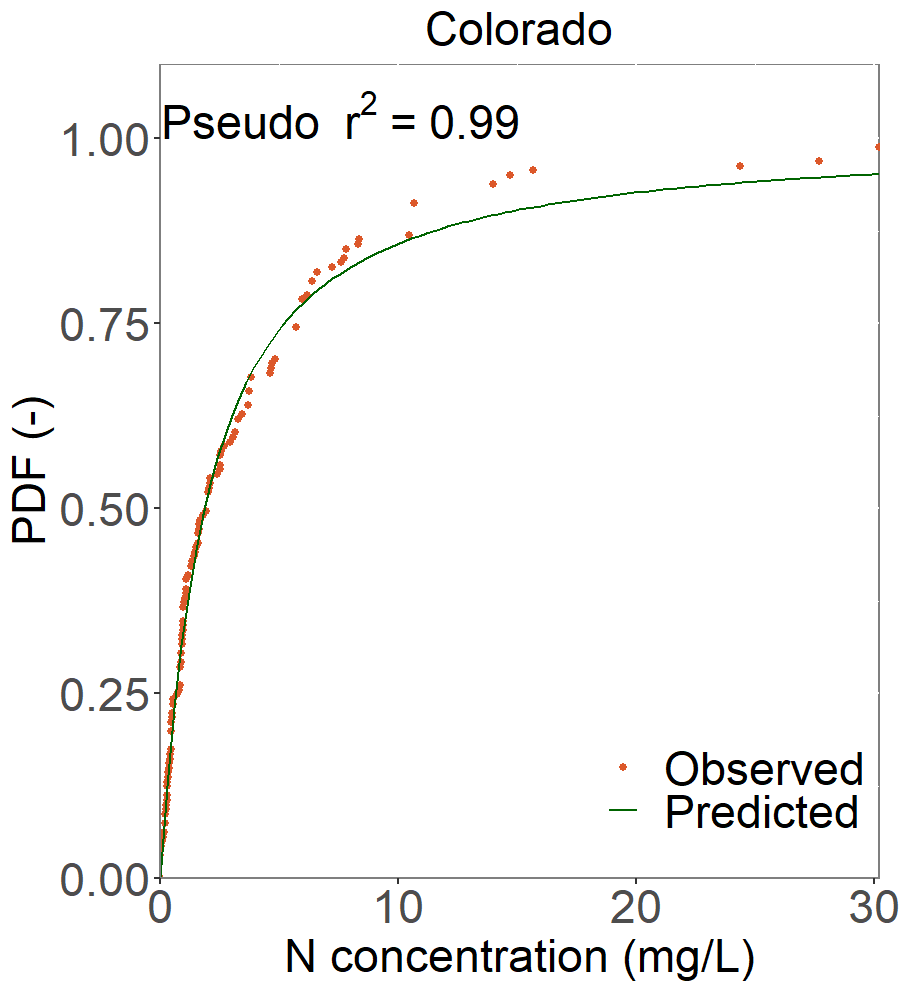

Supplement: Supplementary file 2 — es2c09333_si_002.zip [file es2c09333_si_002.zip › SSD_Ecoregion/Colorado.tif]

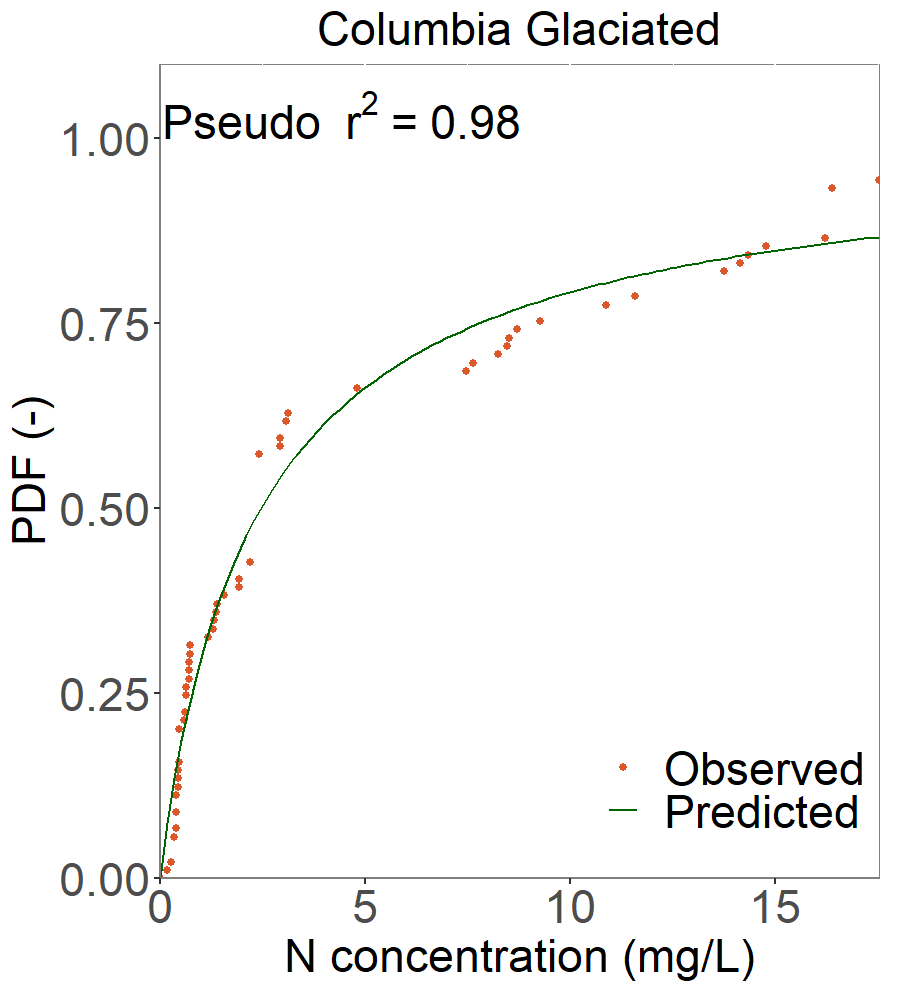

Supplement: Supplementary file 2 — es2c09333_si_002.zip [file es2c09333_si_002.zip › SSD_Ecoregion/Columbia Glaciated.tif]

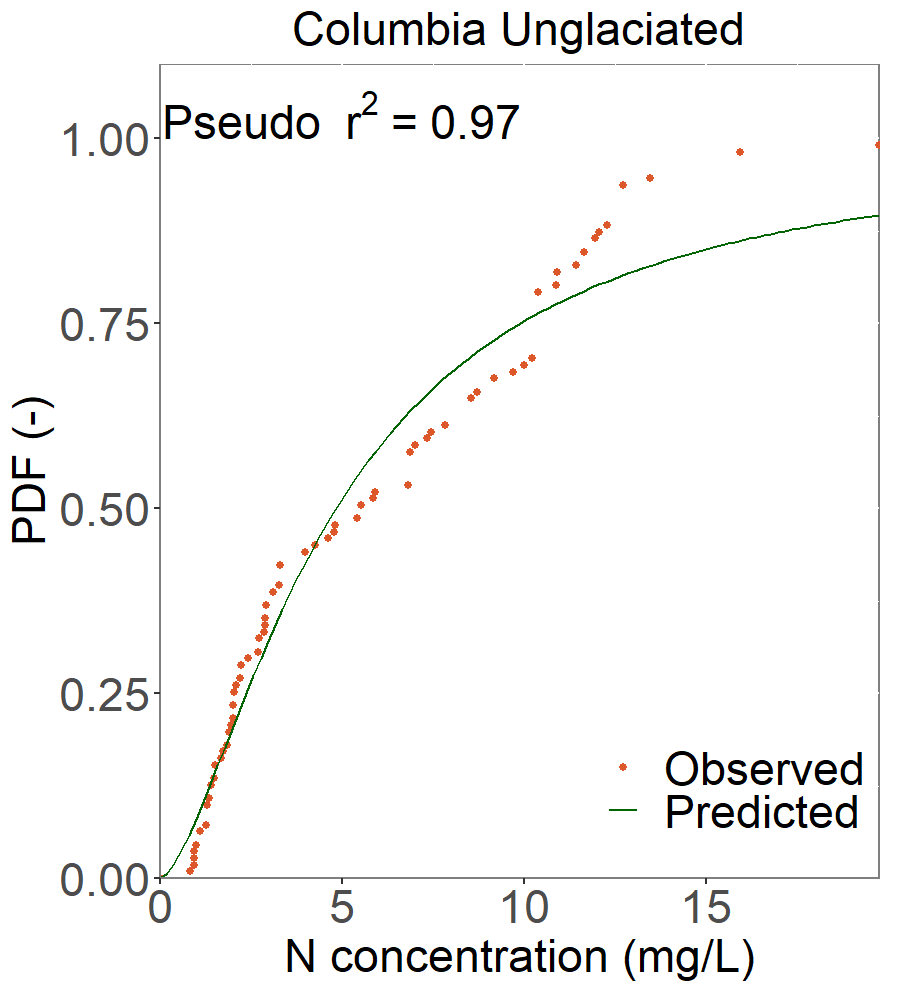

Supplement: Supplementary file 2 — es2c09333_si_002.zip [file es2c09333_si_002.zip › SSD_Ecoregion/Columbia Unglaciated.tif]

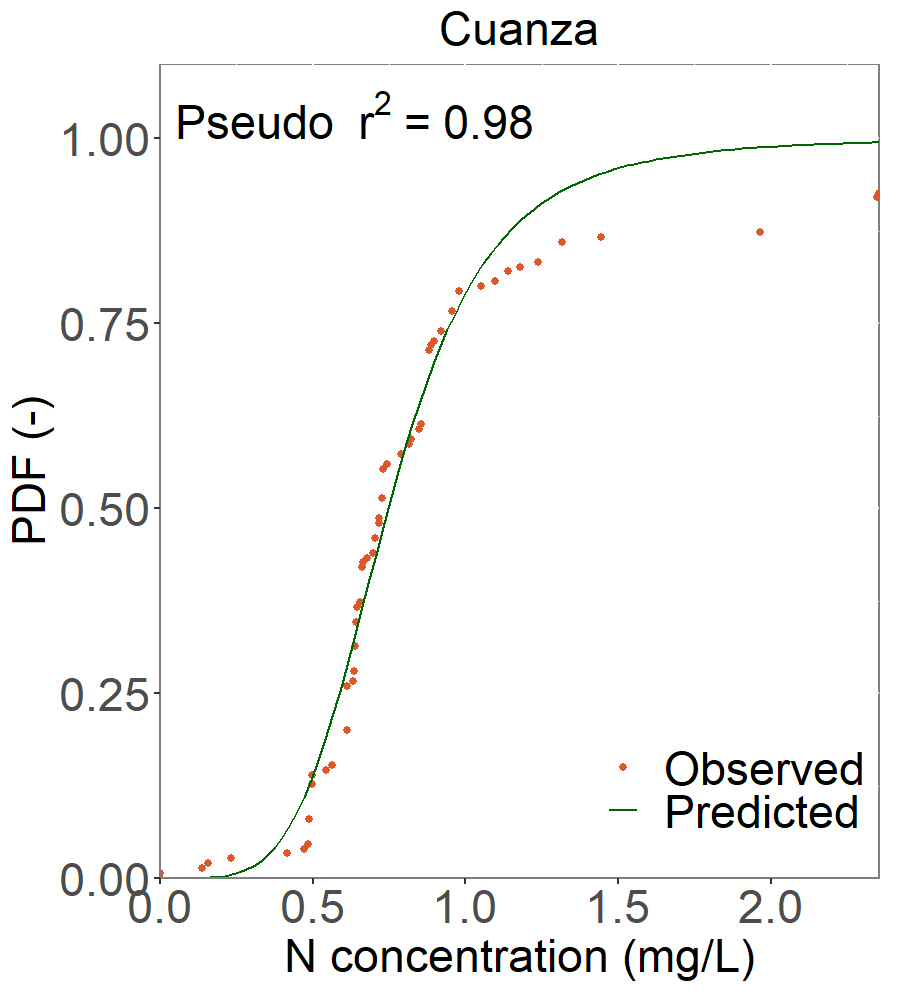

Supplement: Supplementary file 2 — es2c09333_si_002.zip [file es2c09333_si_002.zip › SSD_Ecoregion/Cuanza.tif]

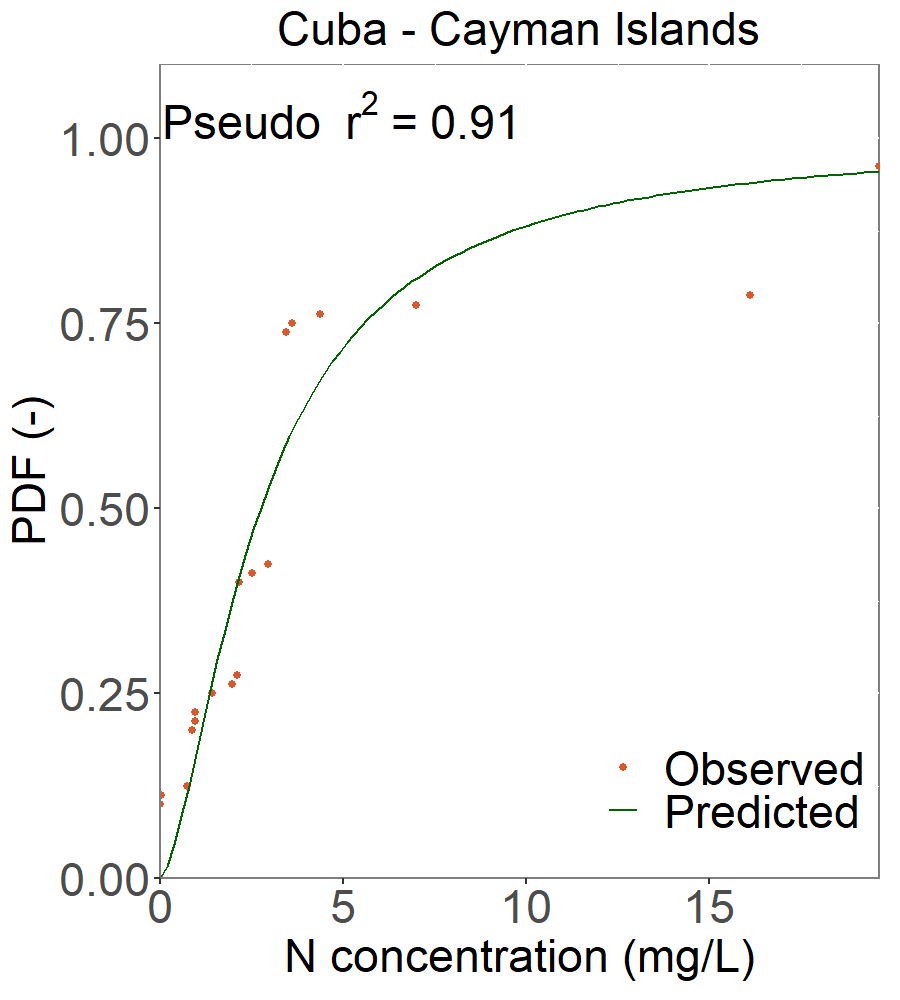

Supplement: Supplementary file 2 — es2c09333_si_002.zip [file es2c09333_si_002.zip › SSD_Ecoregion/Cuba - Cayman Islands.tif]

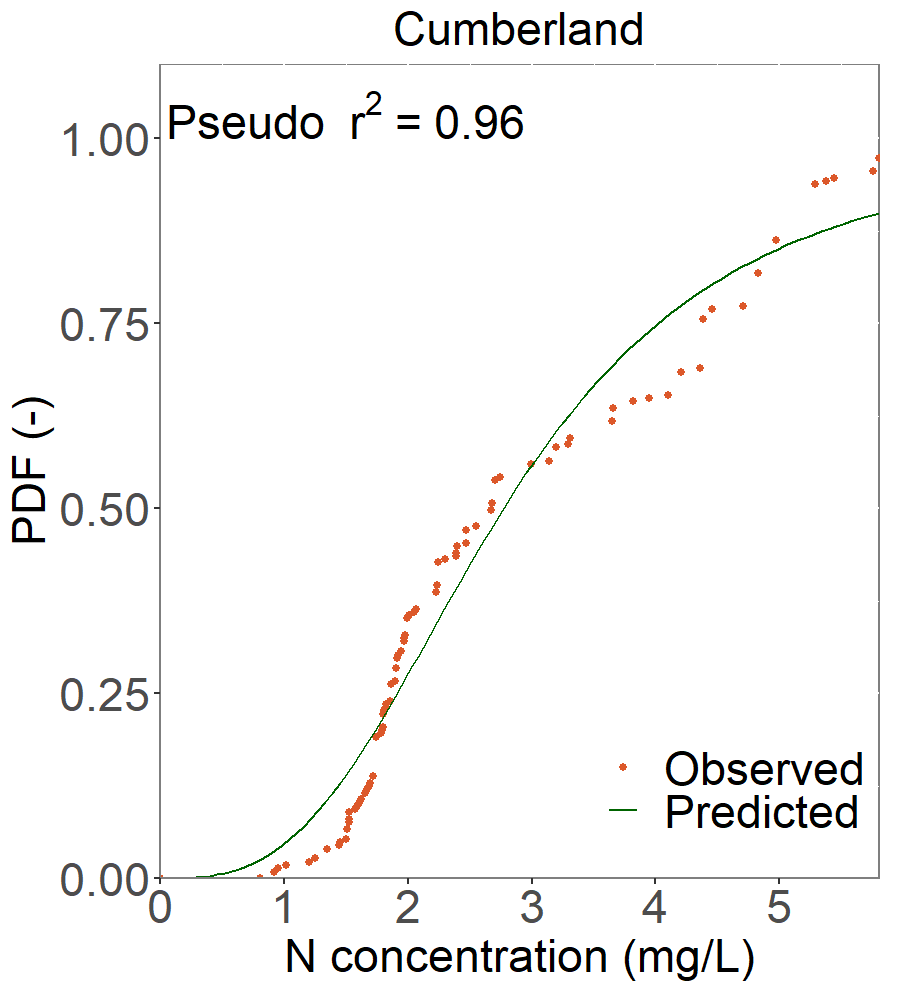

Supplement: Supplementary file 2 — es2c09333_si_002.zip [file es2c09333_si_002.zip › SSD_Ecoregion/Cumberland.tif]

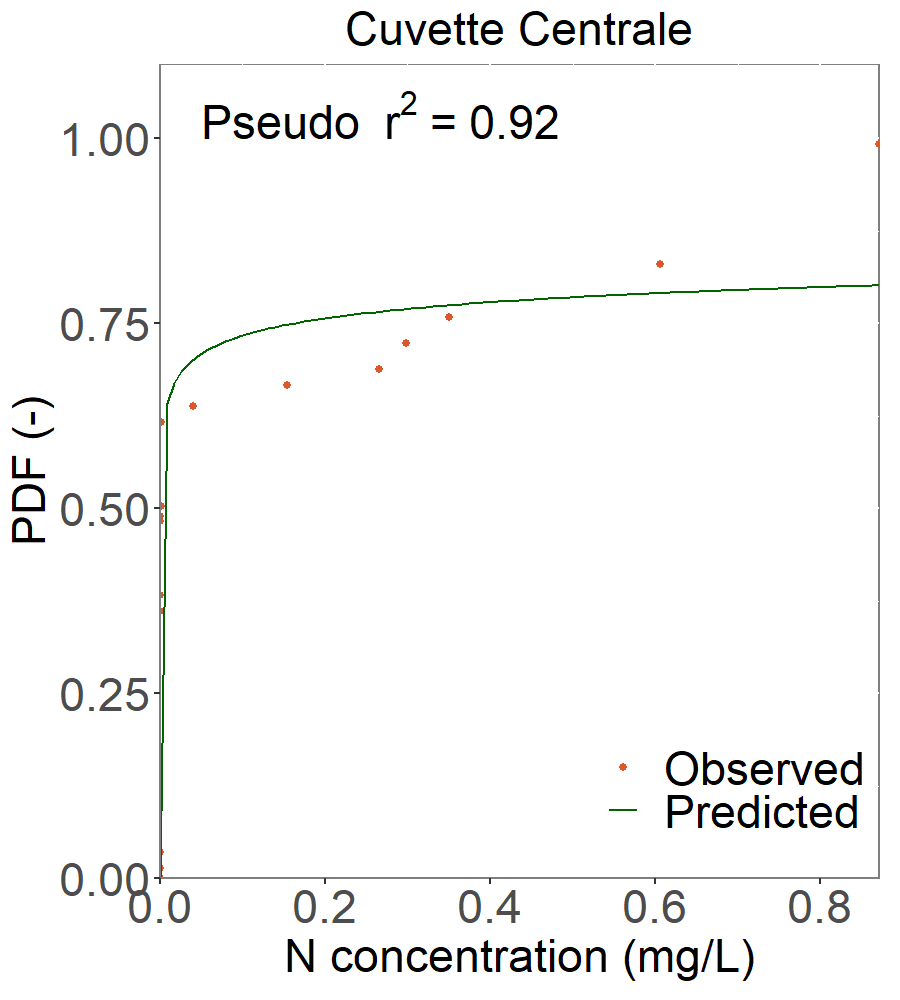

Supplement: Supplementary file 2 — es2c09333_si_002.zip [file es2c09333_si_002.zip › SSD_Ecoregion/Cuvette Centrale.tif]

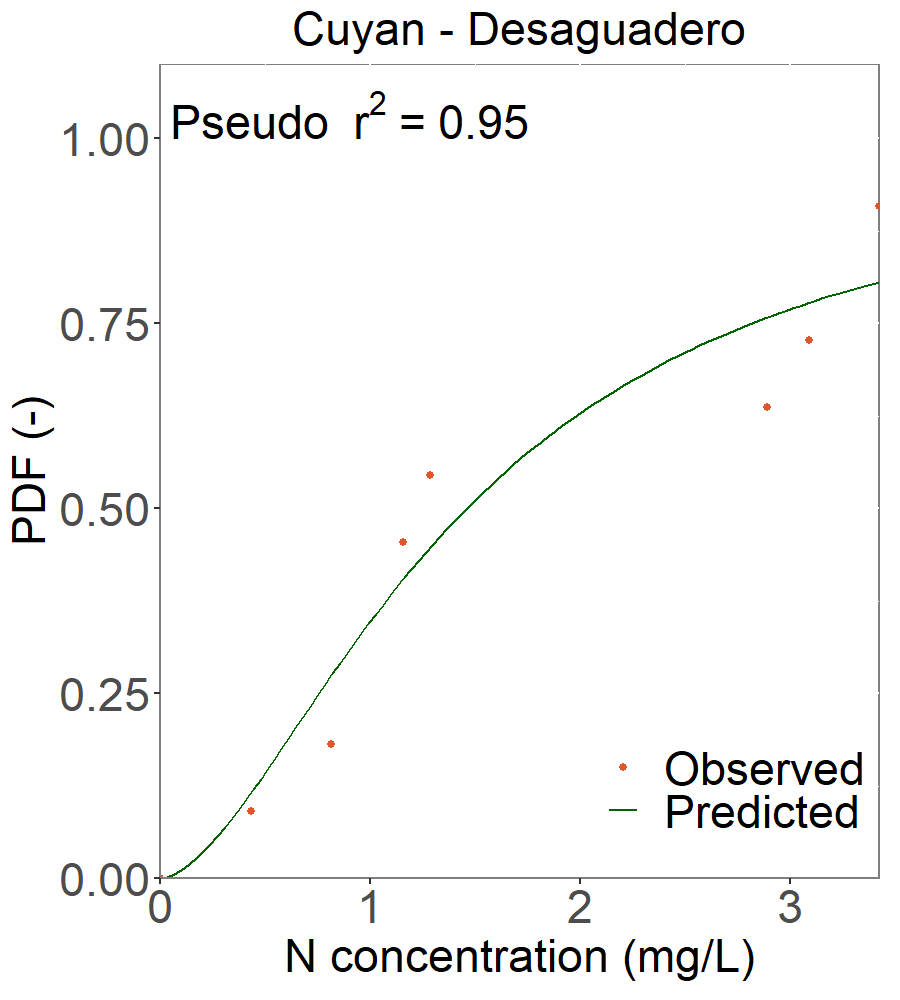

Supplement: Supplementary file 2 — es2c09333_si_002.zip [file es2c09333_si_002.zip › SSD_Ecoregion/Cuyan - Desaguadero.tif]

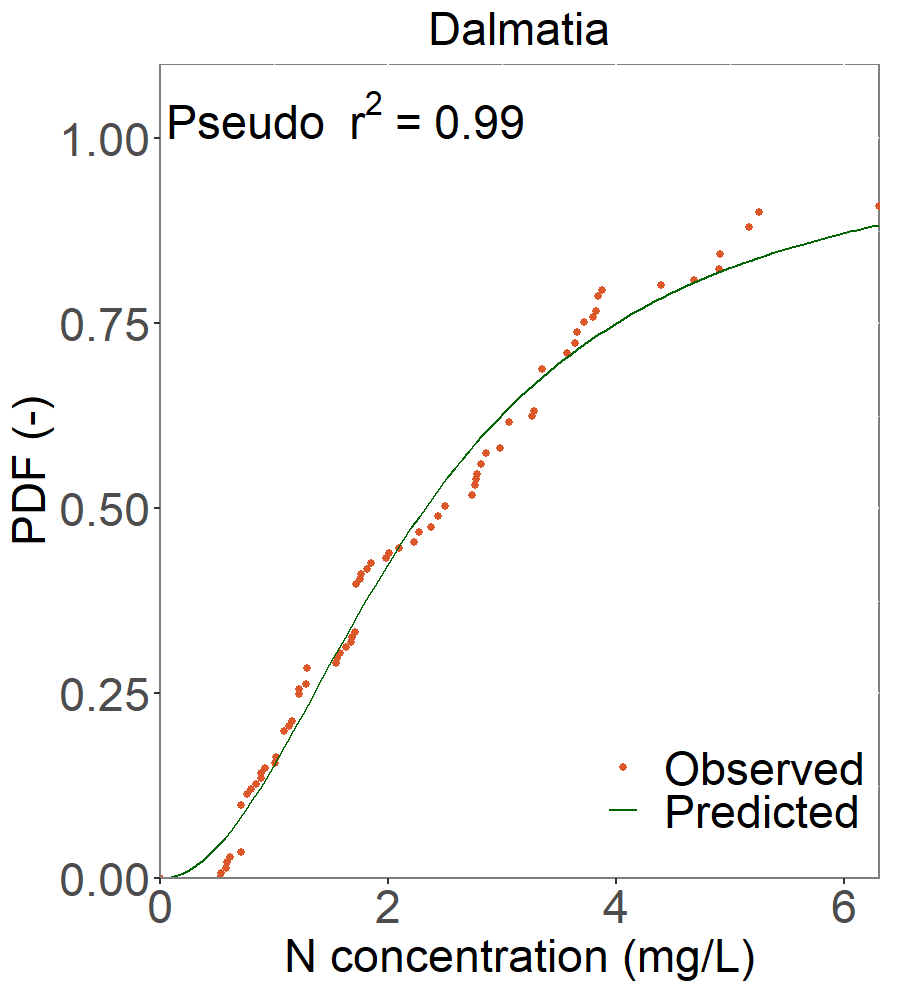

Supplement: Supplementary file 2 — es2c09333_si_002.zip [file es2c09333_si_002.zip › SSD_Ecoregion/Dalmatia.tif]

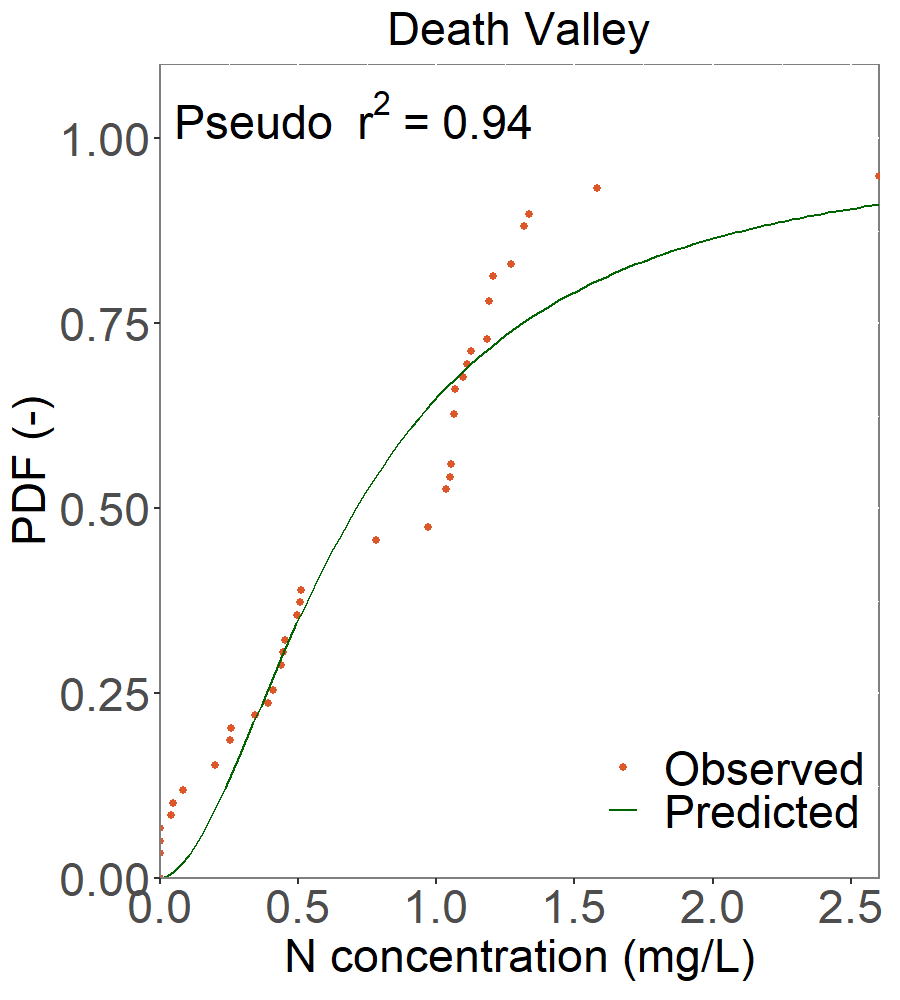

Supplement: Supplementary file 2 — es2c09333_si_002.zip [file es2c09333_si_002.zip › SSD_Ecoregion/Death Valley.tif]

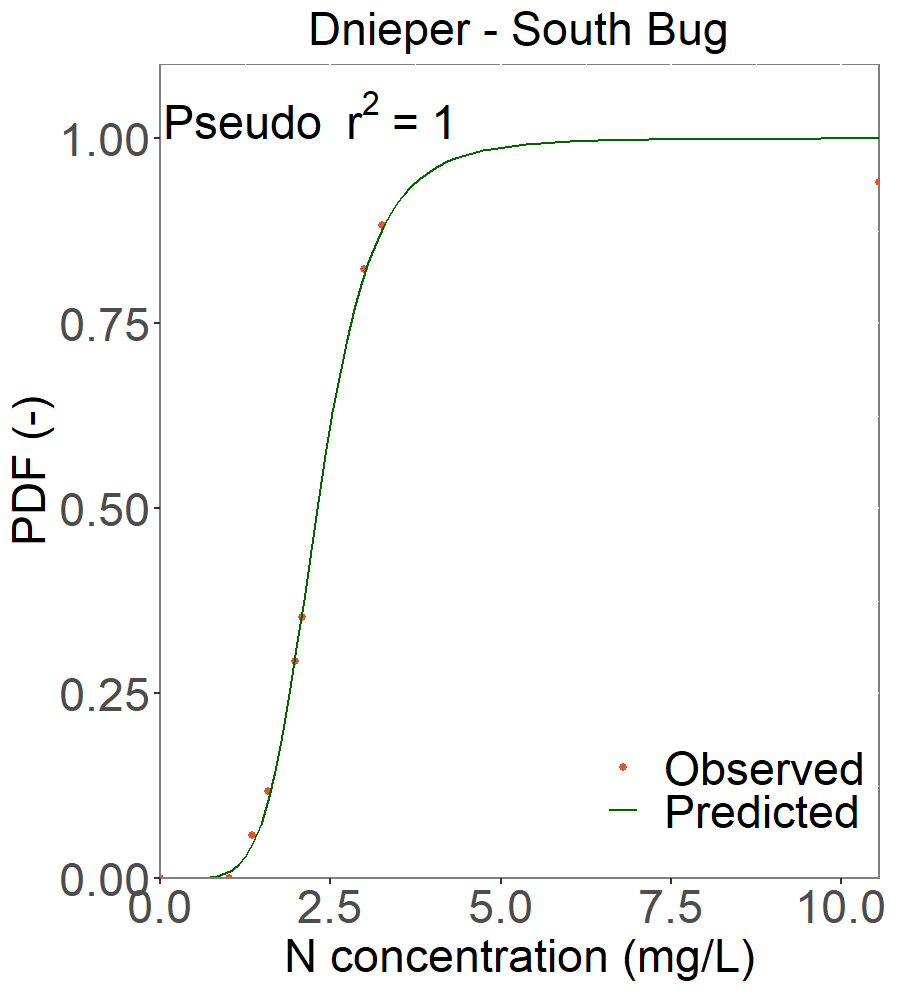

Supplement: Supplementary file 2 — es2c09333_si_002.zip [file es2c09333_si_002.zip › SSD_Ecoregion/Dnieper - South Bug.tif]

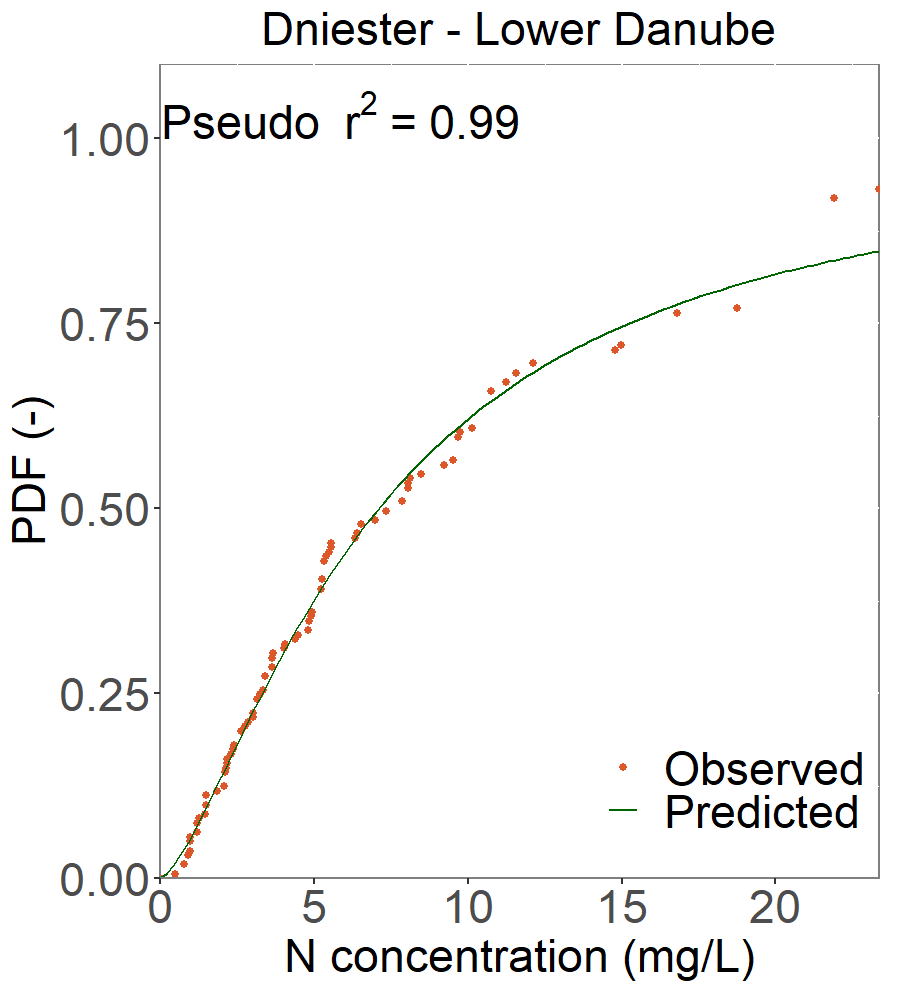

Supplement: Supplementary file 2 — es2c09333_si_002.zip [file es2c09333_si_002.zip › SSD_Ecoregion/Dniester - Lower Danube.tif]

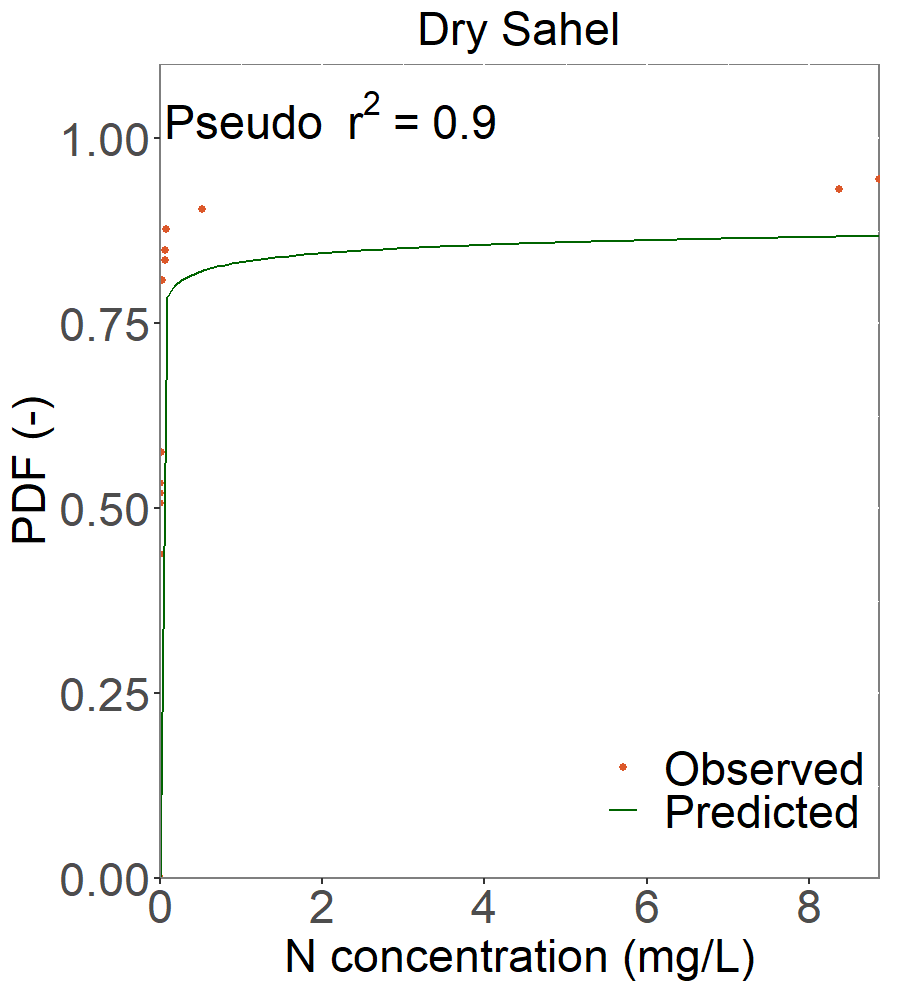

Supplement: Supplementary file 2 — es2c09333_si_002.zip [file es2c09333_si_002.zip › SSD_Ecoregion/Dry Sahel.tif]

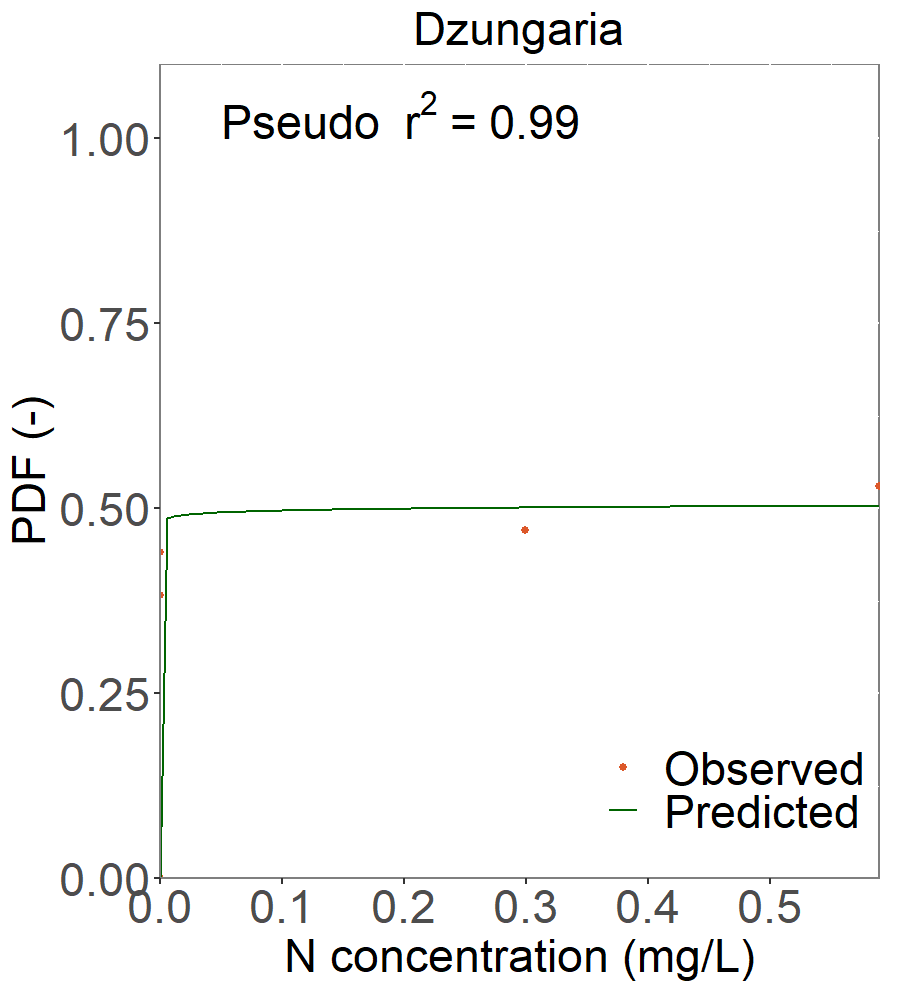

Supplement: Supplementary file 2 — es2c09333_si_002.zip [file es2c09333_si_002.zip › SSD_Ecoregion/Dzungaria.tif]

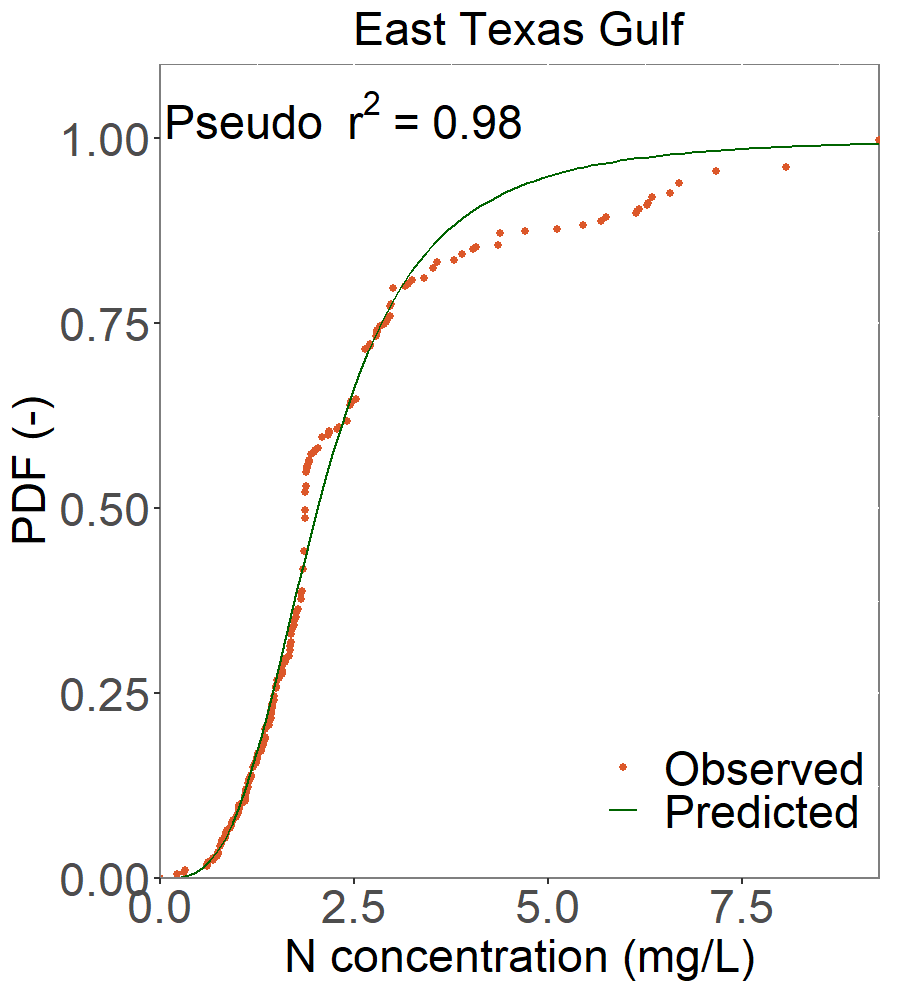

Supplement: Supplementary file 2 — es2c09333_si_002.zip [file es2c09333_si_002.zip › SSD_Ecoregion/East Texas Gulf.tif]

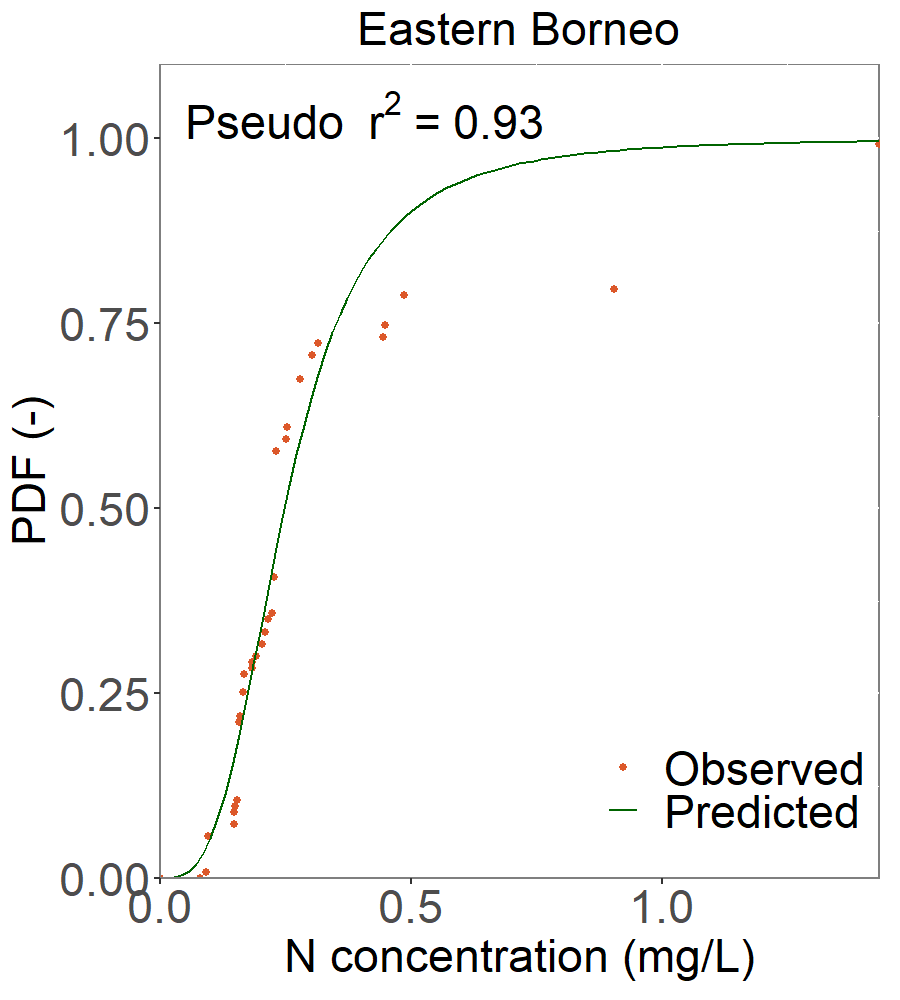

Supplement: Supplementary file 2 — es2c09333_si_002.zip [file es2c09333_si_002.zip › SSD_Ecoregion/Eastern Borneo.tif]

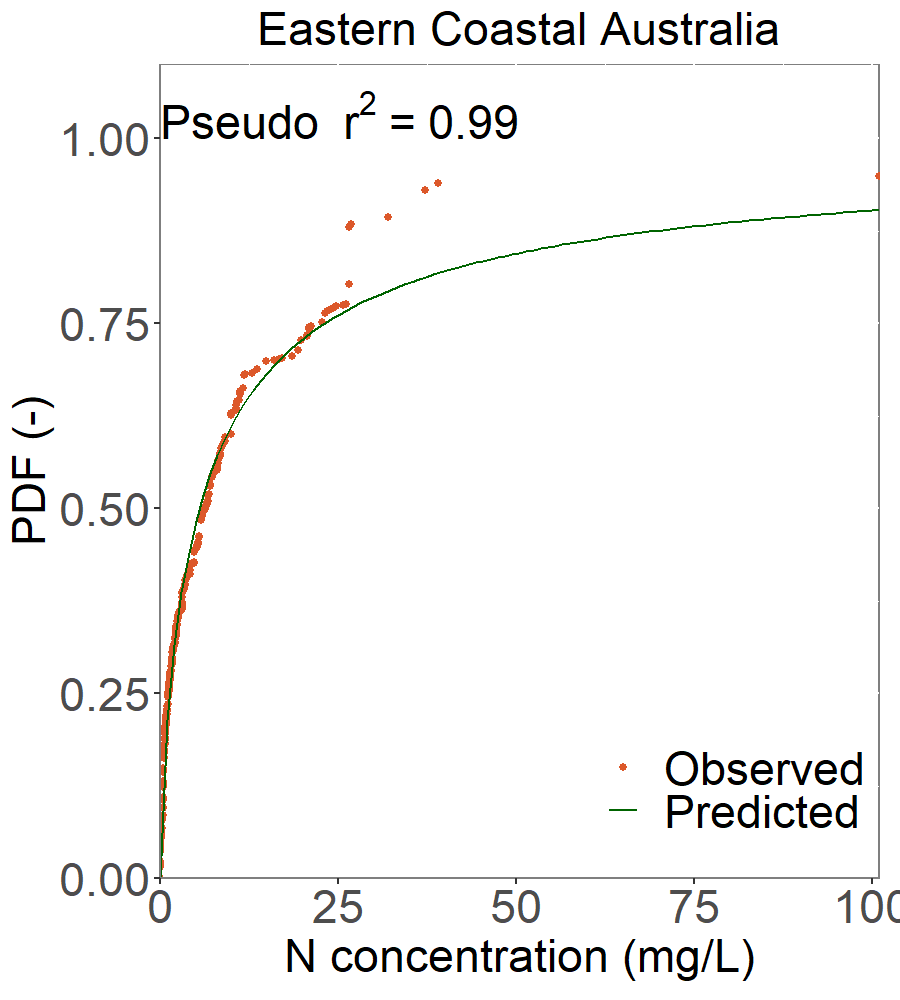

Supplement: Supplementary file 2 — es2c09333_si_002.zip [file es2c09333_si_002.zip › SSD_Ecoregion/Eastern Coastal Australia.tif]

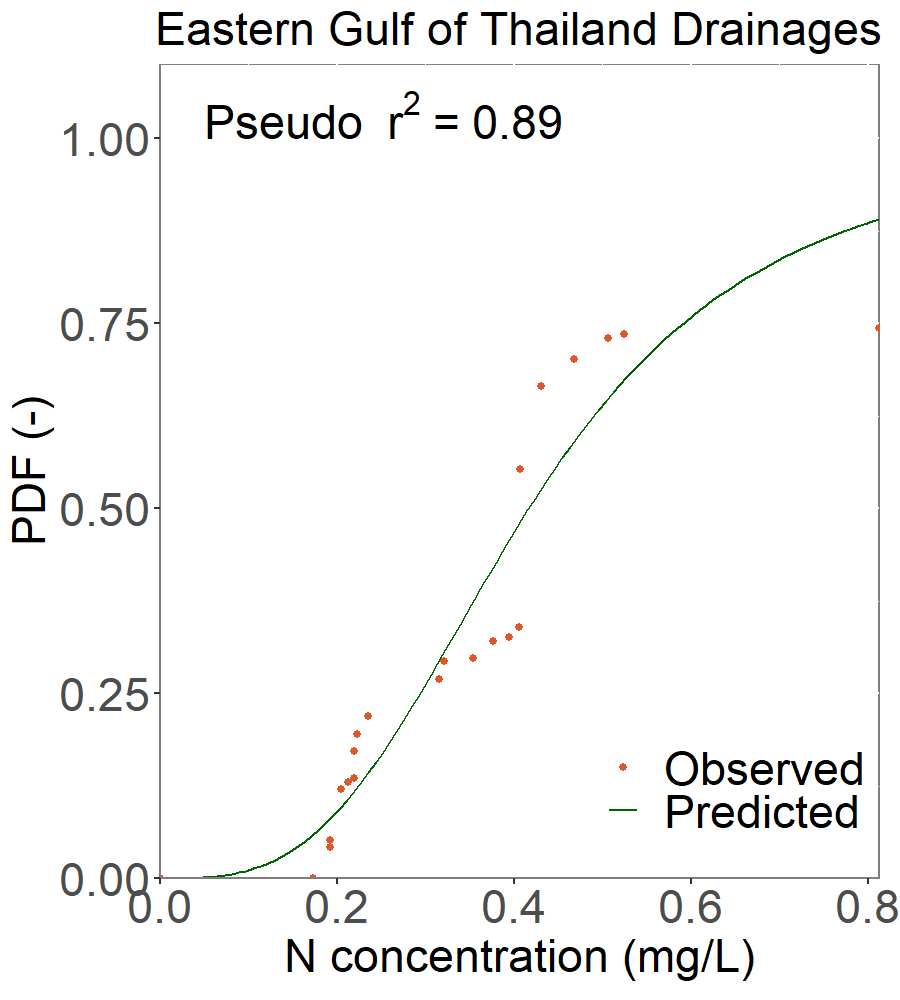

Supplement: Supplementary file 2 — es2c09333_si_002.zip [file es2c09333_si_002.zip › SSD_Ecoregion/Eastern Gulf of Thailand Drainages.tif]

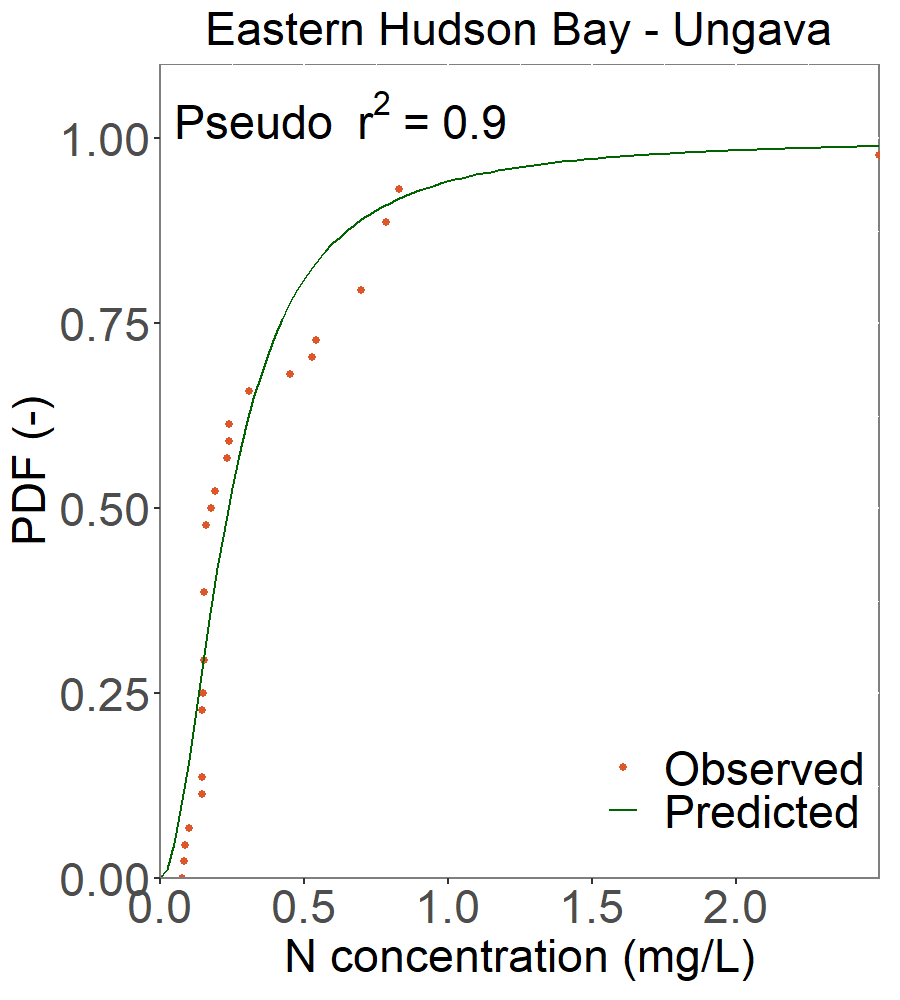

Supplement: Supplementary file 2 — es2c09333_si_002.zip [file es2c09333_si_002.zip › SSD_Ecoregion/Eastern Hudson Bay - Ungava.tif]

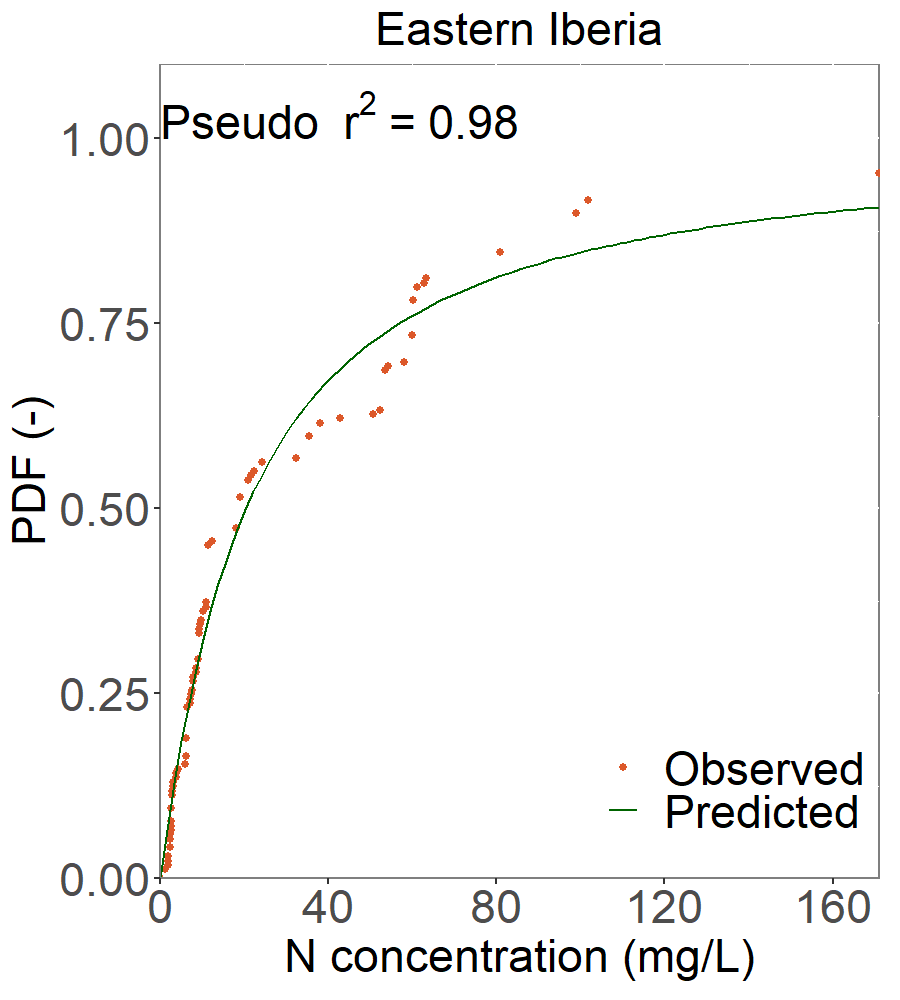

Supplement: Supplementary file 2 — es2c09333_si_002.zip [file es2c09333_si_002.zip › SSD_Ecoregion/Eastern Iberia.tif]

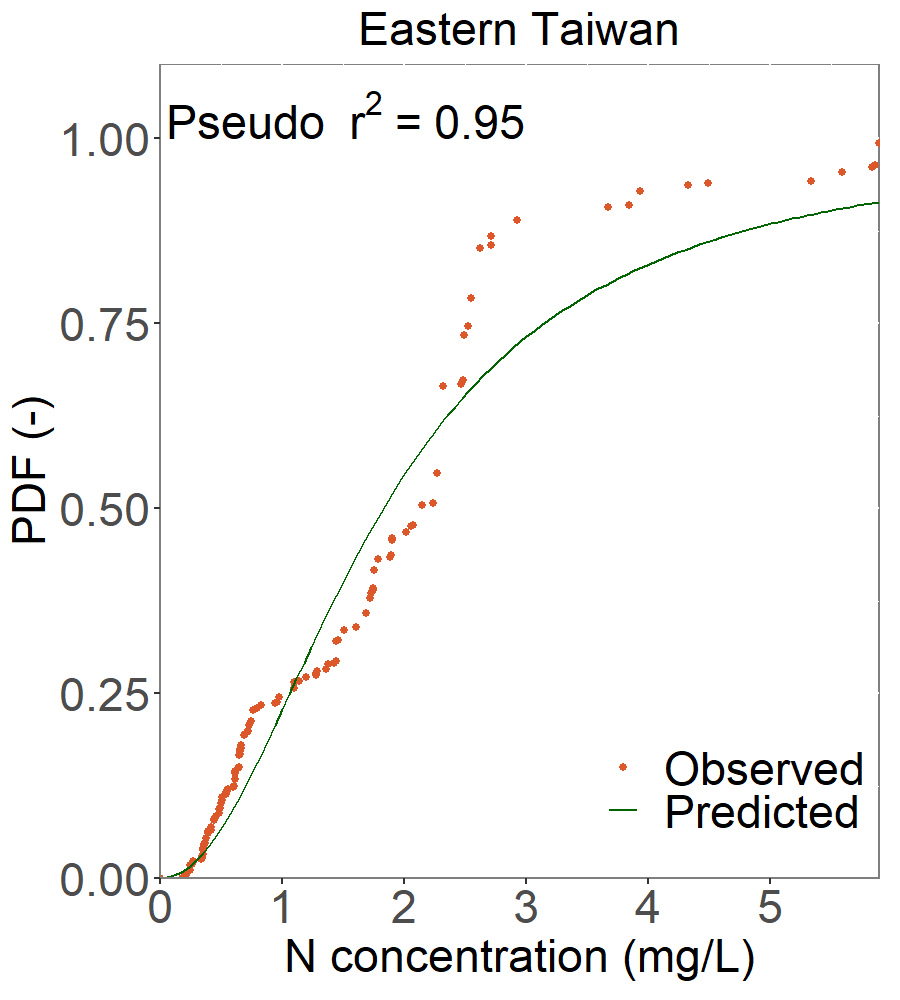

Supplement: Supplementary file 2 — es2c09333_si_002.zip [file es2c09333_si_002.zip › SSD_Ecoregion/Eastern Taiwan.tif]

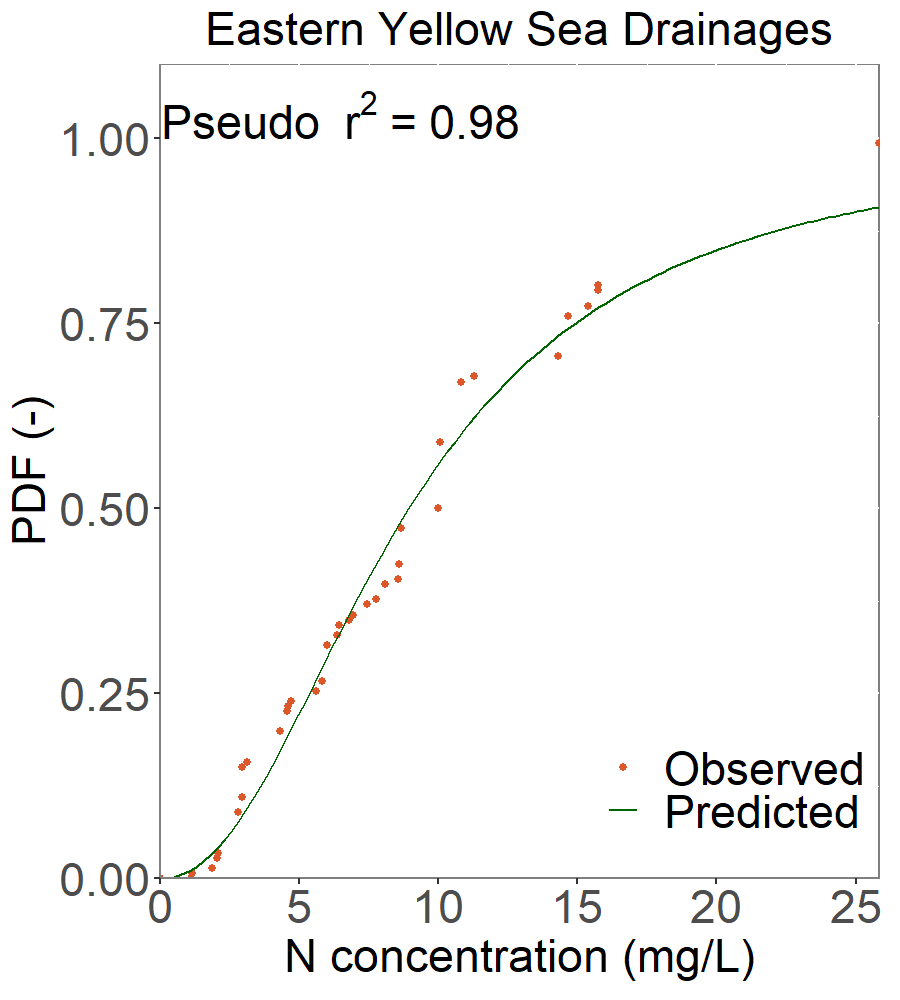

Supplement: Supplementary file 2 — es2c09333_si_002.zip [file es2c09333_si_002.zip › SSD_Ecoregion/Eastern Yellow Sea Drainages.tif]

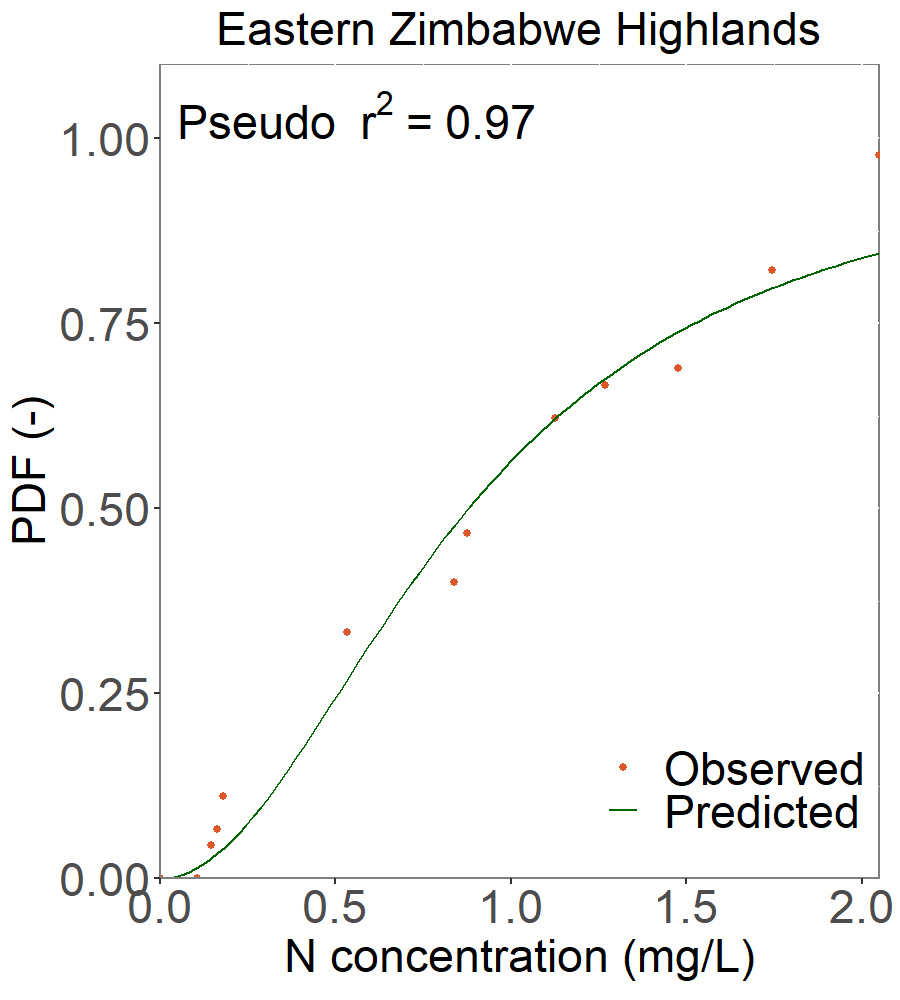

Supplement: Supplementary file 2 — es2c09333_si_002.zip [file es2c09333_si_002.zip › SSD_Ecoregion/Eastern Zimbabwe Highlands.tif]

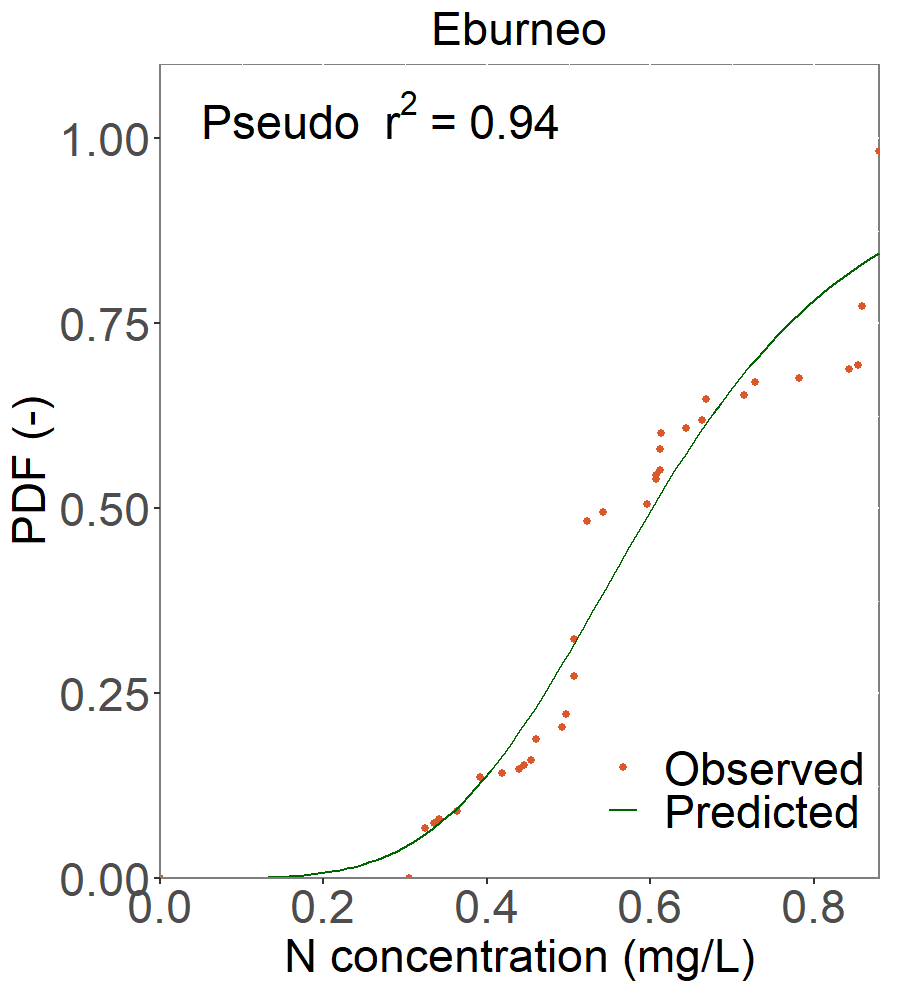

Supplement: Supplementary file 2 — es2c09333_si_002.zip [file es2c09333_si_002.zip › SSD_Ecoregion/Eburneo.tif]

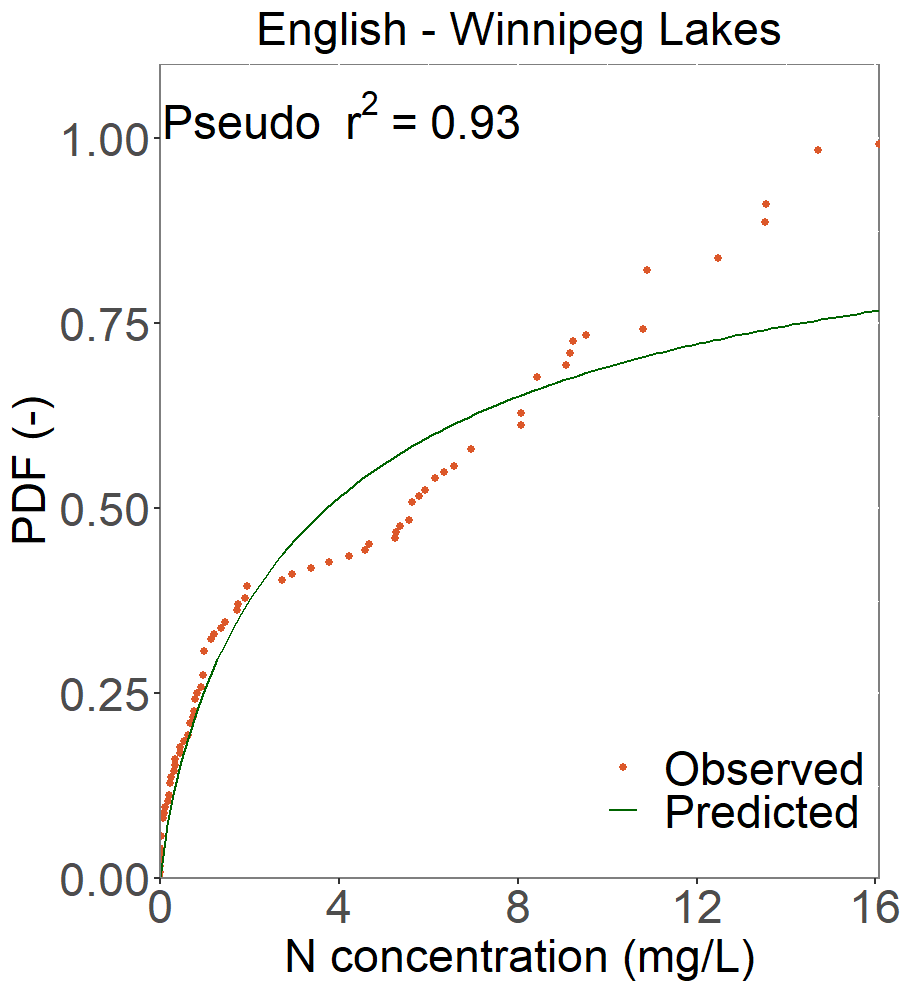

Supplement: Supplementary file 2 — es2c09333_si_002.zip [file es2c09333_si_002.zip › SSD_Ecoregion/English - Winnipeg Lakes.tif]

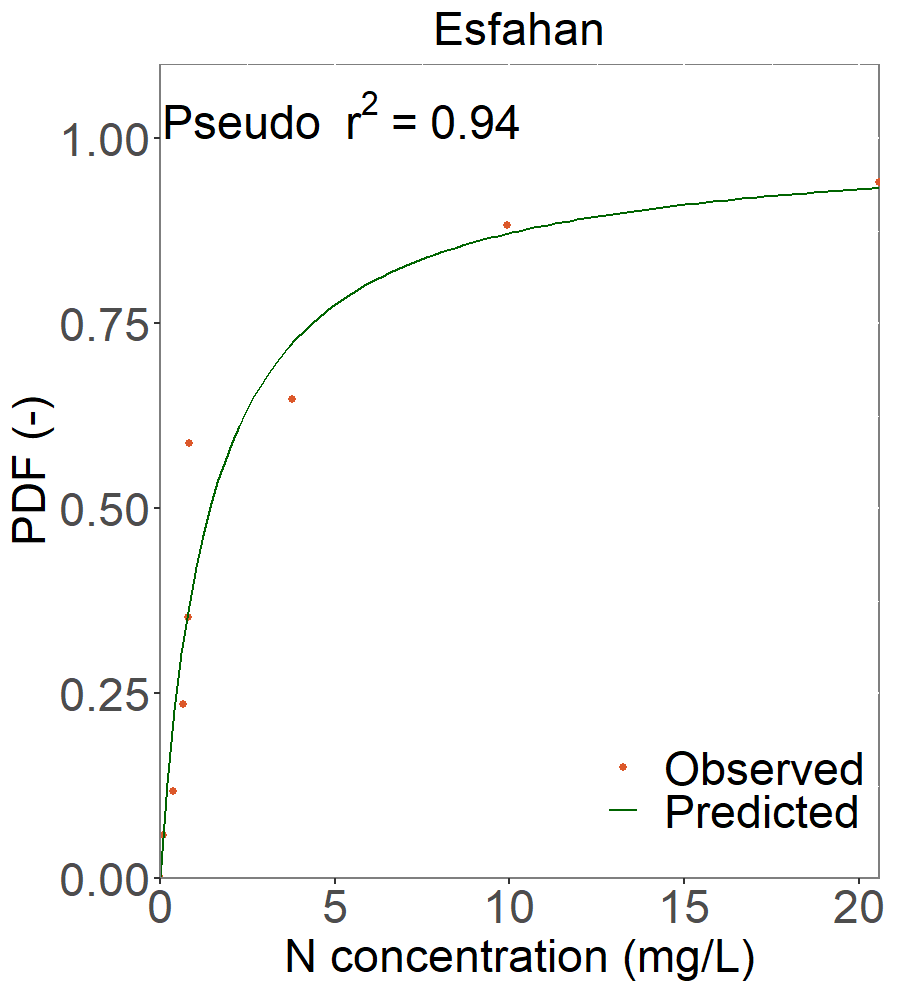

Supplement: Supplementary file 2 — es2c09333_si_002.zip [file es2c09333_si_002.zip › SSD_Ecoregion/Esfahan.tif]

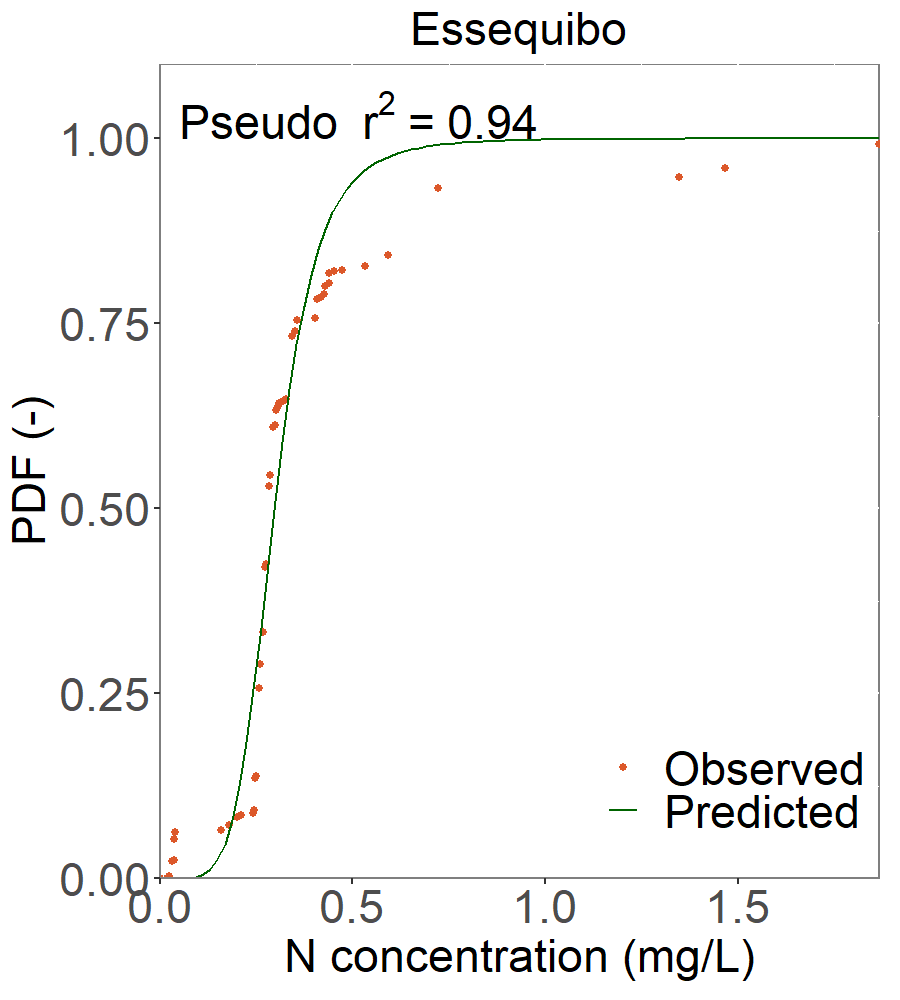

Supplement: Supplementary file 2 — es2c09333_si_002.zip [file es2c09333_si_002.zip › SSD_Ecoregion/Essequibo.tif]

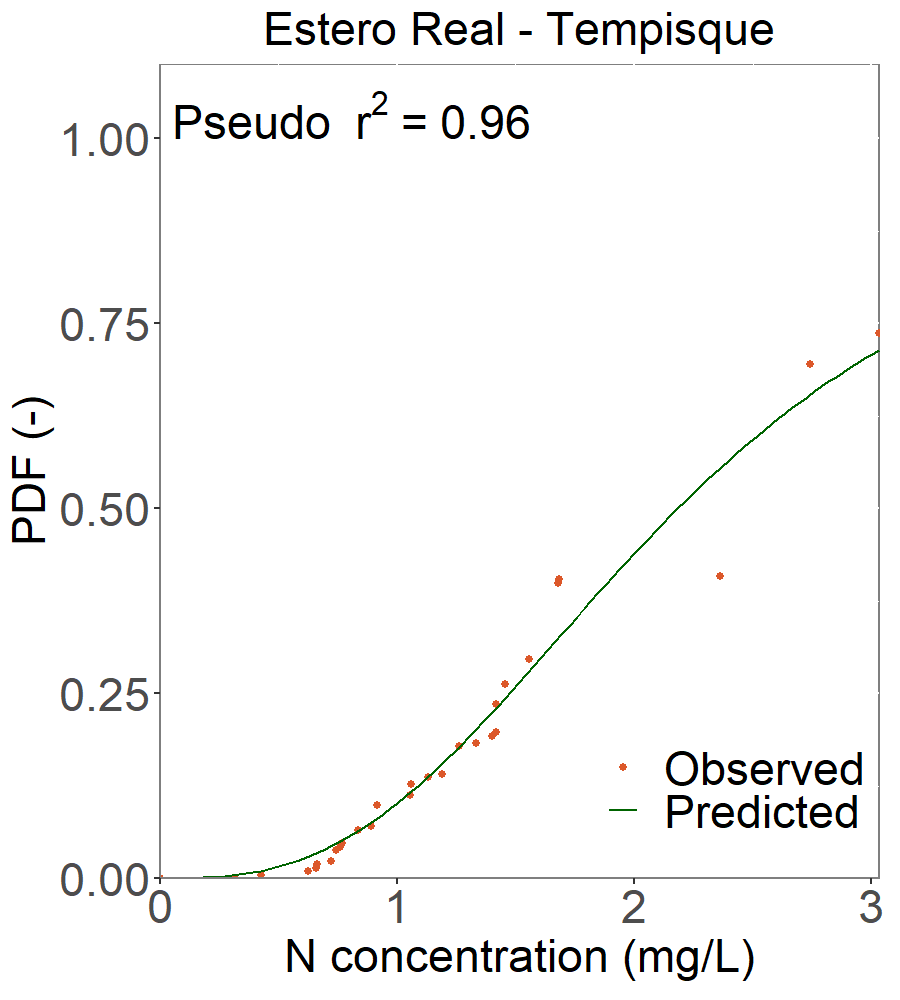

Supplement: Supplementary file 2 — es2c09333_si_002.zip [file es2c09333_si_002.zip › SSD_Ecoregion/Estero Real - Tempisque.tif]

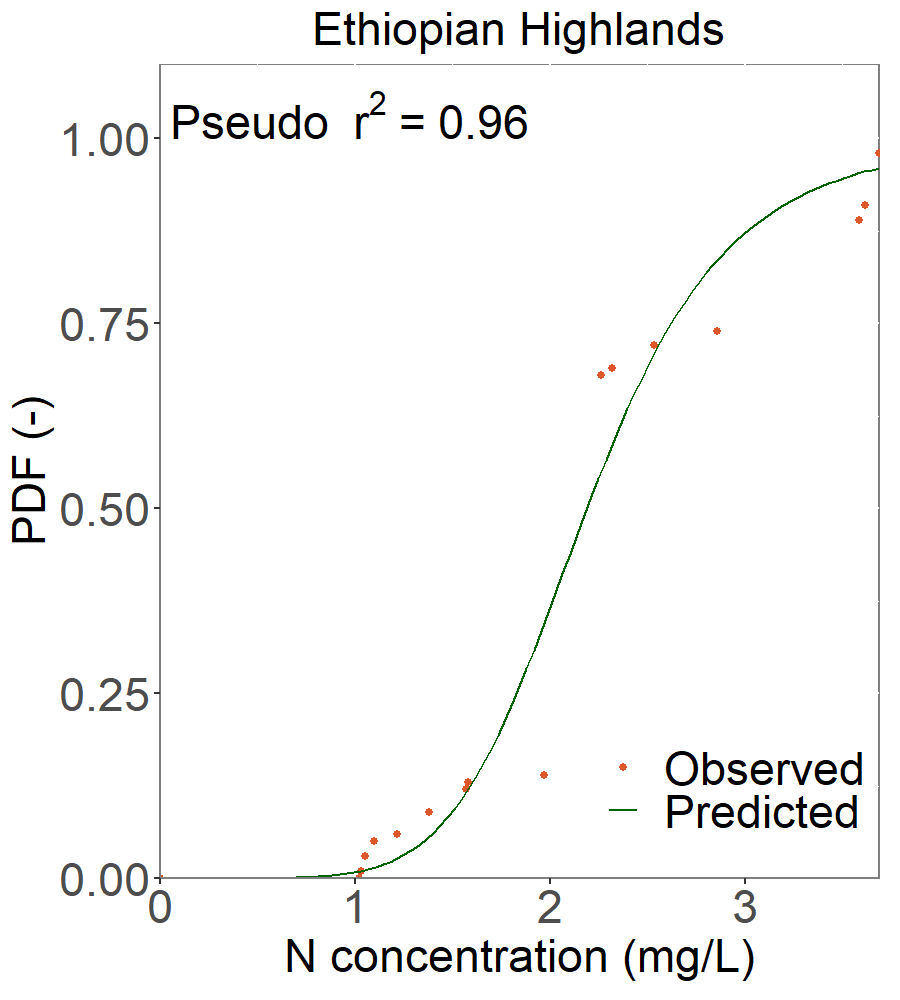

Supplement: Supplementary file 2 — es2c09333_si_002.zip [file es2c09333_si_002.zip › SSD_Ecoregion/Ethiopian Highlands.tif]

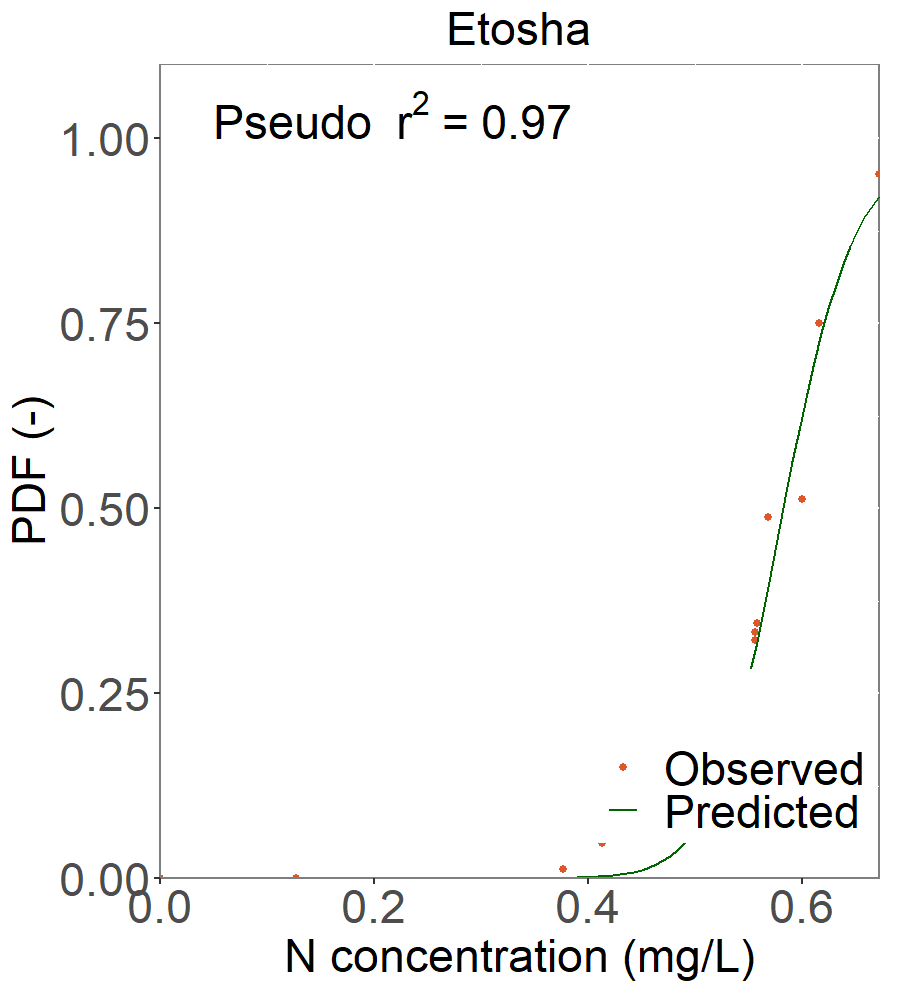

Supplement: Supplementary file 2 — es2c09333_si_002.zip [file es2c09333_si_002.zip › SSD_Ecoregion/Etosha.tif]

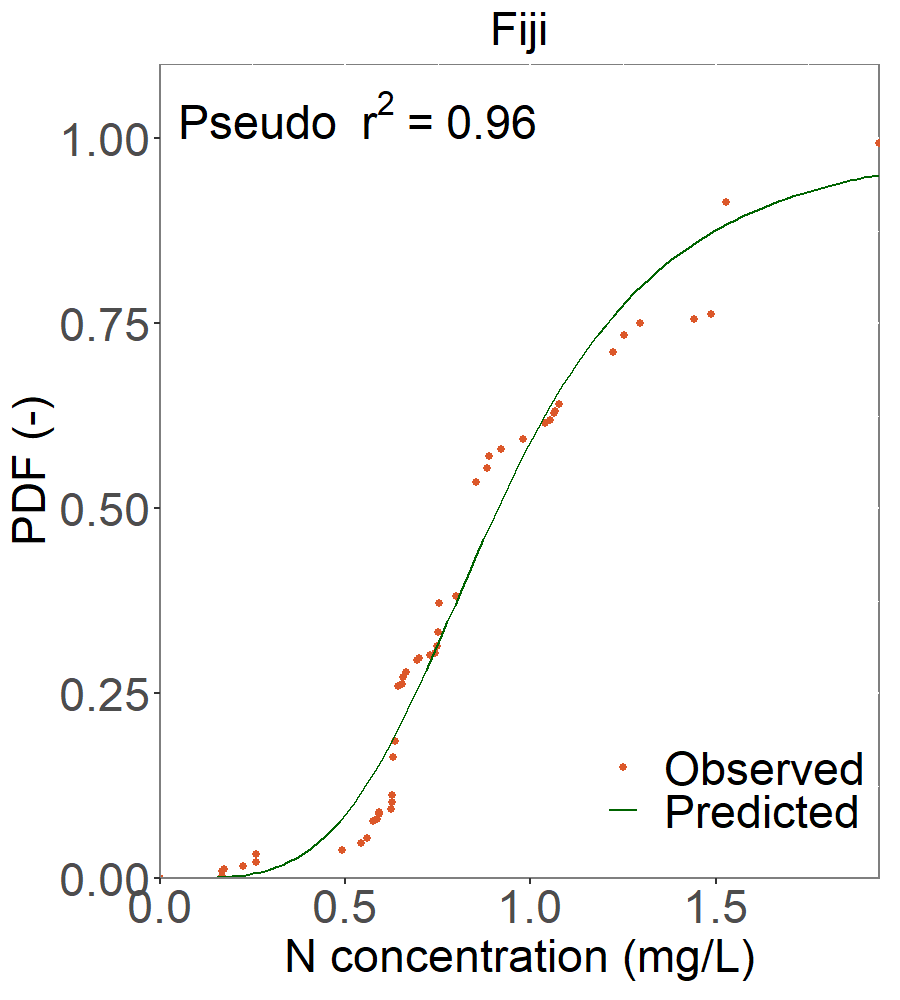

Supplement: Supplementary file 2 — es2c09333_si_002.zip [file es2c09333_si_002.zip › SSD_Ecoregion/Fiji.tif]

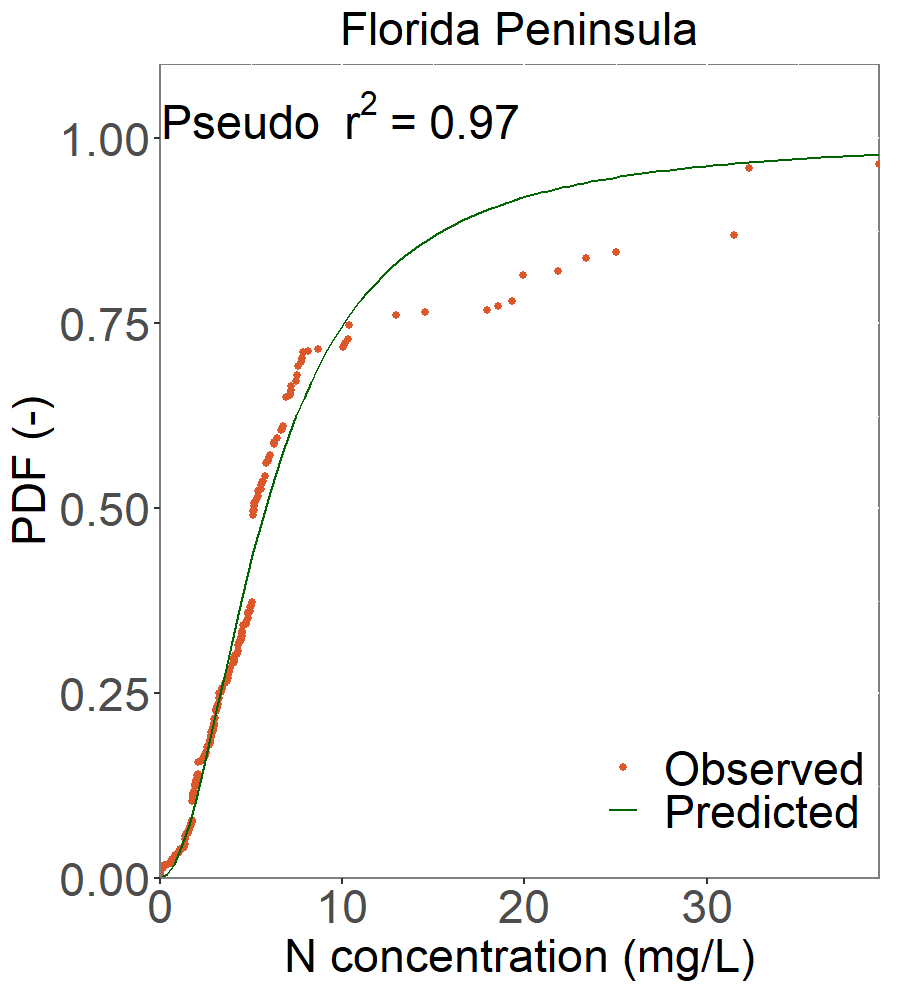

Supplement: Supplementary file 2 — es2c09333_si_002.zip [file es2c09333_si_002.zip › SSD_Ecoregion/Florida Peninsula.tif]

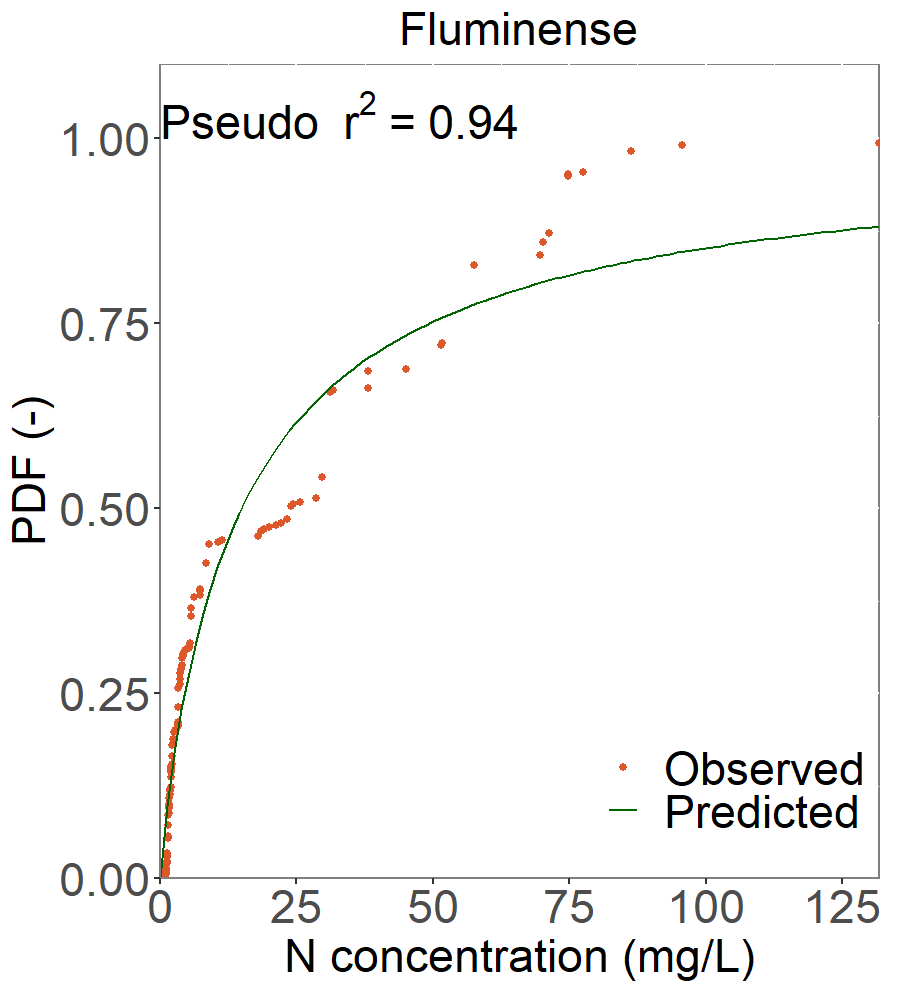

Supplement: Supplementary file 2 — es2c09333_si_002.zip [file es2c09333_si_002.zip › SSD_Ecoregion/Fluminense.tif]

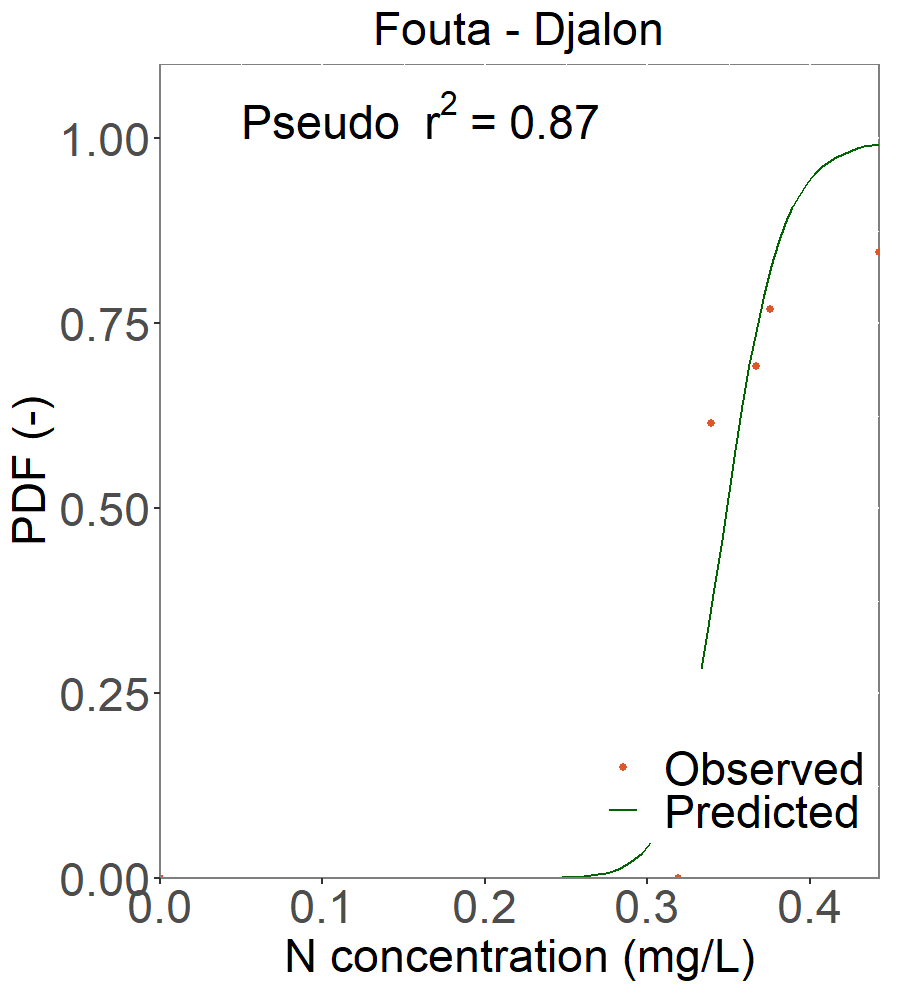

Supplement: Supplementary file 2 — es2c09333_si_002.zip [file es2c09333_si_002.zip › SSD_Ecoregion/Fouta - Djalon.tif]

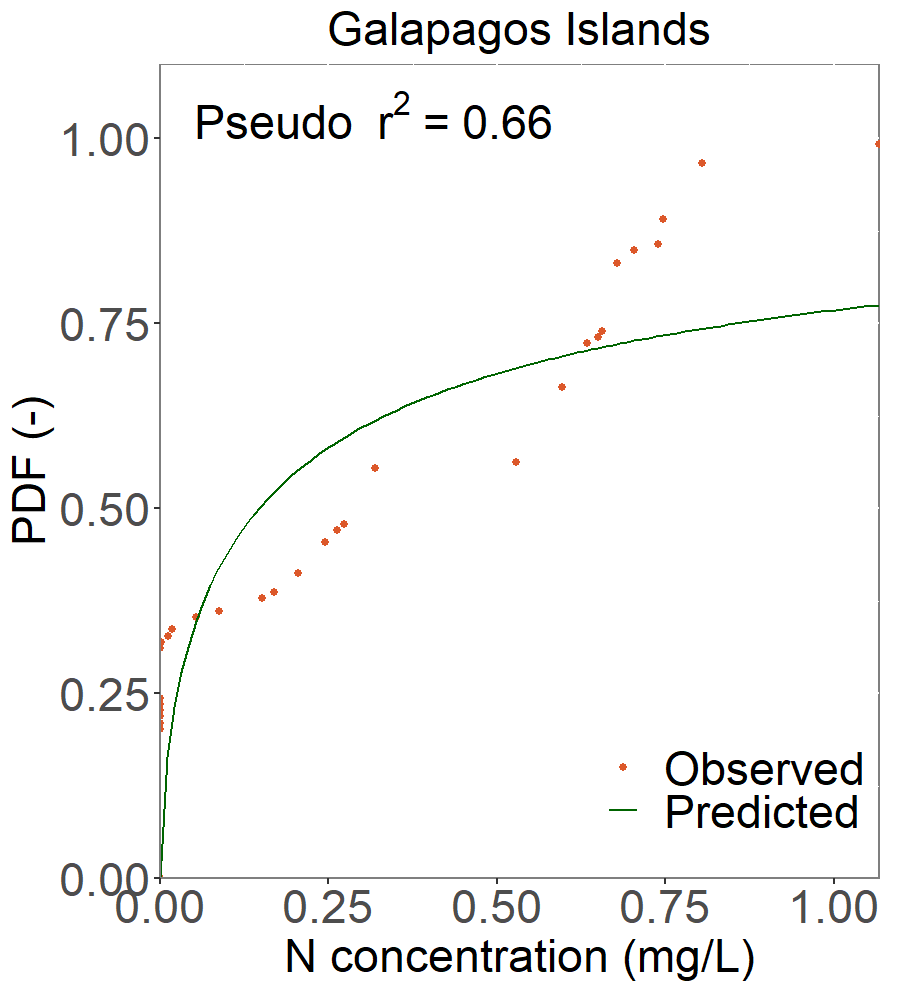

Supplement: Supplementary file 2 — es2c09333_si_002.zip [file es2c09333_si_002.zip › SSD_Ecoregion/Galapagos Islands.tif]

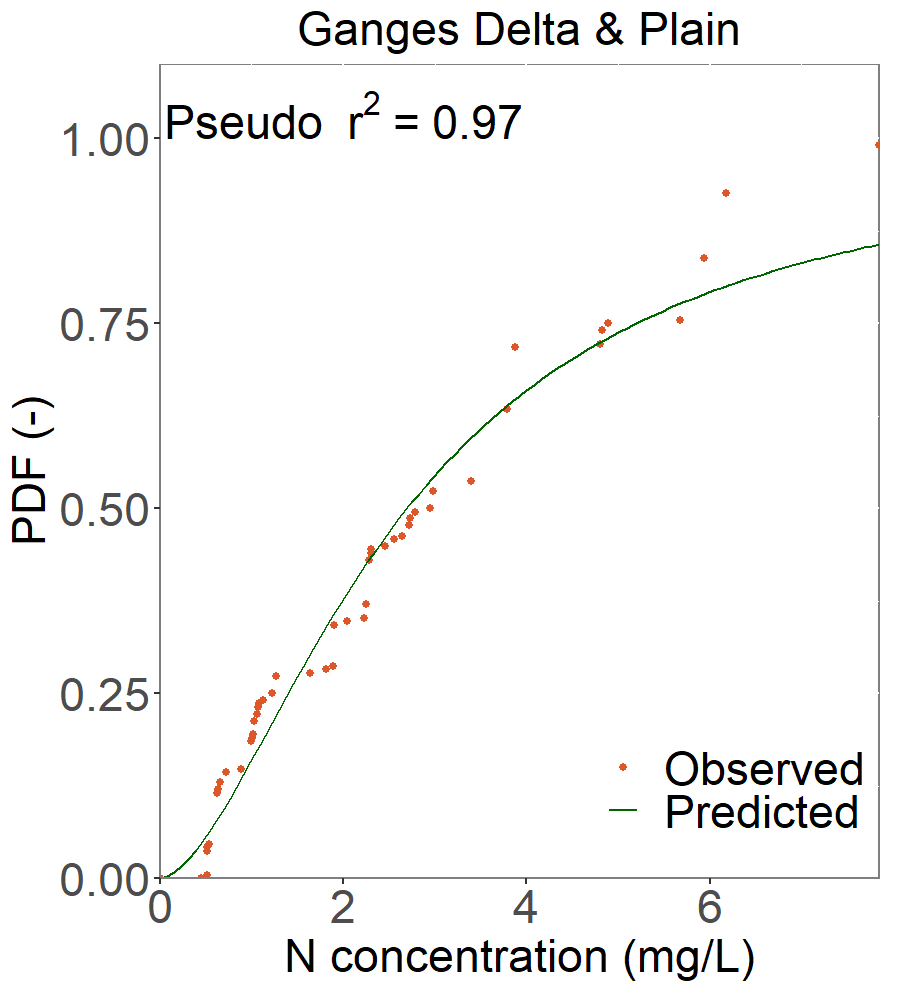

Supplement: Supplementary file 2 — es2c09333_si_002.zip [file es2c09333_si_002.zip › SSD_Ecoregion/Ganges Delta & Plain.tif]

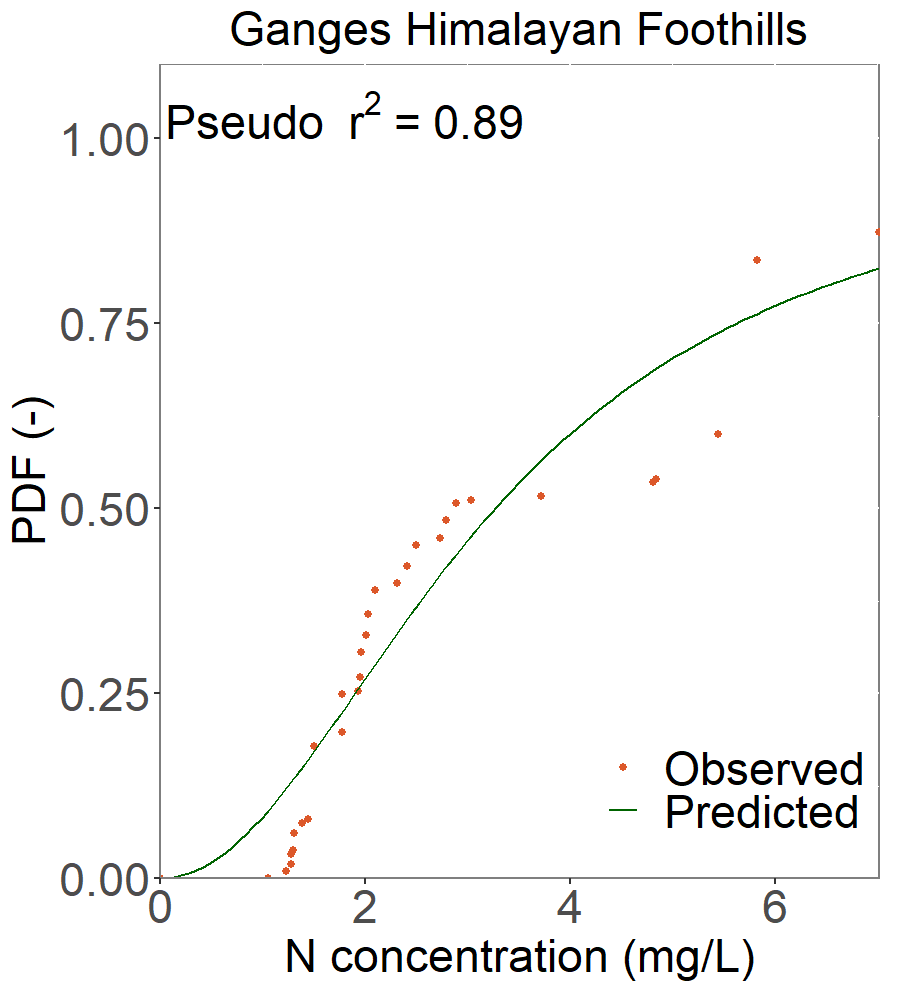

Supplement: Supplementary file 2 — es2c09333_si_002.zip [file es2c09333_si_002.zip › SSD_Ecoregion/Ganges Himalayan Foothills.tif]

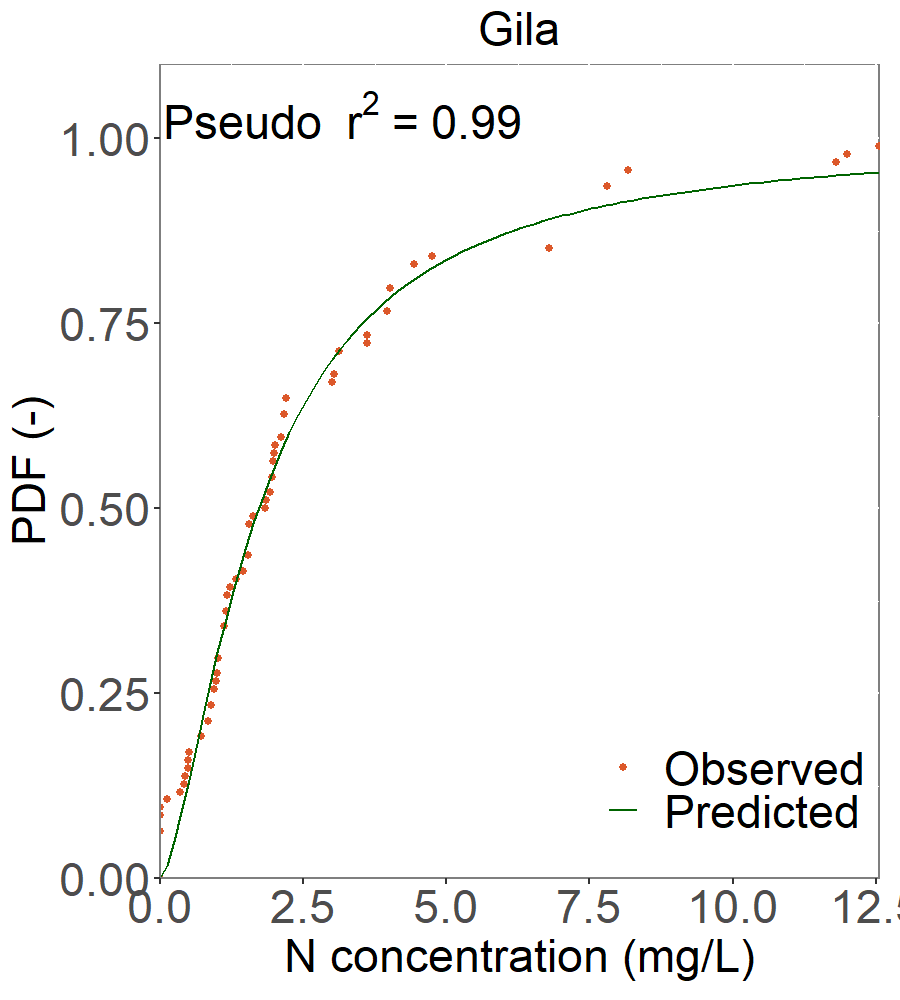

Supplement: Supplementary file 2 — es2c09333_si_002.zip [file es2c09333_si_002.zip › SSD_Ecoregion/Gila.tif]

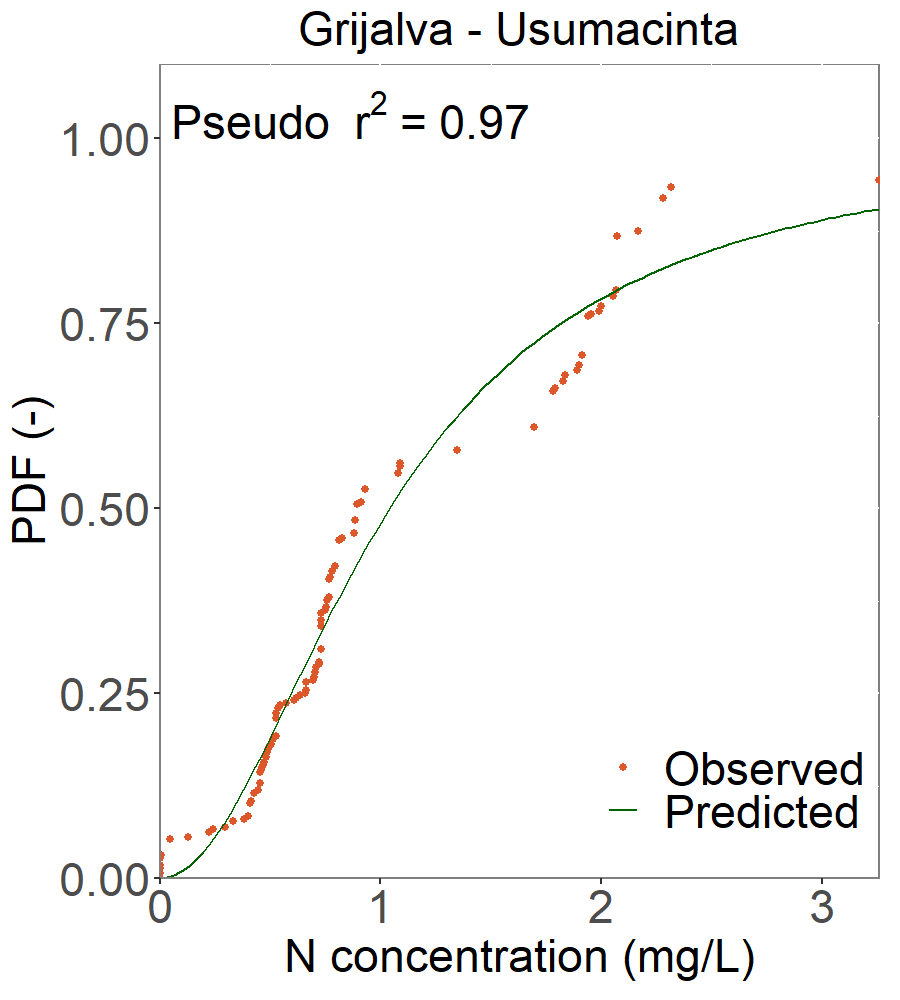

Supplement: Supplementary file 2 — es2c09333_si_002.zip [file es2c09333_si_002.zip › SSD_Ecoregion/Grijalva - Usumacinta.tif]

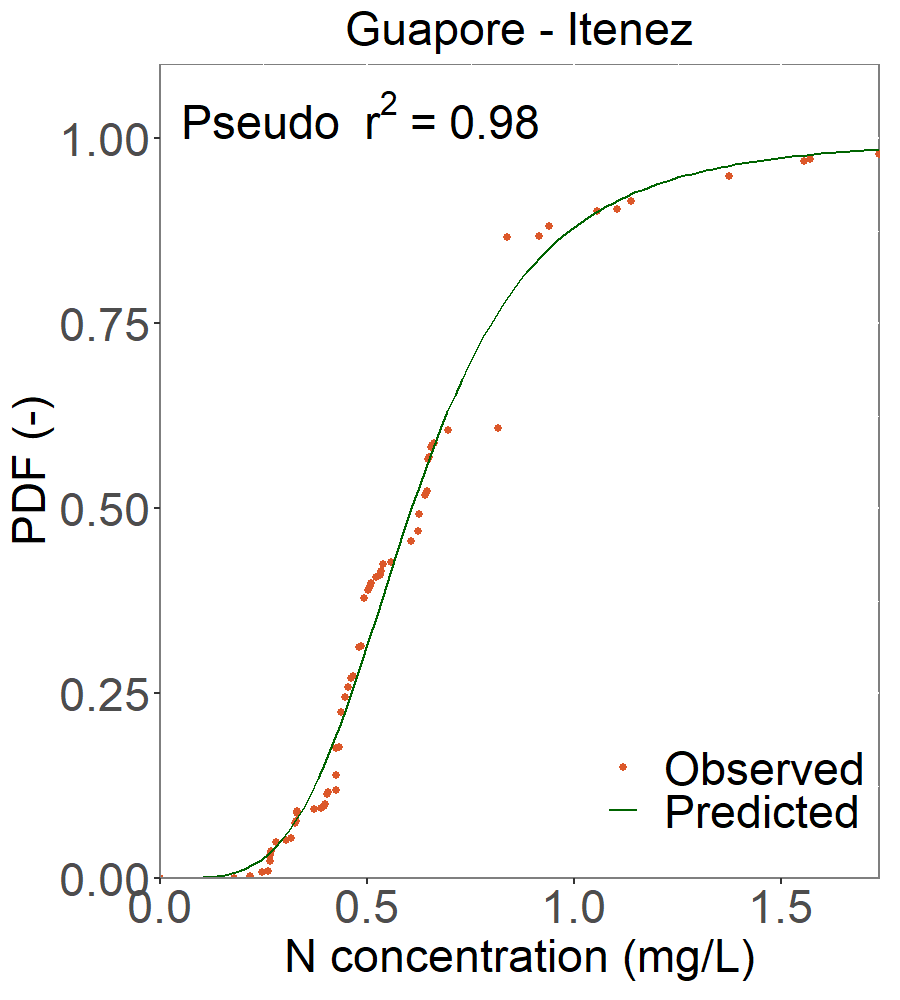

Supplement: Supplementary file 2 — es2c09333_si_002.zip [file es2c09333_si_002.zip › SSD_Ecoregion/Guapore - Itenez.tif]

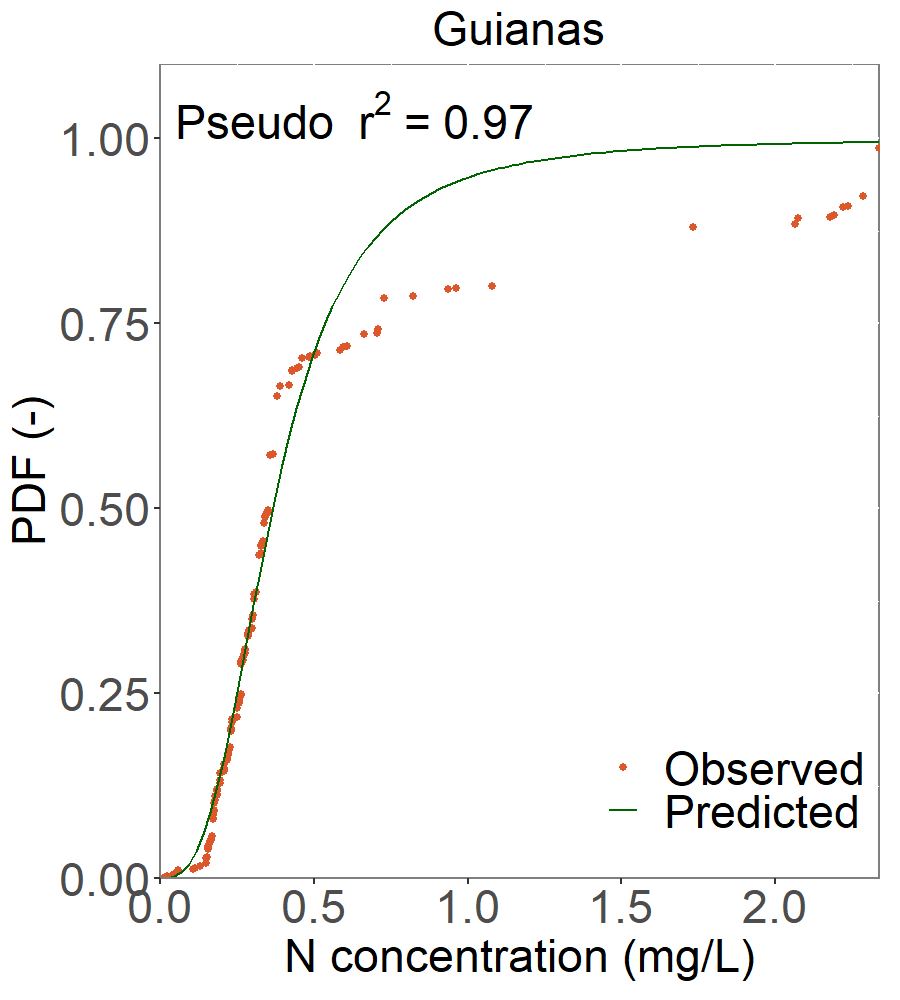

Supplement: Supplementary file 2 — es2c09333_si_002.zip [file es2c09333_si_002.zip › SSD_Ecoregion/Guianas.tif]

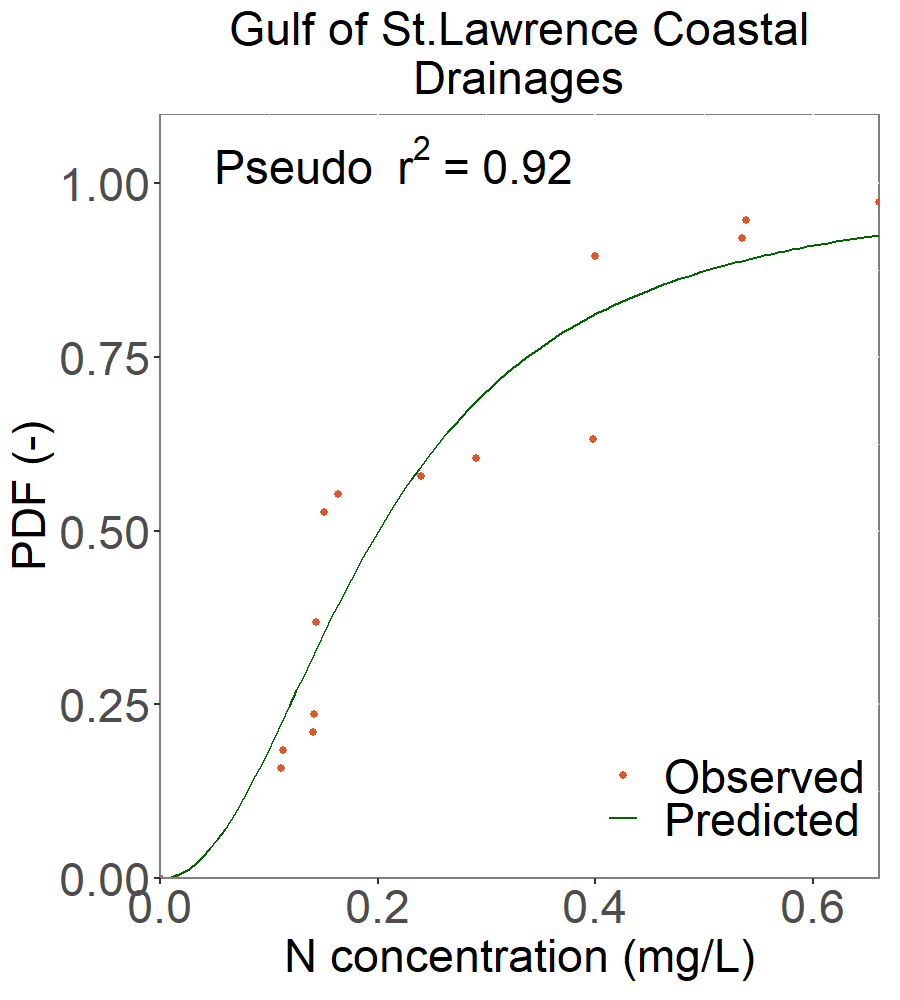

Supplement: Supplementary file 2 — es2c09333_si_002.zip [file es2c09333_si_002.zip › SSD_Ecoregion/Gulf of St.Lawrence Coastal Drainages.tif]

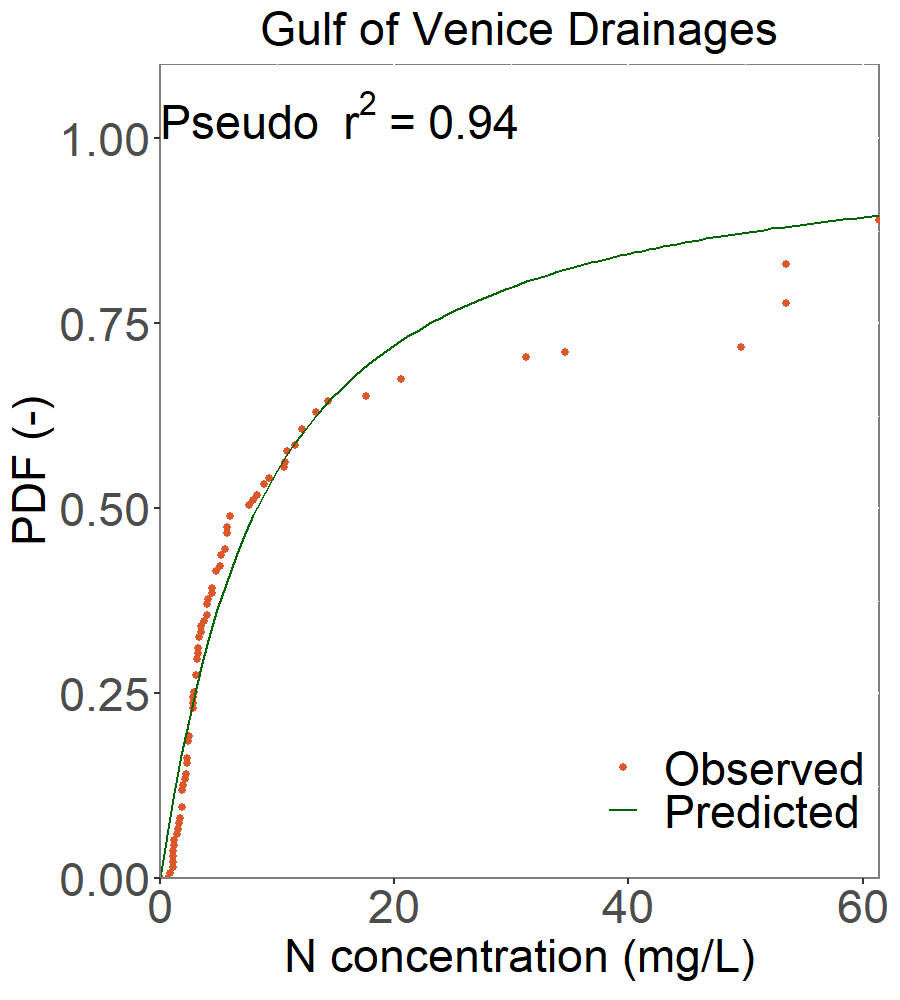

Supplement: Supplementary file 2 — es2c09333_si_002.zip [file es2c09333_si_002.zip › SSD_Ecoregion/Gulf of Venice Drainages.tif]

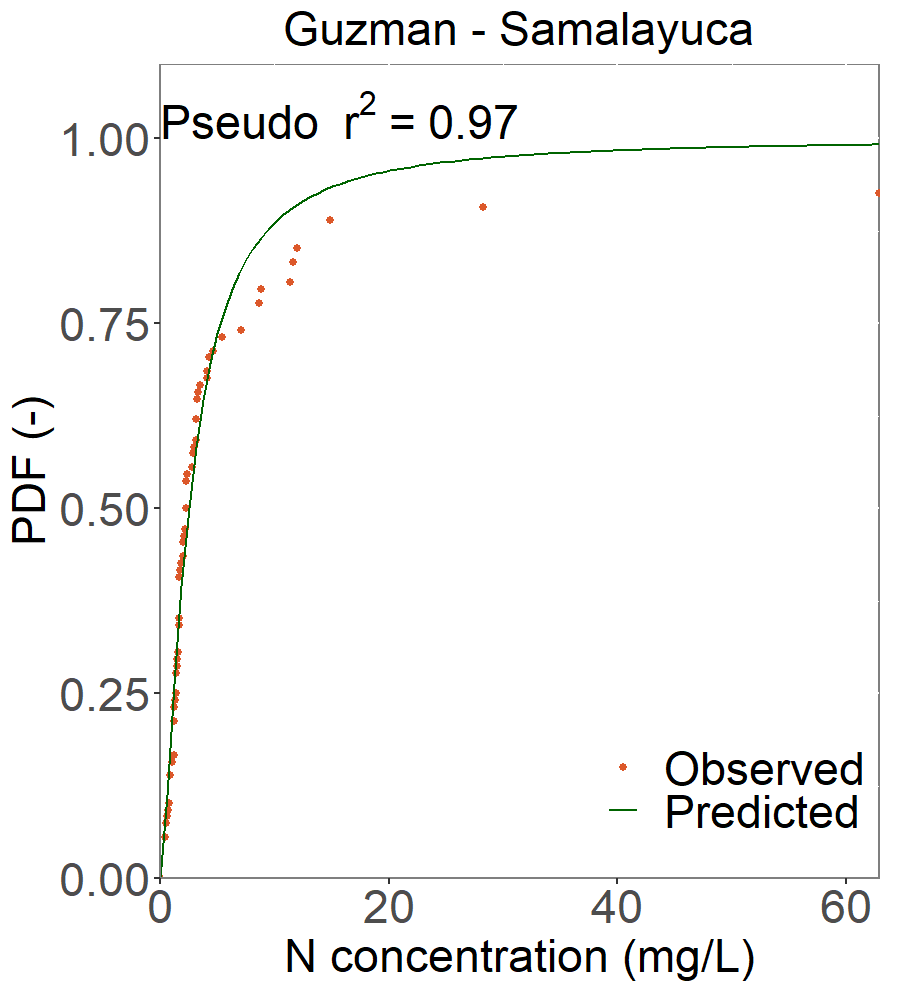

Supplement: Supplementary file 2 — es2c09333_si_002.zip [file es2c09333_si_002.zip › SSD_Ecoregion/Guzman - Samalayuca.tif]

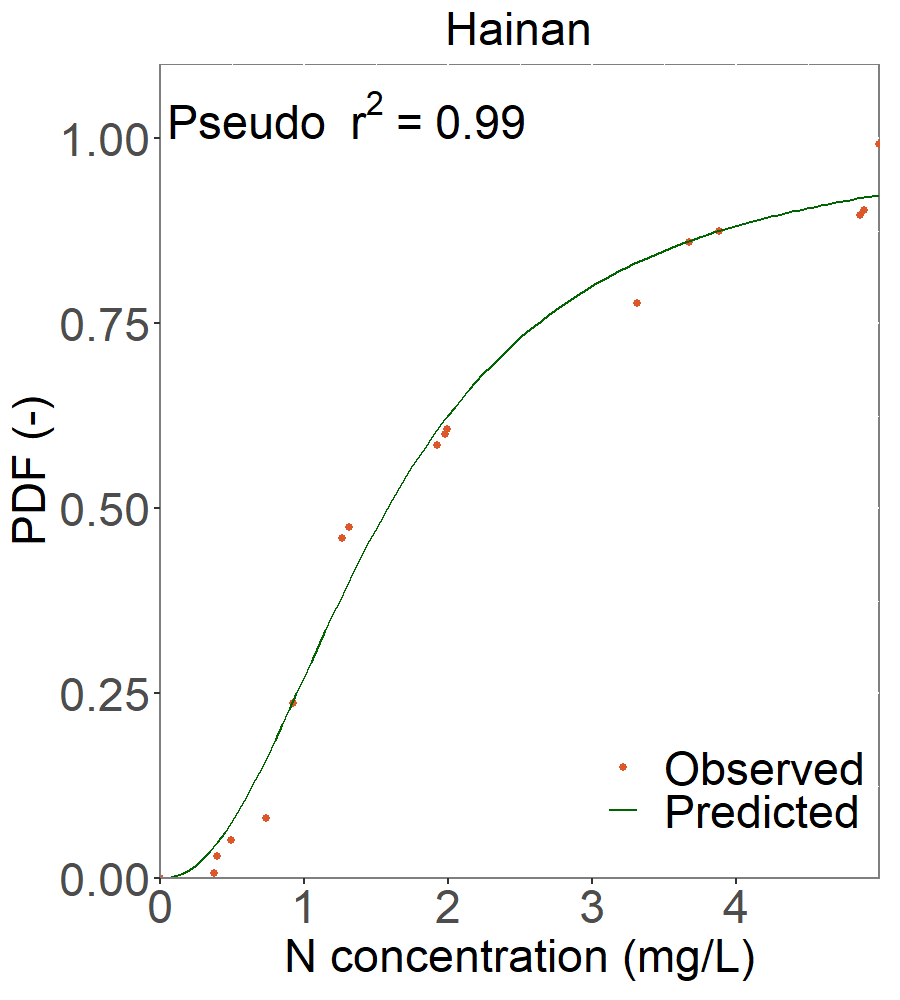

Supplement: Supplementary file 2 — es2c09333_si_002.zip [file es2c09333_si_002.zip › SSD_Ecoregion/Hainan.tif]

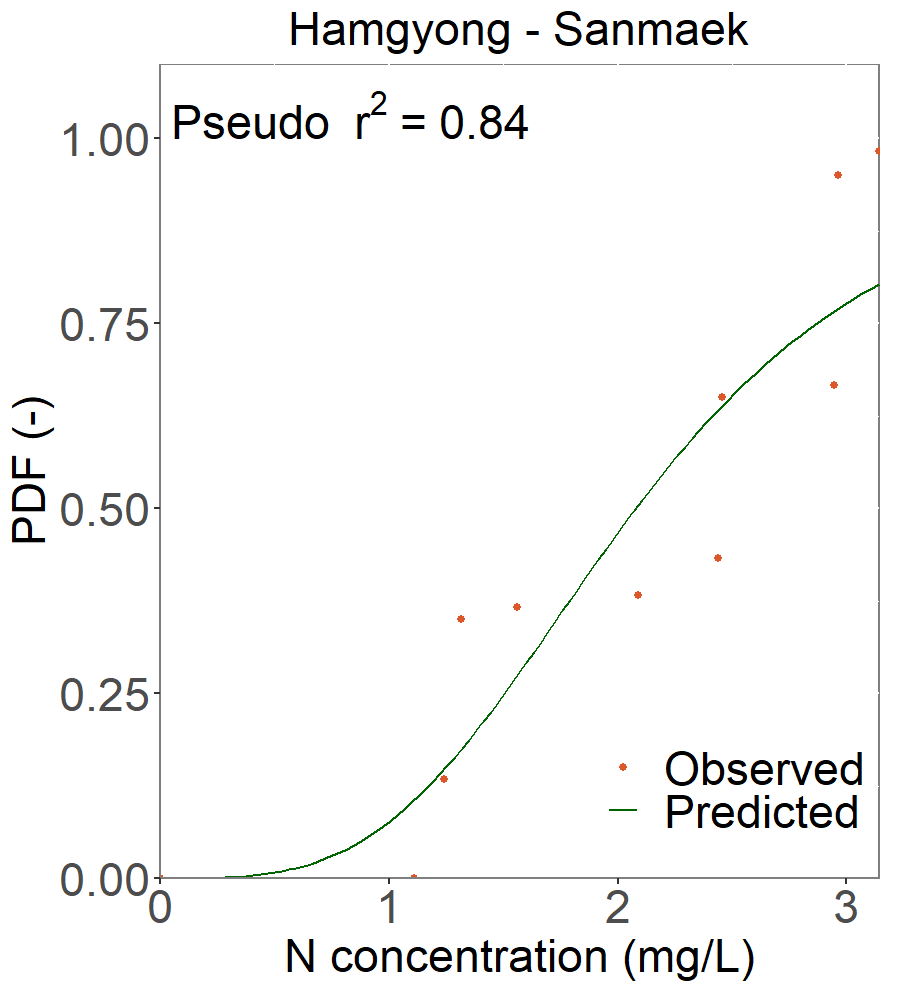

Supplement: Supplementary file 2 — es2c09333_si_002.zip [file es2c09333_si_002.zip › SSD_Ecoregion/Hamgyong - Sanmaek.tif]
