# Supplementary figures and images for: Effects of Nitrogen Emissions on Fish Species Richness across the World’s Freshwater Ecoregions (part 3 of 3)
Source: Environ Sci Technol. 2023 May 22;57(22):8347–54. doi: 10.1021/acs.est.2c09333 (PMC10249400; doi:10.1021/acs.est.2c09333)

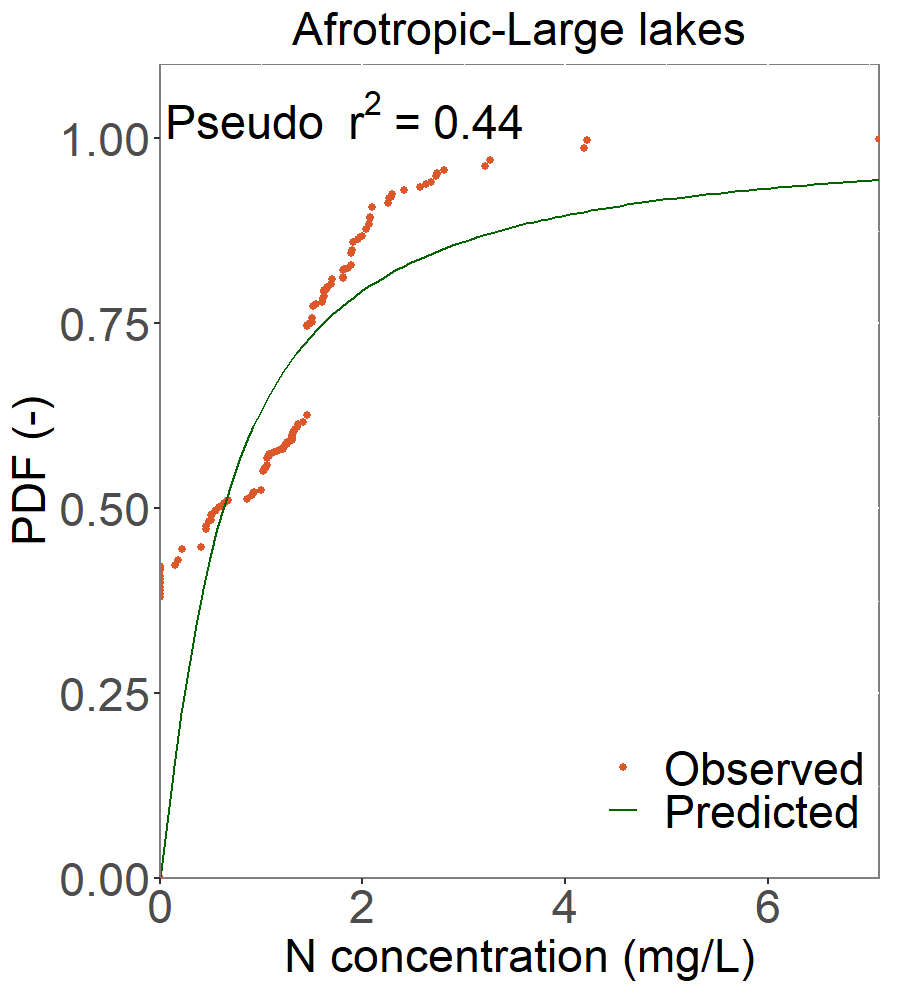

Supplement: Supplementary file 3 — es2c09333_si_003.zip [file es2c09333_si_003.zip › SSD_RMHT/Afrotropic-Large lakes.tif]

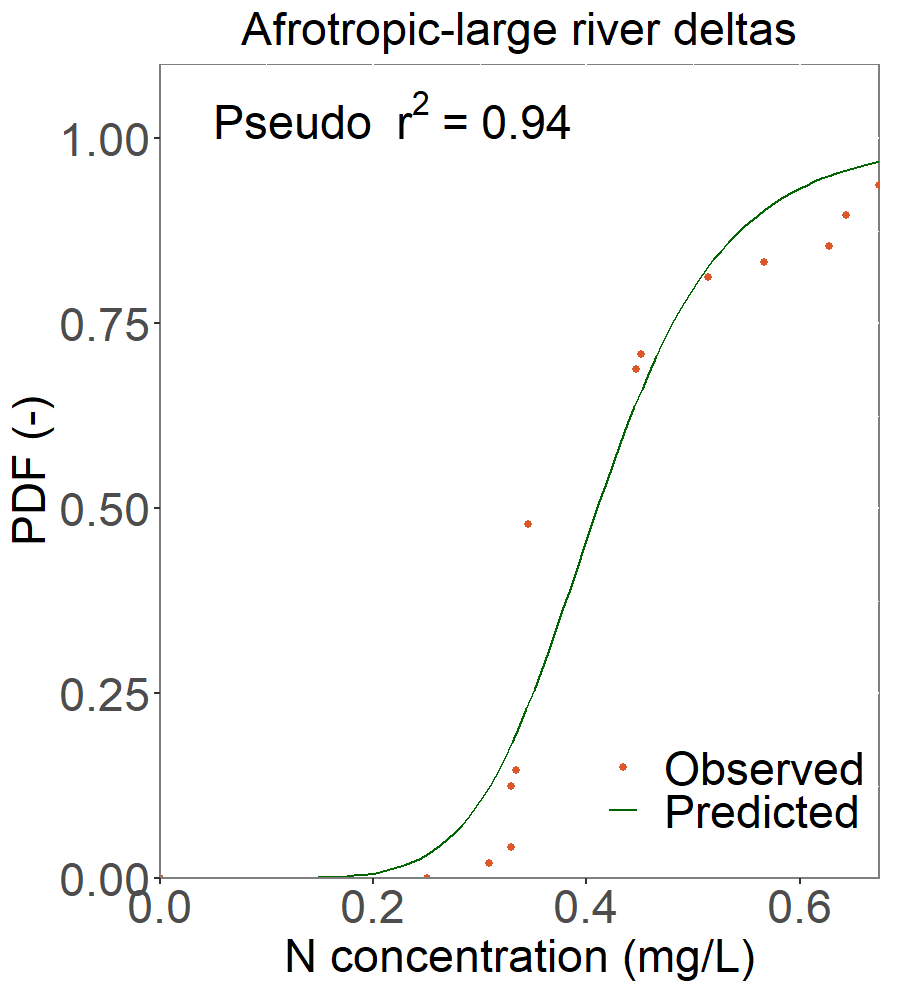

Supplement: Supplementary file 3 — es2c09333_si_003.zip [file es2c09333_si_003.zip › SSD_RMHT/Afrotropic-large river deltas.tif]

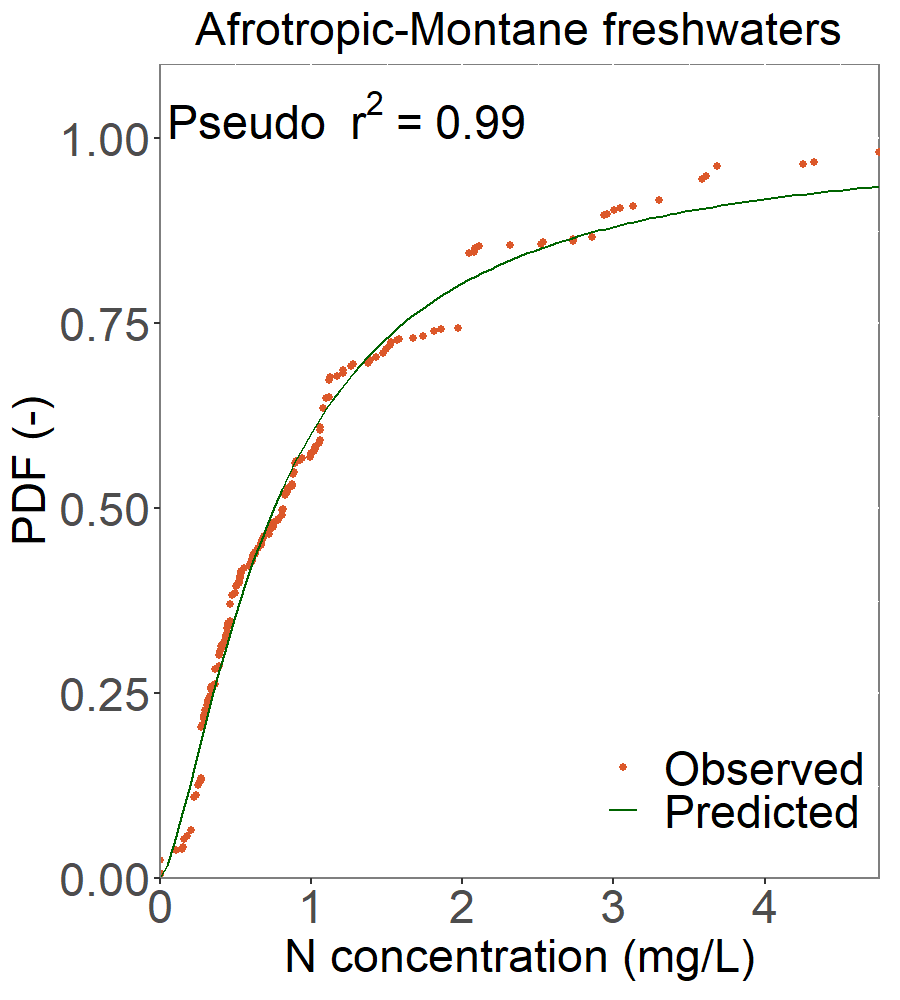

Supplement: Supplementary file 3 — es2c09333_si_003.zip [file es2c09333_si_003.zip › SSD_RMHT/Afrotropic-Montane freshwaters.tif]

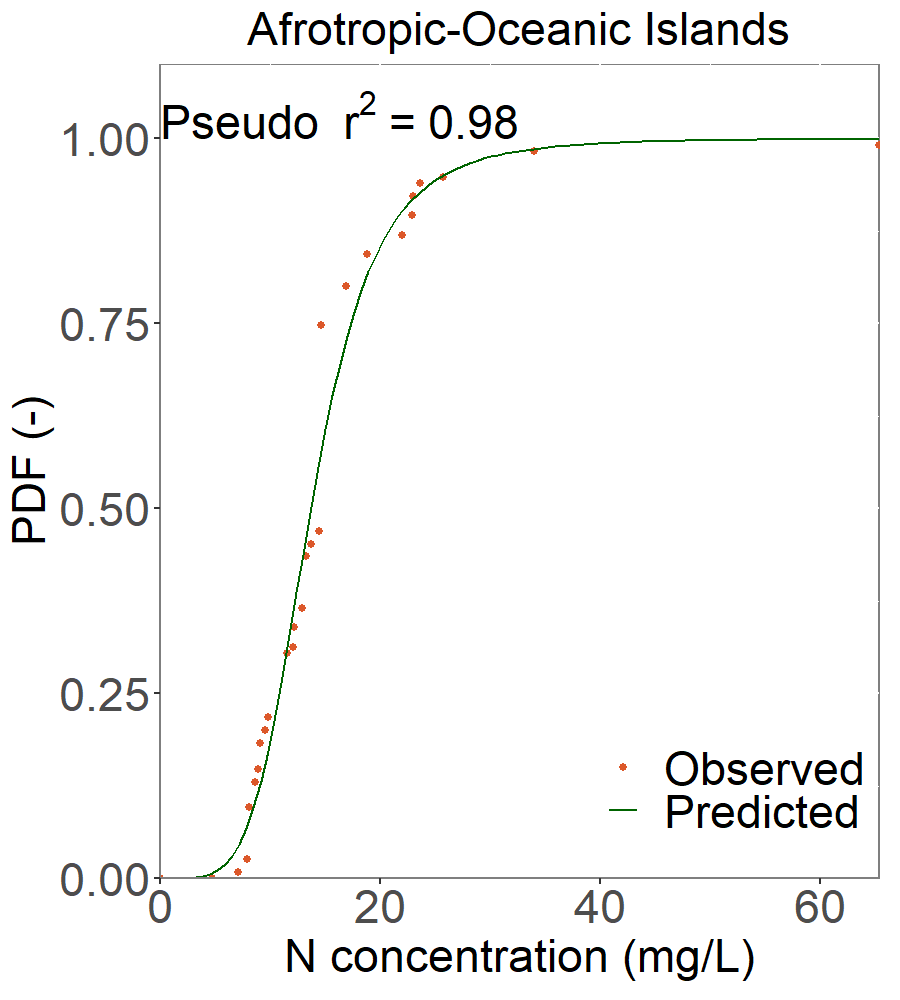

Supplement: Supplementary file 3 — es2c09333_si_003.zip [file es2c09333_si_003.zip › SSD_RMHT/Afrotropic-Oceanic Islands.tif]

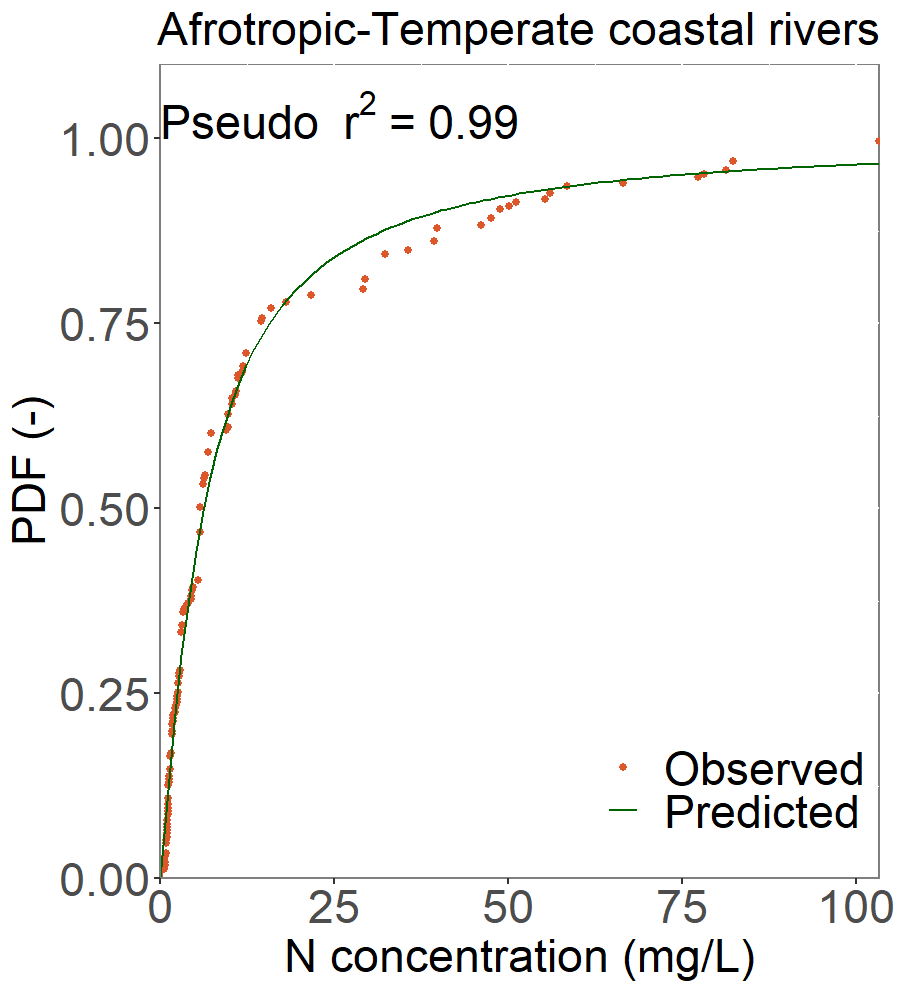

Supplement: Supplementary file 3 — es2c09333_si_003.zip [file es2c09333_si_003.zip › SSD_RMHT/Afrotropic-Temperate coastal rivers.tif]

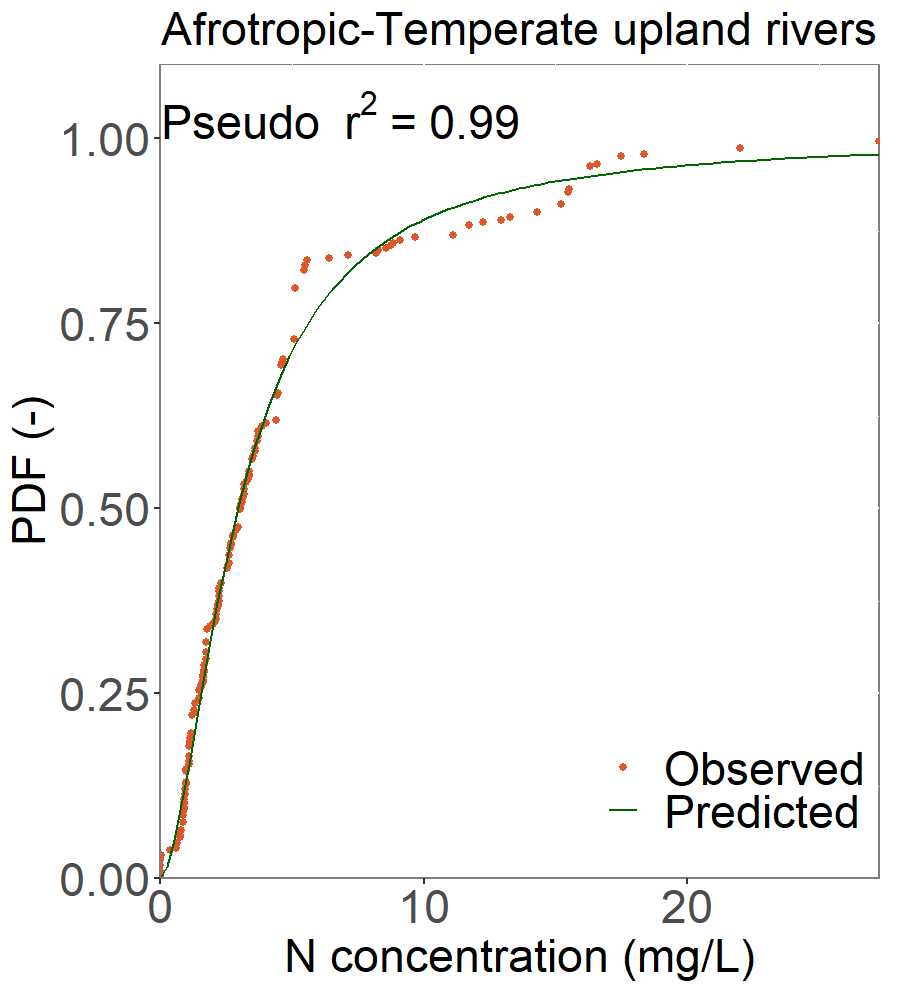

Supplement: Supplementary file 3 — es2c09333_si_003.zip [file es2c09333_si_003.zip › SSD_RMHT/Afrotropic-Temperate upland rivers.tif]

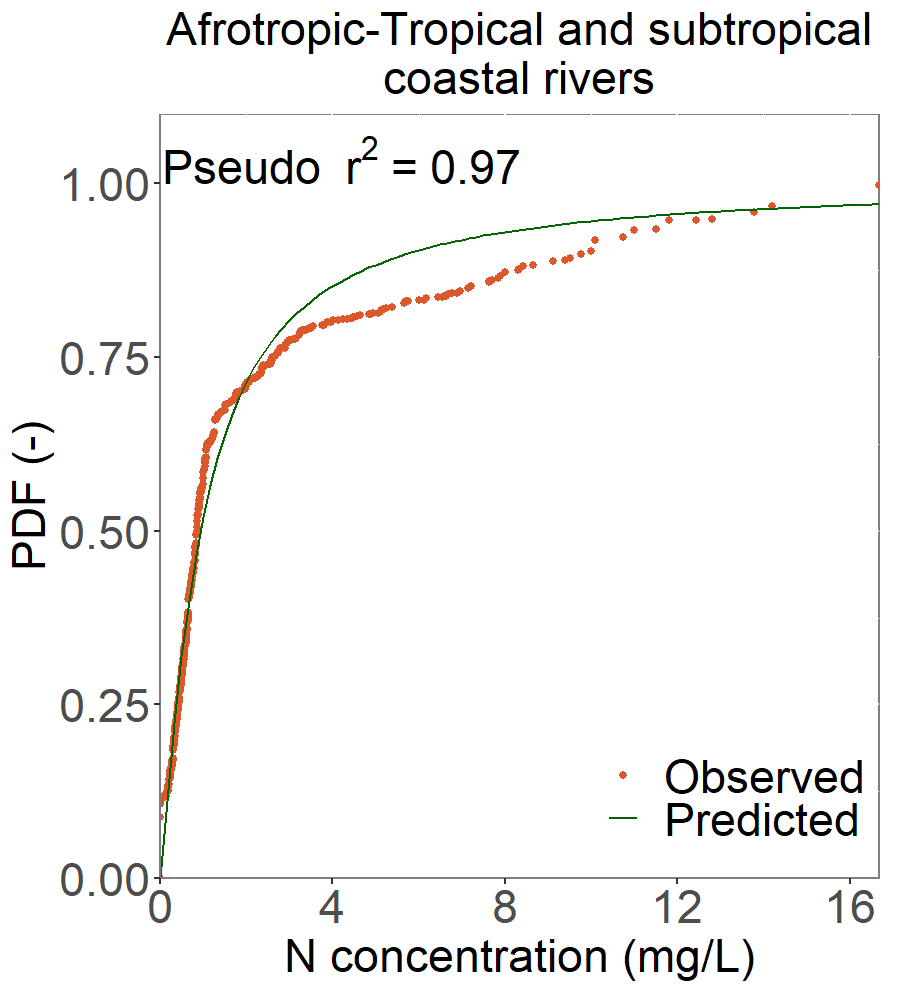

Supplement: Supplementary file 3 — es2c09333_si_003.zip [file es2c09333_si_003.zip › SSD_RMHT/Afrotropic-Tropical and subtropical coastal rivers.tif]

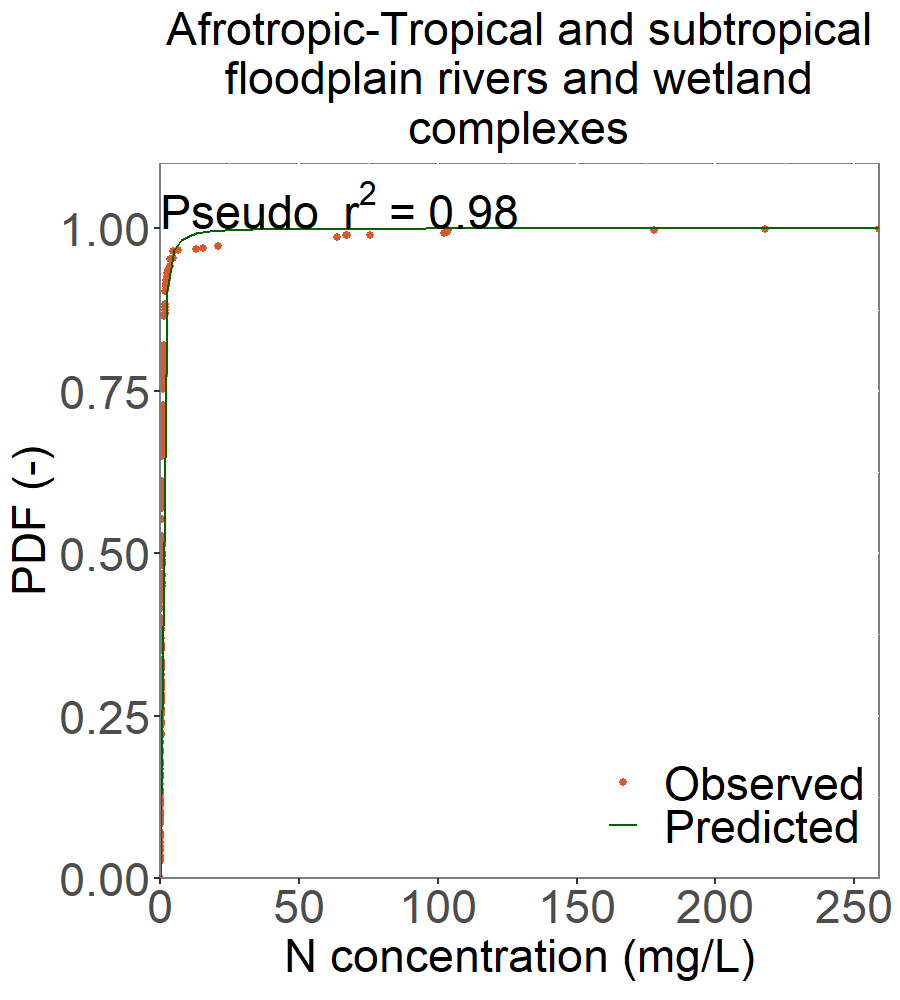

Supplement: Supplementary file 3 — es2c09333_si_003.zip [file es2c09333_si_003.zip › SSD_RMHT/Afrotropic-Tropical and subtropical floodplain rivers and wetland complexes.tif]

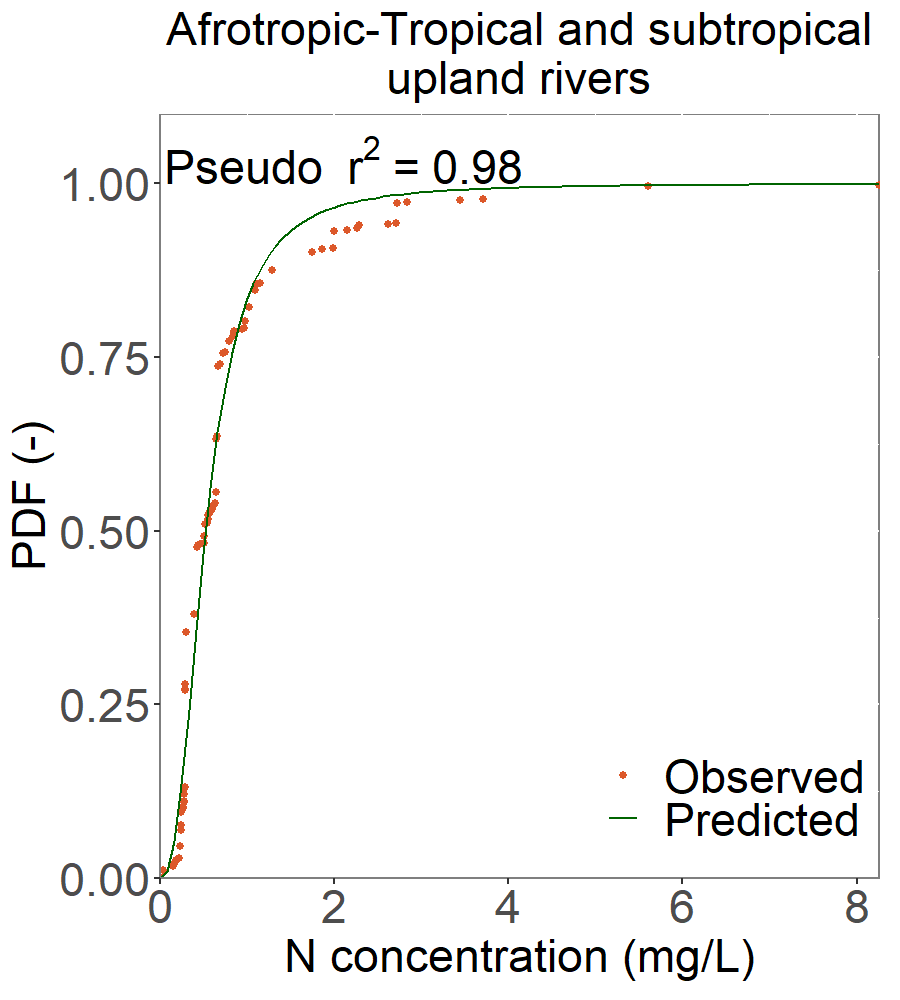

Supplement: Supplementary file 3 — es2c09333_si_003.zip [file es2c09333_si_003.zip › SSD_RMHT/Afrotropic-Tropical and subtropical upland rivers.tif]

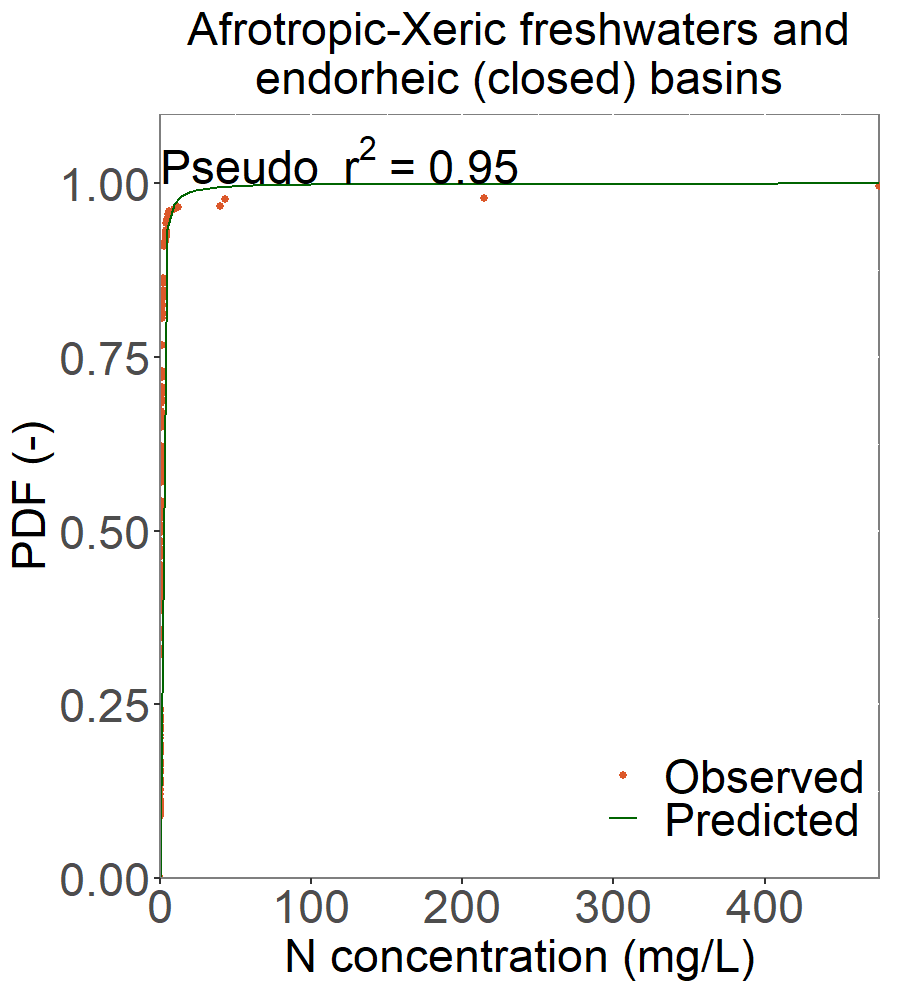

Supplement: Supplementary file 3 — es2c09333_si_003.zip [file es2c09333_si_003.zip › SSD_RMHT/Afrotropic-Xeric freshwaters and endorheic (closed) basins.tif]

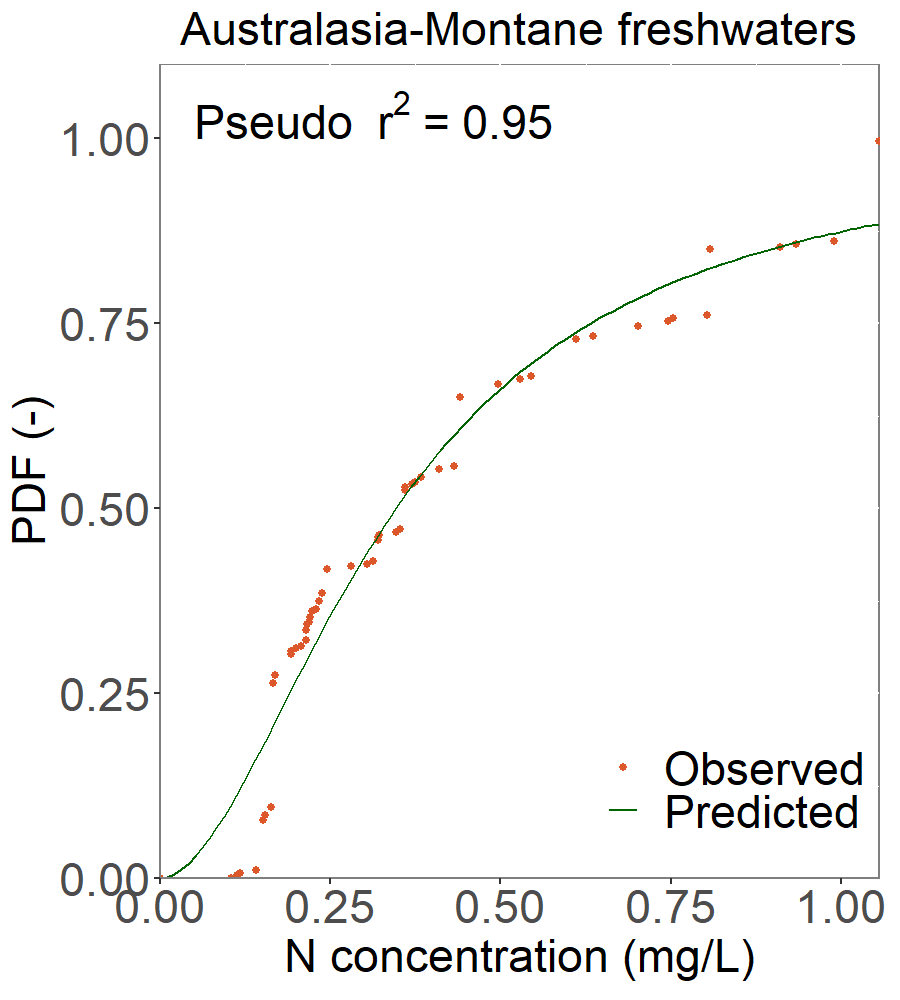

Supplement: Supplementary file 3 — es2c09333_si_003.zip [file es2c09333_si_003.zip › SSD_RMHT/Australasia-Montane freshwaters.tif]

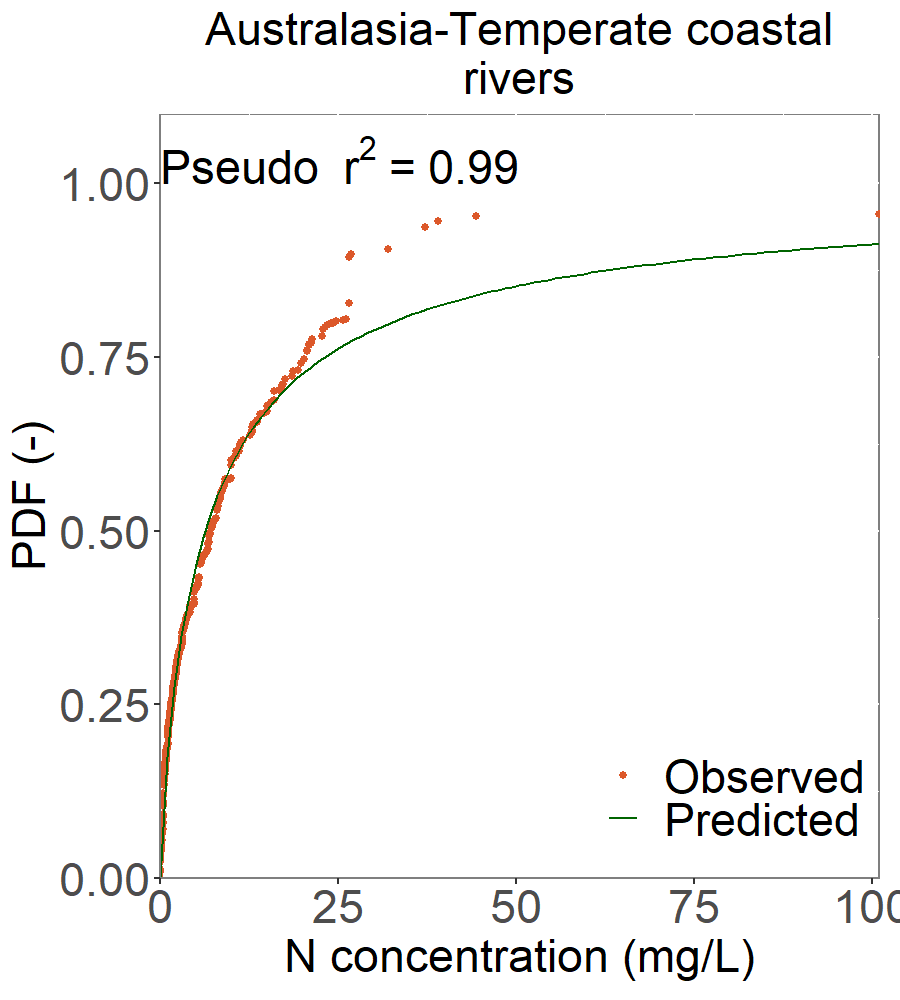

Supplement: Supplementary file 3 — es2c09333_si_003.zip [file es2c09333_si_003.zip › SSD_RMHT/Australasia-Temperate coastal rivers.tif]

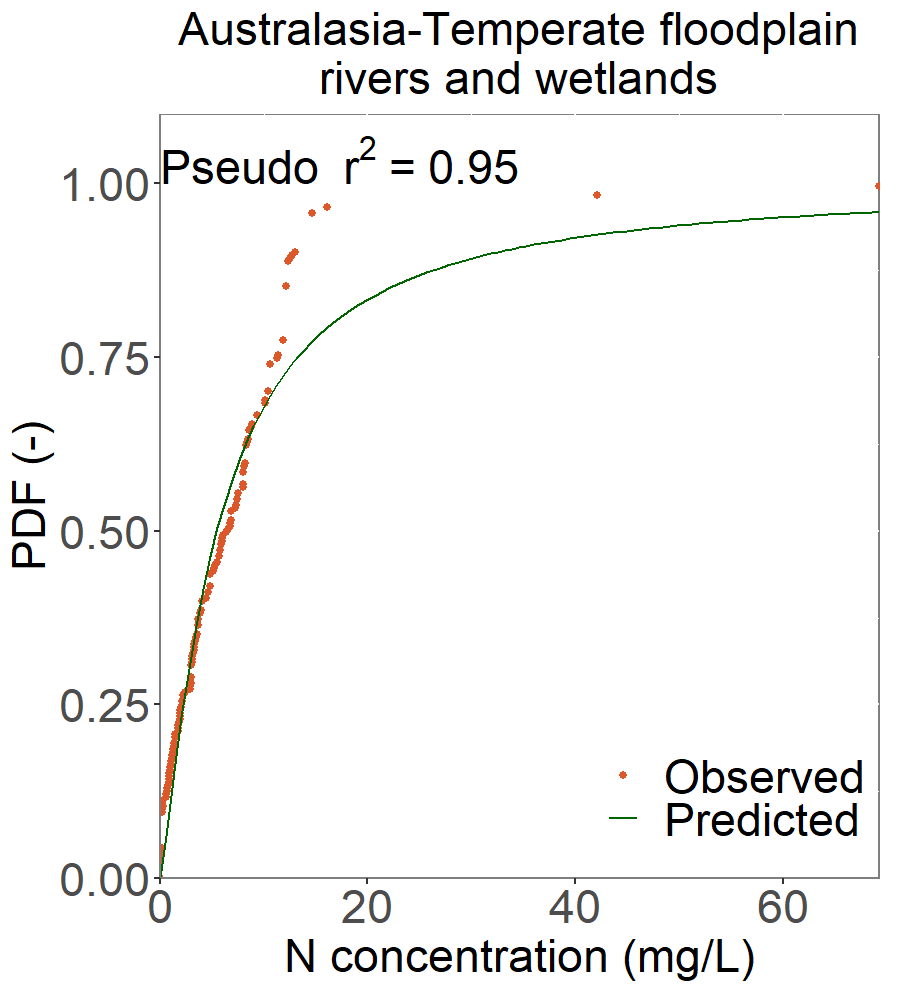

Supplement: Supplementary file 3 — es2c09333_si_003.zip [file es2c09333_si_003.zip › SSD_RMHT/Australasia-Temperate floodplain rivers and wetlands.tif]

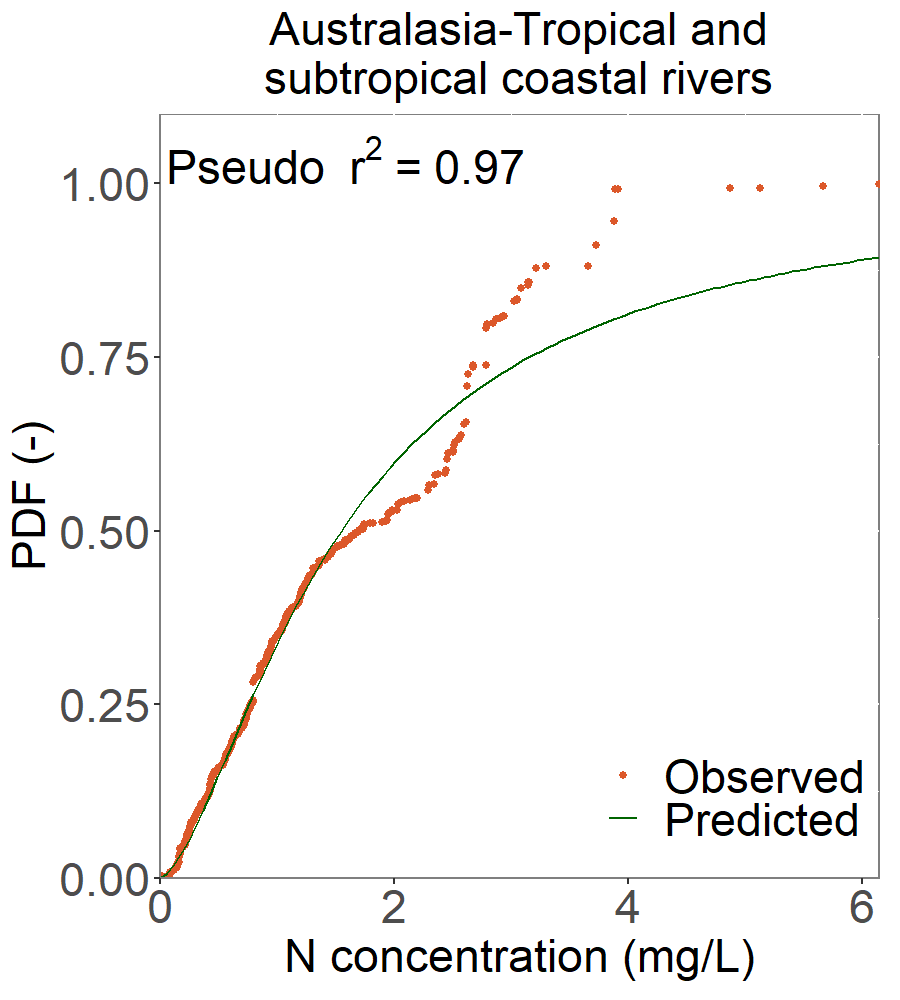

Supplement: Supplementary file 3 — es2c09333_si_003.zip [file es2c09333_si_003.zip › SSD_RMHT/Australasia-Tropical and subtropical coastal rivers.tif]

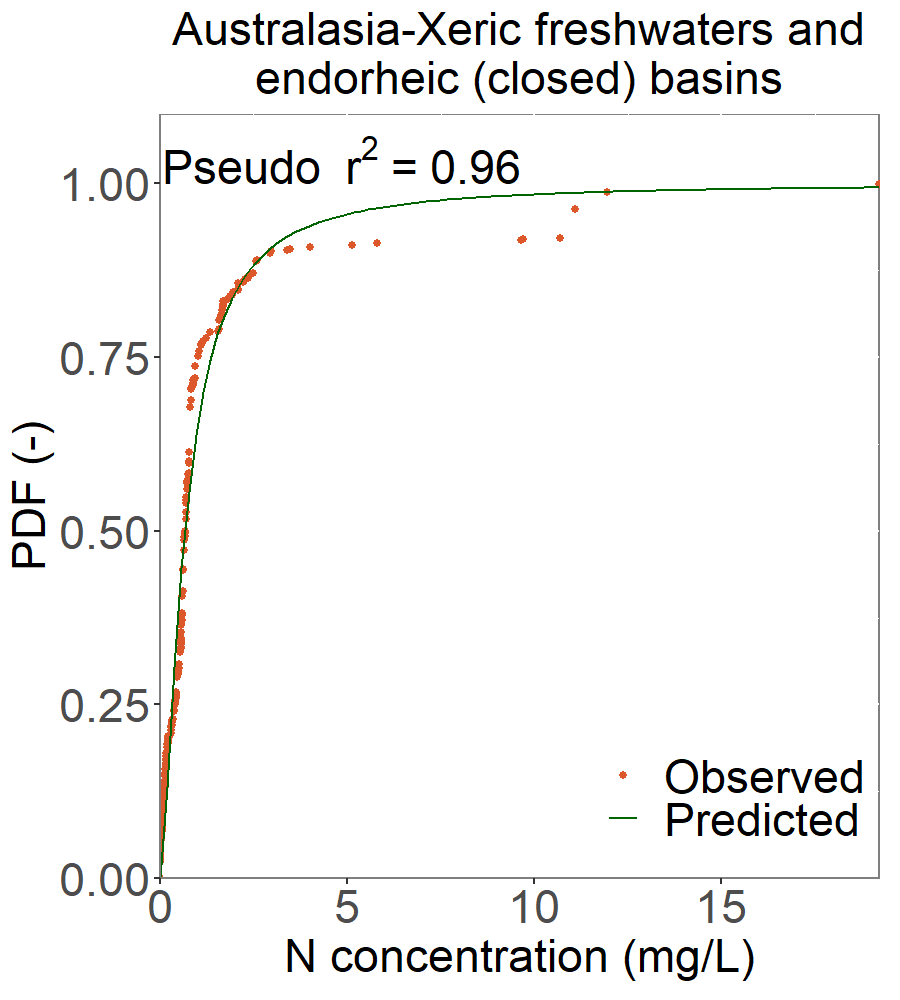

Supplement: Supplementary file 3 — es2c09333_si_003.zip [file es2c09333_si_003.zip › SSD_RMHT/Australasia-Xeric freshwaters and endorheic (closed) basins.tif]

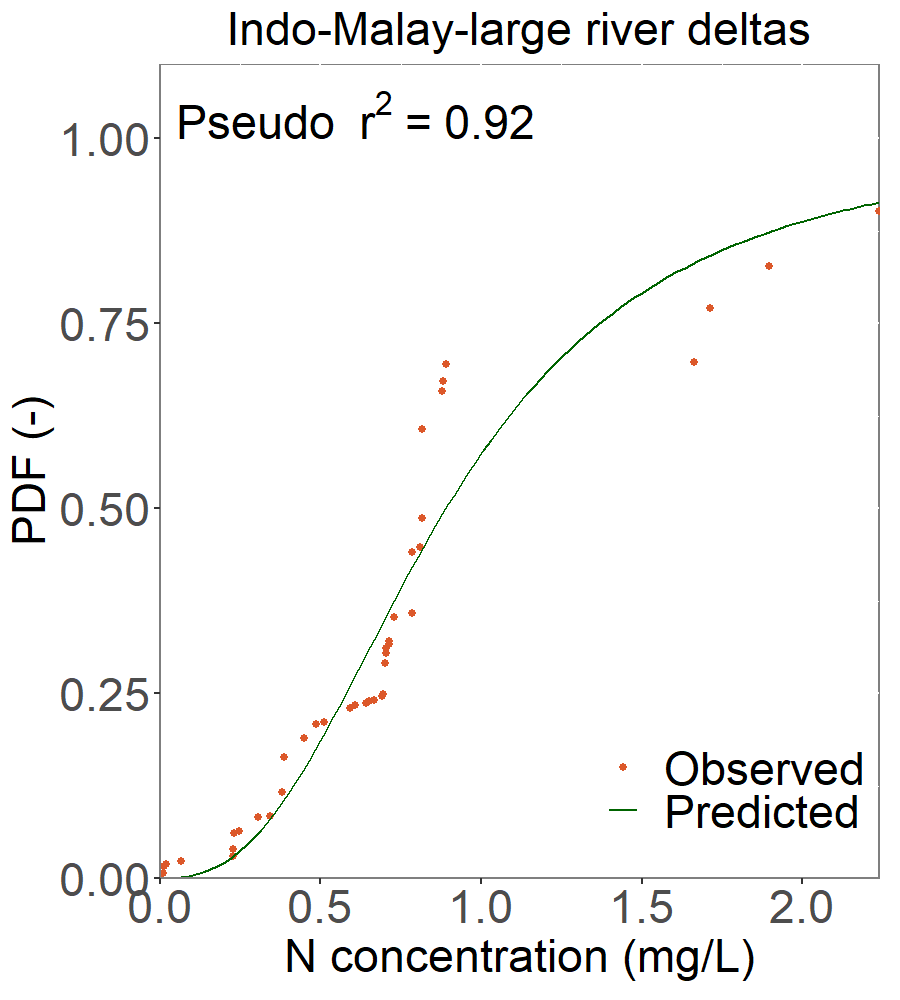

Supplement: Supplementary file 3 — es2c09333_si_003.zip [file es2c09333_si_003.zip › SSD_RMHT/Indo-Malay-large river deltas.tif]

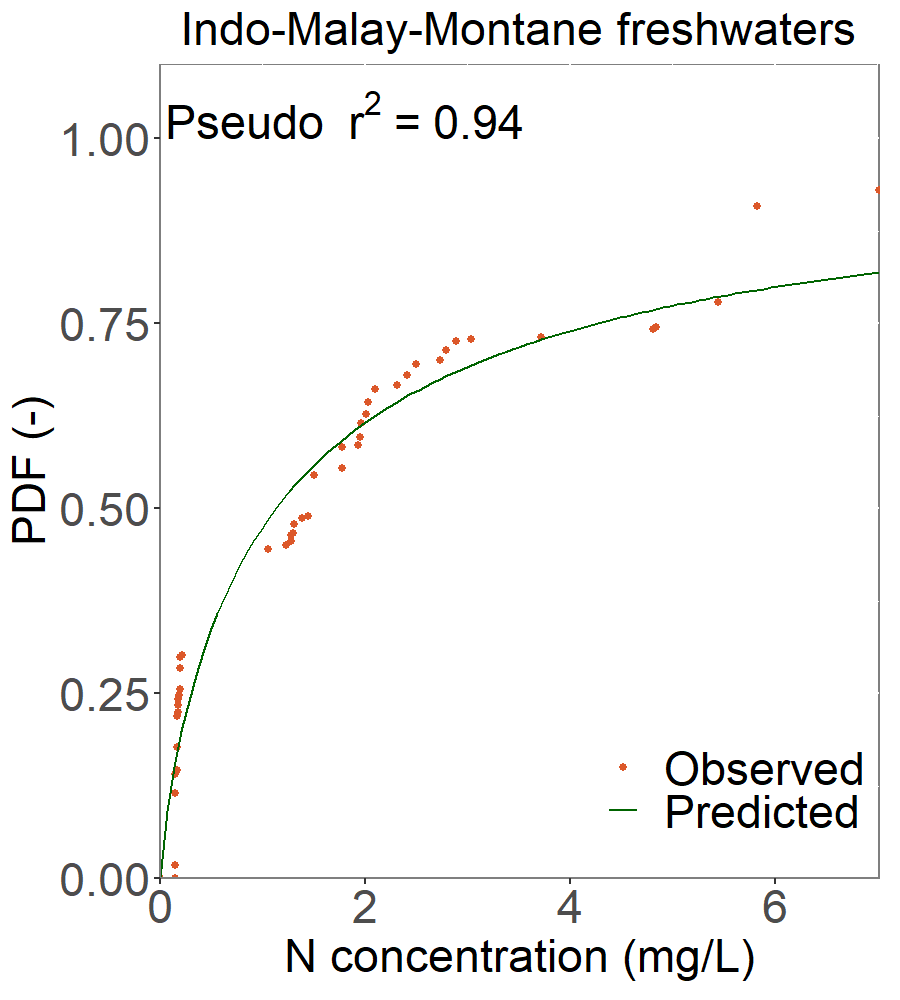

Supplement: Supplementary file 3 — es2c09333_si_003.zip [file es2c09333_si_003.zip › SSD_RMHT/Indo-Malay-Montane freshwaters.tif]

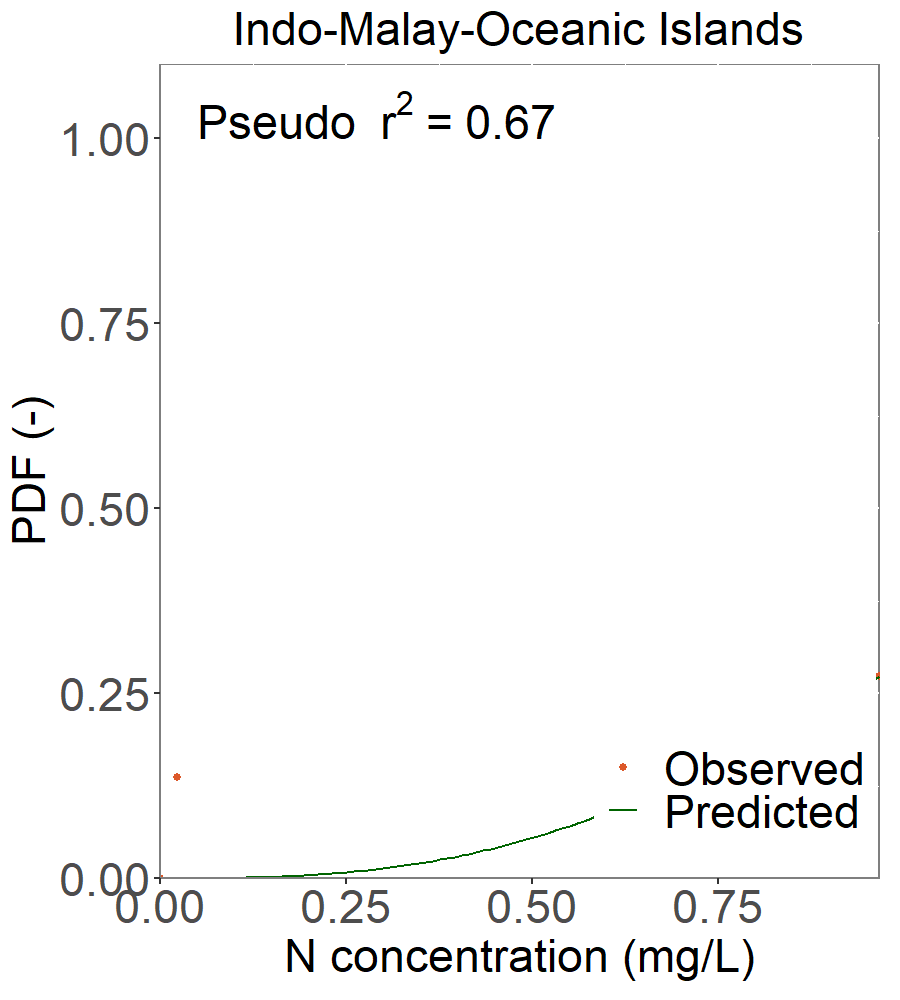

Supplement: Supplementary file 3 — es2c09333_si_003.zip [file es2c09333_si_003.zip › SSD_RMHT/Indo-Malay-Oceanic Islands.tif]

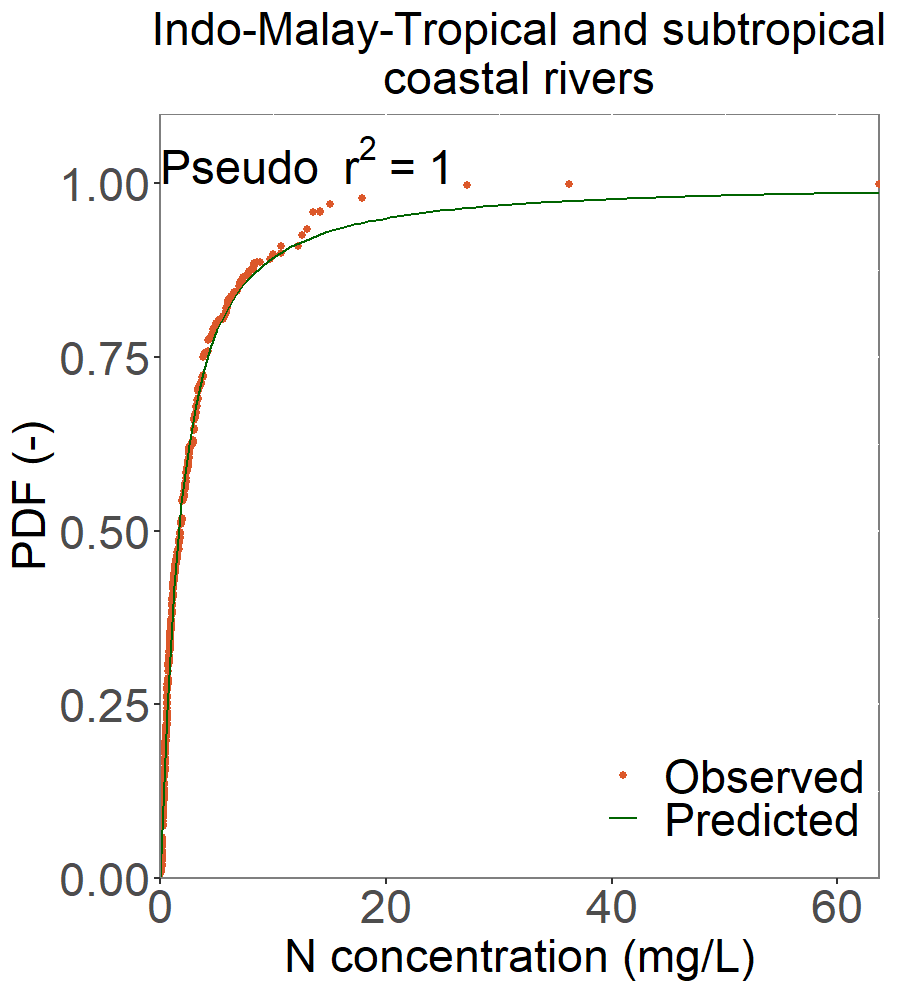

Supplement: Supplementary file 3 — es2c09333_si_003.zip [file es2c09333_si_003.zip › SSD_RMHT/Indo-Malay-Tropical and subtropical coastal rivers.tif]

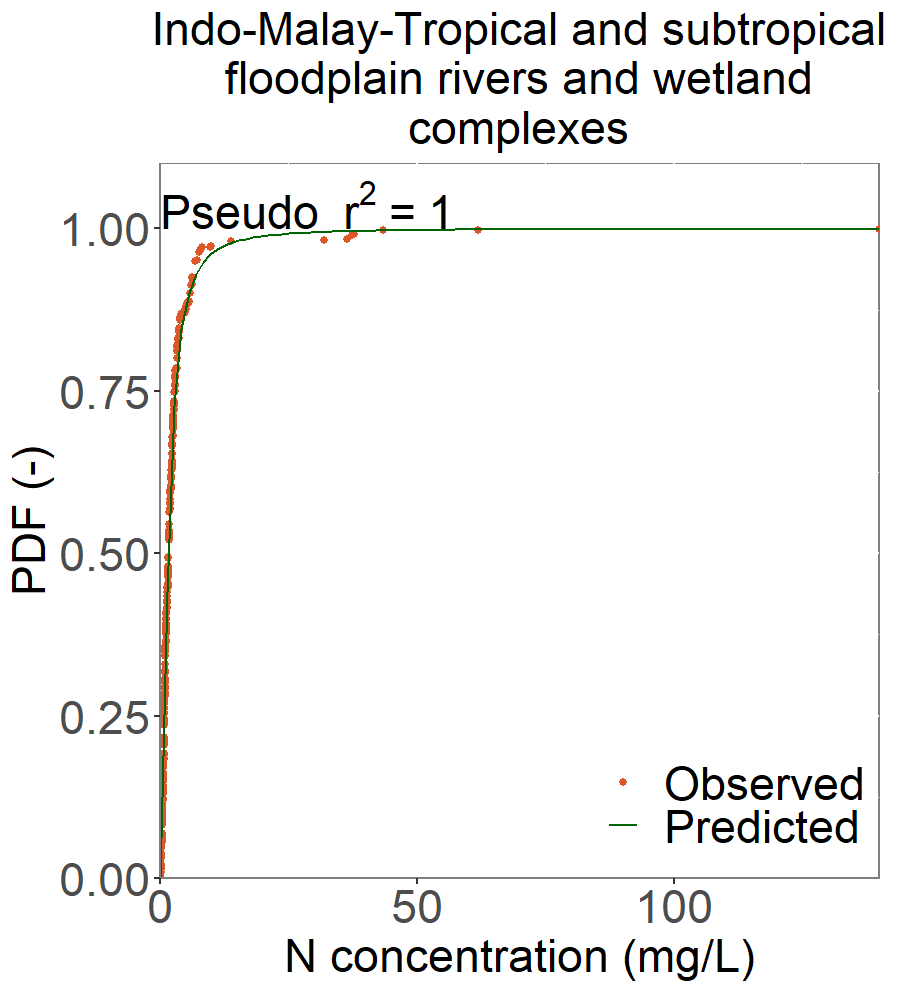

Supplement: Supplementary file 3 — es2c09333_si_003.zip [file es2c09333_si_003.zip › SSD_RMHT/Indo-Malay-Tropical and subtropical floodplain rivers and wetland complexes.tif]

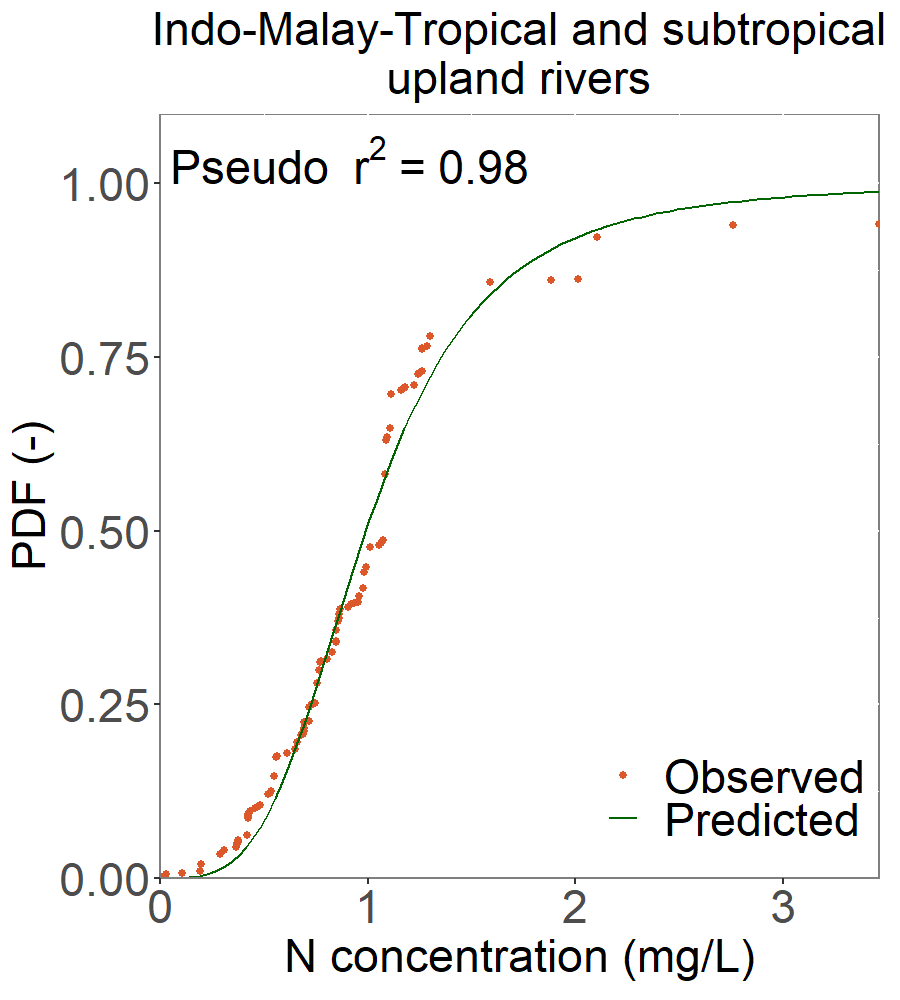

Supplement: Supplementary file 3 — es2c09333_si_003.zip [file es2c09333_si_003.zip › SSD_RMHT/Indo-Malay-Tropical and subtropical upland rivers.tif]

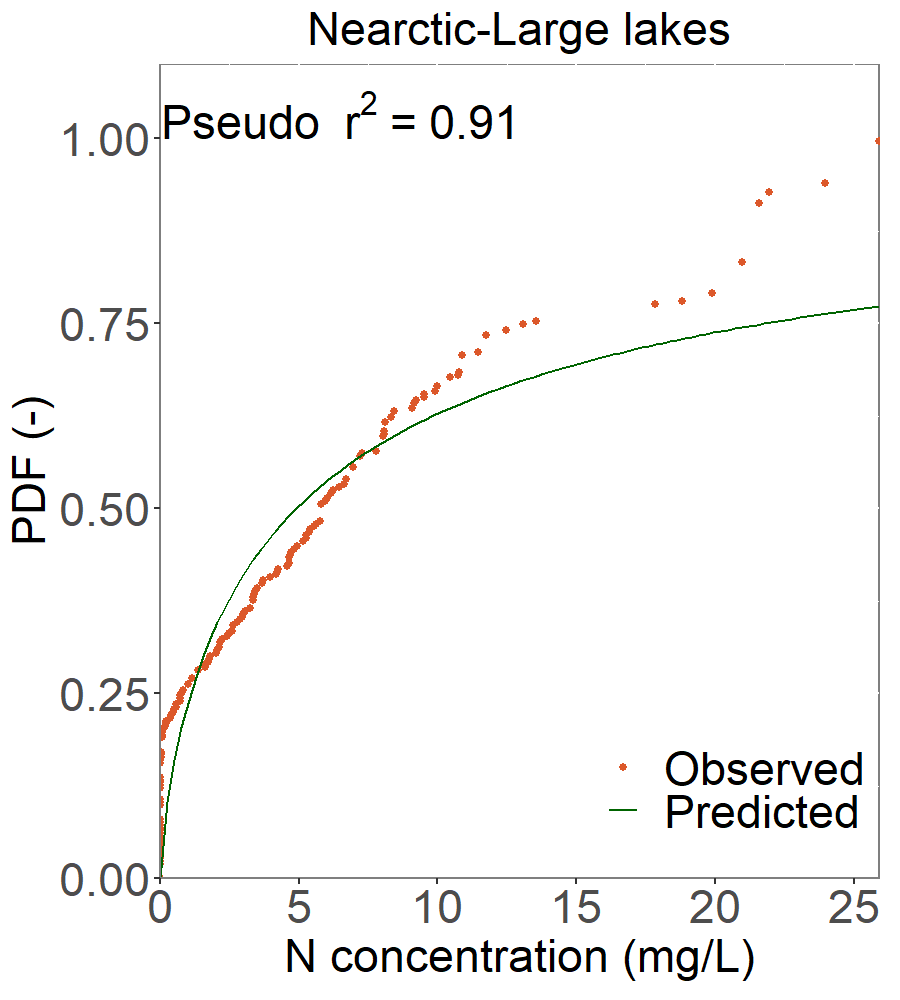

Supplement: Supplementary file 3 — es2c09333_si_003.zip [file es2c09333_si_003.zip › SSD_RMHT/Nearctic-Large lakes.tif]

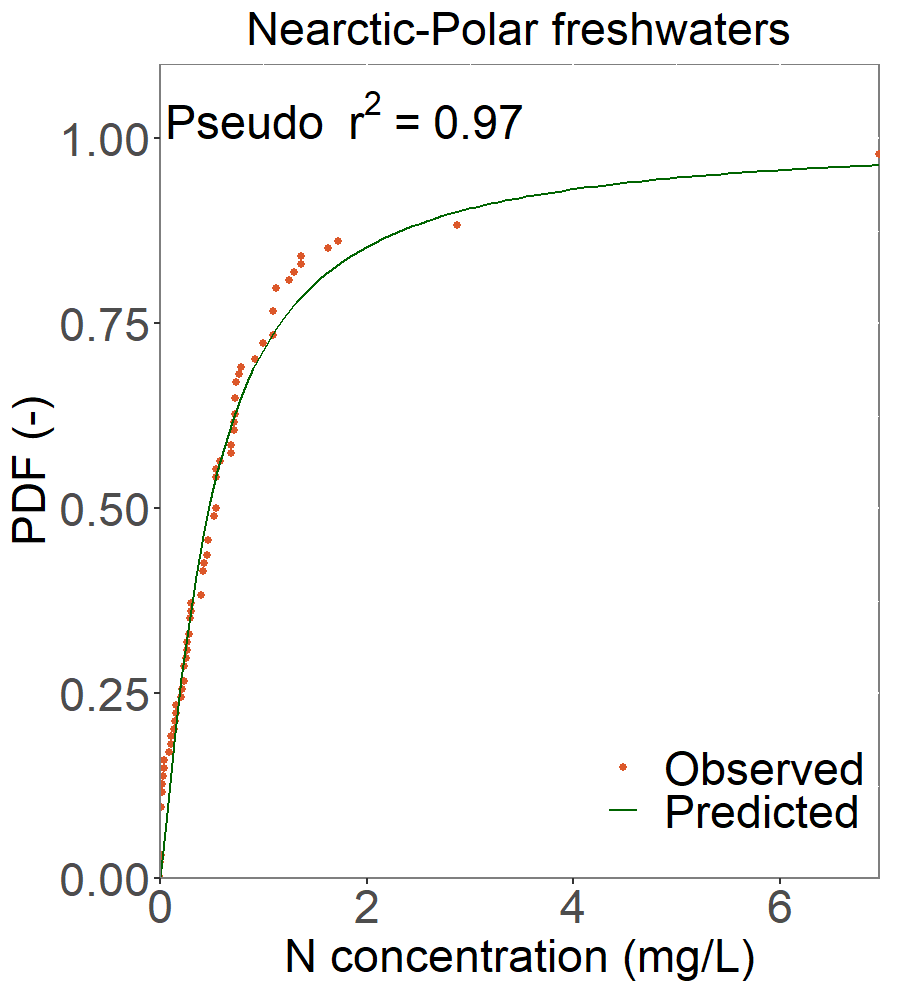

Supplement: Supplementary file 3 — es2c09333_si_003.zip [file es2c09333_si_003.zip › SSD_RMHT/Nearctic-Polar freshwaters.tif]

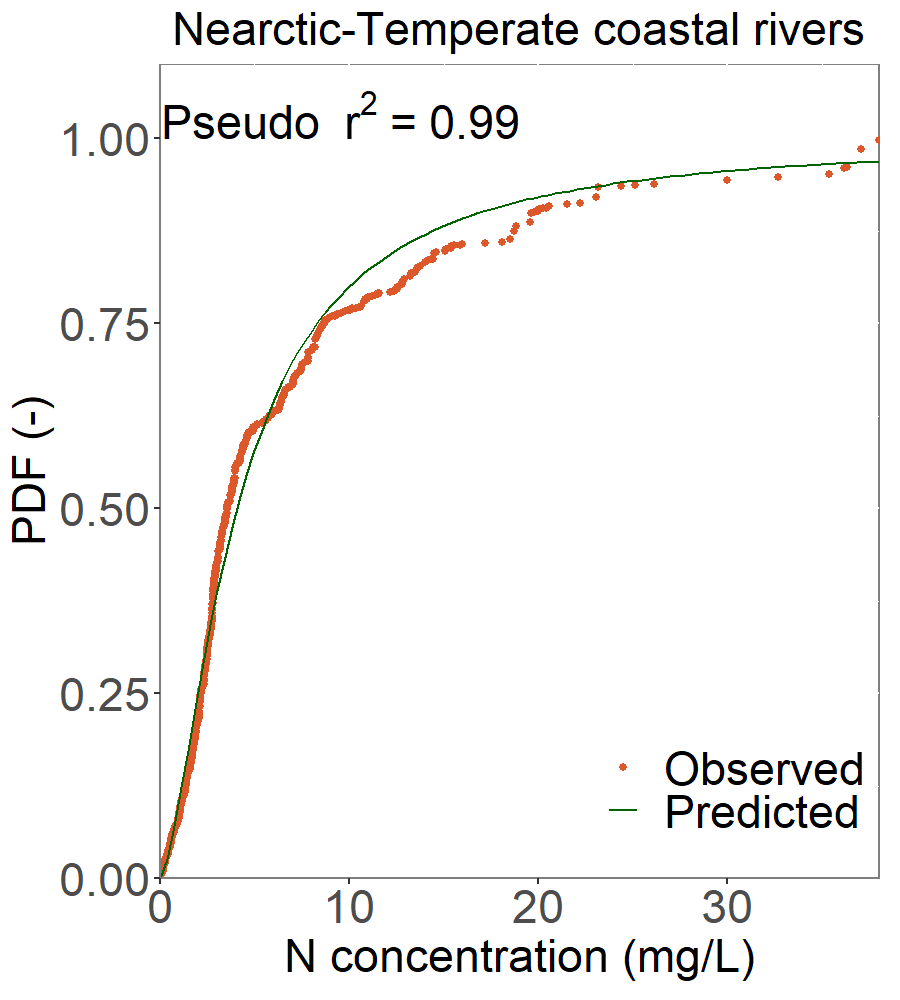

Supplement: Supplementary file 3 — es2c09333_si_003.zip [file es2c09333_si_003.zip › SSD_RMHT/Nearctic-Temperate coastal rivers.tif]

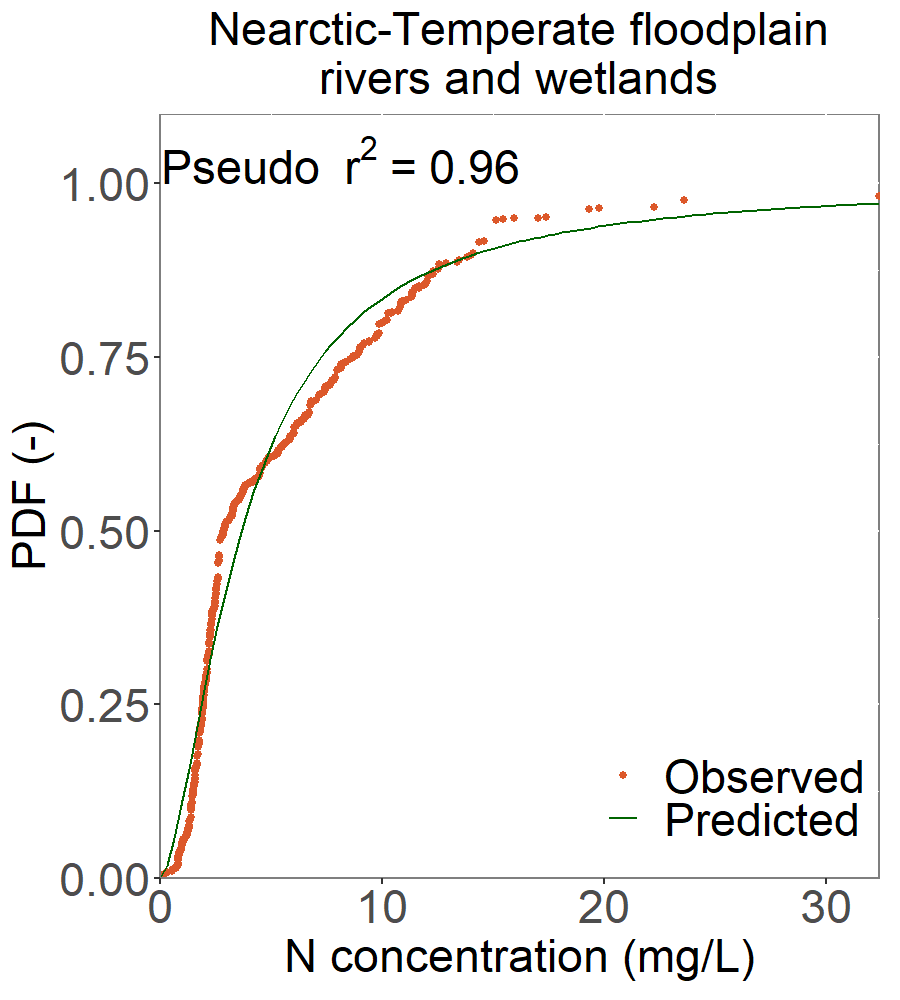

Supplement: Supplementary file 3 — es2c09333_si_003.zip [file es2c09333_si_003.zip › SSD_RMHT/Nearctic-Temperate floodplain rivers and wetlands.tif]

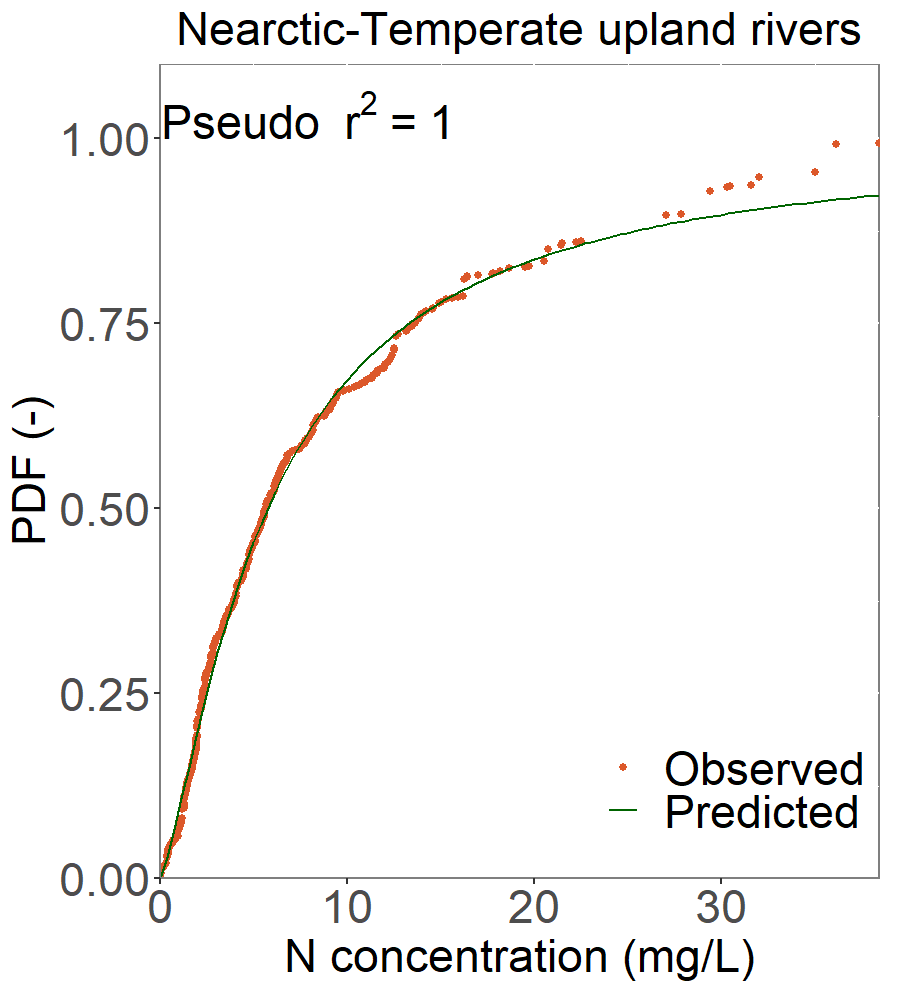

Supplement: Supplementary file 3 — es2c09333_si_003.zip [file es2c09333_si_003.zip › SSD_RMHT/Nearctic-Temperate upland rivers.tif]

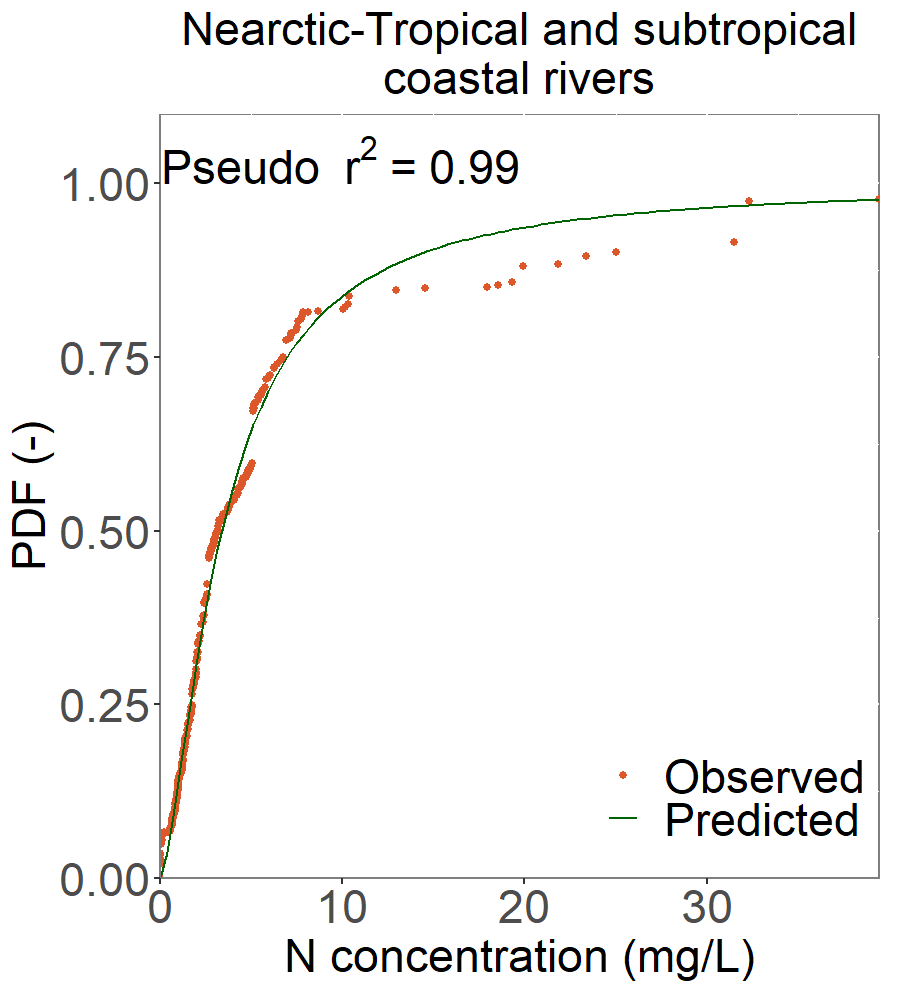

Supplement: Supplementary file 3 — es2c09333_si_003.zip [file es2c09333_si_003.zip › SSD_RMHT/Nearctic-Tropical and subtropical coastal rivers.tif]

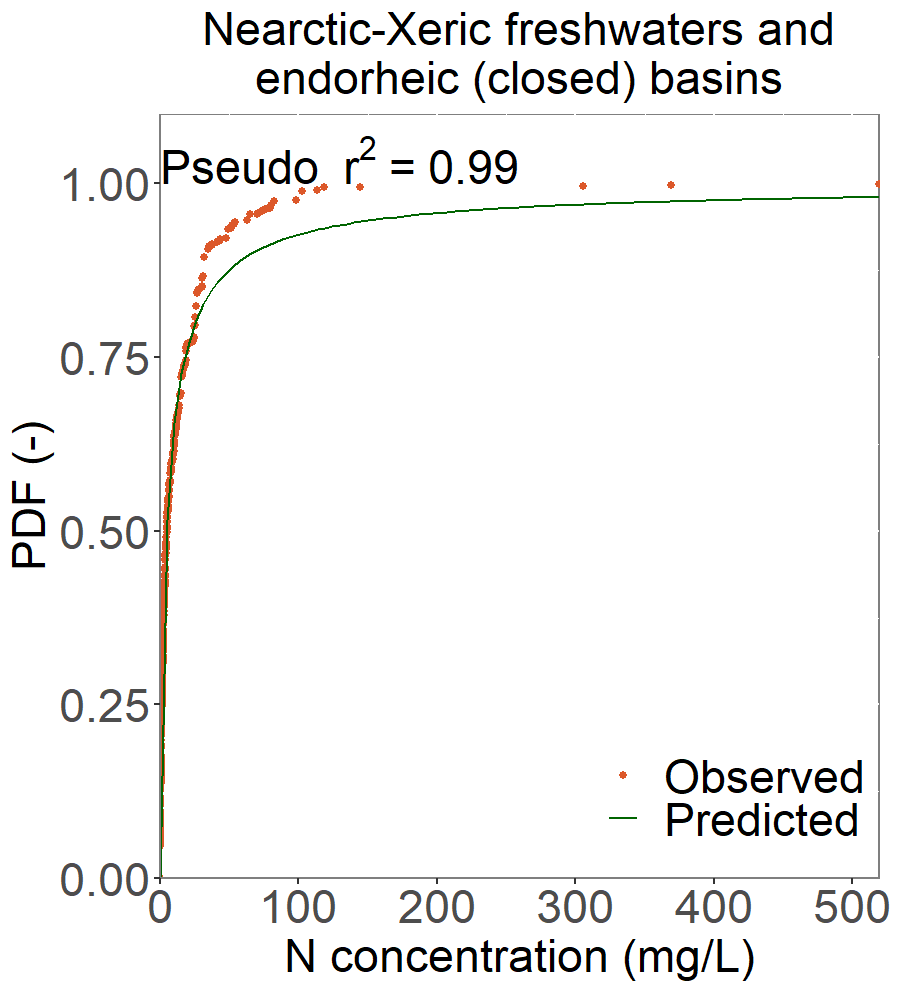

Supplement: Supplementary file 3 — es2c09333_si_003.zip [file es2c09333_si_003.zip › SSD_RMHT/Nearctic-Xeric freshwaters and endorheic (closed) basins.tif]

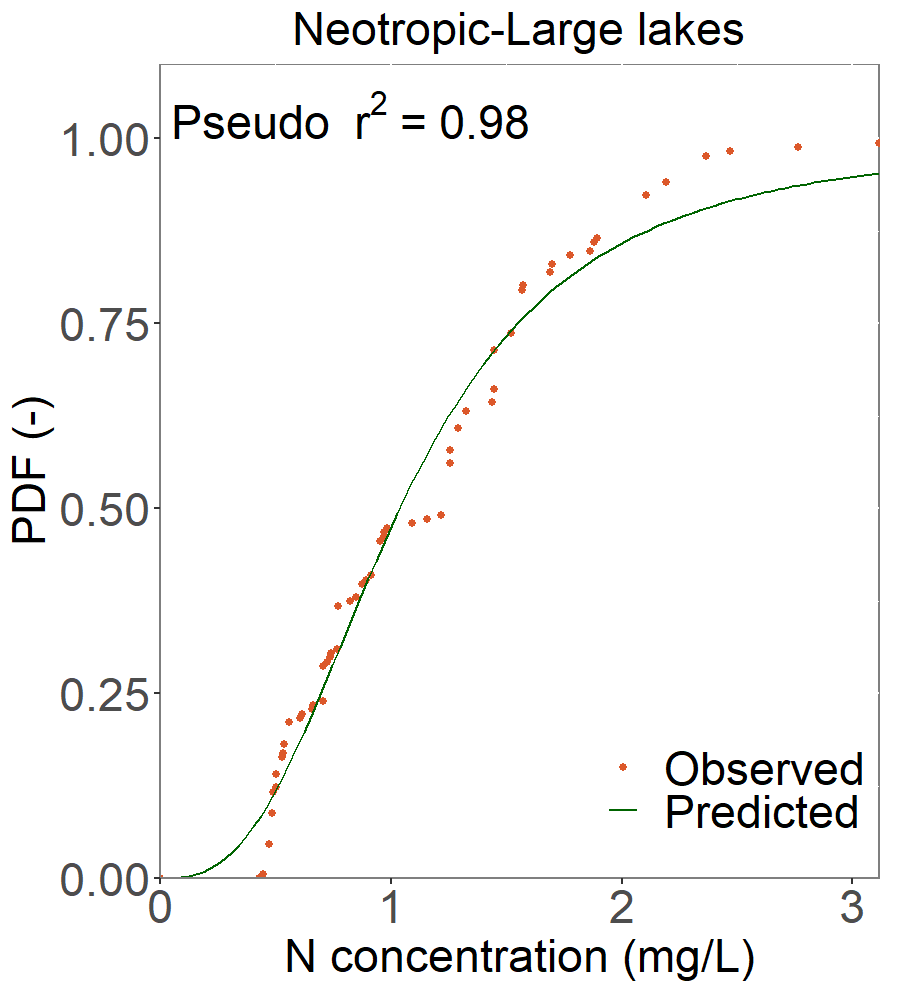

Supplement: Supplementary file 3 — es2c09333_si_003.zip [file es2c09333_si_003.zip › SSD_RMHT/Neotropic-Large lakes.tif]

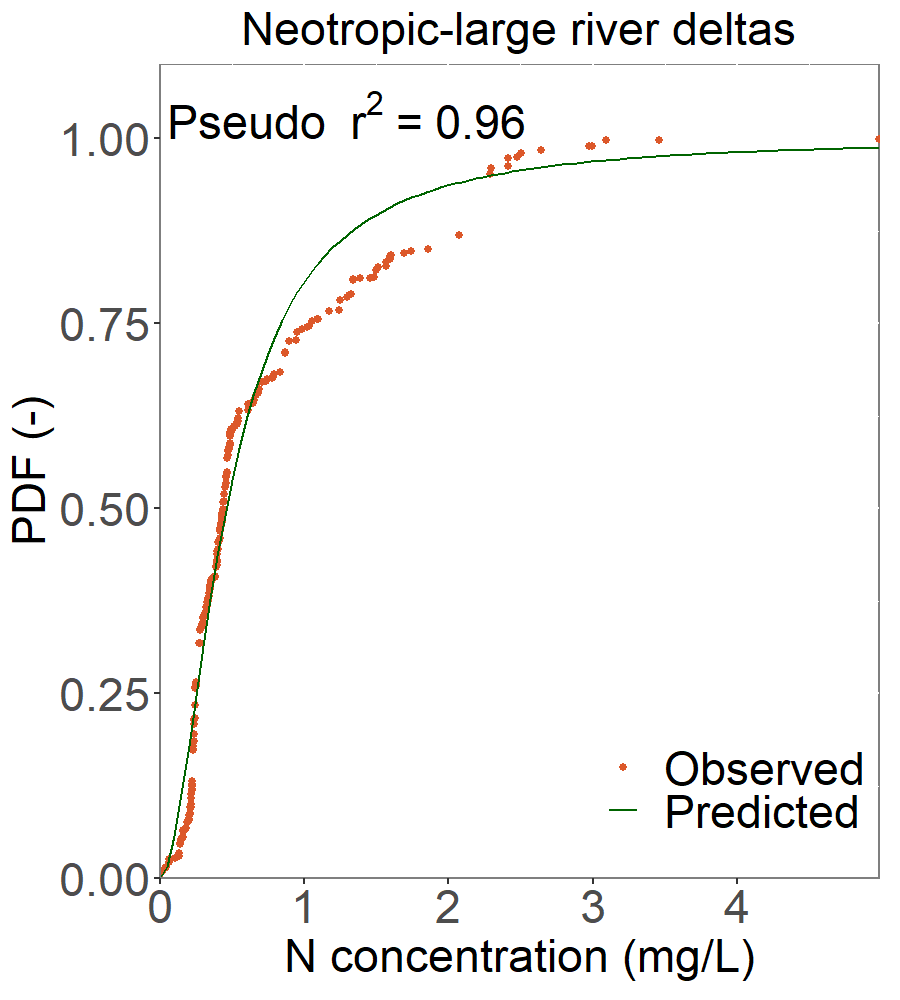

Supplement: Supplementary file 3 — es2c09333_si_003.zip [file es2c09333_si_003.zip › SSD_RMHT/Neotropic-large river deltas.tif]

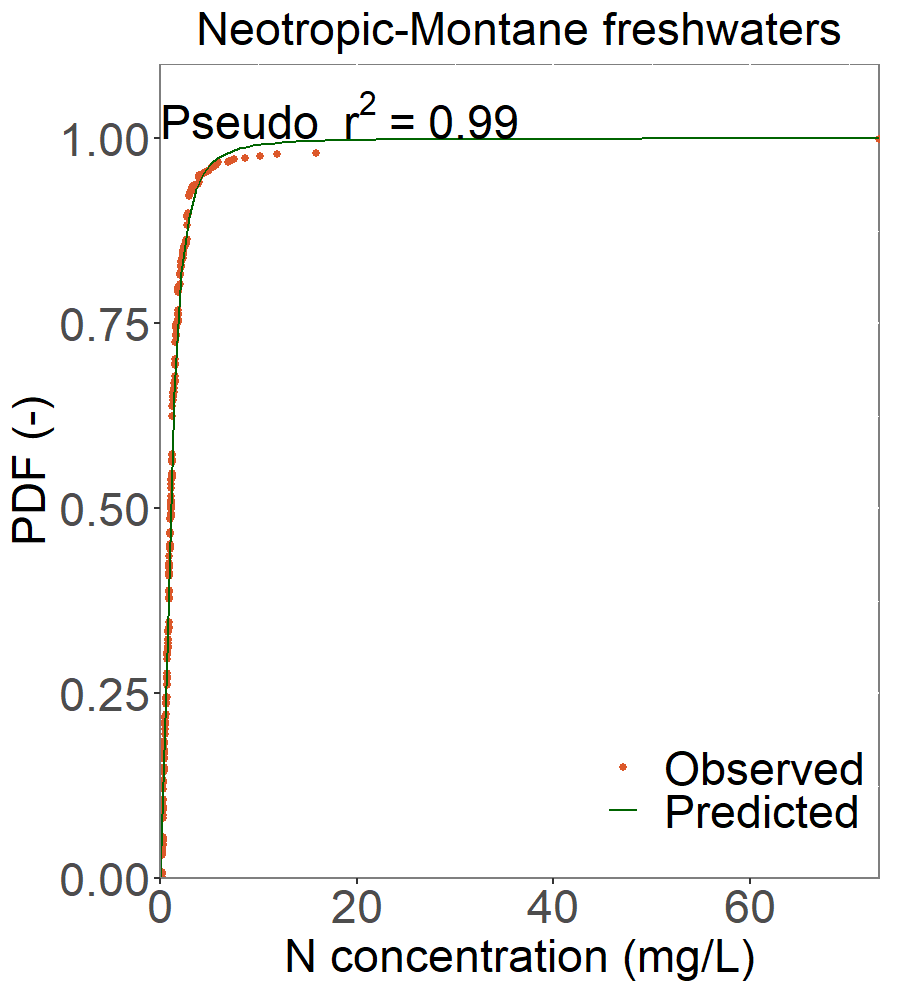

Supplement: Supplementary file 3 — es2c09333_si_003.zip [file es2c09333_si_003.zip › SSD_RMHT/Neotropic-Montane freshwaters.tif]

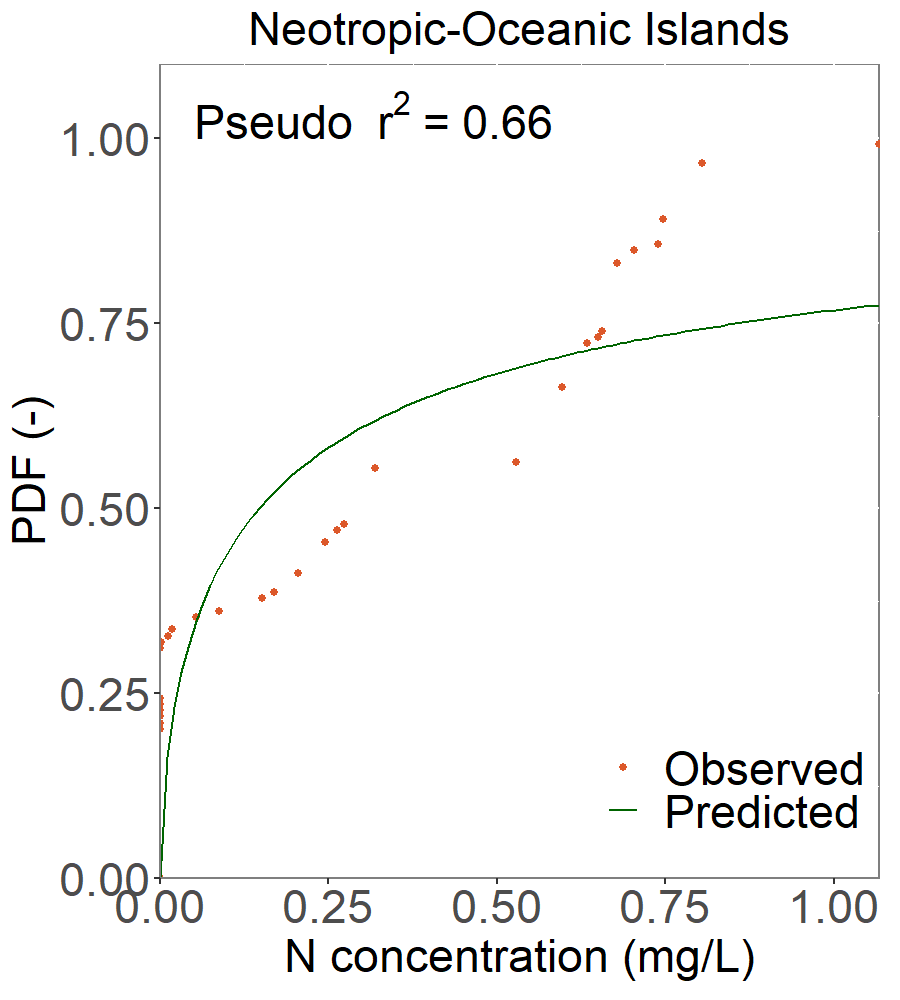

Supplement: Supplementary file 3 — es2c09333_si_003.zip [file es2c09333_si_003.zip › SSD_RMHT/Neotropic-Oceanic Islands.tif]

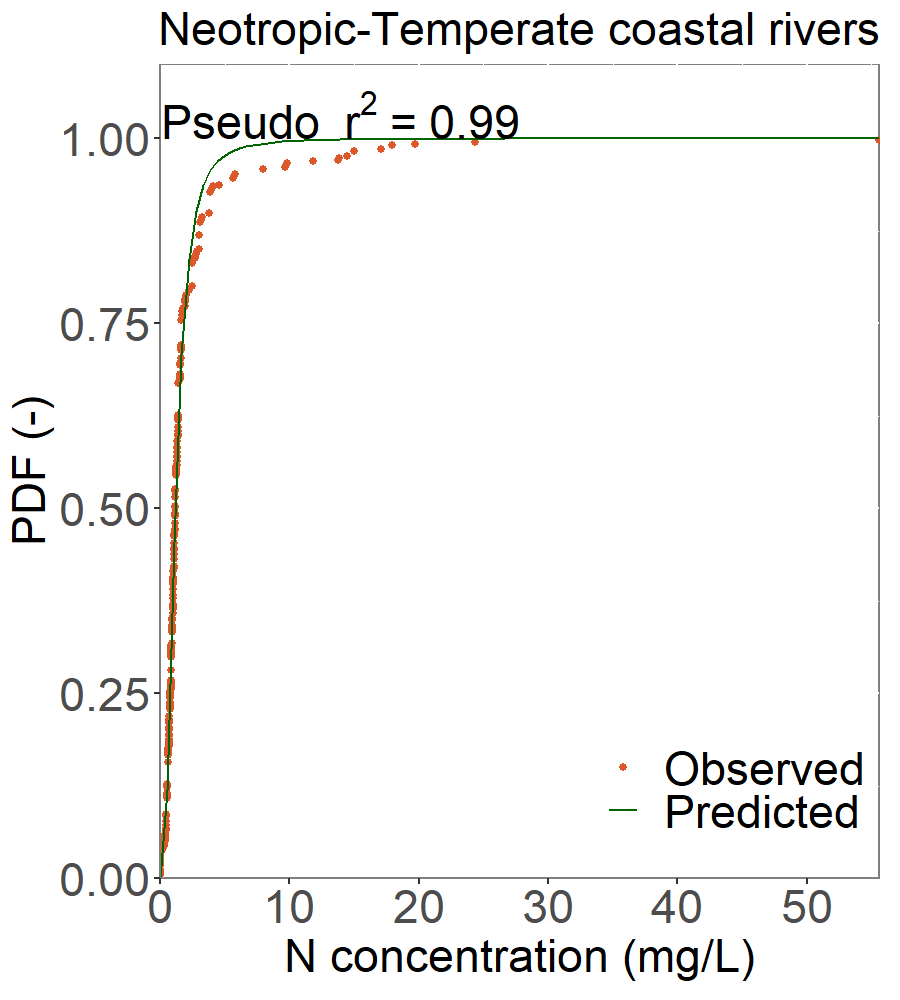

Supplement: Supplementary file 3 — es2c09333_si_003.zip [file es2c09333_si_003.zip › SSD_RMHT/Neotropic-Temperate coastal rivers.tif]

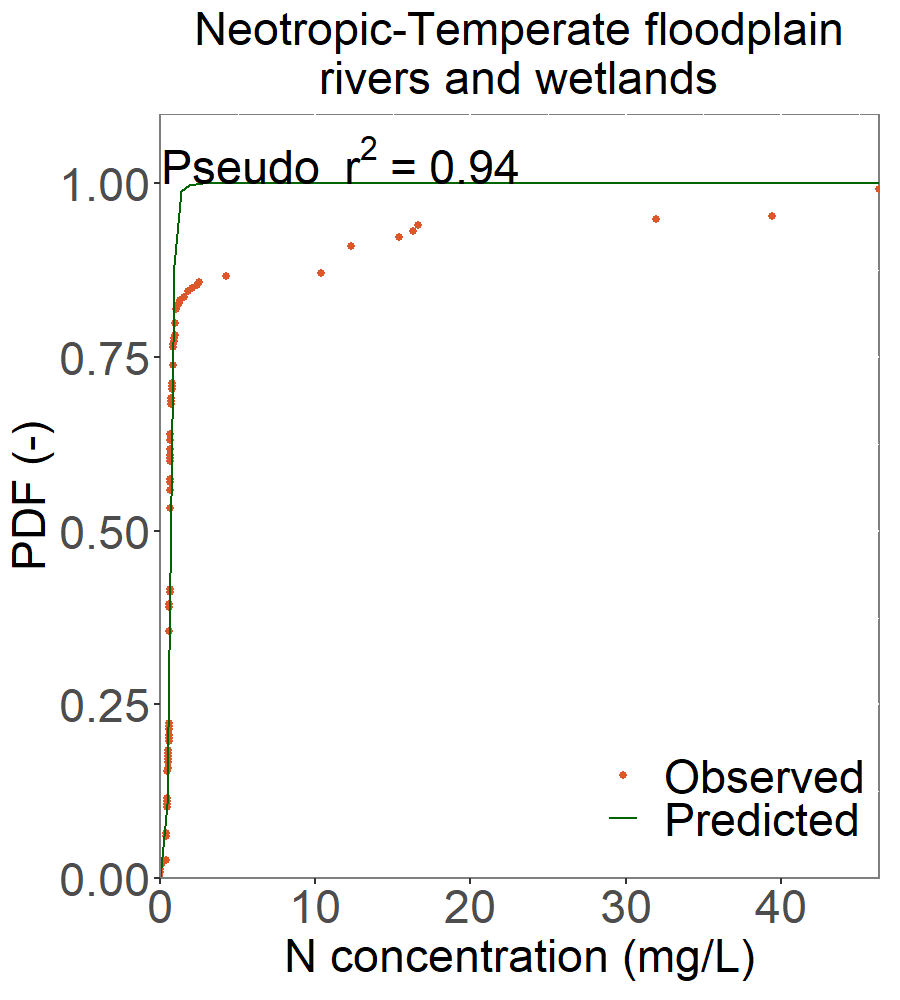

Supplement: Supplementary file 3 — es2c09333_si_003.zip [file es2c09333_si_003.zip › SSD_RMHT/Neotropic-Temperate floodplain rivers and wetlands.tif]

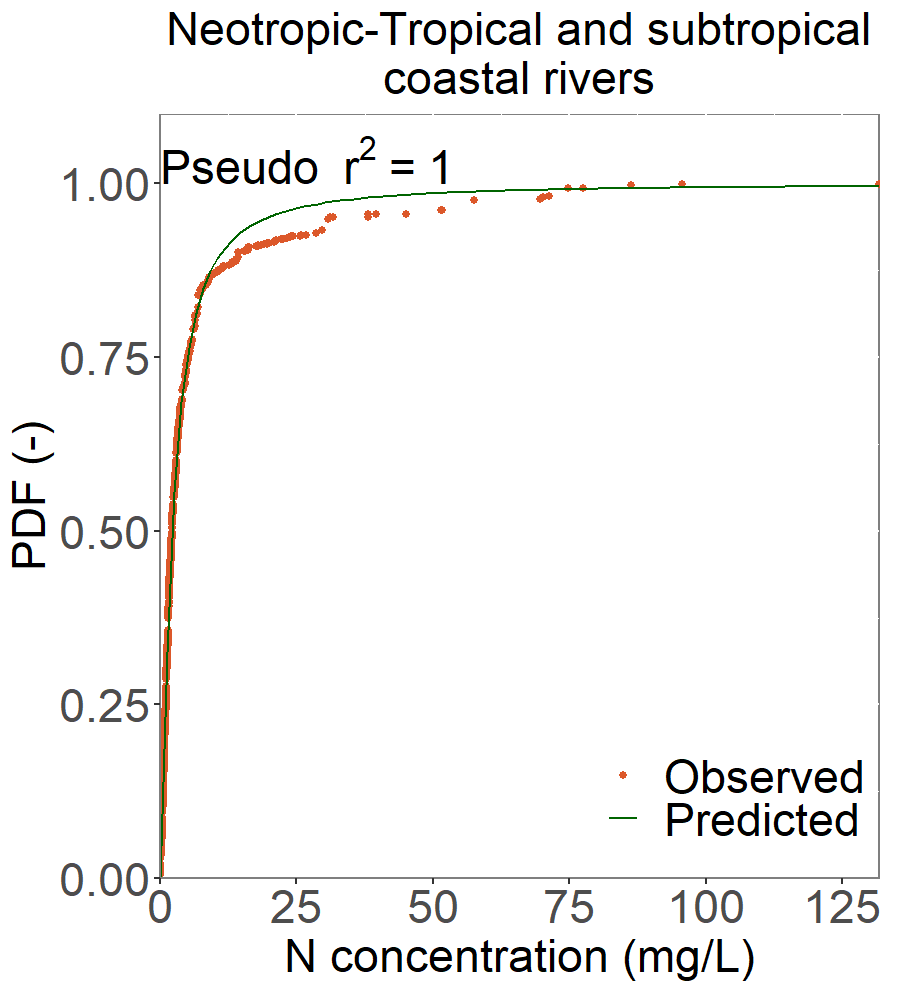

Supplement: Supplementary file 3 — es2c09333_si_003.zip [file es2c09333_si_003.zip › SSD_RMHT/Neotropic-Tropical and subtropical coastal rivers.tif]

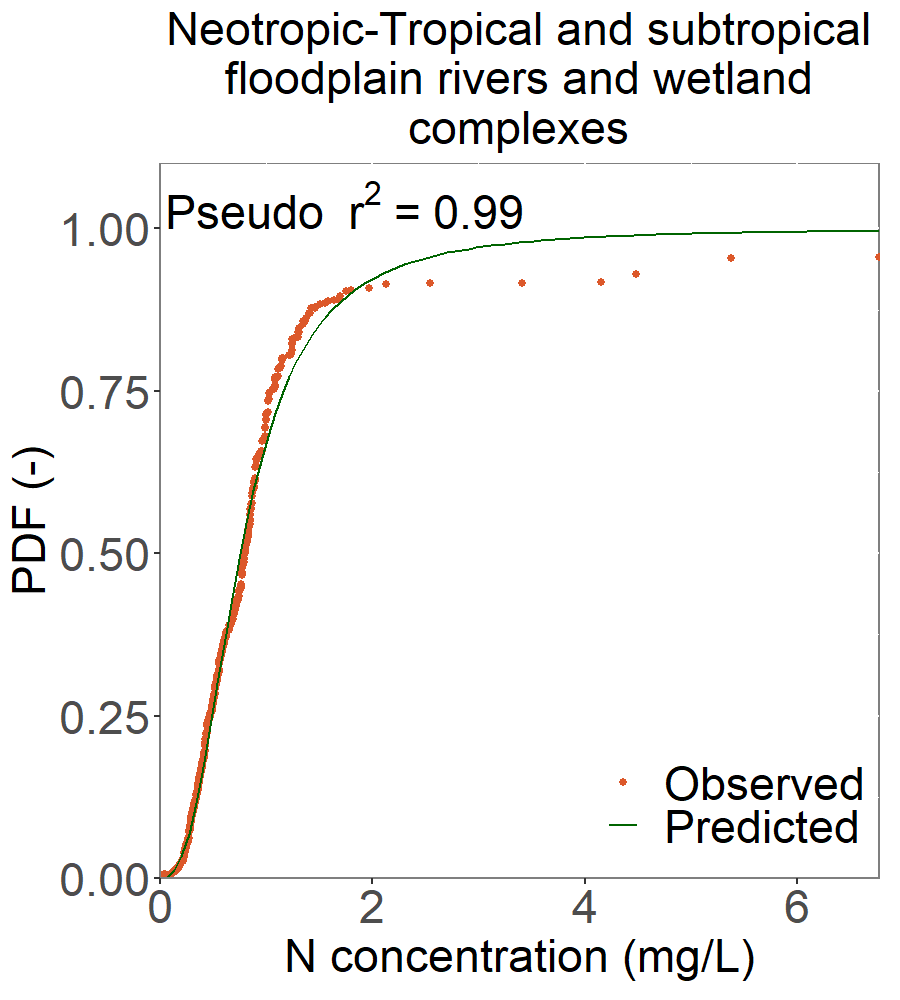

Supplement: Supplementary file 3 — es2c09333_si_003.zip [file es2c09333_si_003.zip › SSD_RMHT/Neotropic-Tropical and subtropical floodplain rivers and wetland complexes.tif]

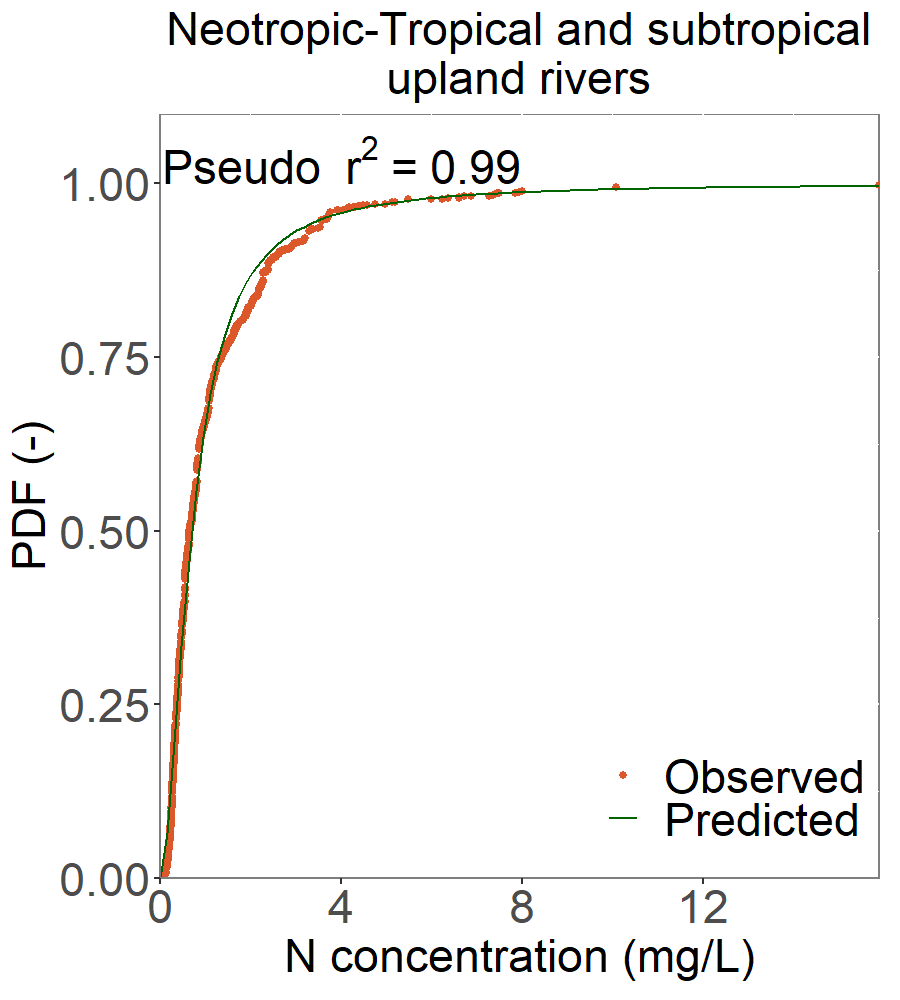

Supplement: Supplementary file 3 — es2c09333_si_003.zip [file es2c09333_si_003.zip › SSD_RMHT/Neotropic-Tropical and subtropical upland rivers.tif]

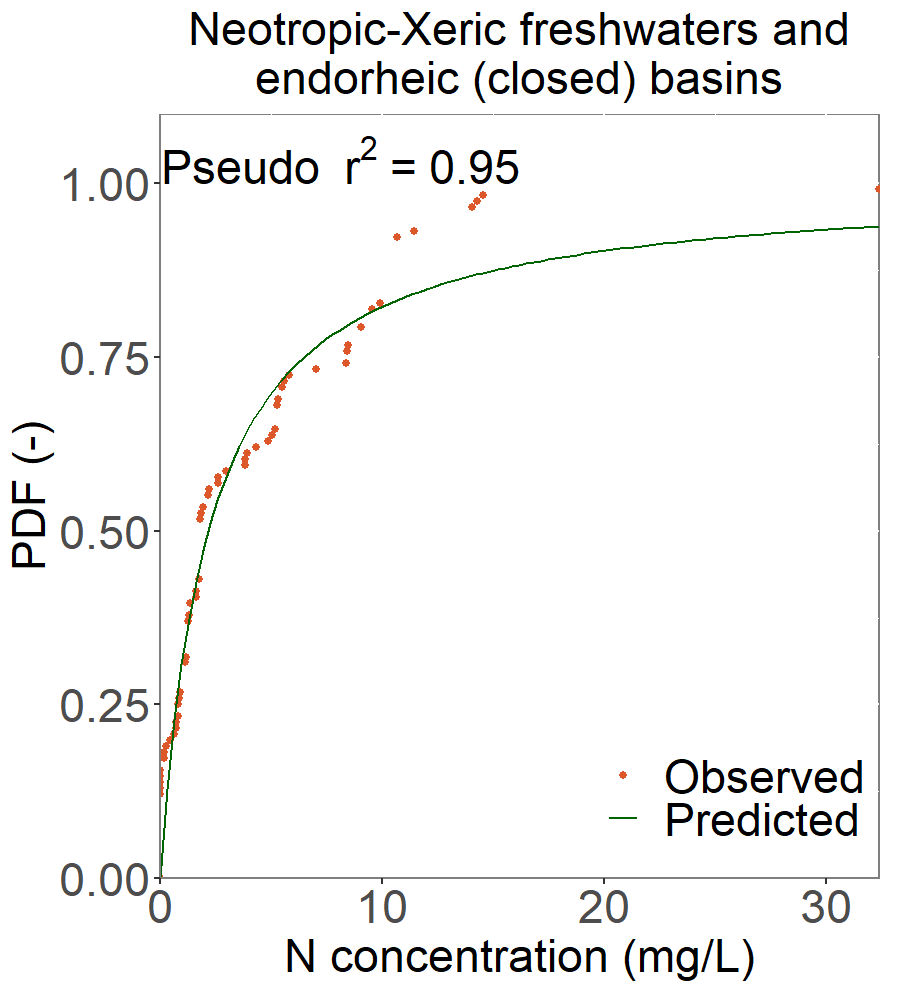

Supplement: Supplementary file 3 — es2c09333_si_003.zip [file es2c09333_si_003.zip › SSD_RMHT/Neotropic-Xeric freshwaters and endorheic (closed) basins.tif]

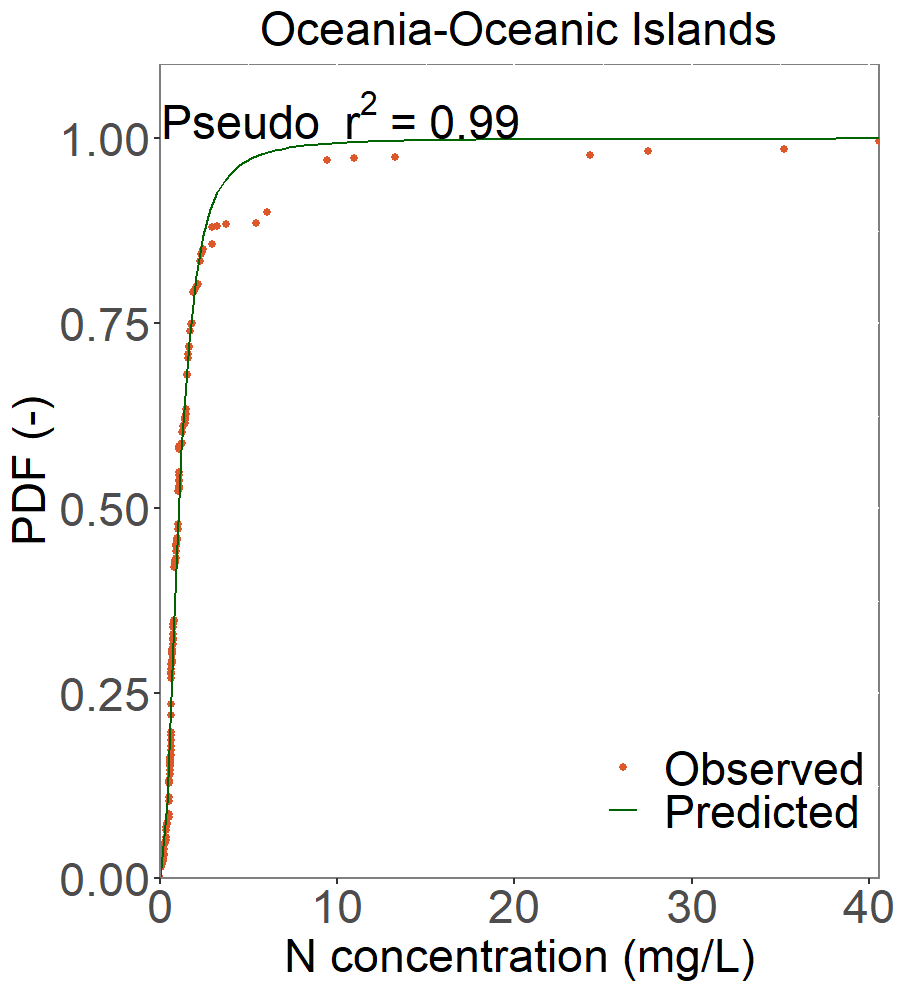

Supplement: Supplementary file 3 — es2c09333_si_003.zip [file es2c09333_si_003.zip › SSD_RMHT/Oceania-Oceanic Islands.tif]

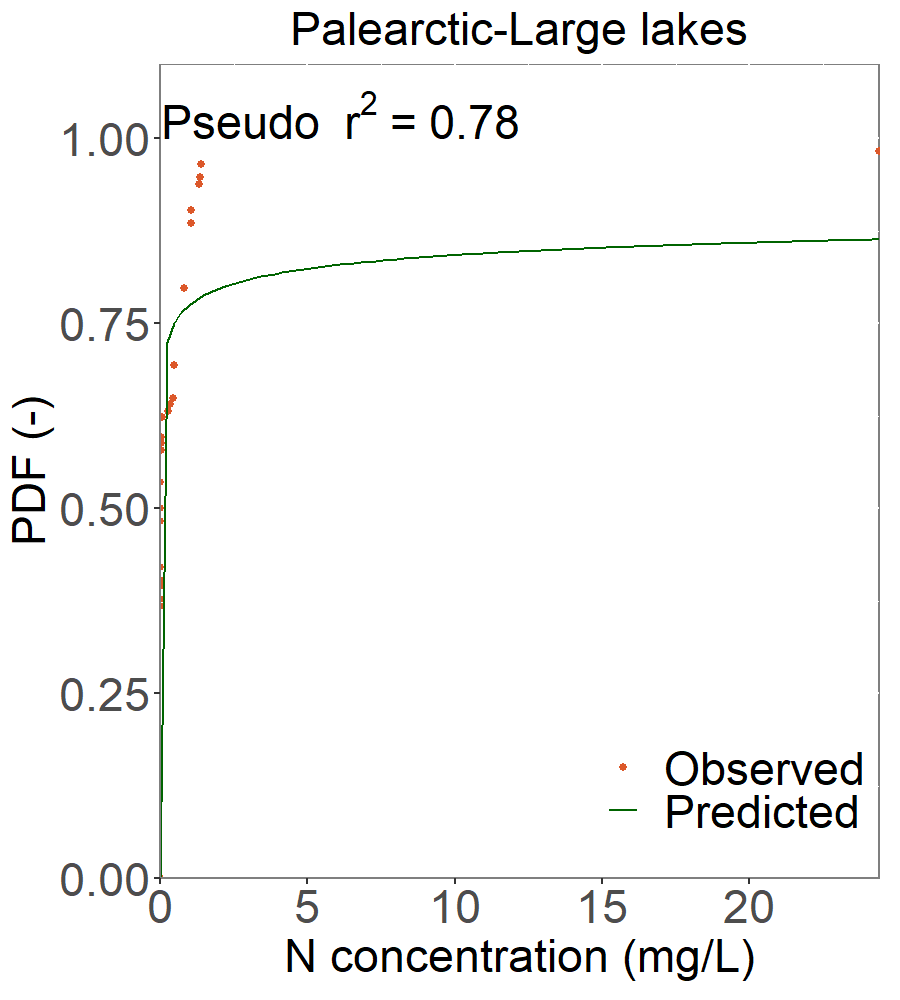

Supplement: Supplementary file 3 — es2c09333_si_003.zip [file es2c09333_si_003.zip › SSD_RMHT/Palearctic-Large lakes.tif]

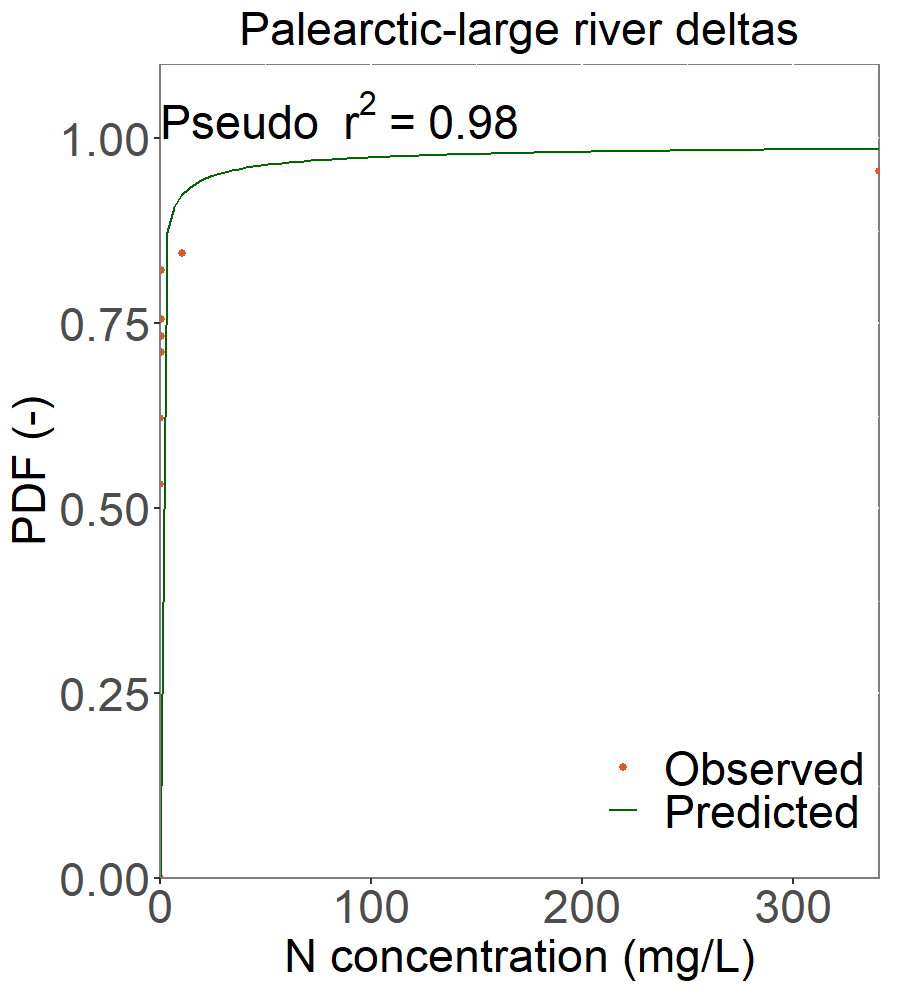

Supplement: Supplementary file 3 — es2c09333_si_003.zip [file es2c09333_si_003.zip › SSD_RMHT/Palearctic-large river deltas.tif]

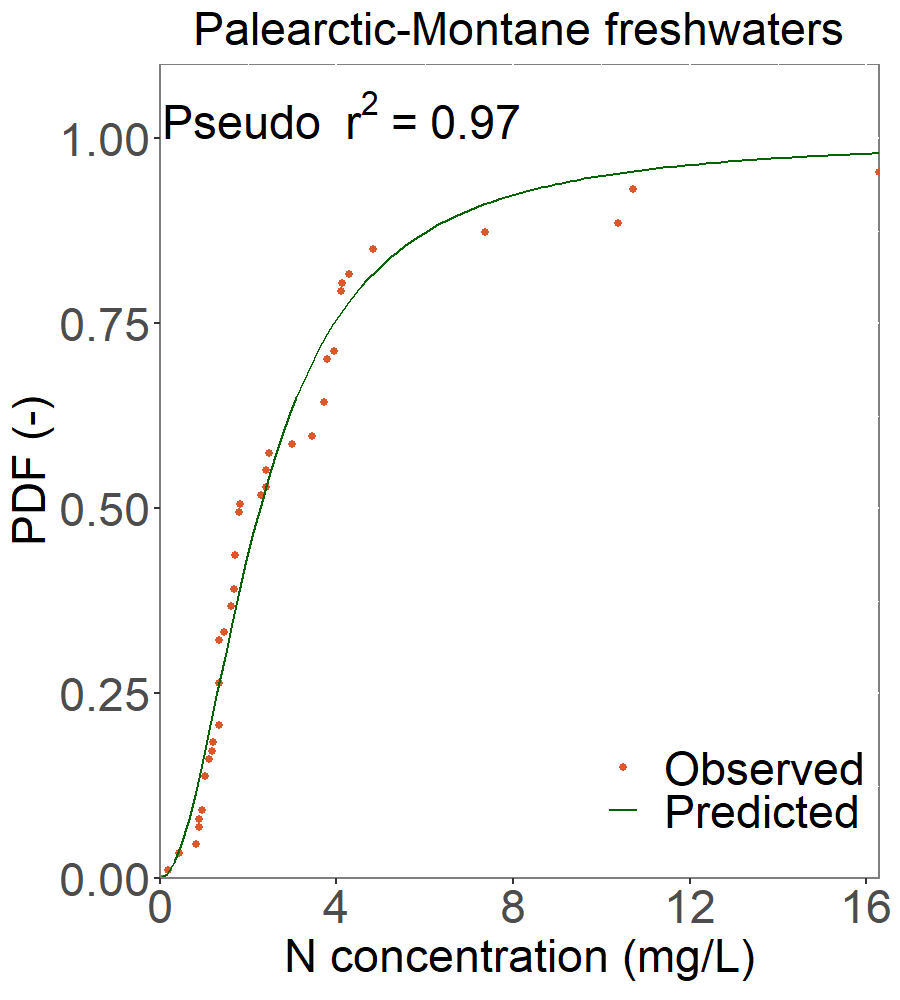

Supplement: Supplementary file 3 — es2c09333_si_003.zip [file es2c09333_si_003.zip › SSD_RMHT/Palearctic-Montane freshwaters.tif]

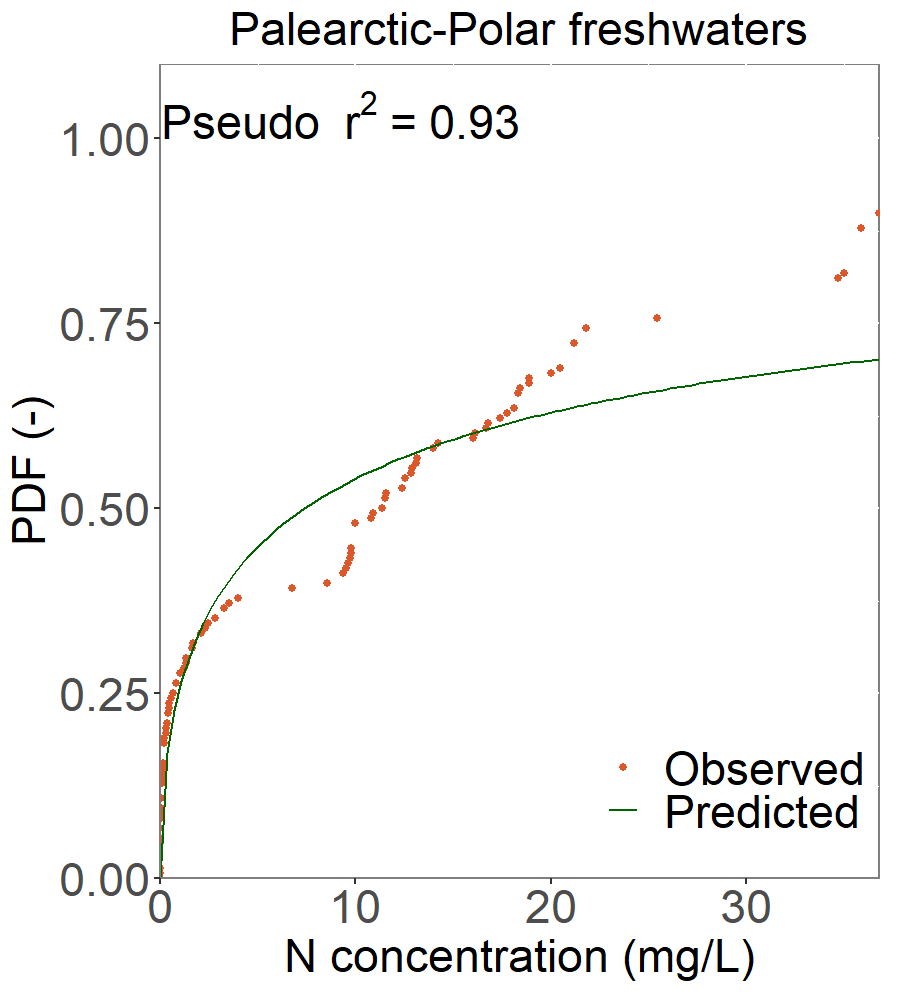

Supplement: Supplementary file 3 — es2c09333_si_003.zip [file es2c09333_si_003.zip › SSD_RMHT/Palearctic-Polar freshwaters.tif]

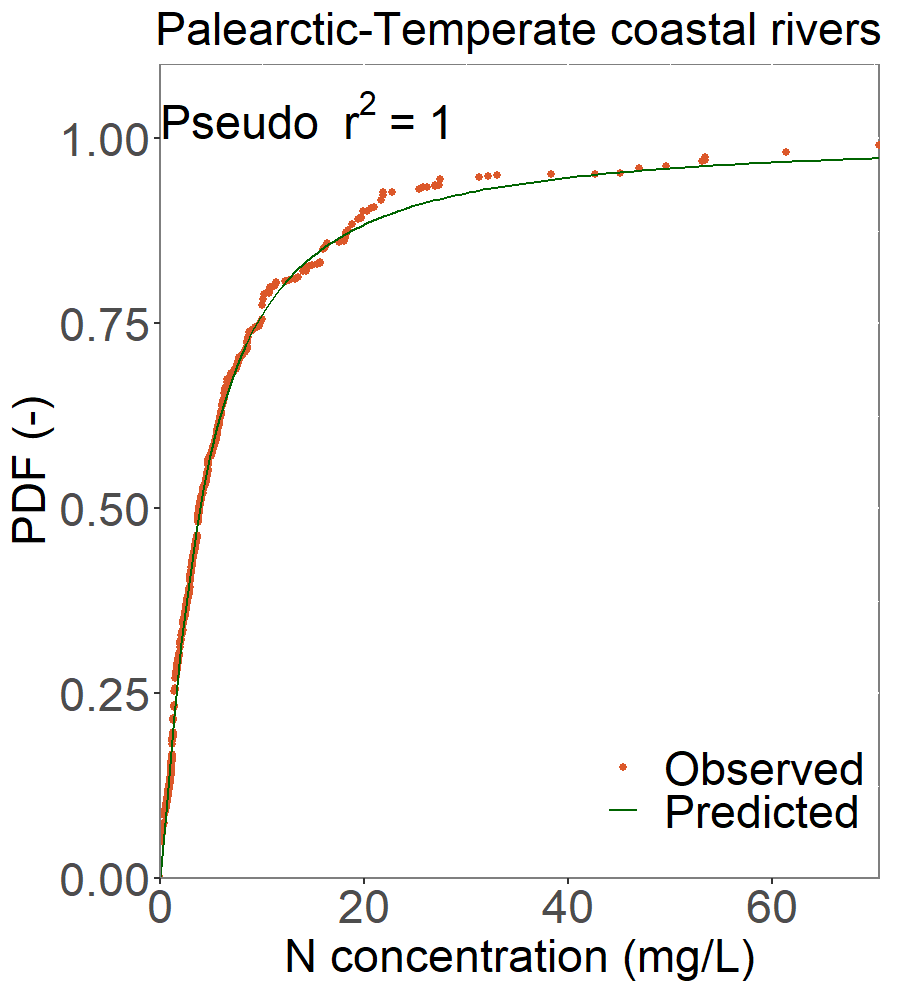

Supplement: Supplementary file 3 — es2c09333_si_003.zip [file es2c09333_si_003.zip › SSD_RMHT/Palearctic-Temperate coastal rivers.tif]

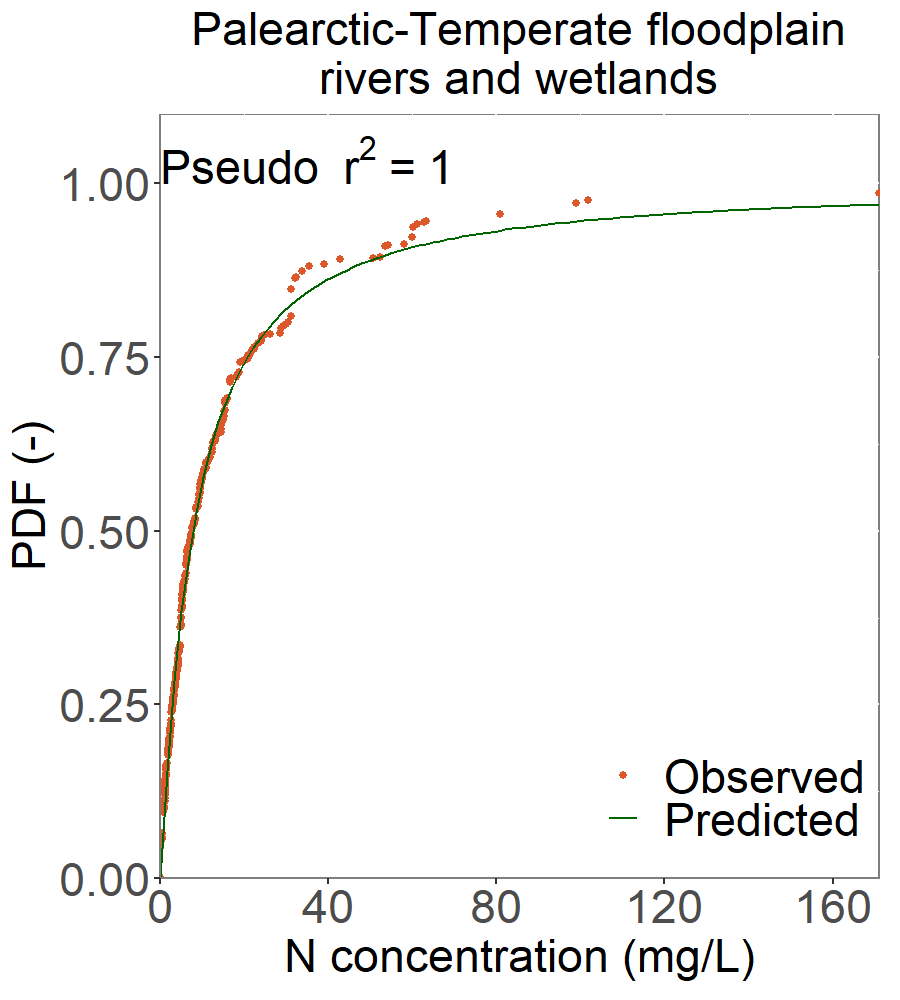

Supplement: Supplementary file 3 — es2c09333_si_003.zip [file es2c09333_si_003.zip › SSD_RMHT/Palearctic-Temperate floodplain rivers and wetlands.tif]

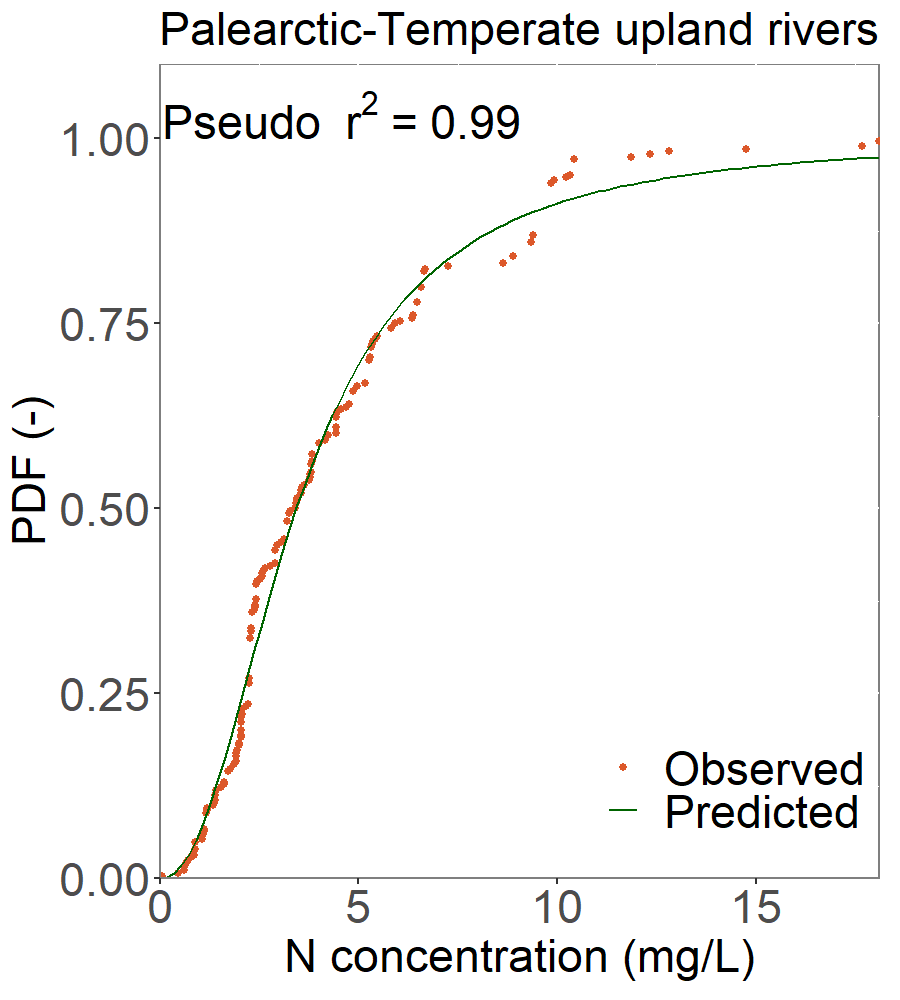

Supplement: Supplementary file 3 — es2c09333_si_003.zip [file es2c09333_si_003.zip › SSD_RMHT/Palearctic-Temperate upland rivers.tif]

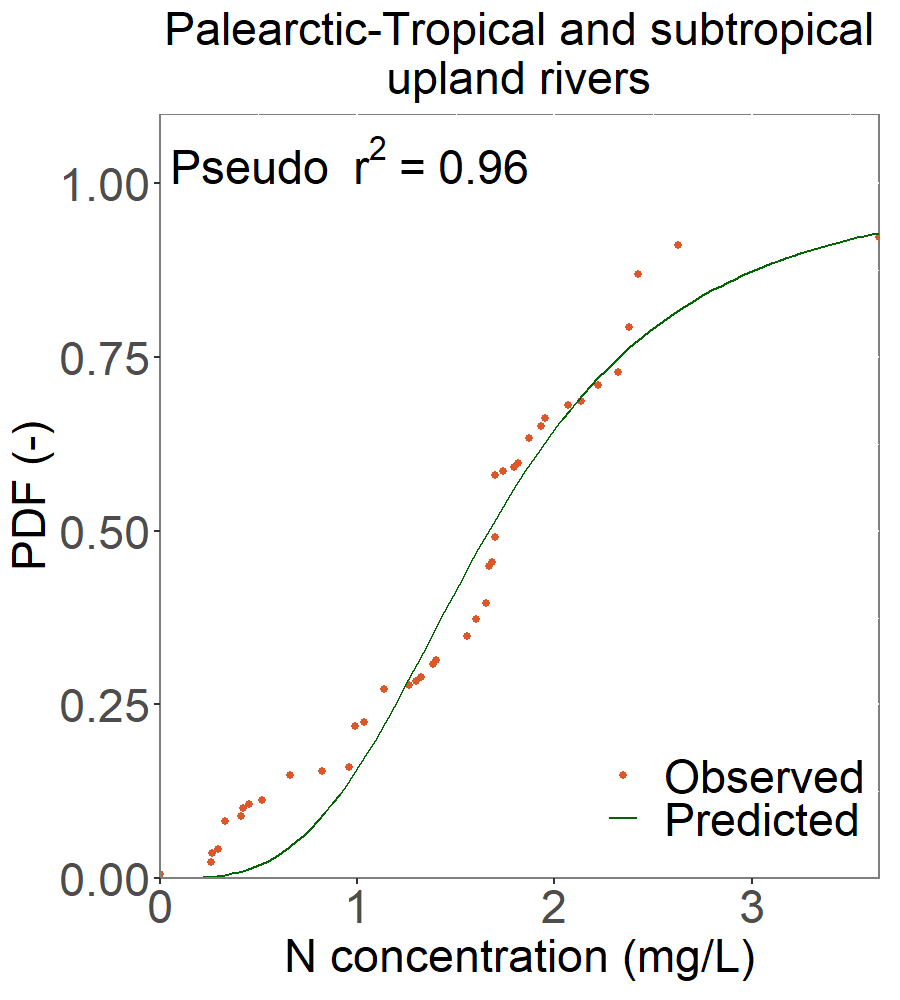

Supplement: Supplementary file 3 — es2c09333_si_003.zip [file es2c09333_si_003.zip › SSD_RMHT/Palearctic-Tropical and subtropical upland rivers.tif]

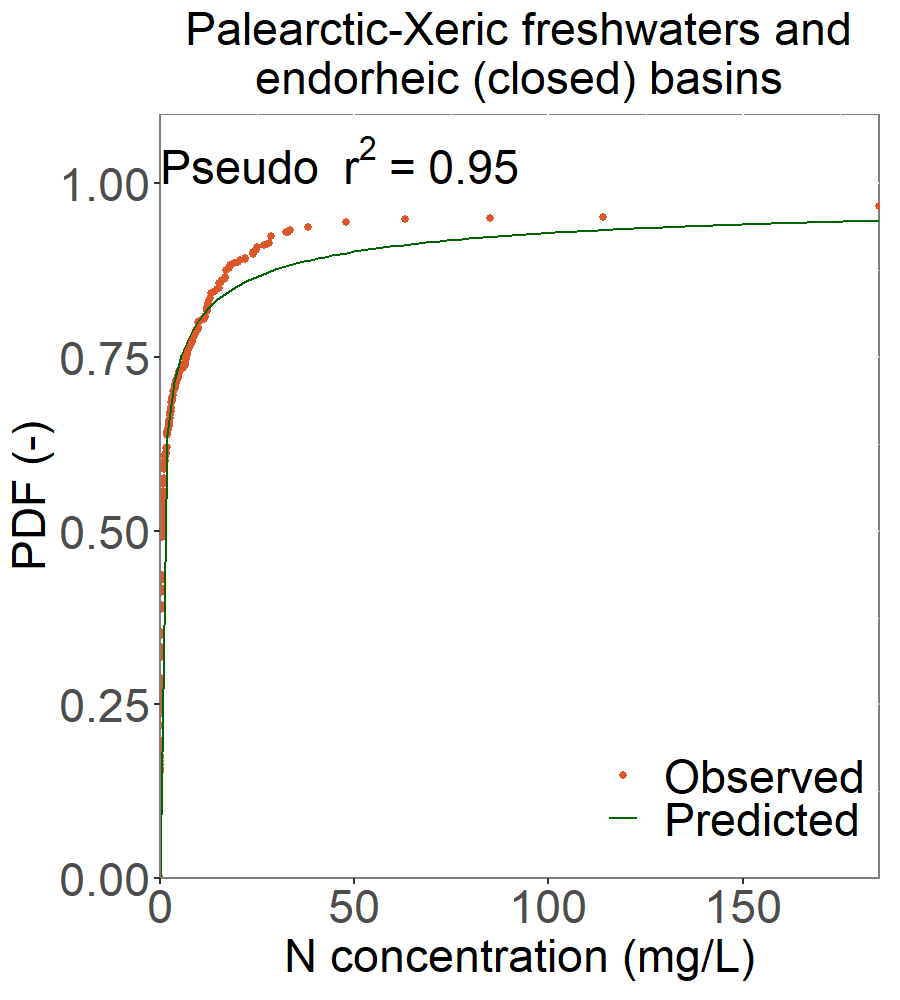

Supplement: Supplementary file 3 — es2c09333_si_003.zip [file es2c09333_si_003.zip › SSD_RMHT/Palearctic-Xeric freshwaters and endorheic (closed) basins.tif]
